# A database for deliquescence and efflorescence relative humidities of compounds with atmospheric relevance

**DOI:** 10.1016/j.fmre.2021.11.021

**Published:** 2021-12-02

**Authors:** Chao Peng, Lanxiadi Chen, Mingjin Tang

**Affiliations:** a State Key Laboratory of Organic Geochemistry, Guangdong Key Laboratory of Environmental Protection and Resources Utilization, and Guangdong-Hong Kong-Macao Joint Laboratory for Environmental Pollution and Control, Guangzhou Institute of Geochemistry, Chinese Academy of Sciences, Guangzhou 510640, China; b CAS Center for Excellence in Deep Earth Science, Guangzhou 510640, China; c University of Chinese Academy of Sciences, Beijing 100049, China

**Keywords:** Deliquescence, Efflorescence, Relative humidity, Hygroscopicity, Temperature dependence

## Abstract

Deliquescence relative humidity (DRH) and efflorescence relative humidity (ERH), the two parameters that regulate phase state and hygroscopicity of substances, play important roles in atmospheric science and many other fields. A large number of experimental studies have measured the DRH and ERH values of compounds with atmospheric relevance, but these values have not yet been summarized in a comprehensive manner. In this work, we develop for the first-of-its-kind a comprehensive database which compiles the DRH and ERH values of 110 compounds (68 inorganics and 42 organics) measured in previous studies, provide the preferred DRH and ERH values at 298 K for these compounds, and discuss the effects of a few key factors (e.g., temperature and particle size) on the measured DRH and ERH values. In addition, we outline future work that will broaden the scope of this database and enhance its accessibility.

## Graphical abstract

**Figure:**
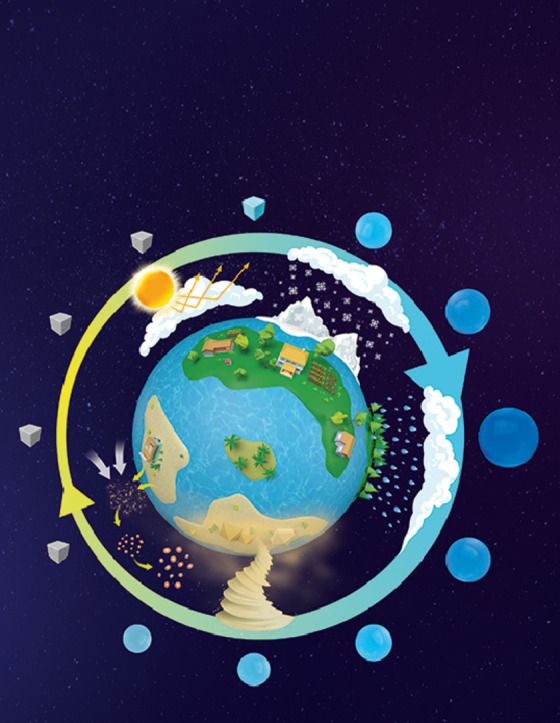

## 1 Introduction

Hygroscopicity is one of the most important physicochemical properties of substances as it regulates their interactions with water vapor under sub- and super-saturated conditions [1], [2], [3]. The hygroscopicity of aerosol particles is of critical importance in atmospheric science due to its effects on the environment and climate. Under a subsaturated condition, such as when the relative humidity (RH) is <100%, aerosol particles absorb or adsorb water vapor from the surrounding environment due to hygroscopicity, leading to changes in particle size and mass and thus affecting their optical and radiative properties [1,4,5]. Under a supersaturated condition (RH>100%), hygroscopicity is closely related to cloud condensation [[1], [2], [3],^6^,^7^] and ice nucleation activities [3,[8], [9], [10], [11]] of aerosol particles, thereby influencing their potential to be activated to cloud droplets and ice crystals. Furthermore, the liquid water contents of aerosol particles will increase with increasing RH due to hygroscopicity, causing changes in other important physicochemical properties of aerosol particles, such as the phase state [12], [13], [14], [15], acidity [16,17], and heterogeneous and multiphase reactivity [18], [19], [20], [21].

Hygroscopicity is also of great interest in many other fields [22], such as thermodynamics, chemical engineering, food and pharmaceutical science, and earth and space science. It determines phase transitions (deliquescence and efflorescence) of hygroscopic compounds at different RHs [12], and water activities and thermodynamics of aqueous solutions are directly related to the hygroscopic properties of solutes [23]. NaCl, a widely used salt in chlor-alkali industry, is crystalline at <75% RH and will be transformed to an aqueous electrolyte solution at >75% RH. In addition, its hygroscopicity significantly impacts the production of downstream chemicals, such as NaOH, H_2_ and Cl_2_ [24], [25], [26]. Hygroscopicity also affects storage and transportation in the food industry. For example, deliquescence will accelerate the degradation of labile food ingredients and decrease the stability of powdered food [27,28]. It plays key roles in the chemical and physical stability of pharmaceuticals, and water uptake by drug powders significantly determines their quality, safety, and efficacy [29], [30], [31], [32], [33], [34], [35], [36], [37], [38]. Furthermore, in atomizing inhalation treatments, transport and deposition of pharmaceutical ingredients in the human upper airway and lung are closely related to the hygroscopicity of drug aerosols [32,35,37,[39], [40], [41], [42], [43], [44], [45], [46]]. It is currently believed that one prerequisite for habitability is the existence of liquid water [47,48]. Although pure liquid water is thermodynamically unstable in the hyper arid environments found on Earth, Mars and probably other planets, highly hygroscopic materials such as chlorates and perchlorates can take up water even at RHs significantly lower than 100%, leading to the formation of aqueous solutions and liquid water [49], [50], [51], [52], [53], [54], [55].

**Fig. 1:**
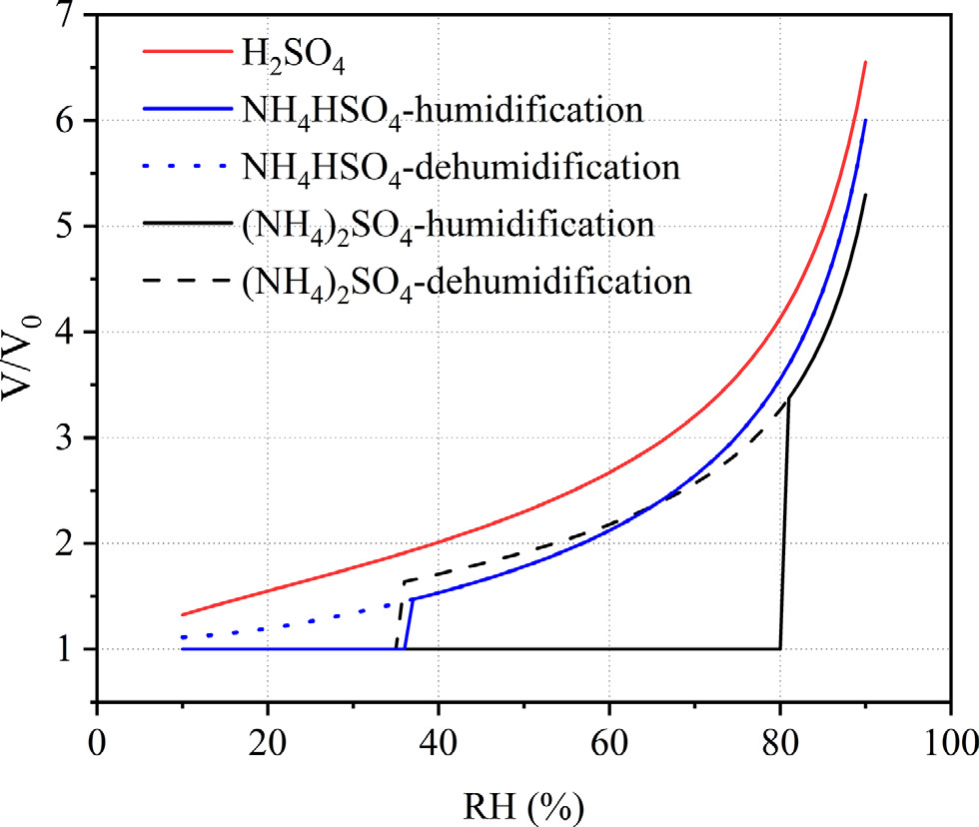
**Relative volume change of (NH_4_)_2_SO_4_, NH_4_HSO_4_ and H_2_SO_4_ particles at 298 K as a function of RH**. V is the particle volume at a given RH and V_0_ is the volume of the dry particle. The data used in the figure were produced using the extended aerosol inorganics model (E-AIM, http://www.aim.env.uea.ac.uk/aim/aim.php) [56].

Inorganic salts, such as (NH_4_)_2_SO_4_, are solid at very low RHs. As RH increases, a particle remains solid until it reaches a specific value (∼80% for (NH_4_)_2_SO_4_ at 298 K) at which the solid particle will abruptly take up large amounts of water vapor and be transformed to a saturated droplet (Fig. 1). This solid-to-liquid phase transition is called deliquescence, and deliquescence relative humidity (DRH) is defined as the RH at which deliquescence takes place. As RH further increases, the deliquesced particle will take up more water vapor, in order to maintain thermodynamic equilibrium between water vapor and aqueous water in the solution.

In contrast, water gradually evaporates from the aqueous particle as RH decreases. However, the aqueous particle will not be transformed to a solid particle when RH is decreased to DRH, and instead it will exist as a supersaturated droplet. This is because supersaturation is essential for an aqueous particle to overcome the excess energy barrier (larger than the Gibbs free energy of a crystalline solid) to form nucleation germs that will induce homogeneous nucleation and efflorescence transitions. When the RH is reduced to a specific RH that is lower than the DRH, efflorescence will occur and the supersaturated droplet will be transformed to a solid particle. The RH at which efflorescence occurs is called the efflorescence relative humidity (ERH). Hysteresis between deliquescence and efflorescence has been observed for many compounds, and the phase state of an aerosol particle is determined not only by the current RH but also by the RH history it has experienced.

It should be emphasized that not all the single-component particles exhibit well-defined deliquescence and efflorescence (Fig. 1). For example, sulfuric acid (H_2_SO_4_), an extremely hygroscopic material, takes up or losses water continuously as RH changes without deliquescence or efflorescence. As a result, H_2_SO_4_ particles always stay in a liquid state. NH_4_HSO_4_ displays a different phase transition when compared to (NH_4_)_2_SO_4_ and H_2_SO_4_: solid NH_4_HSO_4_ particles will be deliquesced to form droplets when RH is increased to 30–41%, whereas NH_4_HSO_4_ droplets will not effloresce to form solid particles even when RH becomes very low (down to 10%). Therefore, once deliquesced, NH_4_HSO_4_ particles will remain as aqueous droplets.

The important roles of databases that summarize thermodynamic and kinetic data have been well recognized in atmospheric chemistry [57,58]. Currently databases are available for kinetic and photochemical data [59], [60], [61], Henry's law constants [62], gas phase diffusion coefficients [63], [64], [65], and etc. Despite the importance of DRH and ERH in many fields, up to now these values have not yet been compiled or summarized in a comprehensive manner. Martin [12] discussed phase transition processes of atmospheric particles, and summarized DRH and ERH values of selected species (<20 compounds) at 298 K. Very recently, Ma et al. [66] reviewed efflorescence kinetics of atmospherically relevant particles, and summarized the measured ERH for seven inorganic compounds, including NaCl, KCl, (NH_4_)_2_SO_4_, Na_2_SO_4_, MgSO_4_, NaNO_3_, and NH_4_NO_3_.

In this work, we have developed for the first time a comprehensive database in which DRH and ERH values measured in previous studies are compiled. This database summarizes DRH and ERH of a total of 110 compounds, including 68 inorganics and 42 organics. It is mainly focused on compounds with atmospheric relevance, but also includes certain inorganic compounds relevant for earth and planetary sciences, such as perchlorates [49,50,53,54]. The motivations for the development of such a database are (1) to provide a comprehensive summary of measured DRH and ERH values and (2) to identify knowledge gaps that will stimulate measurements of DRH and ERH values in future work. In addition, many studies which measured DRH or ERH values of some compounds also reported their hygroscopic growth factors (or water-to-solute ratios) as a function of RH (or water activities). Although our present work does not compile growth factors or water-to-solute ratios for compounds included, it can serve as a starting point (i.e., as a collection of references) for people who are interested in growth factors or water-to-solute ratios measured by previous studies.

## 2 Techniques used to measure deliquescence and efflorescence relative humidities

A large number of experimental techniques have been employed to measure DRH and ERH (and hygroscopicity in general) of compounds with atmospheric relevance. Samples under investigation broadly include four types, such as bulk solutions, samples deposited on substrates, levitated single particles, and aerosol particles [22]. These techniques were reviewed and discussed in a recent paper [22], and therefore, here we only describe in brief four techniques widely used to measure DRH and ERH.

The nonisopiestic method is a bulk solution-based technique and measures RH of the air in equilibrium with an electrolyte solution with a given concentration (i.e., a given water-to-solute ratio), and DRH is equal to the RH of the air in equilibrium with the saturated solution. This method has been used to measure DRH values of many electrolytes and their water-to-solute ratios as a function of water activity [67], [68], [69], [70]. For example, it was used to measure DRH of 28 electrolytes (including sulfates, nitrates and halides) as a function of temperature (273–373 K) [70], and reported DRH values ranged from ∼3% to 98%. This method is simple and accurate, but it cannot be used to determine ERH values.

Hygroscopicity can be quantified by measuring sample mass as a function of RH, using a RH-controlled balance [71,72] or a vapor sorption analyzer [73,74], and DRH is equal to the RH at which an abrupt increase in sample mass is observed during humidification. For example, a vapor sorption analyzer was used to determine DRH values of six inorganic compounds (CaBr_2_, MgCl_2_·6H_2_O, Mg(NO_3_)_2_·6H_2_O, NaCl, (NH_4_)_2_SO_4_ and KCl) at different temperatures (278–303 K) [74], and the measured values (<20% to >85%) agreed well with those reported by Greenspan [70]. Similar to the nonisopiestic method, this method cannot be used to determine ERH values either.

Electrodynamic balance (EDB) is a single-particle levitation technique widely employed to study aerosol hygroscopicity [75], [76], [77]. In a typical EDB measurement, a single particle (typically 1–100 μm in diameter) can be levitated in the EDB chamber by adjusting the alternating current (AC) and direct current (DC) electric fields surrounding the particle, and relative mass change of the particle (for example, due to condensation or evaporation of water) is equal to the change in the DC voltage needed to levitate the particle. The temperature and RH in the EDB chamber can be regulated, and the RH at which an abrupt increase (or decrease) in the relative mass of the sample occurs during humidification (or dehumidification) is equal to its DRH (or ERH). Tang and Munkelwitz [77] employed this technique to determine the DRH values of six inorganic salts ((NH_4_)_2_SO_4_, Na_2_SO_4_, NaNO_3_, NH_4_NO_3_, KCl and NaCl) as a function of temperature (278–308 K), and found that the temperature dependence of their DRH values could be well described by the Clausius-Clapeyron equation. In addition, EDB has been used to measure ERH values of many compounds with atmospheric relevance [78], [79], [80].

The humidity tandem differential mobility analyzer (H-TDMA), which measures the mobility diameters of aerosol particles at different RHs, has been extensively used to investigate hygroscopic properties of aerosol particles [81], [82], [83], [84]. The measured DRH (or ERH) is defined as the RH at which a sudden increase (or decrease) in aerosol mobility diameter takes place during humidification (or dehumidification). An H-TDMA was employed to measure DRH values and hygroscopic properties of 10 water-soluble carboxylic salts at 293 K [85]: the DRH was determined to be 39–42% for sodium acetate, 81–82% for sodium pyruvate, and >90% for sodium oxalate and ammonium oxalate, while the other six salts (sodium malonate, sodium succinate, sodium maleate, ammonium tartrate, sodium tartrate and humic acid sodium salts) displayed continuous water uptake when RH increased from 5% to 90%. This technique has also been widely used to measure ERH values of particles with atmospheric relevance [86], [87], [88], [89], [90], [91], [92].

## 3 Overview of the database

The database we have developed provides a comprehensive compilation of DRH and ERH of 110 compounds measured at different temperatures in previous work. These species were classified into 11 groups of inorganic species (including six sulfates, three bisulfates, nine nitrates, three fluorides, 12 chlorides, eight bromides, six iodides, four chlorates, three iodates, 10 perchlorates and four carbonates) and four groups of organic species (including five methanesulfonates, 12 monocarboxylic salts, 13 dicarboxylic acids and 12 dicarboxylic salts). For each compound, their DRH and ERH values measured by previous studies at different temperatures are summarized in individual tables and also briefly discussed (in supplementary materials). This document to a large extent adopts the format used by the International Union of Pure and Applied Chemistry Task Group on Atmospheric Chemical Kinetic Data Evaluation (https://iupac-aeris.ipsl.fr/). In addition, we have attempted to provide the preferred DRH and ERH values at 298 K for compounds included in the database, and these values are summarized in Table 1.

**Table 1:** **Preferred deliquescence relative humidity (DRH) and efflorescence relative humidity (ERH) at 298 K.** DRH and ERH values are given in %.

Compounds	DRH	ERH	Compounds	DRH	ERH
**Sulfates**					
(NH_4_)_2_SO_4_	78–82	30–48	Li_2_SO_4_	82–85	
Na_2_SO_4_	82–87	55–59	K_2_SO_4_	95–99	58–62
MgSO_4_			MgSO_4_∙7H_2_O	87–89	
**Bisulfates**					
NH_4_HSO_4_	30–41		NaHSO_4_		
KHSO_4_					
**Nitrates**					
NH_4_NO_3_	60–66	25–36	LiNO_3_	41–44	
LiNO_3_∙3H_2_O	47		NaNO_3_	71–76	35–45
KNO_3_	92–94		Ca(NO_3_)_2_		
Ca(NO_3_)_2_∙4H_2_O	49–56		Mg(NO_3_)_2_	52–55	
Mg(NO_3_)_2_∙6H_2_O	49–54				
**Fluorides**					
NaF	94–97	69–77	KF	30–32	8–13
KF∙2H_2_O	34				
**Chlorides**					
NH_4_Cl	76–79	45	LiCl	9–12	1–4
LiCl∙H_2_O	11		NaCl	73–77	41–51
KCl	83–86	50–60	CaCl_2_	27–31	
CaCl_2_∙2H_2_O	18–19		CaCl_2_∙4H_2_O	23	
CaCl_2_∙6H_2_O	28–29		MgCl_2_	31–35	<1.5
MgCl_2_∙4H_2_O	15–17	6–8	MgCl_2_∙6H_2_O	30–35	9–11
**Bromides**					
NH_4_Br	80		LiBr	6–7	
LiBr∙2H_2_O	7		NaBr	56–60	21–30
NaBr∙2H_2_O	57–58		KBr	80–83	51–56
CaBr_2_	14–18		MgBr_2_	31	
**Iodides**					
NH_4_I	73		LiI	17–18	
LiI∙3H_2_O	19		NaI	37–39	8–11
NaI∙2H_2_O	39		KI	65–70	36–50
**Chlorates**					
NaClO_3_	72–74		KClO_3_	97–98	
Ca(ClO_3_)_2_∙2H_2_O	26–27		Mg(ClO_3_)_2_∙6H_2_O	20–21	
**Iodates**					
I_2_O_5_	78–81		HIO_3_	82–85	
KIO_3_	93–94				
**Perchlorates**					
NH_4_ClO_4_	94–95		LiClO_4_		3
LiClO_4_∙3H_2_O	67	40	NaClO_4_	38–49	15–23
NaClO_4_∙H_2_O	44–48		KClO_4_	>99	
Ca(ClO_4_)_2_			Ca(ClO_4_)_2_∙4H_2_O	16–17	
Mg(ClO_4_)_2_	50–51	10–18	Mg(ClO_4_)_2_∙6H_2_O	40–41	
**Carbonates**					
LiCO_3_	>85		Na_2_CO_3_	>60	39–60
K_2_CO_3_	43–50	9–25	K_2_CO_3_∙2H_2_O	42–43	
**Methanesulfonates**					
CH_3_SO_3_NH_4_			CH_3_SO_3_Na	65–73	48–52
CH_3_SO_3_K	70–75		Ca(CH_3_SO_3_)_2_	70–75	
Mg(CH_3_SO_3_)_2_					
**Monocarboxylic salts**					
HCOONa	50–62	26–37	Ca(HCOO)_2_	>95	
Mg(HCOO)_2_∙2H_2_O	>95		CH_3_COONH_4_	57–68	
CH_3_COOLi	68		CH_3_COONa	39–48	34–43
CH_3_COOK	18–23				
Ca(CH_3_COO)_2_	83–84		Ca(CH_3_COO)_2_∙H_2_O	89–92	
Mg(CH_3_COO)_2_	65–74	43–44	Mg(CH_3_COO)_2_∙4H_2_O	70–73	
sodium pyruvate	71–84	56–60			
**Dicarboxylic acids**					
Oxalic acid	>90		Malonic acid	65–76	<22
Succinic acid	>90	50–60	Glutaric acid	80–93	18–43
Adipic acid	>88		Pimelic acid	>90	51–53
Suberic acid	>90	>85	Azelaic acid	>86	>85
Maleic acid	71–99	18–51	D,L-malic acid	78–81	
L-malic acid	56–62		Tartaric acid	77–78	
Phthalic acid	>88				
**Dicarboxylic salts**					
Ammonium oxalate	>90	53–56	Ammonium oxalate monohydrate	>95	
Sodium oxalate	>90	72–84	Potassium oxalate	84–86	49–56
Calcium oxalate	>90		Calcium oxalate monohydrate	>95	
Sodium malonate			Sodium malonate monohydrate	65–66	
Sodium succinate	63–79	46–59			
Sodium maleate					
Ammonium tartrate	85–86		Sodium tartrate		

As shown in Table 1, the preferred DRH values at 298 K are provided for 99 compounds among the 110 compounds included in the database. Such values are not provided for the other 11 compounds, including MgSO_4_, NaHSO_4_, KHSO_4_, Ca(NO_3_)_2_, LiClO_4_, Ca(ClO_4_)_2_, CH_3_SO_3_NH_4_, Mg(CH_3_SO_3_)_2_, sodium malonate, sodium maleate, and sodium tartrate. This is due to one or more of the following reasons: 1) no DRH values have been measured for NaHSO_4_, KHSO_4_ and LiClO_4_; 2) several compounds, including CH_3_SO_3_NH_4_, Mg(CH_3_SO_3_)_2_, sodium malonate, sodium maleate and sodium tartrate, take up water vapor continuously as RH increases, and thus they do not exhibit distinctive deliquescence; and 3) the DRH values measured by different studies displayed large discrepancies for MgSO_4_, Ca(NO_3_)_2_ and Ca(ClO_4_)_2_, precluding us from suggesting preferred DRH values for these compounds.

The interaction of water vapor with compounds of atmospheric relevance has been much less investigated during dehumidification than humidification, and the number of compounds for which the ERH values have been measured is much smaller than the number of compounds for which the DRH values have been measured. This is the major reason why we were able to provide preferred ERH values at 298 K for only 40 compounds in Table 1; for comparison, the preferred DRH values are provided for 99 compounds. There are also two other reasons why the preferred ERH values at 298 K are not given: (1) for some compounds, such as NaHSO_4_ and Ca(NO_3_)_2_, water vapor evaporates continuously as RH decreases, and thus these compounds do not have well-defined ERH values; (2) compared to the DRH values, large discrepancies for ERH values measured by different studies occurred more frequently, and therefore we were unable to provide the preferred ERH values for these compounds. Below we discuss how the measured DRH (Section 3.1) and ERH values (Section 3.2) can be affected by environmental parameters and experimental conditions.

### 3.1 Deliquescence relative humidity

#### 3.1.1 Temperature

Deliquescence consists of two processes: condensation of water vapor on the solute followed by dissolution of the solute. The overall enthalpy change of the deliquescence (Δ*H*) can be expressed by Eq. 1:

(1) $$\Delta H=n\cdot \Delta H_{s}-\Delta H_{v}$$

where *n* is the solubility of the solute in water (moles of solute per mole of water), Δ*H_s_* is the enthalpy of dissolution (J mol^−1^), and Δ*H_v_* is the heat of water vaporization (J mol^−1^). As the Clausius-Clapeyron equation can be used to describe vapor pressures of pure water and over the solution at different temperatures, the dependence of DRH on temperature can be obtained:

(2) $$\frac{d\text{ln}\left(\text{DRH}/100\right)}{dT}=-n\frac{\Delta H_{s}}{RT^{2}}$$

where *T* is the temperature (K) and *R* is the universal gas constant (8.314 J mol^−1^ K^−1^). If we assume that Δ*H*_s_, the enthalpy of dissolution, does not vary with temperature, the integration of Eq. 2 gives Eq. 3:

(3) $$\text{DRH}\left(T\right)=\text{DRH}\left(298\right)\cdot \text{exp}\left\{\frac{\Delta H_{s}}{R}\left[A\left(\frac{1}{T}-\frac{1}{298}\right)-B\text{ln}\frac{T}{298}-C\left(T-298\right)\right]\right\}$$

where DRH(*T*) and DRH (298) are the DRH values at *T* and 298 K, respectively. The three parameters, *A, B* and *C*, describe the dependence of solubility (*n*) on temperature, as expressed by Eq. 4:

(4) $$n=A+BT+CT^{2}$$

If we further assume constant solubility (i.e., *B*=*C*=0), Eq. 3 can be simplified to Eq. 5, which is widely used to approximate the dependence of DRH on temperature due to its simplicity.

(5) $$\text{DRH}\left(T\right)=\text{DRH}\left(298\right)\cdot \text{exp}\frac{A\cdot \Delta H_{s}}{R}\left(\frac{1}{T}-\frac{1}{298}\right)$$

Further information on how to derive Eqs. 3 and 5 can be found elsewhere [77,93,94]. The temperature dependence of DRH is determined by the overall enthalpy change of deliquescence (Δ*H*). DRH decreases with temperature if Δ*H* is positive and increases with temperature if Δ*H* is negative.

**Fig. 2:**
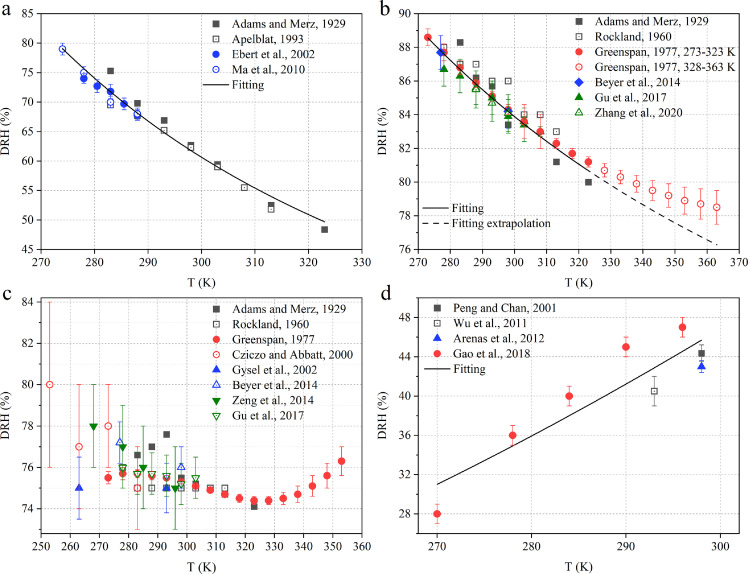
**The DRH values as a function of temperature:** (a) NH_4_NO_3_ [67,[95], [96], [97]]; (b) KCl [67,69,70,73,74,98]; (c) NaCl [67,69,70,73,74,81,99,100]; and (d) CH_3_COONa [85,[101], [102], [103]]. The black solid lines represent fittings using Eq. 5.

It shows DRH values of NH_4_NO_3_, KCl, NaCl and CH_3_COONa at different temperatures measured by previous studies (Fig. 2). As displayed in Fig. 2a, the DRH values of NH_4_NO_3_ show a negative dependence on temperature, decreasing from 79 ± 1% at 274 K to 48.4% at 323 K, and the temperature dependence can be well approximated by Eq. 5. Similarly, the DRH values of KCl also decrease with temperature, from 88.6 ± 0.5% at 273 K to 78.5 ± 1.0% at 363 K. Nevertheless, as shown in Fig. 2b, Eq. 5 can only describe the temperature dependence of its DRH values for temperature between 283 and 323 K, while the DRH values measured at 328–363 K [70] are significantly larger than those extrapolated from the fitted curve using Eq. 5. This underscores that extrapolation using Eq. 5 may not always be valid and should be conducted with caution.

The DRH values were determined to be 73–84% at 253–353 K for NaCl (Fig. 2c), showing no obvious dependence on temperature. In addition, the DRH values showed a positive temperature dependence for CH_3_COONa (Fig. 2d), increasing from 27–29% at 270 K to 43–45% at 298 K, and the positive dependence can be well approximated using Eq. 5.

#### 3.1.2 Hydration form

It was argued in a previous study [104] that DRH values would be different for anhydrous and hydrated forms of the same compound, and this was supported by some previous measurements. For example, the DRH values at 223–253 K were found to be <20% for CaCl_2_·2H_2_O and 50–80% for CaCl_2_·6H_2_O [105]. However, one may argue from a thermodynamic view that DRH values should be identical for anhydrous and hydrated forms. Take MgCl_2_ and MgCl_2_·6H_2_O as an example: once deliquesced, MgCl_2_ and MgCl_2_·6H_2_O will both be transformed to saturated MgCl_2_ solutions with identical water activities; as a result, MgCl_2_ and MgCl_2_·6H_2_O should have the same DRH value at a given temperature. This was also supported by some previous experimental work. For example, good agreement in DRH values was found between MgCl_2_ [69,70] and MgCl_2_·6H_2_O [74,106], and between Ca(NO_3_)_2_ [69,107] and Ca(NO_3_)_2_·4H_2_O [67,106,108]. More experimental and theoretical work is required to better understand the effects of hydration forms on DRH values.

#### 3.1.3 Particle size

Several previous studies measured DRH values as a function of particle size [86,87,91,[109], [110], [111], [112], [113], [114], [115], [116]]. The RH on the curved surface of a droplet can be described by the Köhler theory [117,118]:

(6) $$RH=a_{w}\cdot K_{e}$$

(7) $$K_{e}=\text{exp}\left(\frac{4M_{w}\sigma }{RT\rho_{w}d_{p}}\right)$$

where *a*_w_ is the water activity in the solution, *K_e_* is the Kelvin term which describes the increase in vapor pressure over a curved surface relative to that over a flat surface, *M_w_* is the molar mass of water (kg mol^−1^), *σ* is the surface tension of the solution (J m^−2^), *ρ_w_* is the density of water (kg m^−3^), and *d*_p_ is the droplet diameter (m). The Kelvin effect is negligible for particles larger than 100 nm [3,118], and this is supported by measurements that found no significant difference in the DRH values between supermicrometer (>1000 nm) and submicrometer (between 100 and 1000 nm) particles. However, for multiple component particles, their morphology and mixing states may vary with particle size, leading to variations in the DRH values (and also hygrosocpic properties) with particle size [113].

**Fig. 3:**
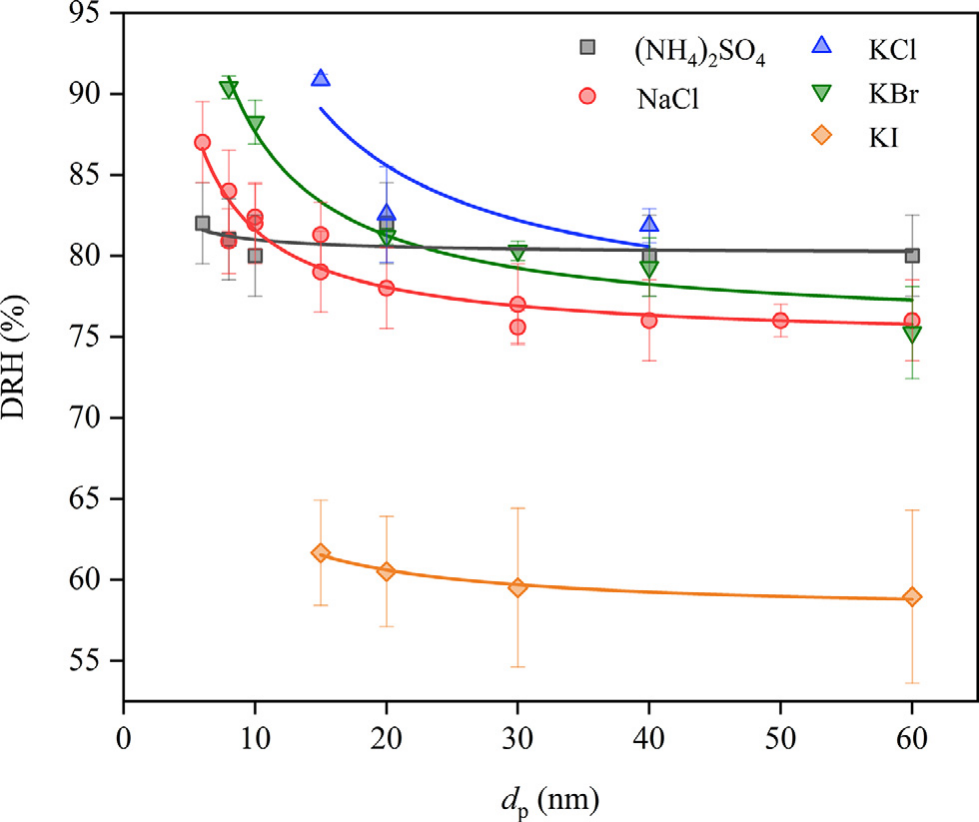
**The DRH values of (NH_4_)_2_SO_4_****[87]**, **NaCl** [86,109****]**, KCl****[91]**, **KBr****[91]**, **and KI****[91]****as a function of particle size at 298 K. The solid curves represent fittings using**Eq. 6.

The Kelvin effect becomes important and particle size plays an indispensable role in DRH for particles smaller than 100 nm. Fig. 3 displays the DRH values as a function of particle size (6–60 nm) at 298 K for (NH_4_)_2_SO_4_, NaCl, KCl, KBr and KI. It is evident that the DRH values show a negative dependence on particle size for all four compounds. For example, the DRH values of NaCl at 298 K decrease from 87 ± 2.5% at 6 nm to 76 ± 2.5% at 60 nm [86], and the dependence of DRH on particle size can be well described by Eq. 6. We also note that a differential Köhler analysis coupled with the Ostwald-Freudlich equation [112] can also describe the size dependence of DRH values.

#### 3.1.4 Initial phase states

The occurrence of deliquescence is closely related to the initial phase state of particles [90]. For example, the DRH values at 298 K were measured to be approximately 49.5%, 52.5%, 31.5%, and 28.5% for crystalline Ca(NO_3_)_2_·H_2_O, Mg(NO_3_)_2_·6H_2_O, MgCl_2_·6H_2_O and CaCl_2_·6H_2_O, respectively, while continuous hygroscopic growth was observed for Ca(NO_3_)_2_, Mg(NO_3_)_2_, MgCl_2_ and CaCl_2_ aerosol particles that were amorphous [106]. The DRH of oxalic acid at around room temperature has been widely investigated [90,[119], [120], [121], [122], [123], [124], [125]], and different deliquescence transitions were observed: DRH was measured to be >90% RH in five studies [119], [120], [121], [122], [123], while three other studies [90,124,125] suggested continuous water uptake with increase in RH. This discrepancy is attributed to the difference in initial phase states of oxalic acid particles (crystalline versus amorphous), which results from different particle diffusion drying methods. Different pretreatments of aerosol particles which were generated via atomization could also affect the measured DRH values. For example, after atomized KNO_3_ particles were heated overnight at 104 °C, they would be transformed to crystalline solids and become deliquesced at >90% RH [126]; for comparison, continuous water uptake was observed for atomized KNO_3_ particles without heating.

#### 3.1.5 Substrates used to support samples

Some studies [116,127] examined whether substrates used to support samples would affect the measured DRH values, and they found their effects to be negligible. For example, Eom et al. [127] investigated the influence of six supporting substrates on the measured DRH values of (NH_4_)_2_SO_4_, NaCl, and KCl particles using optical microscopy. The measured DRH values agreed well with theoretical values for particles deposited on hydrophobic substrates (transmission electron microscopy grids and parafilm-M); while the measured DRH values were 1–2% lower than the theoretical values for particles deposited on hydrophilic substrates (Al foil, Ag foil, silicon wafer and cover glass). The authors suggested that the observed lower DRHs were attributed to a slight increase in the Gibbs free energy for solid particles due to absorbed water on the particles and hydrophilic substrates prior to deliquescence [127]. However, such small differences might be insignificant.

### 3.2 Efflorescence relative humidity

#### 3.2.1 Temperature

**Fig. 4:**
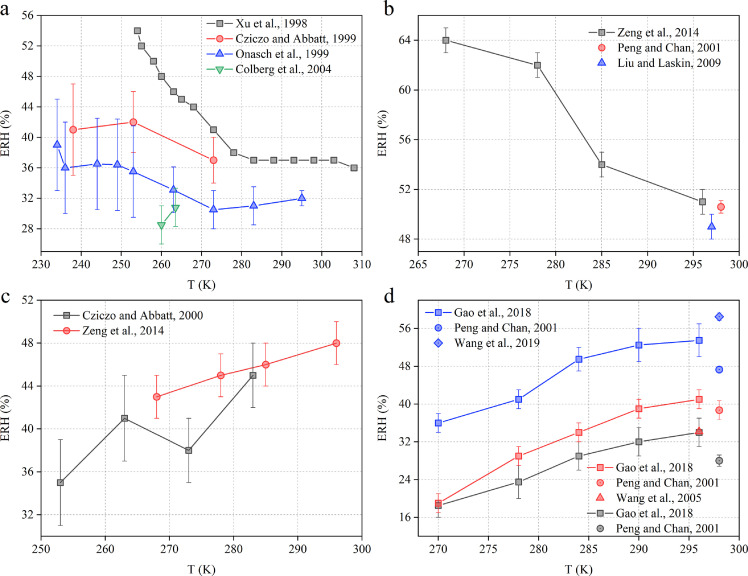
**The ERH values as a function of temperature:** (a) (NH_4_)_2_SO_4_[128], [129], [130], [131], (b) CH_3_SO_3_Na [100,101,132], (c) NaCl [99,100], and (d) sodium formate (black symbols) [101,103], sodium acetate (red symbols) [101,103,133], and sodium succinate (blue symbols) [101,103,134].

Four previous studies [128], [129], [130], [131] measured ERH values of (NH_4_)_2_SO_4_ at different temperatures. As shown in Fig. 4a, Xu et al. [128] found that it first decreased with temperature from 54% at 254 K to 37% at 283 K and did not change significantly when the temperature was further increased to 308 K; Cziczo and Abbatt [129] found that it decreased with temperature from 41 ± 6% at 238 K to 37 ± 3% at 273 K; Onasch et al. [130] suggested that it first decreased with temperature from 39.0 ± 6% at 234 K to 30.5 ± 2.5% at 273 K, and then slightly increased with temperature to 32.0 ± 1% at 295 K. Although these three studies [128], [129], [130] showed substantial discrepancies, one may conclude that ERH values of (NH_4_)_2_SO_4_ first decreased with temperature (up to ∼280 K) and then did not change significantly when the temperature was further increased. However, the fourth study [131] suggested that it increased slightly with temperature from 28.5 ± 2.5% at 260 K to 30.8 ± 2.5% at 263.5 K. The ERH values also showed a negative temperature dependence for CH_3_SO_3_Na (Fig. 4b), decreasing from 63–65% at 268 K to 50–52% at 296 K.

The ERH values of NaCl were measured at different temperatures by two previous studies [99,100] that both revealed positive dependence on temperature. As shown in Fig. 4c, ERH values of NaCl were reported to increase with temperature from 35 ± 4% at 253 K to 45 ± 3% at 283 K [99] and from 43 ± 2% at 268 K to 48 ± 2% at 296 K [100]. Gao et al. [103] measured ERH values of sodium formate, sodium acetate, and sodium succinate at different temperatures, and they revealed positive dependence of ERH values for the three compounds, similar to NaCl. As shown in Fig. 4d, ERH values were found to increase with temperature from 16–21% at 270 K to 31–37% at 296 K for sodium formate, 17–21% at 270 K to 39–43% at 296 K for sodium acetate, and 34–38% at 270 K to 50–57% at 296 K for sodium succinate.

Compared to DRH values, the temperature dependence of ERH values has been less investigated. It was revealed by previous studies that ERH values could decrease, increase, or show no significant change with an increase in temperature, similar to DRH values. To the best of our knowledge, there are no equations that can generally describe or approximate the dependence of ERH values on temperature, partially because relevant experimental studies are very limited, and measured ERH values are more uncertain when compared to measured DRH values.

#### 3.2.2 Particle size

Classical nucleation theory suggests that the expectation time (*τ*) for production of a crystalline germ at a specific temperature or relative humidity can be described by Eq. 8 [12]:

(8) $$\tau =\frac{1}{J\cdot V}$$

where *J* is the homogeneous nucleation rate (cm^−3^ s^−1^) and *V* is the aqueous solution droplet volume (cm^3^). Eq. 8 suggests that the expectation time for a single nucleation germ formation requires less time for larger particles. Therefore, nucleation germ formation is more favored for larger particles [12], and consequently the ERH values are expected to increase with particle size.

Laskina et al. [113] found that the ERH values of (NH_4_)_2_SO_4_ and NaCl at 298 K both increase with particle size: the ERH of (NH_4_)_2_SO_4_ was measured to be 37.7 ± 4.1% for 100 nm particles and 43.5 ± 2.1% for 3–10 μm particles, and the ERH of NaCl was measured to be 43.0 ± 1.0% for 100 nm particles and 52.8 ± 1.1% for 3–10 μm particles. In contrast, ERH values were determined to be 35–40% for 100 nm and 5–25 μm (NH_4_)_2_SO_4_ particles [89], showing no dependence on particle size.

**Fig 5:**
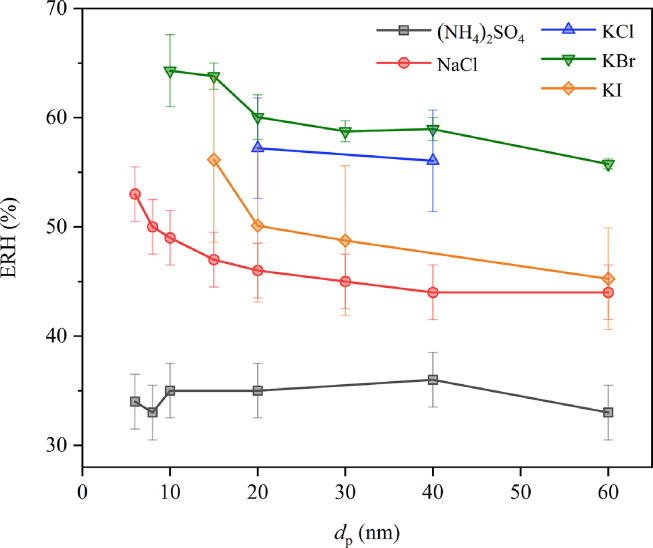
**The ERH values of (NH_4_)_2_SO_4_****[87]**, **NaCl****[86]**, **KCl****[91]**, **KBr****[91]**, **and KI****[91]****as a function of particle size (<100 nm) at 298 K**.

It summarizes ERH values of (NH_4_)_2_SO_4_ [87], NaCl [86], KCl [91], KBr [91], and KI [91] as a function of particle sizes (<100 nm) (Fig. 5). Two different types of size dependence of ERH can be identified for the five compounds included in Fig. 5. While no significant size dependence was observed for (NH_4_)_2_SO_4_ and KCl, ERH values decreased with particle size for NaCl, KBr and KI. For example, ERH values of NaCl were found to decrease with particle size from 53 ± 2.5% at 6 nm to 44 ± 2.5% at 60 nm [86]. Furthermore, as shown in the supplementary materials, ERH values of (NH_4_)_2_SO_4_, NaCl and KCl were determined to be 30–48%, 41–51% and 50–60% for 0.1–100 μm particles, agreeing well with those measured for <100 nm particles.

To summarize, although it is theoretically suggested that ERH values would increase with particle size, different size dependences of ERH values were observed for different compounds. To better understand the size dependence of ERH values, further experimental measurements are encouraged, and one critical aspect of such measurements should be to reduce the uncertainties of measured ERH values.

#### 3.2.3 Substrates used to support samples

The contact of supporting substrates with aqueous particles can significantly influence their nucleation mechanisms [12]. For airborne or levitated aqueous particles, critical germ formation occurs via homogeneous nucleation. For droplets deposited on substrates, the critical cluster formation frequency increases at the substrate interface, thus the formed crystal germ at the interface can induce heterogeneous nucleation of droplets, leading to efflorescence at higher RHs.

Zhang and co-authors investigated efflorescence transitions of Na_2_SO_4_ [135], NaNO_3_ [136], and sodium succinate [103,134] droplets deposited on ZnSe, and ERH values were measured to be 68–82% for Na_2_SO_4_, 62.5 ± 2.5% for NaNO_3_ and 50–59% for sodium succinate at 294–298 K. For comparison, ERH values were reported to be 55–59% for Na_2_SO_4_ [78,79,137,138], 35–45% for NaNO_3_ [139,140] and 46.7–47.9% for sodium succinate [101] at 298 K using airborne or levitated particles, significantly lower than those determined by Zhang and co-workers. It is suggested that defects or impurities in the ZnSe substrate can act as nucleation centers to enhance heterogeneous nucleation in droplets, thereby resulting in the occurrence of efflorescence at higher RHs [136].

However, not all the substrates on which aqueous droplets are deposited will promote efflorescence transitions. For example, Eom et al. [127] suggested that the observed ERH values of (NH_4_)_2_SO_4_, NaCl and KCl droplets deposited on transmission electron microscopy grids, parafilm-M, Al foil, silicon wafer and cover glass agreed well with those for airborne or levitated particles. In another study [141], the ERH was determined to be ∼45% at 298 K for NaNO_3_ particles deposited on CaF_2_ crystals, in good agreement with those (35–45%) reported by other studies [139,140,142].

In addition, it has been widely recognized that inclusion of [143], [144], [145], [146], [147] and collision with [147], [148], [149] insoluble minerals and soot would lead to the occurrence of efflorescence at higher RHs.

## 4 Summary and outlook

Deliquescence and efflorescence are two phase transition processes of critical importance in atmospheric science and many other fields. A large number of experimental studies have been carried out to measure deliquescence relative humidity (DRH) and efflorescence relative humidity (ERH) of compounds with atmospheric relevance. However, DRH and ERH values have not yet been summarized in a comprehensive manner. In this work, we have developed a comprehensive database that summarizes the measured DRH and ERH values of 110 compounds (68 inorganics and 42 organics), and provided the preferred DRH and ERH values at 298 K for these compounds (Table 1). In addition, we have also discussed the effects of a few key factors (e.g., temperature and particle size) on the measured DRH and ERH values.

A comprehensive summary of DRH and ERH values has been provided in the supplementary materials. We also plan to upload our summary sheets to a website that is funded by the National Natural Science Foundation of China (this website is under construction and will become online in 2022). This website will have an interactive and user-friendly interface in order to increase data accessibility, and it will also enable dynamic updates to include additional data in time. Currently this database only includes 42 organic compounds (i.e., five methanesulfonates, 12 monocarboxylic salts, 13 dicarboxylic acids, and 12 dicarboxylic salts), while there are many thousands of organic compounds contained by atmospheric aerosols. One major task in the future is to expand this database to include additional organic compounds with atmospheric and pharmaceutical relevance.

For the 110 compounds included in the database, 99 compounds have preferred DRH values and 40 compounds have ERH values (Table 1) at 298 K. One primary reason that the DRH and ERH values are not provided for many compounds is that large discrepancies found for reported DRH and ERH values precluded us from providing the preferred values. Therefore, careful and robust measurements are needed to reduce the uncertainties in the DRH and especially the ERH values of these compounds.

For many compounds included in the database, DRH and ERH measurements have only been provided at around room temperature (∼298 K), and the temperature dependence of DRH and ERH was only examined for a limited number of compounds. The temperature in the troposphere can range from <200 to >300 K; as a result, DRH and ERH measurements at different temperatures (especially <273 K) will be very useful to better understand phase transitions in the troposphere as well as under other cold environments (such as Mars) and to elucidate temperature dependence of DRH and ERH at the fundamental level in physical chemistry.

DRH and ERH are two RH thresholds at which abrupt changes in liquid water contents take place due to an increase or decrease in RH. In addition to DRH and ERH values, it is very important to know changes in the liquid water contents (i.e., hygroscopic growth factors or water-to-solute ratios) with RH. While the current database can serve as a starting point to summarize hygroscopic growth factors or water-to-solute ratios, we have not included these experimental data at this moment as it requires much more work, and one major task in the future would be to include these data in this database.

## Declaration of Competing Interest

The authors declare that they have no conflicts of interest in this work.

## Appendix Supplementary materials

Image, application 1
