# Supplementary material for: A database for deliquescence and efflorescence relative humidities of compounds with atmospheric relevance
Source: Fundam Res. 2021 Dec 2;2(4):578–87. doi: 10.1016/j.fmre.2021.11.021 (PMC11197750; doi:10.1016/j.fmre.2021.11.021)
Supplement: Supplementary file 1 [file mmc1.docx]

*Supplementary material of*

A database for deliquescence and efflorescence relative humidities of compounds with atmospheric relevance

Chao Peng,^1,2^ Lanxiadi Chen,^1,2,3^ Mingjin Tang^1,2,3,*^

^1^ State Key Laboratory of Organic Geochemistry, Guangdong Key Laboratory of Environmental Protection and Resources Utilization, and Guangdong-Hong Kong-Macao Joint Laboratory for Environmental Pollution and Control, Guangzhou Institute of Geochemistry, Chinese Academy of Sciences, Guangzhou 510640, China

^2^ CAS Center for Excellence in Deep Earth Science, Guangzhou 510640, China

^3^ University of Chinese Academy of Sciences, Beijing 100049, China

* Correspondence: Mingjin Tang ([mingjintang@gig.ac.cn)](mailto:mingjintang@gig.ac.cn))

**Table of Contents**

[1 Sulfates 1](#_Toc83562986)

[1.1 (NH_4_)_2_SO_4_ (ammonium sulfate) 1](#_Toc83562987)

[1.2 Li_2_SO_4_ (lithium sulfate) 14](#_Toc83562988)

[1.3 Na_2_SO_4_ (sodium sulfate) 17](#_Toc83562989)

[1.4 K_2_SO_4_ (potassium sulfate) 21](#_Toc83562990)

[1.5 MgSO_4_ (magnesium sulfate) and MgSO_4_∙7H_2_O (magnesium sulfate heptahydrate) 25](#_Toc83562991)

[2 Bisulfates 28](#_Toc83562992)

[2.1 NH_4_HSO_4_ (ammonium bisulfate) 28](#_Toc83562993)

[2.2 NaHSO_4_ (sodium bisulfate) 31](#_Toc83562994)

[2.3 KHSO_4_ (potassium bisulfate) 33](#_Toc83562995)

[3 Nitrates 35](#_Toc83562996)

[3.1 NH_4_NO_3_ (ammonium nitrate) 35](#_Toc83562997)

[3.2 LiNO_3_ (lithium nitrate) and LiNO_3_∙3H_2_O (lithium nitrate trihydrate) 41](#_Toc83562998)

[3.3 NaNO_3_ (sodium nitrate) 43](#_Toc83562999)

[3.4 KNO_3_ (potassium nitrate) 51](#_Toc83563000)

[3.5 Ca(NO_3_)_2_ (calcium nitrate) 55](#_Toc83563001)

[3.6 Ca(NO_3_)_2_∙4H_2_O (calcium nitrate tetrahydrate) 59](#_Toc83563002)

[3.7 Mg(NO_3_)_2_ (magnesium nitrate) 62](#_Toc83563003)

[3.8 Mg(NO_3_)_2_∙6H_2_O (magnesium nitrate hexahydrate) 66](#_Toc83563004)

[4 Fluorides 69](#_Toc83563005)

[4.1 NaF (sodium fluoride) 69](#_Toc83563006)

[4.2 KF (potassium fluoride) and KF∙2H_2_O (potassium fluoride dihydrate) 71](#_Toc83563007)

[5 Chlorides 74](#_Toc83563008)

[5.1 NH_4_Cl (ammonium chloride) 74](#_Toc83563009)

[5.2 LiCl (lithium chloride) and LiCl∙H_2_O (lithium chloride monohydrate) 77](#_Toc83563010)

[5.3 NaCl (sodium chloride) 82](#_Toc83563011)

[5.4 KCl (potassium chloride) 93](#_Toc83563012)

[5.5 CaCl_2_ (calcium chloride) 100](#_Toc83563013)

[5.6 CaCl_2_∙2H_2_O, CaCl_2_∙4H_2_O and CaCl_2_∙6H_2_O (calcium chloride didehydrate, calcium chloride tetradehydrate and calcium chloride hexadehydrate) 102](#_Toc83563014)

[5.7 MgCl_2_ (magnesium chloride) 104](#_Toc83563015)

[5.8 MgCl_2_∙4H_2_O and MgCl_2_∙6H_2_O (magnesium chloride tetrahydrate and magnesium chloride hexahydrate) 108](#_Toc83563016)

[6 Bromides 111](#_Toc83563017)

[6.1 NH_4_Br (ammonium bromide) 111](#_Toc83563018)

[6.2 LiBr (lithium bromide) and LiBr∙2H_2_O (lithium bromide dihydrate) 113](#_Toc83563019)

[6.3 NaBr (sodium bromide) 115](#_Toc83563020)

[6.4 NaBr∙2H_2_O (sodium bromide dihydrate) 122](#_Toc83563021)

[6.5 KBr (potassium bromide) 124](#_Toc83563022)

[6.6 CaBr_2_ (calcium bromide) 128](#_Toc83563023)

[6.7 MgBr_2_ (magnesium bromide) 131](#_Toc83563024)

[7 Iodides 133](#_Toc83563025)

[7.1 NH_4_I (ammonium iodide) 133](#_Toc83563026)

[7.2 LiI (lithium iodide) and LiI∙3H_2_O (lithium iodide trihydrate) 135](#_Toc83563027)

[7.3 NaI (sodium iodide) and NaI∙2H_2_O (sodium iodide dihydrate) 137](#_Toc83563028)

[7.4 KI (potassium iodide) 140](#_Toc83563029)

[8 Chlorates 144](#_Toc83563030)

[8.1 NaClO_3_ (sodium chlorate) 144](#_Toc83563031)

[8.2 KClO_3_ (potassium chlorate) 146](#_Toc83563032)

[8.3 Ca(ClO_3_)_2_∙2H_2_O (calcium chlorate dihydrate) and Mg(ClO_3_)_2_∙6H_2_O (magnesium chlorate hexahydrate) 148](#_Toc83563033)

[9 Iodates 150](#_Toc83563034)

[9.1 I_2_O_5_ (iodic anhydride) 150](#_Toc83563035)

[9.2 HIO_3_ (iodic acid) 152](#_Toc83563036)

[9.3 KIO_3_ (potassium iodate) 154](#_Toc83563037)

[10 Perchlorates 156](#_Toc83563038)

[10.1 NH_4_ClO_4_ (ammonium perchlorate) 156](#_Toc83563039)

[10.2 LiClO_4_ (lithium perchlorate) and LiClO_4_∙3H_2_O (lithium perchlorate trihydrate) 158](#_Toc83563040)

[10.3 NaClO_4_ (sodium perchlorate) 160](#_Toc83563041)

[10.4 NaClO_4_∙H_2_O (sodium perchlorate monohydrate) 163](#_Toc83563042)

[10.5 KClO_4_ (potassium perchlorate) 165](#_Toc83563043)

[10.6 Ca(ClO_4_)_2_ (calcium perchlorate) 167](#_Toc83563044)

[10.7 Ca(ClO_4_)_2_∙4H_2_O (calcium perchlorate tetrahydrate) 169](#_Toc83563045)

[10.8 Mg(ClO_4_)_2_ (magnesium perchlorate) 171](#_Toc83563046)

[10.9 Mg(ClO_4_)_2_∙6H_2_O (magnesium perchlorate hexahydrate) 173](#_Toc83563047)

[11 Carbonates 175](#_Toc83563048)

[11.1 Li_2_CO_3_ (lithium carbonate) 175](#_Toc83563049)

[11.2 Na_2_CO_3_ (sodium carbonate) 177](#_Toc83563050)

[11.3 K_2_CO_3_ (potassium carbonate) and K_2_CO_3_∙2H_2_O (potassium carbonate dihydrate) 180](#_Toc83563051)

[12 Methanesulfonates 183](#_Toc83563052)

[12.1 CH_3_SO_3_NH_4_ (ammonium methanesulfonate) 183](#_Toc83563053)

[12.2 CH_3_SO_3_Na (sodium methanesulfonate) 185](#_Toc83563054)

[12.3 CH_3_SO_3_K (potassium methanesulfonate) 187](#_Toc83563055)

[12.4 Ca(CH_3_SO_3_)_2_ (calcium methanesulfonate) 189](#_Toc83563056)

[12.5 Mg(CH_3_SO_3_)_2_ (magnesium methanesulfonate) 191](#_Toc83563057)

[13 Monocarboxylic salts 193](#_Toc83563058)

[13.1 HCOONa (sodium formate) 193](#_Toc83563059)

[13.2 Ca(HCOO)_2_ (calcium formate) and Mg(HCOO)_2_∙2H_2_O (magnesium formate dihydrate) 195](#_Toc83563060)

[13.3 CH_3_COONH_4_ (ammonium acetate) 197](#_Toc83563061)

[13.4 CH_3_COOLi (lithium acetate) 199](#_Toc83563062)

[13.5 CH_3_COONa (sodium acetate) 201](#_Toc83563063)

[13.6 CH_3_COOK (potassium acetate) 204](#_Toc83563064)

[13.7 Ca(CH_3_COO)_2_ (calcium acetate) and Ca(CH_3_COO)_2_∙H_2_O (calcium acetate monohydrate) 207](#_Toc83563065)

[13.8 Mg(CH_3_COO)_2_ (magnesium acetate) and Mg(CH_3_COO)_2_∙4H_2_O (magnesium acetate tetrahydrate) 209](#_Toc83563066)

[13.9 NaC_3_H_3_O_3_ (sodium pyruvate) 211](#_Toc83563067)

[14 Dicarboxylic acids 213](#_Toc83563068)

[14.1 H_2_C_2_O_4_ (oxalic acid) 213](#_Toc83563069)

[14.2 CH_2_(COOH)_2_ (malonic acid) 217](#_Toc83563070)

[14.3 (CH_2_)_2_(COOH)_2_ (succinic acid) 223](#_Toc83563071)

[14.4 (CH_2_)_3_(COOH)_2_ (glutaric acid) 226](#_Toc83563072)

[14.5 (CH_2_)_4_(COOH)_2_ (adipic acid) 232](#_Toc83563073)

[14.6 (CH_2_)_5_(COOH)_2_ (pimelic acid) 235](#_Toc83563074)

[14.7 (CH_2_)_6_(COOH)_2_ (suberic acid) 237](#_Toc83563075)

[14.8 (CH_2_)_7_(COOH)_2_ (azelaic acid) 239](#_Toc83563076)

[14.9 C_2_H_2_(COOH)_2_ (maleic acid) 241](#_Toc83563077)

[14.10 (CH_2_CHOH)(COOH)_2_ (malic acid) 244](#_Toc83563078)

[14.11 (CHOH)_2_(COOH)_2_ (tartaric acid) 247](#_Toc83563079)

[14.12 C_6_H_4_(COOH)_2_ (phthalic acid) 249](#_Toc83563080)

[15 Dicarboxylic salts 251](#_Toc83563081)

[15.1 (NH_4_)_2_C_2_O_4_ (ammonium oxalate) 251](#_Toc83563082)

[15.2 (NH_4_)_2_C_2_O_4_·H_2_O (ammonium oxalate monohydrate) 254](#_Toc83563083)

[15.3 Na_2_C_2_O_4_ (sodium oxalate) 256](#_Toc83563084)

[15.4 K_2_C_2_O_4_ (potassium oxalate) 259](#_Toc83563085)

[15.5 CaC_2_O_4_ (calcium oxalate) and CaC_2_O_4_·H_2_O (calcium oxalate monohydrate) 261](#_Toc83563086)

[15.6 Na_2_C_3_H_2_O_4_ (sodium malonate) and Na_2_C_3_H_2_O_4_·H_2_O (sodium malonate monohydrate) 263](#_Toc83563087)

[15.7 Na_2_C_4_H_4_O_4_ (sodium succinate) 266](#_Toc83563088)

[15.8 Na_2_C_4_H_2_O_4_ (sodium maleate) 269](#_Toc83563089)

[15.9 (NH_4_)_2_C_4_H_4_O_6_ (ammonium tartrate) 271](#_Toc83563090)

[15.10 Na_2_C_4_H_4_O_6_ (sodium tartrate) 273](#_Toc83563091)

# Sulfates

## (NH_4_)_2_SO_4_ (ammonium sulfate)

| Reference | *T* (K) | *D* | DRH (%) | ERH (%) | Techniques/Comments |
| --- | --- | --- | --- | --- | --- |
| Adams and Merz, 1929 | 283 | - | 79.8 | - | Isopiestic method |
|  | 288 |  | 79.3 |  |  |
|  | 293 |  | 81.0 |  |  |
|  | 298 |  | 81.8 |  |  |
|  | 303 |  | 79.2 |  |  |
|  | 313 |  | 78.2 |  |  |
|  | 323 |  | 77.8 |  |  |
| Rockland, 1960 | 278 | - | 81 | - | Nonisopiestic method |
|  | 283 |  | 80 |  |  |
|  | 288 |  | 79 |  |  |
|  | 293 |  | 79 |  |  |
|  | 298 |  | 79 |  |  |
|  | 303 |  | 79 |  |  |
|  | 308 |  | 79 |  |  |
|  | 313 |  | 79 |  |  |
| Greenspan, 1977 | 273 | - | 82.3±0.9 | - | Nonisopiestic method |
|  | 278 |  | 82.4±0.7 |  |  |
|  | 283 |  | 82.1±0.5 |  |  |
|  | 288 |  | 81.7±0.4 |  |  |
|  | 293 |  | 81.3±0.3 |  |  |
|  | 298 |  | 81.0±0.3 |  |  |
|  | 303 |  | 80.6±0.3 |  |  |
|  | 308 |  | 80.3±0.4 |  |  |
|  | 313 |  | 79.9±0.5 |  |  |
|  | 318 |  | 79.6±0.7 |  |  |
|  | 323 |  | 79.2±0.9 |  |  |
| Charlson et al., 1978 | 298 | - | 81 | - | Nonisopiestic method |
| Richardson and Spann, 1984 | 297 | ~4 μm | 80.0±1.2 | 34.7±0.4 | EDB |
| Cohen et al., 1987 | 293 | 20 μm | 80.6-81.3 | 48 | EDB |
| Chan et al., 1992 | 298 | 30 μm | - | 47.5 | EDB |
| Tang and Munkelwitz, 1993 | 298 | 6-8 μm | 79.9±0.5 | - | EDB |
| Tang and Munkelwitz, 1994 | 298 | 6-8 μm | 80 | 37-40 | EDB |
| Tang et al., 1995 | 298 | 14-16 μm | 80 | 37 | EDB |
| Cziczo et al., 1997 | 298 | 0.45 μm | 79±1 | 33±2 | FTIR |
| Dougle et al., 1998 | 298 | ~0.5 μm | 80 | 42 | Nephelometer |
| Lee and Hsu, 1998 | 296 | ~0.8 μm | 80±1 | 35±1 | Katharometer |
| Xu et al., 1998 | 254 | 5-10 μm | 83 | 54 | EDB |
|  | 255 |  | 83 | 52 |  |
|  | 258 |  | 82 | 50 |  |
|  | 260 |  | 82 | 48 |  |
|  | 263 |  | 82 | 46 |  |
|  | 265 |  | 82 | 45 |  |
|  | 268 |  | 82 | 44 |  |
|  | 273 |  | 81 | 41 |  |
|  | 278 |  | 81 | 38 |  |
|  | 283 |  | 81 | 37 |  |
|  | 288 |  | 81 | 37 |  |
|  | 293 |  | 80 | 37 |  |
|  | 298 |  | 80 | 37 |  |
|  | 303 |  | 80 | 37 |  |
|  | 308 |  | 79 | 36 |  |
| Cziczo and Abbatt, 1999 | 238 | ~0.35 μm | - | 41±6 | FTIR |
|  | 254 |  | 83±4 | 42±4 (253 K) |  |
|  | 263 |  | 82±3 | - |  |
|  | 273 |  | 82±2 | 37±3 |  |
|  | 283 |  | 81±2 | - |  |
| Han and Martin, 1999 | 298 | ~2 μm | 79±2 | 35±2 | FTIR |
| Onasch et al., 1999 | 234 | 0.65-0.75 μm | - | 39.0±6 | FTIR |
|  | 236 |  | - | 36.0±6 |  |
|  | 244 |  | - | 36.5±6 |  |
|  | 249 |  | - | 36.4±6 |  |
|  | 253 |  | - | 35.5±6 |  |
|  | 258 |  | 82.2±2 | - |  |
|  | 263 |  | 82.5±2 | 33.1±3 |  |
|  | 268 |  | 81.4±2 | - |  |
|  | 273 |  | 81.3±2 | 30.5±2.5 |  |
|  | 283 |  | 80.3±2 | 31.0±2.5 |  |
|  | 295 |  | 80.0±2 | 32.0±1 |  |
| Cruz and Pandis, 2000 | 297 | 50-100 nm | 79±1 | - | HTDMA |
| Hämeri et al., 2000 | 298 | 8-50 nm | 79-81 | - | HTDMA |
| Onasch et al., 2000 | 298 | 10 μm | - | 37±1.4 | EDB |
| Joutsensaari et al., 2001 | 296 | 100 nm | 79 | - | HTDMA |
| Prenni et al., 2001 | 303 | 100 nm | 77.5±1.5 | - | HTDMA |
| Brooks et al., 2002 | 263 | - | 81.2±3 | - | Nonisopiestic method |
|  | 277 |  | 81.9±3 |  |  |
|  | 297 |  | 81.7±3 |  |  |
| Ebert et al., 2002 | 278 | 0.1-20 μm | 81.3±1.5 | - | ESEM |
| Gysel et al., 2002 | 263 | 100 nm | 82.5±1.5 | - | HTDMA |
|  | 293 |  | 80.0±1.2 | - |  |
| Wise et al., 2003 | 298 | - | 80.0 | - | Nonisopiestic method |
| Colberg et al., 2004 | 260 | 2-20 μm | 81.5 | 28.5±2.5 | EDB |
|  | 263.5 |  | 81 | 30.8±2.5 |  |
| Parsons et al., 2004 | 262 | 2-40 μm | 82.5±2.1 | - | Optical microscopy |
|  | 273 |  | 81.1±2.1 |  |  |
|  | 273.5 |  | 82.0±2.1 |  |  |
|  | 282 |  | 81.5±2.1 |  |  |
|  | 283 |  | 81.0±2.1 |  |  |
|  | 291 |  | 80.5±2.1 |  |  |
|  | 292.5 |  | 80.2±2.1 |  |  |
| Schlenker et al., 2004 | 293 | 300 nm | 80.0 | 35.0 | FTIR |
| Biskos et al., 2006 | 298 | 6 nm | 82±2.5 | 34±2.5 | HTDMA |
|  |  | 8 nm | 81±2.5 | 33±2.5 |  |
|  |  | 10 nm | 80±2.5 | 35±2.5 |  |
|  |  | 20 nm | 82±2.5 | 35±2.5 |  |
|  |  | 40 nm | 80±2.5 | 36±2.5 |  |
|  |  | 60 nm | 80±2.5 | 33±2.5 |  |
| Gao et al., 2006 | 298 | 43, 47 nm | - | 31.0±6.4 | TDMA |
| Pant et al., 2006 | 293 | 5-30 μm | - | 35-38 | Optical microscopy |
| Parsons et al., 2006 | 298 | 10-30 μm | - | 34-39 | EDB |
| Schuttlefield et al., 2007 | 298 | - | 81±2 | - | ATR-FTIR |
| Treuel et al., 2008 | 283 | ~50 μm | 81 | - | EDB |
|  | 288 |  | 81 |  |  |
|  | 297 |  | 80 |  |  |
| Zardini et al., 2008 | 291 | 5-25 μm | 80 | 35-40 | EDB |
|  | 292 | 100 nm | 80 | 35-40 | HTDMA |
| Mikhailov et al., 2009 | 298 | 100 nm | 80 | 30 | HTDMA |
| Ahn et al., 2010 | 298 | 2.5-10 μm | 79.9±0.3 | 37.9±0.5 | Optical microscopy |
| Ciobanu et al., 2010 | 293 | 16-31 μm | - | 36.3-43.7 | Optical microscopy |
| Yeung and Chan, 2010 | 297 | 10-30 μm | 79.5 | 39.5 | Micro-Raman |
| Arenas et al., 2012 | 298 | - | 82.6±2.2 | - | QCM |
| Beyer et al., 2014 | 277 | - | 82.9±1 | - | VSA |
|  | 298 | - | 81.9±1 |  |  |
| Laskina et al., 2015 | 298 K | 100 nm | 80.4±0.6 | 37.7±4.1 | HTDMA |
|  |  | 3-12 μm | 82.3±2.5 | 43.5±2.1 | Micro-Raman |
| Gu et al., 2017 | 278 | - | 82.4±0.7 | - | VSA |
|  | 283 |  | 82.1±0.5 |  |  |
|  | 288 |  | 81.7±0.4 |  |  |
|  | 293 |  | 81.3±0.3 |  |  |
|  | 298 |  | 81.0±0.3 |  |  |
|  | 303 |  | 80.6±0.3 |  |  |
| Lei et al., 2018 | 298 | 100 nm | 80 | 35 | HTDMA |
| Zielinski et al., 2018 | 292-295 | 300 nm | 79-81 | 35-43 | MEMS |
| Ma et al., 2019 | 298 | ~3 μm | ~80 | 36-46 | Vacuum-FTIR |
| Lei et al., 2020 | 292 | 6-100 | 80-83 | 30-34 | Nano-HTDMA |

**Comments:**

The DRH of (NH_4_)_2_SO_4_ was measured by a large number of studies (Adams and Merz, 1929; Rockland, 1960; Greenspan, 1977; Charlson et al., 1978; Richardson and Spann, 1984; Cohen et al., 1987; Chan et al., 1992; Tang and Munkelwitz, 1993; Tang and Munkelwitz, 1994; Tang et al., 1995; Cziczo et al., 1997; Dougle et al., 1998; Lee and Hsu, 1998; Xu et al., 1998; Cziczo and Abbatt, 1999; Han and Martin, 1999; Onasch et al., 1999; Cruz and Pandis, 2000; Hämeri et al., 2000; Onasch et al., 2000; Joutsensaari et al., 2001; Prenni et al., 2001; Brooks et al., 2002; Ebert et al., 2002; Gysel et al., 2002; Wise et al., 2003; Colberg et al., 2004; Parsons et al., 2004; Schlenker et al., 2004; Biskos et al., 2006; Gao et al., 2006; Pant et al., 2006; Parsons et al., 2006; Schuttlefield et al., 2007; Treuel et al., 2008; Zardini et al., 2008; Mikhailov et al., 2009; Ahn et al., 2010; Ciobanu et al., 2010; Yeung and Chan, 2010; Arenas et al., 2012; Beyer et al., 2014; Laskina et al., 2015; Gu et al., 2017; Lei et al., 2018; Zielinski et al., 2018; Ma et al., 2019; Lei et al., 2020). It was measured to be 78-82% at 298 K, showing good agreement among diferent studies.


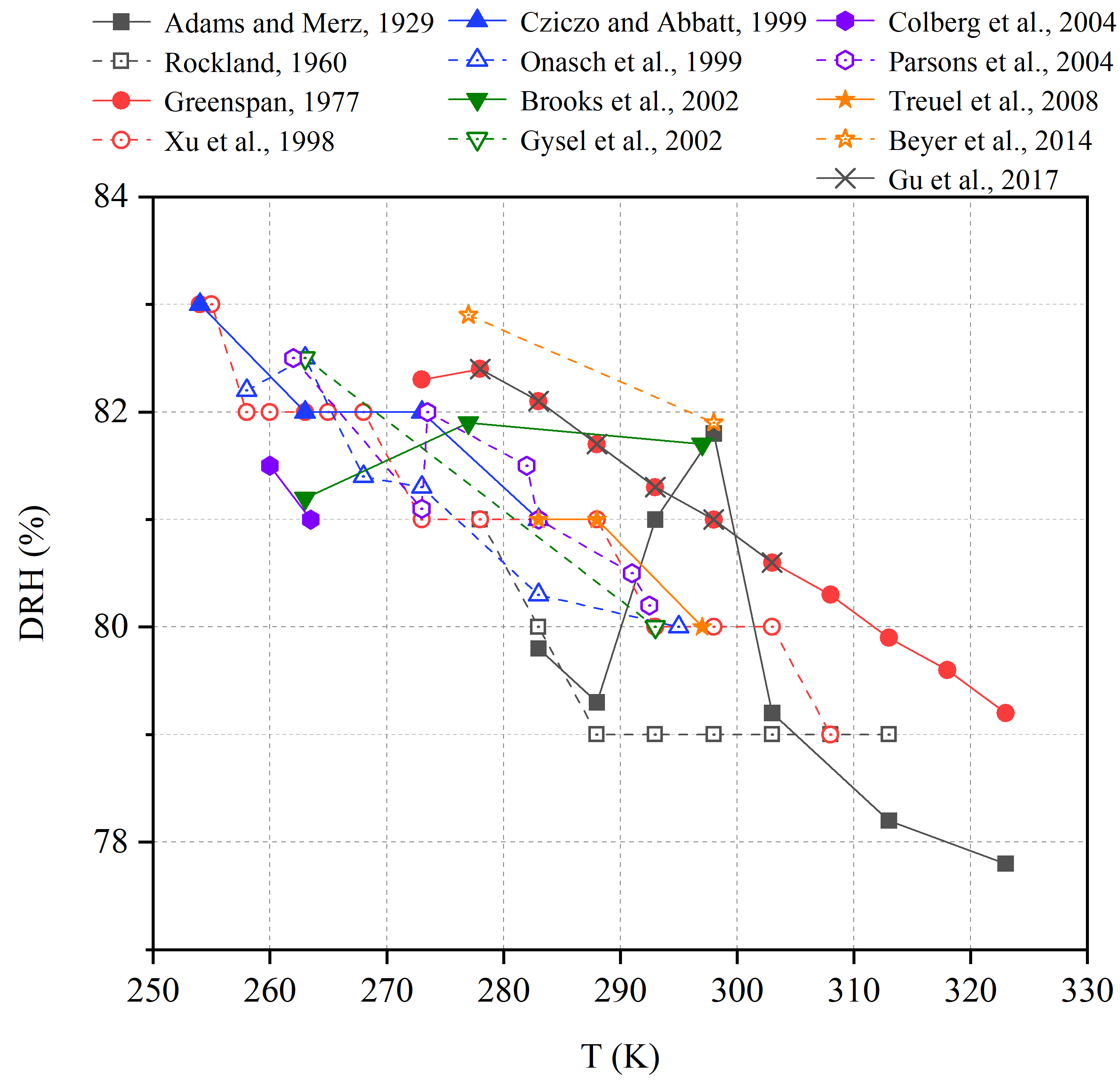


Several studies (Adams and Merz, 1929; Rockland, 1960; Greenspan, 1977; Xu et al., 1998; Cziczo and Abbatt, 1999; Onasch et al., 1999; Gysel et al., 2002; Colberg et al., 2004; Parsons et al., 2004; Treuel et al., 2008; Beyer et al., 2014; Gu et al., 2017) measured DRH of (NH_4_)_2_SO_4_ at different temperature, and revealed a slightly negative dependence on temperature.

Adams and Merz (1929) found that the DRH of (NH_4_)_2_SO_4_ decreased slightly with temperature from 79.8% at 283 to 77.8% at 323 K, Rockland (1960) found that it decreased with temperature from 81% at 278 K to 79% at 313 K, and Greenspan (1977) found that it decreased with temperature from 82.3±0.9% at 273 K to 79.2±0.9% at 323 K; Xu et al. (1998) found that it decreased with temperature from 83% at 254 K to 79% at 308 K, Cziczo and Abbatt (1999) found that it decreased with temperature from 83±4% at 254 K to 81±2% at 283 K, and Onasch et al. (1999) found that it decreased with temperature from 82.2±2% at 258 K to 80.0±2% at 295 K; Gysel et al. (2002) found that it decreased with temperature from 82.5±1.5% at 263 K to 80.0±1.2% at 293 K, Colberg et al. (2004) found that it decreased with temperature from 81.5% at 260 K to 81% at 263.5 K, and Parsons et al. (2004) found that it decreased with temperature from 82.5±2.1% at 262 K to 80.2±2.1% at 292.5 K; Treuel et al. (2008) found that it decreased with temperature from 81% at 283 K to 80% at 297 K, Beyer et al. (2014) found that it decreased with temperature from 82.9±1% at 277 K to 81.9±1% at 298 K, and Gu et al. (2017) suggested that it decreased with temperature from 82.4±0.7% at 278 K to 80.6±0.3% at 303 K. In addition, DRH of (NH_4_)_2_SO_4_ was measured to be 81.2±3% at 263 K, 81.9±3% at 277 K and 81.7±3% at 297 K (Brooks et al., 2002), showing no significant dependence on temperature.

The ERH values of (NH_4_)_2_SO_4_ were measured by a number of studies (Richardson and Spann, 1984; Cohen et al., 1987; Chan et al., 1992; Tang and Munkelwitz, 1994; Tang et al., 1995; Cziczo et al., 1997; Dougle et al., 1998; Lee and Hsu, 1998; Xu et al., 1998; Cziczo and Abbatt, 1999; Han and Martin, 1999; Onasch et al., 1999; Onasch et al., 2000; Colberg et al., 2004; Schlenker et al., 2004; Biskos et al., 2006; Gao et al., 2006; Pant et al., 2006; Parsons et al., 2006; Zardini et al., 2008; Mikhailov et al., 2009; Ahn et al., 2010; Ciobanu et al., 2010; Yeung and Chan, 2010; Laskina et al., 2015; Lei et al., 2018; Zielinski et al., 2018; Ma et al., 2019; Lei et al., 2020). It was determined to be 30-48% at 298 K, showed relatively good agreement among different studies.

Temperature dependence of ERH of (NH_4_)_2_SO_4_ was investigated by four studies (Xu et al., 1998; Cziczo and Abbatt, 1999; Onasch et al., 1999; Colberg et al., 2004). Xu et al. (1998) found that it first decreased with temperature from 54% at 254 K to 37% at 283 K, and then showed no significant change when temperature was further increased to 308 K. Cziczo and Abbatt (1999) found that it decreased with temperature from 41±6% at 238 K to 37±3% at 273 K. Onasch et al. (1999) suggested that it first decreased with temperature from 39.0±6% at 234 K to 30.5±2.5% at 273 K, and then slightly increased with temperature to 32.0±1% at 295 K. Colberg et al. (2004) suggested that it slightly increased with temperature from 28.5±2.5% at 260 K to 30.8±2.5% at 263.5 K.


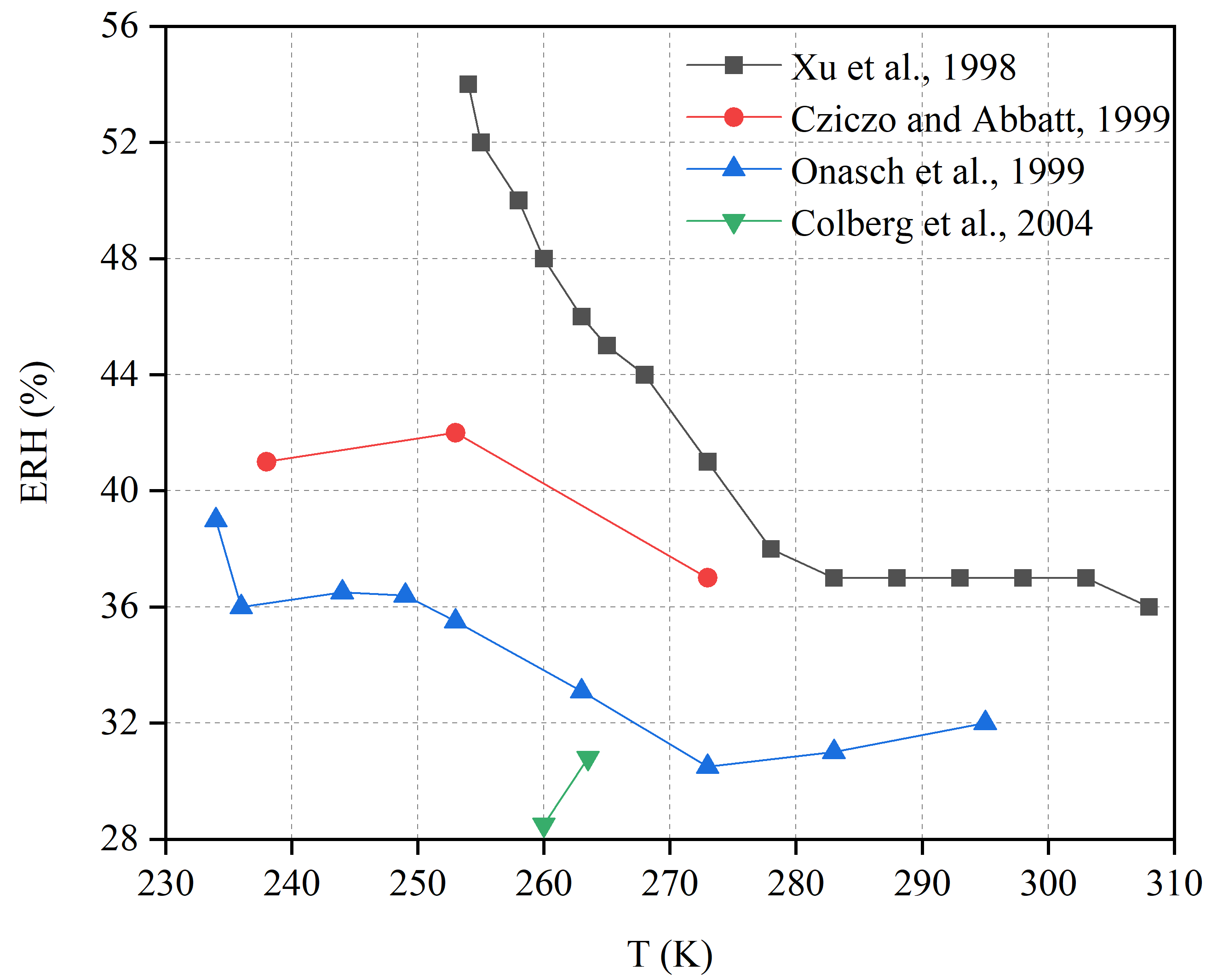


**Preferred values at 298 K for (NH_4_)_2_SO_4_:**

DRH: 78-82%

ERH: 30-48%

**References:**

Adams, J. R., and Merz, A. R.: Hygroscopicity of fertilizer materials and mixtures, Industrial and Engineering Chemistry, 21, 305-307, 1929.

Ahn, K.-H., Kim, S.-M., Jung, H.-J., Lee, M.-J., Eom, H.-J., Maskey, S., and Ro, C.-U.: Combined Use of Optical and Electron Microscopic Techniques for the Measurement of Hygroscopic Property, Chemical Composition, and Morphology of Individual Aerosol Particles, Analytical Chemistry, 82, 7999-8009, 2010.

Arenas, K. J. L., Schill, S. R., Malla, A., and Hudson, P. K.: Deliquescence Phase Transition Measurements by Quartz Crystal Microbalance Frequency Shifts, Journal of Physical Chemistry A, 116, 7658-7667, 2012.

Beyer, K. D., Schroeder, J. R., and Kissinger, J. A.: Temperature-Dependent Deliquescence Relative Humidities and Water Activities Using Humidity Controlled Thermogravimetric Analysis with Application to Malonic Acid, Journal of Physical Chemistry A, 118, 2488-2497, 2014.

Biskos, G., Paulsen, D., Russell, L. M., Buseck, P. R., and Martin, S. T.: Prompt deliquescence and efflorescence of aerosol nanoparticles, Atmos. Chem. Phys., 6, 4633-4642, 2006.

Brooks, S. D., Wise, M. E., Cushing, M., and Tolbert, M. A.: Deliquescence behavior of organic/ammonium sulfate aerosol, Geophysical Research Letters, 29, 2002.

Chan, C. K., Flagan, R. C., and Seinfeld, J. H.: Water activities of NH_4_NO_3_/(NH_4_)_2_SO_4_ solutions, Atmospheric Environment. Part A. General Topics, 26, 1661-1673, 1992.

Charlson, R. J., Covert, D. S., Larson, T. V., and Waggoner, A. P.: Chemical properties of tropospheric sulfur aerosols, Atmospheric Environment (1967), 12, 39-53, 1978.

Ciobanu, V. G., Marcolli, C., Krieger, U. K., Zuend, A., and Peter, T.: Efflorescence of Ammonium Sulfate and Coated Ammonium Sulfate Particles: Evidence for Surface Nucleation, Journal of Physical Chemistry A, 114, 9486-9495, 2010.

Cohen, M. D., Flagan, R. C., and Seinfeld, J. H.: Studies of concentrated electrolyte-solutions using the electrodynamic balance .1. Water activities for single-electrolyte solutions, Journal of Physical Chemistry, 91, 4563-4574, 1987.

Cohen, M. D., Flagan, R. C., and Seinfeld, J. H.: Studies of concentrated electrolyte solutions using the electrodynamic balance. 3. Solute nucleation, Journal of Physical Chemistry, 91, 4583-4590, 1987.

Colberg, C. A., Krieger, U. K., and Peter, T.: Morphological investigations of single levitated H_2_SO_4_/NH_3_/H_2_O aerosol particles during deliquescence/efflorescence experiments, Journal of Physical Chemistry A, 108, 2700-2709, 2004.

Cruz, C. N., and Pandis, S. N.: Deliquescence and hygroscopic growth of mixed inorganic-organic atmospheric aerosol, Environmental Science & Technology, 34, 4313-4319, 2000.

Cziczo, D. J., and Abbatt, J. P. D.: Deliquescence, efflorescence, and supercooling of ammonium sulfate aerosols at low temperature: Implications for cirrus cloud formation and aerosol phase in the atmosphere, Journal of Geophysical Research-Atmospheres, 104, 13781-13790, 1999.

Cziczo, D. J., Nowak, J. B., Hu, J. H., and Abbatt, J. P. D.: Infrared spectroscopy of model tropospheric aerosols as a function of relative humidity: Observation of deliquescence and crystallization, Journal of Geophysical Research-Atmospheres, 102, 18843-18850, 1997.

Dougle, P. G., Veefkind, J. P., and ten Brink, H. M.: Crystallisation of mixtures of ammonium nitrate, ammonium sulphate and soot, Journal of Aerosol Science, 29, 375-386, 1998.

Ebert, M., Inerle-Hof, M., and Weinbruch, S.: Environmental scanning electron microscopy as a new technique to determine the hygroscopic behaviour of individual aerosol particles, Atmospheric Environment, 36, 5909-5916, 2002.

Gao, Y., Chen, S. B., and Yu, L. E.: Efflorescence relative humidity for ammonium sulfate particles, Journal of Physical Chemistry A, 110, 7602-7608, 2006.

Greenspan, L.: Humidity fixed-points of binary saturated aqueous-solutions, Journal of Research of the National Bureau of Standards Section a-Physics and Chemistry, 81, 89-96, 1977.

Gu, W., Li, Y., Zhu, J., Jia, X., Lin, Q., Zhang, G., Ding, X., Song, W., Bi, X., Wang, X., and Tang, M.: Investigation of water adsorption and hygroscopicity of atmospherically relevant particles using a commercial vapor sorption analyzer, Atmospheric Measurement Techniques, 10, 3821-3832, 2017.

Gysel, M., Weingartner, E., and Baltensperger, U.: Hygroscopicity of aerosol particles at low temperatures. 2. Theoretical and experimental hygroscopic properties of laboratory generated aerosols, Environmental Science & Technology, 36, 63-68, 2002.

Hameri, K., Vakeva, M., Hansson, H. C., and Laaksonen, A.: Hygroscopic growth of ultrafine ammonium sulphate aerosol measured using an ultrafine tandem differential mobility analyzer, Journal of Geophysical Research-Atmospheres, 105, 22231-22242, 2000.

Han, J. H., and Martin, S. T.: Heterogeneous nucleation of the efflorescence of (NH_4_)_2_SO_4_ particles internally mixed with Al_2_O_3_, TiO_2_, and ZrO_2_, Journal of Geophysical Research-Atmospheres, 104, 3543-3553, 1999.

Joutsensaari, J., Vaattovaara, P., Vesterinen, M., Hameri, K., and Laaksonen, A.: A novel tandem differential mobility analyzer with organic vapor treatment of aerosol particles, Atmospheric Chemistry and Physics, 1, 51-60, 2001.

Laskina, O., Morris, H. S., Grandquist, J. R., Qin, Z., Stone, E. A., Tivanski, A. V., and Grassian, V. H.: Size Matters in the Water Uptake and Hygroscopic Growth of Atmospherically Relevant Multicomponent Aerosol Particles, The journal of physical chemistry. A, 119, 4489-4497, 2015.

Lee, C. T., and Hsu, W. C.: A novel method to measure aerosol water mass, Journal of Aerosol Science, 29, 827-837, 1998.

Lei, T., Ma, N., Hong, J., Tuch, T., Wang, X., Wang, Z., Pöhlker, M., Ge, M., Wang, W., Mikhailov, E., Hoffmann, T., Pöschl, U., Su, H., Wiedensohler, A., and Cheng, Y.: Nano-hygroscopicity tandem differential mobility analyzer (nano-HTDMA) for investigating hygroscopic properties of sub-10&thinsp;nm aerosol nanoparticles, Atmos. Meas. Tech., 13, 5551-5567, 2020.

Lei, T., Zuend, A., Cheng, Y., Su, H., Wang, W., and Ge, M.: Hygroscopicity of organic surrogate compounds from biomass burning and their effect on the efflorescence of ammonium sulfate in mixed aerosol particles, Atmospheric Chemistry and Physics, 18, 1045-1064, 2018.

Ma, S.-S., Yang, W., Zheng, C.-M., Pang, S.-F., and Zhang, Y.-H.: Subsecond measurements on aerosols: From hygroscopic growth factors to efflorescence kinetics, Atmospheric Environment, 210, 177-185, 2019.

Mikhailov, E., Vlasenko, S., Martin, S. T., Koop, T., and Poeschl, U.: Amorphous and crystalline aerosol particles interacting with water vapor: conceptual framework and experimental evidence for restructuring, phase transitions and kinetic limitations, Atmospheric Chemistry And Physics, 9, 9491-9522, 2009.

Onasch, T. B., McGraw, R., and Imre, D.: Temperature-dependent heterogeneous efflorescence of mixed ammonium sulfate/calcium carbonate particles, Journal of Physical Chemistry A, 104, 10797-10806, 2000.

Onasch, T. B., Siefert, R. L., Brooks, S. D., Prenni, A. J., Murray, B., Wilson, M. A., and Tolbert, M. A.: Infrared spectroscopic study of the deliquescence and efflorescence of ammonium sulfate aerosol as a function of temperature, Journal of Geophysical Research-Atmospheres, 104, 21317-21326, 1999.

Pant, A., Parsons, M. T., and Bertram, A. K.: Crystallization of aqueous ammonium sulfate particles internally mixed with soot and kaolinite: Crystallization relative humidities and nucleation rates, Journal of Physical Chemistry A, 110, 8701-8709, 2006.

Parsons, M. T., Mak, J., Lipetz, S. R., and Bertram, A. K.: Deliquescence of malonic, succinic, glutaric, and adipic acid particles, Journal of Geophysical Research-Atmospheres, 109, 2004.

Parsons, M. T., Riffell, J. L., and Bertram, A. K.: Crystallization of aqueous inorganic-malonic acid particles: Nucleation rates, dependence on size, and dependence on the ammonium-to-sulfate, Journal of Physical Chemistry A, 110, 8108-8115, 2006.

Prenni, A. J., DeMott, P. J., Kreidenweis, S. M., Sherman, D. E., Russell, L. M., and Ming, Y.: The effects of low molecular weight dicarboxylic acids on cloud formation, Journal of Physical Chemistry A, 105, 11240-11248, 2001.

Richardson, C. B., and Spann, J. F.: Measurement of the water cycle in a levitated ammonium-sulfate particle, Journal of Aerosol Science, 15, 563-571, 1984.

Rockland, L. B.: Saturated Salt Solutions for Static Control of Relative Humidity between 5° and 40°C., Analytical Chemistry, 32, 1375-1376, 1960.

Schlenker, J. C., Malinowski, A., Martin, S. T., Hung, H. M., and Rudich, Y.: Crystals formed at 293 K by aqueous sulfate-nitrate-ammonium-proton aerosol particles, Journal of Physical Chemistry A, 108, 9375-9383, 2004.

Schuttlefield, J., Al-Hosney, H., Zachariah, A., and Grassian, V. H.: Attenuated Total Reflection Fourier Transform Infrared Spectroscopy to Investigate Water Uptake and Phase Transitions in Atmospherically Relevant Particles, Applied Spectroscopy, 61, 283-292, 2007.

Tang, I. N., Fung, K. H., Imre, D. G., and Munkelwitz, H. R.: Phase Transformation and Metastability of Hygroscopic Microparticles, Aerosol Science and Technology, 23, 443-453, 1995.

Tang, I. N., and Munkelwitz, H. R.: Composition and temperature-dependence of the deliquescence properties of hygroscopic aerosols, Atmospheric Environment Part a-General Topics, 27, 467-473, 1993.

Tang, I. N., and Munkelwitz, H. R.: Water activities, densities, and refractive indices of aqueous sulfates and sodium nitrate droplets of atmospheric importance, Journal of Geophysical Research-Atmospheres, 99, 18801-18808, 1994.

Treuel, L., Schulze, S., Leisner, T., and Zellner, R.: Deliquescence behaviour of single levitated ternary salt/carboxylic acid/water microdroplets, Faraday Discussions, 137, 265-278, 2008.

Wise, M. E., Surratt, J. D., Curtis, D. B., Shilling, J. E., and Tolbert, M. A.: Hygroscopic growth of ammonium sulfate/dicarboxylic acids, Journal Of Geophysical Research-Atmospheres, 108, 2003.

Xu, J., Imre, D., McGraw, R., and Tang, I.: Ammonium sulfate: Equilibrium and metastability phase diagrams from 40 to -50 degrees C, Journal of Physical Chemistry B, 102, 7462-7469, 1998.

Yeung, M. C., and Chan, C. K.: Water Content and Phase Transitions in Particles of Inorganic and Organic Species and their Mixtures Using Micro-Raman Spectroscopy, Aerosol Science and Technology, 44, 269-280, 2010.

Zardini, A. A., Sjogren, S., Marcolli, C., Krieger, U. K., Gysel, M., Weingartner, E., Baltensperger, U., and Peter, T.: A combined particle trap/HTDMA hygroscopicity study of mixed inorganic/organic aerosol particles, Atmospheric Chemistry and Physics, 8, 5589-5601, 2008.

Zielinski, A. T., Gallimore, P. J., Griffiths, P. T., Jones, R. L., Seshia, A. A., and Kalberer, M.: Measuring Aerosol Phase Changes and Hygroscopicity with a Microresonator Mass Sensor, Analytical Chemistry, 90, 9716-9724, 2018.

## Li_2_SO_4_ (lithium sulfate)

| Reference | *T* (K) | *D* | DRH (%) | ERH (%) | Techniques/Comments |
| --- | --- | --- | --- | --- | --- |
| Rockland, 1960 | 278 | - | 84 | - | Nonisopiestic method |
|  | 283 |  | 84 |  |  |
|  | 288 |  | 84 |  |  |
|  | 293 |  | 85 |  |  |
|  | 298 |  | 85 |  |  |
|  | 303 |  | 85 |  |  |
|  | 308 |  | 85 |  |  |
|  | 313 |  | 81 |  |  |
| Goldberg, 1981 | 298 | - | 84.4 | - | Nonisopiestic method |
| Apelblat and Korin, 1998 | 278 | - | 72.0 | - | Nonisopiestic method |
|  | 283 |  | 75.0 |  |  |
|  | 288 |  | 77.7 |  |  |
|  | 293 |  | 80.1 |  |  |
|  | 298 |  | 82.1 |  |  |
|  | 303 |  | 83.8 |  |  |
|  | 308 |  | 85.1 |  |  |
|  | 313 |  | 86.1 |  |  |
|  | 318 |  | 86.8 |  |  |
|  | 323 |  | 87.2 |  |  |
| Rard et al., 2007 | 298 | - | 84.4 | - | Isopiestic method |
|  | 323 |  | 87.3 |  |  |

**Comments:**

The DRH of Li_2_SO_4_ was measured by four studies (Rockland, 1960; Goldberg, 1981; Apelblat and Korin, 1998; Rard et al., 2007), and it was determined to be 82-85% at 298 K, suggesting good agreement among these studies.


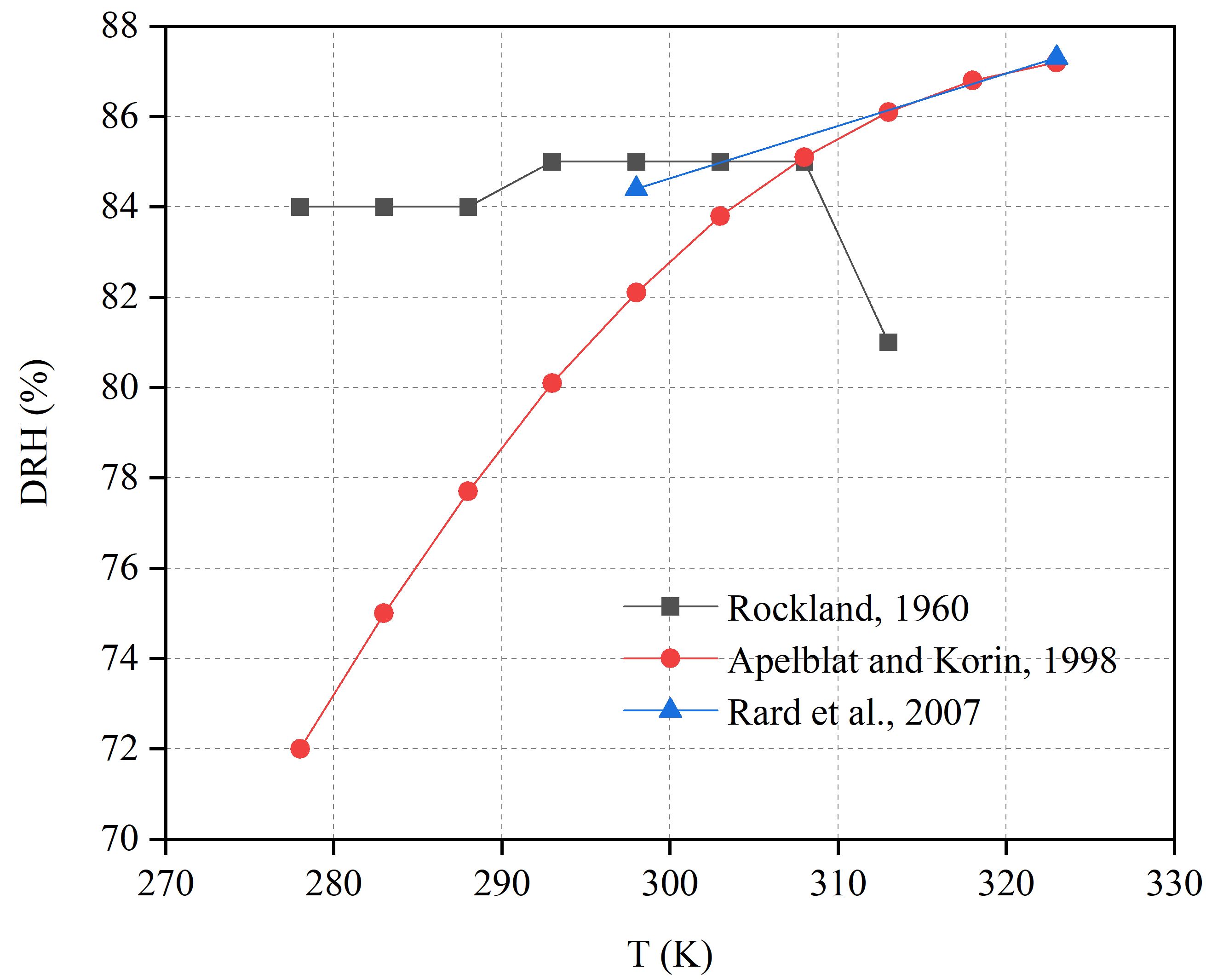


Three studies (Rockland, 1960; Apelblat and Korin, 1998; Rard et al., 2007) investigated the DRH of Li_2_SO_4_ at different temperature. Two studies showed similar temperature dependence: Apelblat and Korin (1998) found that the DRH of Li_2_SO_4_ increased with temperature from 72.0% at 278 K to 87.2% at 323 K, and Rard et al. (2007) suggested that it increased with temperature from 84.4% at 298 K to 87.3% at 323 K. The other study (Rockland, 1960) found that the measured DRH (84-85%) did not change significantly when temperature was increased from 278 to 308 K, and then decreased to 81% when temperature was further increased to 313 K.

**Preferred values at 298 K for Li_2_SO_4_:**

DRH: 82-85%

ERH: no preferred value

**References:**

Apelblat, A., and Korin, E.: Vapour pressures of saturated aqueous solutions of ammonium iodide, potassium iodide, potassium nitrate, strontium chloride, lithium sulphate, sodium thiosulphate, magnesium nitrate, and uranyl nitrate from T = (278 to 323) K, Journal of Chemical Thermodynamics, 30, 459-471, 1998.

Goldberg, R. N.: Evaluated activity and osmotic coefficients for aqueous solutions: thirty-six uni-bivalent electrolytes, Journal of Physical and Chemical Reference Data, 10, 671-764, 1981.

Rard, J. A., Clegg, S. L., and Palmer, D. A.: Isopiestic determination of the osmotic and activity coefficients of Li_2_SO_4_(aq) at T=298.15 and 323.15 k, and representation with an extended ion-interaction (Pitzer) model, Journal of Solution Chemistry, 36, 1347-1371, 2007.

Rockland, L. B.: Saturated Salt Solutions for Static Control of Relative Humidity between 5° and 40°C., Analytical Chemistry, 32, 1375-1376, 1960.

## Na_2_SO_4_ (sodium sulfate)

| Reference | *T* (K) | *D* | DRH (%) | ERH (%) | Techniques/Comments |
| --- | --- | --- | --- | --- | --- |
| Goldberg, 1981 | 298 | - | 93.6 | - | Nonisopiestic method |
| Cohen et al., 1987 | 293 | 20 μm | 85.4-86.5 | 55 | EDB |
| Tang and Munkelwitz, 1993 | 298 | 6-8 μm | 84.2±0.4 | - | EDB |
| Tang and Munkelwitz, 1994 | 298 | 6-8 μm | 84 | 57-59 | EDB |
| Tang et al., 1995 | 298 | 14-16 μm | 84 | 58 | EDB |
| Tang, 1996 | 298 | 6-8 μm | 84 | 57-59 | EDB |
| Lee and Hsu, 1998 | 296 | ~0.8 μm | 85±1 | 57±1 | Katharometer |
| Ebert et al., 2002 | 278 | 0.1-20 μm | 82.8±1.2 | - | ESEM |
| Lee and Chang, 2002 | 298 | 0.6-1.3 μm | 85 | 58 | Katharometer |
| Hiranuma et al., 2008 | 288 | ~20 μm | ~82 | - | ESEM |
| Ahn et al., 2010 | 298 | 2.5-10 μm | 84.4±0.3 | 58.5±0.6 | Optical microscopy |
| Darr et al., 2014 | 298 | 0.35 μm | 84±2 | 57±2 | FTIR |
| Feng et al., 2014 | 294 | 1-5 μm | 78-98 | 68-82 | ATR-FTIR |
| Zhang et al., 2020 | 288 | - | 84.5±1.0 | - | VSA |
|  | 293 |  | 85.5±1.0 |  |  |
|  | 298 |  | 86.5±1.0 |  |  |
|  | 303 |  | 87.5±1.0 |  |  |
|  | 308 |  | 88.5±1.0 |  |  |

**Comments:**

The DRH of Na_2_SO_4_ was measured by a number of studies (Cohen et al., 1987; Tang and Munkelwitz, 1993; Tang and Munkelwitz, 1994; Tang et al., 1995; Tang, 1996; Lee and Hsu, 1998; Ebert et al., 2002; Lee and Chang, 2002; Hiranuma et al., 2008; Ahn et al., 2010; Darr et al., 2014; Feng et al., 2014; Zhang et al., 2020), and it was determined to be 82-87% at 278-298 K by most of these studies, showing relatively good agreement. Moreover, the DRH of Na_2_SO_4_ was reported to be 93.6% at 298 K (Goldberg, 1981), significantly higher than those reported by the other studies.

One study (Zhang et al., 2020) measured DRH of Na_2_SO_4_ at different temperatures, and found that it increased slightly with temperature from 84.5±1.0% at 288 K to 88.5±1.0% at 308 K.

Eight studies (Cohen et al., 1987; Tang and Munkelwitz, 1994; Tang et al., 1995; Tang, 1996; Lee and Hsu, 1998; Lee and Chang, 2002; Ahn et al., 2010; Darr et al., 2014) measured the ERH values of Na_2_SO_4_. It was determined to be 55-59% at 293-298 K, showed good agreement. Moreover, it was measured to be 68-82% at 294 K (Feng et al., 2014), significantly higher than those reported by the other studies.

**Preferred values at 298 K for Na_2_SO_4_:**

DRH: 82-87%

ERH: 55-59%

**References:**

Ahn, K.-H., Kim, S.-M., Jung, H.-J., Lee, M.-J., Eom, H.-J., Maskey, S., and Ro, C.-U.: Combined Use of Optical and Electron Microscopic Techniques for the Measurement of Hygroscopic Property, Chemical Composition, and Morphology of Individual Aerosol Particles, Analytical Chemistry, 82, 7999-8009, 2010.

Cohen, M. D., Flagan, R. C., and Seinfeld, J. H.: Studies of concentrated electrolyte-solutions using the electrodynamic balance .1. Water activities for single-electrolyte solutions, Journal of Physical Chemistry, 91, 4563-4574, 1987.

Cohen, M. D., Flagan, R. C., and Seinfeld, J. H.: Studies of concentrated electrolyte solutions using the electrodynamic balance. 3. Solute nucleation, Journal of Physical Chemistry, 91, 4583-4590, 1987.

Darr, J. P., Davis, S. Q., Kohno, Y., McKenna, K., and Morales, P.: Morphological effects on the hygroscopic properties of sodium chloride-sodium sulfate aerosols, Journal of Aerosol Science, 77, 158-167, 2014.

Ebert, M., Inerle-Hof, M., and Weinbruch, S.: Environmental scanning electron microscopy as a new technique to determine the hygroscopic behaviour of individual aerosol particles, Atmospheric Environment, 36, 5909-5916, 2002.

Feng, X.-N., Chen, H.-N., Luan, Y.-M., Tan, S.-H., Pang, S.-F., and Zhang, Y.-H.: In-situ FTIR-ATR spectroscopic observation on the dynamic efflorescence/deliquescence processes of Na_2_SO_4_ and mixed Na_2_SO_4_/glycerol droplets, Chemical Physics, 430, 78-83, 2014.

Goldberg, R. N.: Evaluated activity and osmotic coefficients for aqueous solutions: thirty‐six uni‐bivalent electrolytes, Journal of Physical and Chemical Reference Data, 10, 671-764, 1981.

Hiranuma, N., Brooks, S. D., Auvermann, B. W., and Littleton, R.: Using environmental scanning electron microscopy to determine the hygroscopic properties of agricultural aerosols, Atmospheric Environment, 42, 1983-1994, 2008.

Lee, C. T., and Chang, S. Y.: A GC-TCD method for measuring the liquid water mass of collected aerosols, Atmospheric Environment, 36, 1883-1894, 2002.

Lee, C. T., and Hsu, W. C.: A novel method to measure aerosol water mass, Journal of Aerosol Science, 29, 827-837, 1998.

Tang, I. N.: Chemical and size effects of hygroscopic aerosols on light scattering coefficients, Journal of Geophysical Research-Atmospheres, 101, 19245-19250, 1996.

Tang, I. N., Fung, K. H., Imre, D. G., and Munkelwitz, H. R.: Phase Transformation and Metastability of Hygroscopic Microparticles, Aerosol Science and Technology, 23, 443-453, 1995.

Tang, I. N., and Munkelwitz, H. R.: Composition and temperature-dependence of the deliquescence properties of hygroscopic aerosols, Atmospheric Environment Part a-General Topics, 27, 467-473, 1993.

Tang, I. N., and Munkelwitz, H. R.: Water activities, densities, and refractive indices of aqueous sulfates and sodium nitrate droplets of atmospheric importance, Journal of Geophysical Research-Atmospheres, 99, 18801-18808, 1994.

Zhang, H., Gu, W., Li, Y. J., and Tang, M.: Hygroscopic properties of sodium and potassium salts as related to saline mineral dusts and sea salt aerosols, Journal of environmental sciences, 95, 65-72, 2020.

## K_2_SO_4_ (potassium sulfate)

| Reference | *T* (K) | *D* | DRH (%) | ERH (%) | Techniques/Comments |
| --- | --- | --- | --- | --- | --- |
| Adams and Merz, 1929 | 283 | - | 99.1 | - | Isopiestic method |
|  | 288 |  | 99.7 |  |  |
|  | 293 |  | 98.5 |  |  |
|  | 298 |  | 98.8 |  |  |
|  | 303 |  | 96.3 |  |  |
|  | 313 |  | 95.7 |  |  |
|  | 323 |  | 95.8 |  |  |
| Rockland, 1960 | 278 | - | 98 | - | Nonisopiestic method |
|  | 283 |  | 97 |  |  |
|  | 288 |  | 97 |  |  |
|  | 293 |  | 97 |  |  |
|  | 298 |  | 97 |  |  |
|  | 303 |  | 97 |  |  |
|  | 308 |  | 96 |  |  |
|  | 313 |  | 96 |  |  |
| Greenspan, 1977 | 273 | - | 98.7±1.1 | - | Nonisopiestic method |
|  | 278 |  | 98.5±0.9 |  |  |
|  | 283 |  | 98.2±0.8 |  |  |
|  | 288 |  | 97.9±0.6 |  |  |
|  | 293 |  | 97.6±0.5 |  |  |
|  | 298 |  | 97.3±0.5 |  |  |
|  | 303 |  | 97.0±0.4 |  |  |
|  | 308 |  | 96.7±0.4 |  |  |
|  | 313 |  | 96.4±0.4 |  |  |
|  | 318 |  | 96.1±0.4 |  |  |
|  | 323 |  | 95.8±0.5 |  |  |
| Goldberg, 1981 | 298 | - | 97.5 | - | Nonisopiestic method |
| Freney et al., 2009 | 288 | ~1 μm | >90 | 60±2 | ETEM |
|  |  |  | 96±1 | - | ESEM |
| Zhang et al., 2020 | 288-308 | - | >95 | - | VSA |

**Comments:**

The DRH of K_2_SO_4_ was measured by six studies (Adams and Merz, 1929; Rockland, 1960; Greenspan, 1977; Goldberg, 1981; Freney et al., 2009; Zhang et al., 2020), and it was determined to be 95-99% at 298 K, showing good agreement among these studies.


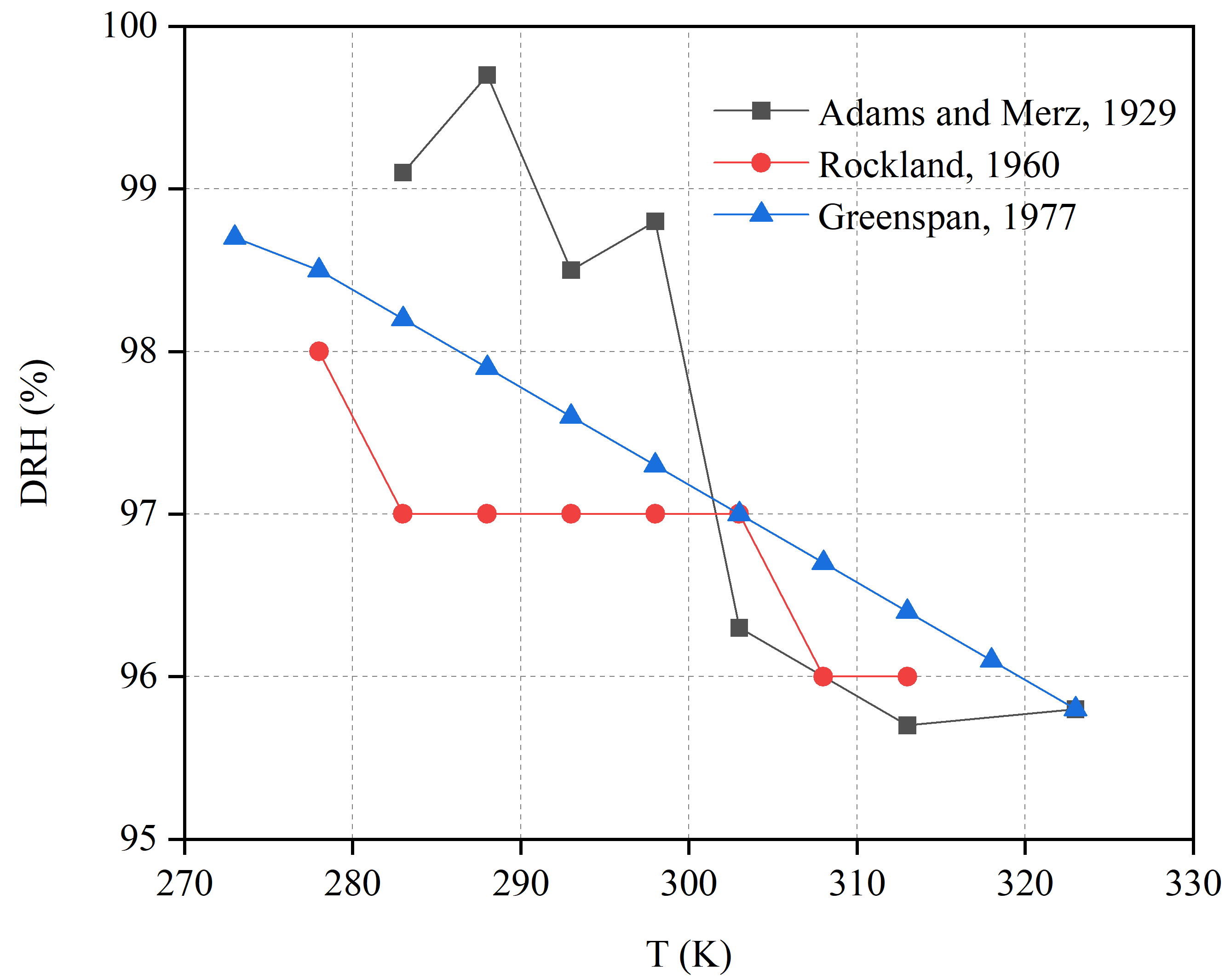


Three studies (Adams and Merz, 1929; Rockland, 1960; Greenspan, 1977) measured DRH of Li_2_SO_4_ at different temperature, and revealed similar temperature dependence. Adams and Merz (1929) found that it decreased with temperature from 99.1% at 283 K to 95.8% at 323K, Rockland (1960) suggested that it decreased with temperature from 98% at 278 K to 96% at 313 K, and Greenspan (1977) found that it decreased with temperature from 98.7±1.1% at 273 K to 95.8±0.5% at 323 K.

One study (Freney et al., 2009) measured the ERH of K_2_SO_4_, and it was determined to be 60±2% at 288 K.

**Preferred values at 298 K for K_2_SO_4_:**

DRH: 95-99%

ERH: 58-62%

**References:**

Adams, J. R., and Merz, A. R.: Hygroscopicity of fertilizer materials and mixtures, Industrial and Engineering Chemistry, 21, 305-307, 1929.

Freney, E. J., Martin, S. T., and Buseck, P. R.: Deliquescence and Efflorescence of Potassium Salts Relevant to Biomass-Burning Aerosol Particles, Aerosol Science and Technology, 43, 799-807, 2009.

Goldberg, R. N.: Evaluated activity and osmotic coefficients for aqueous solutions: thirty-six uni-bivalent electrolytes, Journal of Physical and Chemical Reference Data, 10, 671-764, 1981.

Greenspan, L.: Humidity fixed-points of binary saturated aqueous-solutions, Journal of Research of the National Bureau of Standards Section a-Physics and Chemistry, 81, 89-96, 1977.

Rockland, L. B.: Saturated Salt Solutions for Static Control of Relative Humidity between 5° and 40°C., Analytical Chemistry, 32, 1375-1376, 1960.

Zhang, H., Gu, W., Li, Y. J., and Tang, M.: Hygroscopic properties of sodium and potassium salts as related to saline mineral dusts and sea salt aerosols, Journal of environmental sciences, 95, 65-72, 2020.

## MgSO_4_ (magnesium sulfate) and MgSO_4_∙7H_2_O (magnesium sulfate heptahydrate)

| Species | Reference | *T* (K) | *D* | DRH (%) | ERH (%) | Techniques/Comments |
| --- | --- | --- | --- | --- | --- | --- |
| MgSO_4_ | Charlson et al., 1978 | 298 | - | 91 | - | Nonisopiestic method |
|  | Tang et al., 1997 | 298 | 6-8 μm | - | n. o. | EDB |
|  | Ha and Chan, 1999 | 296 | 10 μm | 86 | n. o. | EDB |
|  | Zhang and Chan, 2000 | 296 | 30 μm | - | n. o. | EDB |
|  | Choi and Chan, 2002 | 296 | 10-15 μm | 40 | - | EDB |
|  | Zhang and Chan, 2002 | 296 | 30-60 μm | - | n. o. | EDB-Raman |
|  | Apelblat and Manzurola, 2003 | 278 | - | 93.0 | - | Nonisopiestic method |
|  |  | 283 |  | 93.1 |  |  |
|  |  | 288 |  | 93.0 |  |  |
|  |  | 293 |  | 92.7 |  |  |
|  |  | 298 |  | 92.1 |  |  |
|  |  | 303 |  | 91.5 |  |  |
|  |  | 308 |  | 90.6 |  |  |
|  |  | 313 |  | 89.6 |  |  |
|  |  | 318 |  | 88.5 |  |  |
|  |  | 323 |  | 87.2 |  |  |
|  | Zhao et al., 2004 | 296 | - | 43±2.5 | - | ATR-FTIR |
|  | Wang et al., 2005 | 296 | ~80 μm | n. o. | n. o. | Micro-Raman |
|  | Wang et al., 2008 | 296 | 200-800 nm | 26.4±2.5 | - | ATR-FTIR |
|  |  |  | 2-20 μm | 41.8±2.5 | - |  |
| MgSO_4_∙7H_2_O | Zhang et al., 2021 | 298 | - | 88±0.6 | - | VSA |

**Comments:**

Seven studies (Charlson et al., 1978; Ha and Chan, 1999; Choi and Chan, 2002; Apelblat and Manzurola, 2003; Zhao et al., 2004; Wang et al., 2005; Wang et al., 2008) measured the DRH of MgSO_4_, and revealed different results.

Three studies (Choi and Chan, 2002; Zhao et al., 2004; Wang et al., 2008) suggested that the deliquescence of MgSO_4_ particles took place at 39-46% at 296 K; in addition, Wang et al. (2008) found that the DRH of MgSO_4_ at 296 K was closely related to the particle diameter: it was determined to be 26.4±2.5% for submicron particles (200-800 nm) and 41.8±2.5% for micron particles. Another three studies (Charlson et al., 1978; Ha and Chan, 1999; Apelblat and Manzurola, 2003) found that the deliquescence of MgSO_4_ at 296-298 K took place at 86-92% RH, significantly higher than those (39-46%) reported by the first three studies (Choi and Chan, 2002; Zhao et al., 2004; Wang et al., 2008). Finally, continuous water uptake was observed for MgSO_4_ with the increase in RH at 296 K (Wang et al., 2005), and thus no DRH was reported.

One study (Apelblat and Manzurola, 2003) measured the DRH of MgSO_4_ at different temperatures, and found that it decreased with temperature from 93.0% at 278 K to 87.2% at 323 K.

The ERH of MgSO_4_ was measured by five studies (Tang et al., 1997; Ha and Chan, 1999; Zhang and Chan, 2000; Zhang and Chan, 2002; Wang et al., 2005). Gradual water evaporation was observed for MgSO_4_ with the decrease in RH, and thus no ERH was reported.

The DRH of MgSO_4_∙7H_2_O was determined to be 88±0.6% at 298 K by Zhang et al. (2021).

**Preferred values at 298 K for MgSO_4_:**

DRH: no preferred value

ERH: no preferred value

**Preferred values at 298 K for MgSO_4_∙7H_2_O:**

DRH: 87-89%

ERH: no preferred value

**References:**

Apelblat, A., and Manzurola, E.: Solubilities and vapour pressures of saturated aqueous solutions of sodium tetraborate, sodium carbonate, and magnesium sulfate and freezing-temperature lowerings of sodium tetraborate and sodium carbonate solutions, Journal of Chemical Thermodynamics, 35, 221-238, 2003.

Charlson, R. J., Covert, D. S., Larson, T. V., and Waggoner, A. P.: Chemical properties of tropospheric sulfur aerosols, Atmospheric Environment (1967), 12, 39-53, 1978.

Choi, M. Y., and Chan, C. K.: Continuous measurements of the water activities of aqueous droplets of water-soluble organic compounds, Journal Of Physical Chemistry A, 106, 4566-4572, 2002.

Ha, Z., and Chan, C. K.: The Water Activities of MgCl_2_, Mg(NO_3_)_2_, MgSO_4_, and Their Mixtures, Aerosol Science and Technology, 31, 154-169, 1999.

Tang, I. N., Tridico, A. C., and Fung, K. H.: Thermodynamic and optical properties of sea salt aerosols, Journal of Geophysical Research-Atmospheres, 102, 23269-23275, 1997.

Wang, F., Zhang, Y. H., Li, S. H., Wang, L. Y., and Zhao, L. J.: A strategy for single supersaturated droplet analysis: Confocal Raman investigations on the complicated hygroscopic properties of individual MgSO4 droplets on the quartz substrate, Analytical Chemistry, 77, 7148-7155, 2005.

Wang, L. Y., Ding, F., Zhang, Y. H., Zhao, L. J., and Hu, Y. A.: Anomalous hygroscopic growth of fine particles of MgSO_4_ aerosols investigated by FTIR/ATR spectroscopy, Spectrochimica Acta Part a-Molecular and Biomolecular Spectroscopy, 71, 682-687, 2008.

Zhang, Y. H., and Chan, C. K.: Study of contact ion pairs of supersaturated magnesium sulfate solutions using raman scattering of levitated single droplets, Journal of Physical Chemistry A, 104, 9191-9196, 2000.

Zhang, Y. H., and Chan, C. K.: Understanding the hygroscopic properties of supersaturated droplets of metal and ammonium sulfate solutions using Raman spectroscopy, Journal of Physical Chemistry A, 106, 285-292, 2002.

Zhang, Q.-N., Zhao, L.-J., Chen, S.-H., Guo, X., Luan, Y.-M., and Zhang, Y.-H.: Hygroscopic property of inorganic salts in atmospheric aerosols measured with physisorption analyzer, Atmospheric Environment, 247, 2021.

Zhao, L. J., Zhang, Y. H., Wang, L. Y., and Cheng, H.: FTIR in situ study of supersaturated solutions of MgSO_4_ and NaClO_4_ aerosols, Chinese Science Bulletin, 49, 1699-1701, 2004.

# Bisulfates

## NH_4_HSO_4_ (ammonium bisulfate)

| Reference | *T* (K) | *D* | DRH (%) | ERH (%) | Techniques/Comments |
| --- | --- | --- | --- | --- | --- |
| Tang and Munkelwitz, 1977 | 298 | 0.4-1 μm | 39.0±0.5 | - | H-DMA-OPC |
|  | 298 | - | 39.7 | - | Nonisopiestic method |
| Spann and Richardson, 1985 | 298 | ~1 μm | 40 | n. o. (<1) | EDB |
| Tang and Munkelwitz, 1994 | 298 | 6-8 μm | 40 | 0.05-22 | EDB |
| Cziczo et al., 1997 | 298 | 0.45 μm | 39±2 | n. o. (<2) | FTIR |
| Imre et al., 1997 | 243 | 5-10 μm | 64 | - | EDB |
|  | 248 |  | 58 | 34 |  |
|  | 253 |  | 57 | - |  |
|  | 258 |  | 55 | - |  |
|  | 263 |  | 51 | - |  |
|  | 274 |  | 49 | - |  |
|  | 298 |  | 40 | - |  |
| Cziczo and Abbatt, 2000 | 238-298 | 0.18 μm | - | n. o. (<2) | FTIR |
| Lee and Hsu, 2000 | 295 | ~1 μm | n. o. | n. o. | Katharometer |

**Comments:**

Five studies (Tang and Munkelwitz, 1977; Spann and Richardson, 1985; Tang and Munkelwitz, 1994; Cziczo et al., 1997; Imre et al., 1997) measured the DRH of NH_4_HSO_4_, and it was determined to be 37-41% at 298 K, showing good agreement among these studies. Moreover, Lee and Hsu (2000) found that NH_4_HSO_4_ displayed continuous hygroscopic growth at 295 K, and thus no DRH was reported.

One study (Imre et al., 1997) measured the DRH of NH_4_HSO_4_ at different temperature, and it was found to decreased with temperature from 64% at 243 K to 40% at 298 K.

The ERH of NH_4_HSO_4_ was measured by six studies (Spann and Richardson, 1985; Tang and Munkelwitz, 1994; Cziczo et al., 1997; Imre et al., 1997; Cziczo and Abbatt, 2000; Lee and Hsu, 2000). Gradual water evaporation was observed in four studies (Spann and Richardson, 1985; Cziczo et al., 1997; Cziczo and Abbatt, 2000; Lee and Hsu, 2000), thus no ERH was reported. In addition, Tang and Munkelwitz (1994) found that the efflorescence of NH_4_HSO_4_ took place at 0.05-22% RH at 298 K, and its ERH was measured to be 34% at 248 K by Imre et al. (1997).

**Preferred values at 298 K for NH_4_HSO_4_:**

DRH: 37-41%

ERH: no preferred value

**References:**

Cziczo, D. J., Nowak, J. B., Hu, J. H., and Abbatt, J. P. D.: Infrared spectroscopy of model tropospheric aerosols as a function of relative humidity: Observation of deliquescence and crystallization, Journal of Geophysical Research-Atmospheres, 102, 18843-18850, 1997.

Cziczo, D. J., and Abbatt, J. P. D.: Infrared observations of the response of NaCl, MgCl_2_, NH_4_HSO_4_, and NH_4_NO_3_ aerosols to changes in relative humidity from 298 to 238 K, Journal of Physical Chemistry A, 104, 2038-2047, 2000.

Imre, D. G., Xu, J., Tang, I. N., and McGraw, R.: Ammonium bisulfate water equilibrium and metastability phase diagrams, Journal of Physical Chemistry A, 101, 4191-4195, 1997.

Lee, C.-T., and Hsu, W.-C.: The measurement of liquid water mass associated with collected hygroscopic particles, Journal of Aerosol Science, 31, 189-197, 2000.

Spann, J. F., and Richardson, C. B.: Measurement of the water cycle in mixed ammonium acid sulfate particles, Atmospheric Environment, 19, 819-825, 1985.

Tang, I. N., and Munkelwitz, H. R.: Aerosol growth studies-III ammonium bisulfate aerosols in a moist atmosphere, Journal Of Aerosol Science, 8, 321-330, 1977.

Tang, I. N., and Munkelwitz, H. R.: Water activities, densities, and refractive indices of aqueous sulfates and sodium nitrate droplets of atmospheric importance, Journal of Geophysical Research-Atmospheres, 99, 18801-18808, 1994.

## NaHSO_4_ (sodium bisulfate)

| Reference | *T* (K) | *D* | DRH (%) | ERH (%) | Techniques/Comments |
| --- | --- | --- | --- | --- | --- |
| Tang and Munkelwitz, 1994 | 298 | 6-8 μm | - | n. o. | EDB |

**Comments:**

Tang and Munkelwitz (1994) investigated the efflorescence of NH_4_HSO_4_ at 298 K. Continuous loss of water was observed and no crystallization occurred; therefore, no ERH was reported.

**Preferred values at 298 K for NaHSO_4_:**

DRH: no preferred value

ERH: no preferred value

**References:**

Tang, I. N., and Munkelwitz, H. R.: Water activities, densities, and refractive indices of aqueous sulfates and sodium nitrate droplets of atmospheric importance, Journal of Geophysical Research-Atmospheres, 99, 18801-18808, 1994.

## KHSO_4_ (potassium bisulfate)

| Reference | *T* (K) | *D* | DRH (%) | ERH (%) | Techniques/Comments |
| --- | --- | --- | --- | --- | --- |
| Tang et al., 1995 | 298 | 14-16 μm | not reported | not reported | EDB |

**Comments:**

Tang et al. (1995) examined phase transformation of KHSO_4_ as a function of RH at 298 K, but they did not report its DRH and ERH.

**Preferred values at 298 K for KHSO_4_:**

DRH: no preferred value

ERH: no preferred value

**References:**

Tang, I. N., Fung, K. H., Imre, D. G., and Munkelwitz, H. R.: Phase Transformation and Metastability of Hygroscopic Microparticles, Aerosol Science and Technology, 23, 443-453, 1995.

# Nitrates

## NH_4_NO_3_ (ammonium nitrate)

| Reference | *T* (K) | *D* | DRH (%) | ERH (%) | Techniques/Comments |
| --- | --- | --- | --- | --- | --- |
| Adams and Merz, 1929 | 283 | - | 75.3 | - | Isopiestic method |
|  | 288 |  | 69.8 |  |  |
|  | 293 |  | 66.9 |  |  |
|  | 298 |  | 62.7 |  |  |
|  | 303 |  | 59.4 |  |  |
|  | 313 |  | 52.5 |  |  |
|  | 323 |  | 48.4 |  |  |
| Richardson and Hightower, 1987 | 298 | ~2 μm | 62.2±0.8 | n. o. | EDB |
| Chan et al., 1992 | 298 | ~30 μm | - | ~30 | EDB |
| Apelblat, 1993 | 283 | - | 69.5 | - | Nonisopiestic method |
|  | 288 |  | 67.7 |  |  |
|  | 293 |  | 65.2 |  |  |
|  | 298 |  | 62.3 |  |  |
|  | 303 |  | 59.0 |  |  |
|  | 308 |  | 55.5 |  |  |
|  | 313 |  | 51.8 |  |  |
| Tang and Munkelwitz, 1993 | 298 | 6-8 μm | 61.8 | - | EDB |
| Tang, 1996 | 298 | 6-8 μm | 62 | 25-32 | EDB |
| Dougle et al., 1998 | 298 | ~0.5 μm | 60 | n. o. (<8) | Nephelometer |
| Cziczo and Abbatt, 2000 | 238-298 | 0.18 μm | - | n. o. (<1) | FTIR |
| Lee and Hsu, 2000 | 294 | ~1 μm | 62 | 32 | Katharometer |
| Lightstone et al., 2000 | 298 | ~8 μm | 61.8±0.3 | n. o. (<1) | EDB |
| ten Brink et al., 2000 | 298 | ~0.5 μm | 62 | <8 | Nephelometer |
| Ebert et al., 2002 | 278 | 0.1-20 μm | 74.0±0.8 | - | ESEM |
|  | 280.5 |  | 72.7±1.1 |  |  |
|  | 283 |  | 71.8±1.2 |  |  |
|  | 285.5 |  | 69.7±1.0 |  |  |
|  | 288 |  | 67.8±0.9 |  |  |
| Han et al., 2002 | 298 | 50-1000 nm | - | n. o. (<1) | FTIR |
| Schuttlefield et al., 2007 | 298 | - | 64±1 | - | ATR-FTIR |
| Ma et al., 2010 | 274 | 1-30 μm | 79±1 | - | PSA |
|  | 278 |  | 75±1 |  |  |
|  | 283 |  | 70±1 |  |  |
|  | 288 |  | 68±1 |  |  |
| Yeung and Chan, 2010 | 297 | 10-30 μm | 63 | 0-30 | Micro-Raman |
| Hu et al., 2011 | 298 | 40-200 nm | n. o. | - | HTDMA |
| Arenas et al., 2012 | 298 | - | 65.5±1.0 | - | QCM |
| Guo et al., 2012 | 296 | 5-20 μm | - | 34±2.5 | Micro-Raman |
| Jing et al., 2018 | 298 | 100 nm | n. o. | - | HTDMA |

**Comments:**

The DRH of NH_4_NO_3_ was measured by a number of studies (Adams and Merz, 1929; Richardson and Hightower, 1987; Apelblat, 1993; Tang and Munkelwitz, 1993; Tang, 1996; Dougle et al., 1998; Lee and Hsu, 2000; Lightstone et al., 2000; ten Brink et al., 2000; Ebert et al., 2002; Schuttlefield et al., 2007; Ma et al., 2010; Yeung and Chan, 2010; Arenas et al., 2012). It was determined to be 60-66% at 298 K, showing good agreement among these studies. Moreover, Hu et al. (2011) and Jing et al. (2018) both found that NH_4_NO_3_ displayed continuous hygroscopic growth at 298 K, and thus no DRH was reported in their studies.


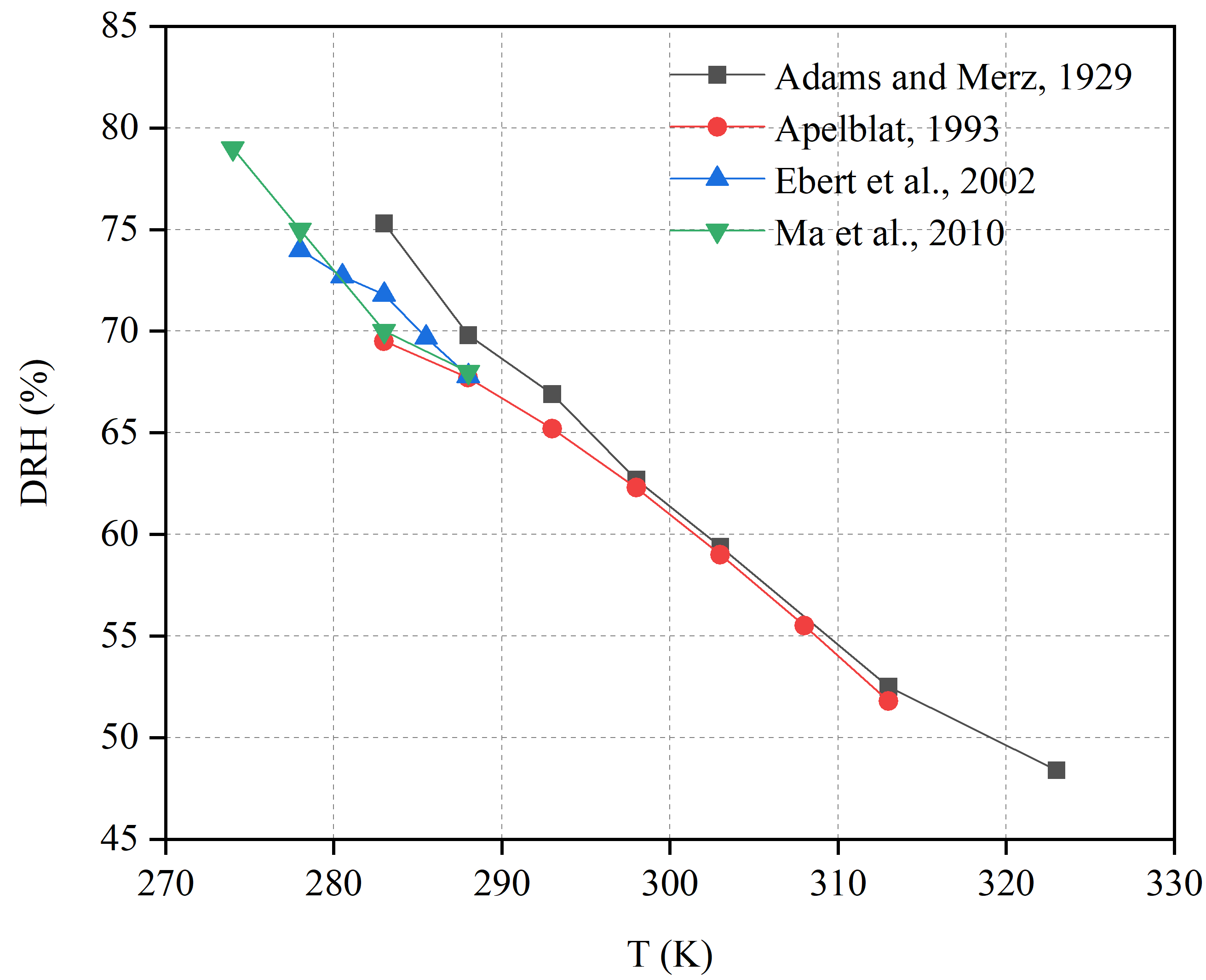


Four studies (Adams and Merz, 1929; Apelblat, 1993; Ebert et al., 2002; Ma et al., 2010) measured the DRH of NH_4_NO_3_ at different temperature, and revealed similar temperature dependence. To be more specific, Adams and Merz (1929) found that it decreased with temperature from 75.3% at 283 K to 48.4% at 323 K, Apelblat (1993) found that it decreased with temperature from 69.5% at 283 K to 51.8% at 313 K, Ebert et al. (2002) suggested that it decreased with temperature from 74.0±0.8% at 278 K to 67.8±0.9% at 288 K, and Ma et al. (2010) suggested that it decreased with temperature from 79±1% at 274 K to 68±1% at 288 K.

The ERH of NH_4_NO_3_ was measured by a number of studies (Richardson and Hightower, 1987; Chan et al., 1992; Tang, 1996; Dougle et al., 1998; Cziczo and Abbatt, 2000; Lee and Hsu, 2000; Lightstone et al., 2000; ten Brink et al., 2000; Han et al., 2002; Yeung and Chan, 2010; Guo et al., 2012).

Four studies (Chan et al., 1992; Tang, 1996; Lee and Hsu, 2000; Guo et al., 2012) suggested that the efflorescence of NH_4_NO_3_ took place at 25-36% RH at 294-298 K. Gradual water evaporation was observed in the five other studies (Richardson and Hightower, 1987; Dougle et al., 1998; Cziczo and Abbatt, 2000; Lightstone et al., 2000; Han et al., 2002), thus no ERH was reported. In addition, Yeung and Chan (2010) observed that the efflorescence of NH_4_NO_3_ occurred stochastically in the range of 0-30% RH at 297 K.

**Preferred values at 298 K for NH_4_NO_3_:**

DRH: 60-66%

ERH: 25-36%

**References:**

Adams, J. R., and Merz, A. R.: Hygroscopicity of fertilizer materials and mixtures, Industrial and Engineering Chemistry, 21, 305-307, 1929.

Apelblat, A.: The vapor-pressures of saturated aqueous lithium-chloride, sodium-bromide, sodium-nitrate, ammonium-nitrate, and ammonium-chloride at temperatures from 283 K to 313 K, Journal of Chemical Thermodynamics, 25, 63-71, 1993.

Arenas, K. J. L., Schill, S. R., Malla, A., and Hudson, P. K.: Deliquescence Phase Transition Measurements by Quartz Crystal Microbalance Frequency Shifts, Journal of Physical Chemistry A, 116, 7658-7667, 2012.

Chan, C. K., Flagan, R. C., and Seinfeld, J. H.: Water activities of NH_4_NO_3_/(NH_4_)_2_SO_4_ solutions, Atmospheric Environment. Part A. General Topics, 26, 1661-1673, 1992.

Cziczo, D. J., and Abbatt, J. P. D.: Infrared observations of the response of NaCl, MgCl_2_, NH_4_HSO_4_, and NH_4_NO_3_ aerosols to changes in relative humidity from 298 to 238 K, Journal of Physical Chemistry A, 104, 2038-2047, 2000.

Dougle, P. G., Veefkind, J. P., and ten Brink, H. M.: Crystallisation of mixtures of ammonium nitrate, ammonium sulphate and soot, Journal of Aerosol Science, 29, 375-386, 1998.

Ebert, M., Inerle-Hof, M., and Weinbruch, S.: Environmental scanning electron microscopy as a new technique to determine the hygroscopic behaviour of individual aerosol particles, Atmospheric Environment, 36, 5909-5916, 2002.

Guo, X., Tan, S.-H., Shang, Z.-J., Guo, Y.-C., and Zhang, Y.-H.: Confocal Raman Spectroscopy Studies on the Interactions between NH_4_^+^, NO_3_^-^ and H_2_O in Supersaturated NH_4_NO_3_ Droplets, Acta Physico-Chimica Sinica, 28, 766-772, 2012.

Han, J. H., Hung, H. M., and Martin, S. T.: Size effect of hematite and corundum inclusions on the efflorescence relative humidities of aqueous ammonium nitrate particles, Journal of Geophysical Research-Atmospheres, 107, 2002.

Hu, D., Chen, J., Ye, X., Li, L., and Yang, X.: Hygroscopicity and evaporation of ammonium chloride and ammonium nitrate: Relative humidity and size effects on the growth factor, Atmospheric Environment, 45, 2349-2355, 2011.

Lee, C.-T., and Hsu, W.-C.: The measurement of liquid water mass associated with collected hygroscopic particles, Journal of Aerosol Science, 31, 189-197, 2000.

Lightstone, J. M., Onasch, T. B., Imre, D., and Oatis, S.: Deliquescence, efflorescence, and water activity in ammonium nitrate and mixed ammonium nitrate/succinic acid microparticles, Journal of Physical Chemistry A, 104, 9337-9346, 2000.

Ma, Q., Liu, Y., and He, H.: The Utilization of Physisorption Analyzer for Studying the Hygroscopic Properties of Atmospheric Relevant Particles, Journal of Physical Chemistry A, 114, 4232-4237, 2010.

Ma, Q., Zhong, C., Liu, C., Liu, J., Ma, J., Wu, L., and He, H.: A Comprehensive Study about the Hygroscopic Behavior of Mixtures of Oxalic Acid and Nitrate Salts: Implication for the Occurrence of Atmospheric Metal Oxalate Complex, Acs Earth and Space Chemistry, 3, 1216-1225, 2019.

Richardson, C. B., and Hightower, R. L.: Evaporation of ammonium nitrate particles, Atmospheric Environment, 21, 971-975, 1987.

Schuttlefield, J., Al-Hosney, H., Zachariah, A., and Grassian, V. H.: Attenuated Total Reflection Fourier Transform Infrared Spectroscopy to Investigate Water Uptake and Phase Transitions in Atmospherically Relevant Particles, Applied Spectroscopy, 61, 283-292, 2007.

Tang, I. N.: Chemical and size effects of hygroscopic aerosols on light scattering coefficients, Journal of Geophysical Research-Atmospheres, 101, 19245-19250, 1996.

Tang, I. N., and Munkelwitz, H. R.: Composition and temperature-dependence of the deliquescence properties of hygroscopic aerosols, Atmospheric Environment Part a-General Topics, 27, 467-473, 1993.

ten Brink, H. M., Khlystov, A., Kos, G. P. A., Tuch, T., Roth, C., and Kreyling, W.: A high-flow humidograph for testing the water uptake by ambient aerosol, Atmospheric Environment, 34, 4291-4300, 2000.

Yeung, M. C., and Chan, C. K.: Water Content and Phase Transitions in Particles of Inorganic and Organic Species and their Mixtures Using Micro-Raman Spectroscopy, Aerosol Science and Technology, 44, 269-280, 2010.

## LiNO_3_ (lithium nitrate) and LiNO_3_∙3H_2_O (lithium nitrate trihydrate)

| Species | Reference | *T* (K) | *D* | DRH (%) | ERH (%) | Techniques/Comments |
| --- | --- | --- | --- | --- | --- | --- |
| LiNO_3_ | Pearce and Nelson, 1932 | 298 | - | 44.2 | - | Nonisopiestic method |
|  | Rockland, 1960 | 278 | - | 61 | - | Nonisopiestic method |
|  |  | 283 |  | 59 |  |  |
|  |  | 288 |  | 55 |  |  |
|  |  | 293 |  | 49 |  |  |
|  |  | 298 |  | 41 |  |  |
|  |  | 303 |  | 31 |  |  |
|  |  | 308 |  | 19 |  |  |
|  |  | 313 |  | 11 |  |  |
| LiNO_3_∙3H_2_O | Stokes and Robinson, 1949 | 298 | - | 47.1 | - | Nonisopiestic method |

**Comments:**

The DRH of LiNO_3_ was measured by two studies (Pearce and Nelson, 1932; Rockland, 1960). It was determined to be 41-44% at 298 K, suggesting good agreement. Rockland (1960) further investigated its dependence on temperature, and found that the measured DRH significantly decreased with temperature from 61% at 278 K to 11% at 313 K.

The DRH of LiNO_3_∙3H_2_O was measured to be 47.1% at 298 K (Stokes and Robinson, 1949).

**Preferred values at 298 K for LiNO_3_:**

DRH: 41-44%

ERH: no preferred value

**Preferred values at 298 K for LiNO_3_∙3H_2_O:**

DRH: 47%

ERH: no preferred value

**References:**

Pearce, J. N., and Nelson, A. F.: The vapor pressures of aqueous solutions of lithium nitrate and the activity coefficients of some alkali salts in solutions of high concentration at 25 degrees, Journal of the American Chemical Society, 54, 3544-3555, 1932.

Rockland, L. B.: Saturated Salt Solutions for Static Control of Relative Humidity between 5° and 40°C., Analytical Chemistry, 32, 1375-1376, 1960.

Stokes, R. H., and Robinson, R. A.: Standard Solutions for Humidity Control at 25°C., Industrial and Engineering Chemistry, 41, 2013-2013, 1949.

## NaNO_3_ (sodium nitrate)

| Reference | *T* (K) | *D* | DRH (%) | ERH (%) | Techniques/Comments |
| --- | --- | --- | --- | --- | --- |
| Adams and Merz, 1929 | 283 | - | 78 | - | Isopiestic method |
|  | 288 |  | 76.8 |  |  |
|  | 293 |  | 77.1 |  |  |
|  | 298 |  | 74.4 |  |  |
|  | 303 |  | 72.4 |  |  |
|  | 313 |  | 70.1 |  |  |
|  | 323 |  | 67.3 |  |  |
| Stokes and Robinson, 1949 | 298 | - | 73.8 | - | Nonisopiestic method |
| Greenspan, 1977 | 278 | - | 78.6±0.5 | - | Nonisopiestic method |
|  | 283 |  | 77.5±0.5 |  |  |
|  | 288 |  | 76.5±1.4 |  |  |
|  | 293 |  | 75.4±0.4 |  |  |
|  | 298 |  | 74.3±0.3 |  |  |
|  | 303 |  | 73.1±0.3 |  |  |
|  | 308 |  | 72.1±0.3 |  |  |
|  | 313 |  | 71.0±0.3 |  |  |
|  | 318 |  | 70.0±0.4 |  |  |
|  | 323 |  | 69.0±0.4 |  |  |
|  | 328 |  | 68.2±0.5 |  |  |
|  | 333 |  | 67.4±0.6 |  |  |
|  | 338 |  | 66.6±0.7 |  |  |
|  | 343 |  | 66.0±0.8 |  |  |
|  | 348 |  | 65.6±0.9 |  |  |
|  | 353 |  | 65.2±1.1 |  |  |
|  | 358 |  | 65.0±1.2 |  |  |
|  | 363 |  | 65.0±1.4 |  |  |
| Apelblat, 1993 | 283 | - | 76.3 | - | Nonisopiestic method |
|  | 288 |  | 76.0 |  |  |
|  | 293 |  | 75.1 |  |  |
|  | 298 |  | 73.9 |  |  |
|  | 303 |  | 72.3 |  |  |
|  | 308 |  | 70.3 |  |  |
|  | 313 |  | 68.1 |  |  |
| Tang and Munkelwitz, 1993 | 298 | 6-8 μm | 74.3±0.4 | - | EDB |
| Richardson and Snyder, 1994 | 298 | 10 μm | 74.3 | n. o. (<1) | EDB |
| Tang and Munkelwitz, 1994 | 298 | 6-8 μm | 74.5 | 0.05-30 | EDB |
| Lamb et al., 1996 | 298 | 20 μm | 72-76 | 36-44 | EDB |
| Tang, 1996 | 298 | 6-8 μm | 74.5 | 0.05-30 | EDB |
| Tang and Fung, 1997 | 298 | 14-16 μm | 74.5 | ~35 | EDB |
| Apelblat and Korin, 1998 | 278 | - | 70.7 | - | Nonisopiestic method |
|  | 283 |  | 72.0 |  |  |
|  | 288 |  | 73.0 |  |  |
|  | 293 |  | 73.5 |  |  |
|  | 298 |  | 73.7 |  |  |
|  | 303 |  | 73.5 |  |  |
|  | 308 |  | 73.0 |  |  |
|  | 313 |  | 72.3 |  |  |
|  | 318 |  | 71.2 |  |  |
|  | 323 |  | 69.9 |  |  |
| Lee and Hsu, 2000 | 294 | ~1 μm | n. o. | n. o. | Katharometer |
| Gysel et al., 2002 | 263 | 100 nm | n. o. | n. o. | HTDMA |
|  | 293 |  | n. o. | n. o. |  |
| Hoffman et al., 2004 | 278 | 0.1-10 μm |  |  | ESEM |
|  |  |  | n. o. | n. o. | atomized particles |
|  |  |  | ~75 | n. o. | crystalline particles |
| Gibson et al., 2006 | 298 | 100 nm | n. o. | - | HTDMA |
| Liu et al., 2008 | 296 | ~1μm | 71-73 | n. o. | FTIR |
| Lu et al., 2008 | 298 | 1-5 μm | n. o. | n. o. | ATR-FTIR |
| Ghorai and Tivanski, 2010 | 299 | ~1 μm | ~75 | ~35 | STXM |
| Kim et al., 2012 | 297 | 2.5-4.0 μm |  |  | Optical microscopy |
|  |  |  | n. o. | n. o. | wet dispersed particles |
|  |  |  | 73.5-74.2 | 26.7-47.1 | dry deposited particles |
| Zhang et al., 2014 | 298 | 1-5 μm | - | 62.5±2.5 | ATR-FTIR |
| Gupta et al., 2015 | 296 | 1-10 μm |  |  | Micro-Raman |
|  |  |  | n. o. | n. o. | wet dispersed particles |
|  |  |  | 74.0±0.5 | 26.7-45.7 | dry deposited particles |
| Ji et al., 2017 | 298 | 2-8 μm | - | ~45 | Vacuum-FTIR |
| Jing et al., 2018 | 298 | 100 nm | n. o. | - | HTDMA |
| Ma et al., 2019 | 298 | 300 nm | n. o. | - | HTDMA |
| Xie et al., 2020 | 298 | 90 nm | n. o. | - | SPRM (Surface Plasmon Resonance Microscopy) |
| Zhang et al., 2020 | 288 | - | 74.5±1.0 | - | VSA |
|  | 293 |  | 74.5±1.0 |  |  |
|  | 298 |  | 73.5±1.0 |  |  |
|  | 303 |  | 72.5±1.0 |  |  |
|  | 308 |  | 71.5±1.0 |  |  |
| Zhang et al., 2021 | 298 | - | 74.5±0.6 | - | VSA |

**Comments:**

A large number of studies (Adams and Merz, 1929; Stokes and Robinson, 1949; Greenspan, 1977; Apelblat, 1993; Tang and Munkelwitz, 1993; Richardson and Snyder, 1994; Tang and Munkelwitz, 1994; Lamb et al., 1996; Tang, 1996; Tang and Fung, 1997; Apelblat and Korin, 1998; Lee and Hsu, 2000; Gysel et al., 2002; Hoffman et al., 2004; Gibson et al., 2006; Liu et al., 2008; Lu et al., 2008; Ghorai and Tivanski, 2010; Kim et al., 2012; Zhang et al., 2014; Gupta et al., 2015; Ji et al., 2017; Jing et al., 2018; Ma et al., 2019; Xie et al., 2020; Zhang et al., 2020; Zhang et al., 2021) measured the DRH of NaNO_3_. Most of these studies (Adams and Merz, 1929; Stokes and Robinson, 1949; Greenspan, 1977; Apelblat, 1993; Tang and Munkelwitz, 1993; Richardson and Snyder, 1994; Tang and Munkelwitz, 1994; Lamb et al., 1996; Tang, 1996; Tang and Fung, 1997; Apelblat and Korin, 1998; Liu et al., 2008; Ghorai and Tivanski, 2010; Zhang et al., 2020; Zhang et al., 2021) showed good agreement, and it was determined to be 71-76% at 296-299 K. Several other studies (Lee and Hsu, 2000; Gysel et al., 2002; Gibson et al., 2006; Lu et al., 2008; Jing et al., 2018; Ma et al., 2019; Xie et al., 2020) found that NaNO_3_ displayed continuous hygroscopic growth, and thus no DRH was reported.

Three studies (Hoffman et al., 2004; Kim et al., 2012; Gupta et al., 2015) suggested that hygroscopic behavior of NaNO_3_ was closely related to how particles were generated: deliquescence took place at 73-75% at 278-297 K for dry deposited (or crystalline) particles, and wet dispersed (or amorphous) particles displayed continuous water uptake.

The DRH of NaNO_3_ at different temperature was measured by five studies (Adams and Merz, 1929; Greenspan, 1977; Apelblat, 1993; Apelblat and Korin, 1998; Zhang et al., 2020). Four studies (Adams and Merz, 1929; Greenspan, 1977; Apelblat, 1993; Zhang et al., 2020) showed similar temperature dependence: Adams and Merz (1929) found that it decreased with temperature from 78% at 283 K to 67.3% at 323 K, Greenspan (1977) found that it decreased with temperature from 78.6±0.5% at 278 K to 65.0±1.4% at 363 K, Apelblat (1993) suggested that it decreased with temperature from 76.3% at 283 K to 68.1% at 313 K, and Zhang et al. (2020) suggested that it decreased with temperature from 74.5±1.0% at 288 K to 71.5±1.0% at 308 K. Moreover, Apelblat and Korin (1998) found that the DRH of NaNO_3_ first increased with temperature from 70.7% at 278 K to 73.7% at 298 K, then decreased with temperature to 69.9% at 323 K.


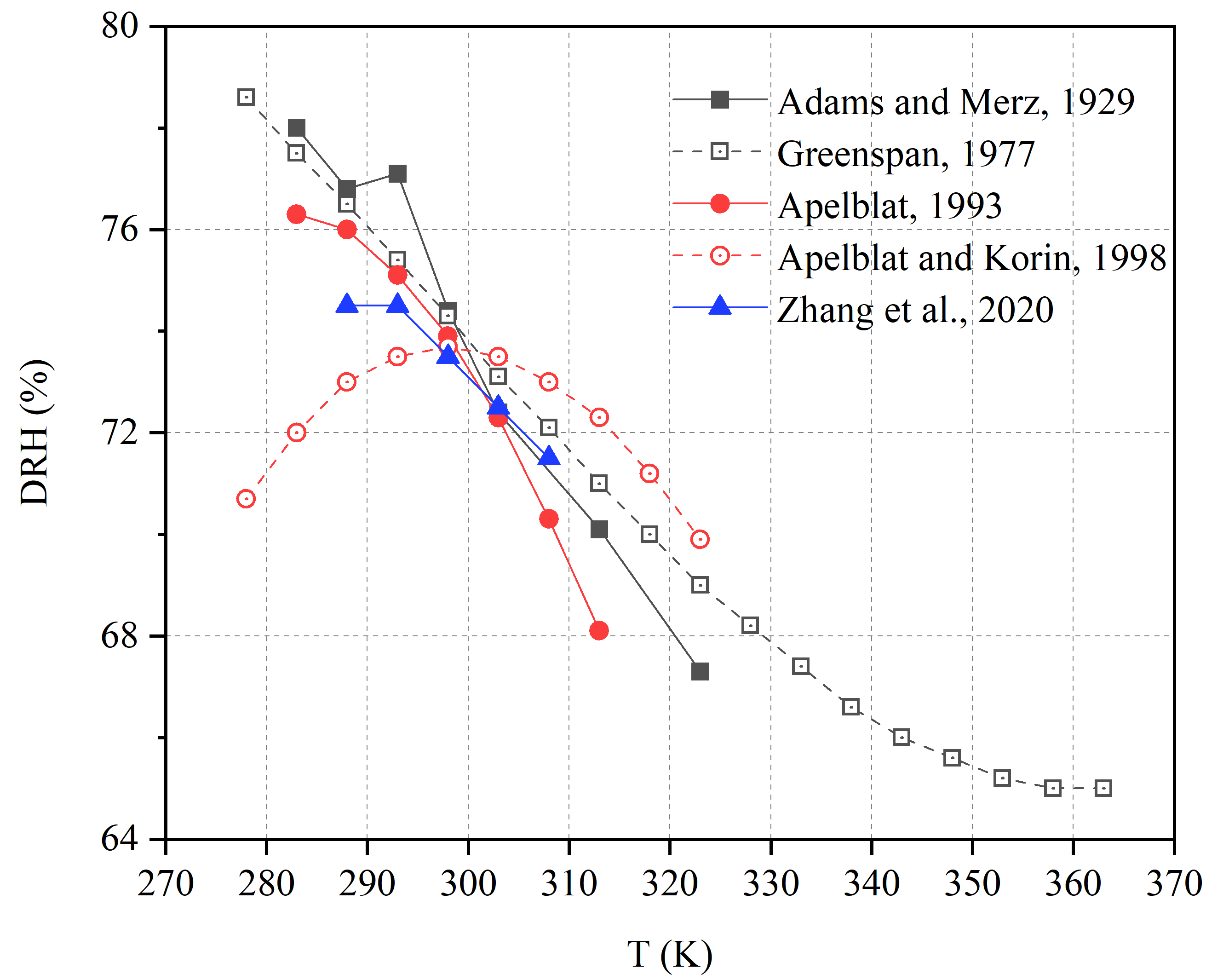


The ERH of NaNO_3_ was also measured by a number of studies (Richardson and Snyder, 1994; Tang and Munkelwitz, 1994; Lamb et al., 1996; Tang, 1996; Tang and Fung, 1997; Lee and Hsu, 2000; Gysel et al., 2002; Hoffman et al., 2004; Liu et al., 2008; Lu et al., 2008; Ghorai and Tivanski, 2010; Kim et al., 2012; Zhang et al., 2014; Gupta et al., 2015; Ji et al., 2017). Four studies (Lamb et al., 1996; Tang and Fung, 1997; Ghorai and Tivanski, 2010; Ji et al., 2017) suggested that the efflorescence of NaNO_3_ took place at 35-45% RH at 298 K, six studies (Richardson and Snyder, 1994; Lee and Hsu, 2000; Gysel et al., 2002; Hoffman et al., 2004; Liu et al., 2008; Lu et al., 2008) found that NaNO_3_ displayed continuous loss of water; in addition, the ERH of NaNO_3_ at 298 K were measured to be 0.05-30% by Tang and Munkelwitz (1994) and Tang (1996) and to be 62.5±2.5% by Zhang et al. (2014).

Kim et al. (2012) and Gupta et al. (2015) suggested that the measured ERH of NaNO_3_ was closely related to how particles were generated: the DRH was determined to be 26-47% at 296-297 K for dry deposited (or crystalline) particles, and wet dispersed (or amorphous) particles displayed continuous water loss.

**Preferred values at 298 K for NaNO_3_:**

DRH: 71-76%

ERH: 35-45%

**References:**

Adams, J. R., and Merz, A. R.: Hygroscopicity of fertilizer materials and mixtures, Industrial and Engineering Chemistry, 21, 305-307, 1929.

Apelblat, A.: The vapor-pressures of saturated aqueous lithium-chloride, sodium-bromide, sodium-nitrate, ammonium-nitrate, and ammonium-chloride at temperatures from 283 K to 313 K, Journal of Chemical Thermodynamics, 25, 63-71, 1993.

Apelblat, A., and Korin, E.: The vapour pressures of saturated aqueous solutions of sodium chloride, sodium bromide, sodium nitrate, sodium nitrite, potassium iodate, and rubidium chloride at temperatures from 227 K to 323 K, Journal of Chemical Thermodynamics, 30, 59-71, 1998.

Ghorai, S., and Tivanski, A. V.: Hygroscopic Behavior of Individual Submicrometer Particles Studied by X-ray Spectromicroscopy, Analytical Chemistry, 82, 9289-9298, 2010.

Gibson, E. R., Hudson, P. K., and Grassian, V. H.: Physicochemical properties of nitrate aerosols: Implications for the atmosphere, Journal of Physical Chemistry A, 110, 11785-11799, 2006.

Greenspan, L.: Humidity fixed-points of binary saturated aqueous-solutions, Journal of Research of the National Bureau of Standards Section a-Physics and Chemistry, 81, 89-96, 1977.

Gupta, D., Kim, H., Park, G., Li, X., Eom, H. J., and Ro, C. U.: Hygroscopic properties of NaCl and NaNO_3_ mixture particles as reacted inorganic sea-salt aerosol surrogates, Atmospheric Chemistry and Physics, 15, 3379-3393, 2015.

Gysel, M., Weingartner, E., and Baltensperger, U.: Hygroscopicity of aerosol particles at low temperatures. 2. Theoretical and experimental hygroscopic properties of laboratory generated aerosols, Environmental Science & Technology, 36, 63-68, 2002.

Hoffman, R. C., Laskin, A., and Finlayson-Pitts, B. J.: Sodium nitrate particles: physical and chemical properties during hydration and dehydration, and implications for aged sea salt aerosols, Journal of Aerosol Science, 35, 869-887, 2004.

Ji, Z.-R., Zhang, Y., Pang, S.-F., and Zhang, Y.-H.: Crystal Nucleation and Crystal Growth and Mass Transfer in Internally Mixed Sucrose/NaNO_3_ Particles, Journal of Physical Chemistry A, 121, 7968-7975, 2017.

Jing, B., Wang, Z., Tan, F., Guo, Y., Tong, S., Wang, W., Zhang, Y., and Ge, M.: Hygroscopic behavior of atmospheric aerosols containing nitrate salts and water-soluble organic acids, Atmospheric Chemistry and Physics, 18, 5115-5127, 2018.

Kim, H., Lee, M.-J., Jung, H.-J., Eom, H.-J., Maskey, S., Ahn, K.-H., and Ro, C.-U.: Hygroscopic behavior of wet dispersed and dry deposited NaNO3 particles, Atmospheric Environment, 60, 68-75, 2012.

Lamb, D., Moyle, A. M., and Brune, W. H.: The environmental control of individual aqueous particles in a cubic electrodynamic levitation system, Aerosol Science and Technology, 24, 263-278, 1996.

Lee, C.-T., and Hsu, W.-C.: The measurement of liquid water mass associated with collected hygroscopic particles, Journal of Aerosol Science, 31, 189-197, 2000.

Liu, Y., Yang, Z., Desyaterik, Y., Gassman, P. L., Wang, H., and Laskin, A.: Hygroscopic behavior of substrate-deposited particles studied by micro-FT-IR spectroscopy and complementary methods of particle analysis, Analytical Chemistry, 80, 633-642, 2008.

Lu, P.-D., Wang, F., Zhao, L.-J., Li, W.-X., Li, X.-H., Dong, J.-L., Zhang, Y.-H., and Lu, G.-Q.: Molecular events in deliquescence and efflorescence phase transitions of sodium nitrate particles studied by Fourier transform infrared attenuated total reflection spectroscopy, Journal of Chemical Physics, 129, 2008.

Ma, Q., Zhong, C., Liu, C., Liu, J., Ma, J., Wu, L., and He, H.: A Comprehensive Study about the Hygroscopic Behavior of Mixtures of Oxalic Acid and Nitrate Salts: Implication for the Occurrence of Atmospheric Metal Oxalate Complex, Acs Earth and Space Chemistry, 3, 1216-1225, 2019.

Richardson, C. B., and Snyder, T. D.: A study of heterogeneous nucleation in aqueous-solutions, Langmuir, 10, 2462-2465, 1994.

Stokes, R. H., and Robinson, R. A.: Standard Solutions for Humidity Control at 25°C., Industrial and Engineering Chemistry, 41, 2013-2013, 1949.

Tang, I. N.: Chemical and size effects of hygroscopic aerosols on light scattering coefficients, Journal of Geophysical Research-Atmospheres, 101, 19245-19250, 1996.

Tang, I. N., and Fung, K. H.: Hydration and Raman scattering studies of levitated microparticles: Ba(NO_3_)_2_, Sr(NO_3_)_2_, and Ca(NO_3_)_2_, Journal of Chemical Physics, 106, 1653-1660, 1997.

Tang, I. N., and Munkelwitz, H. R.: Composition and temperature-dependence of the deliquescence properties of hygroscopic aerosols, Atmospheric Environment Part a-General Topics, 27, 467-473, 1993.

Tang, I. N., and Munkelwitz, H. R.: Water activities, densities, and refractive indices of aqueous sulfates and sodium nitrate droplets of atmospheric importance, Journal of Geophysical Research-Atmospheres, 99, 18801-18808, 1994.

Xie, Z., Kuai, Y., Liu, J., Gui, H., Zhang, J., Dai, H., Xiao, H., Chen, D.-R., and Zhang, D.: In Situ Quantitative Observation of Hygroscopic Growth of Single Nanoparticle Aerosol by Surface Plasmon Resonance Microscopy, Analytical Chemistry, 92, 11062-11071, 2020.

Zhang, H., Gu, W., Li, Y. J., and Tang, M.: Hygroscopic properties of sodium and potassium salts as related to saline mineral dusts and sea salt aerosols, Journal of environmental sciences, 95, 65-72, 2020.

Zhang, Q.-N., Zhang, Y., Cai, C., Guo, Y.-C., Reid, J. P., and Zhang, Y.-H.: In Situ Observation on the Dynamic Process of Evaporation and Crystallization of Sodium Nitrate Droplets on a ZnSe Substrate by FTIR-ATR, Journal of Physical Chemistry A, 118, 2728-2737, 2014.

Zhang, Q.-N., Zhao, L.-J., Chen, S.-H., Guo, X., Luan, Y.-M., and Zhang, Y.-H.: Hygroscopic property of inorganic salts in atmospheric aerosols measured with physisorption analyzer, Atmospheric Environment, 247, 2021.

## KNO_3_ (potassium nitrate)

| Reference | *T* (K) | *D* | DRH (%) | ERH (%) | Techniques/Comments |
| --- | --- | --- | --- | --- | --- |
| Adams and Merz, 1929 | 283 | - | 97.0 | - | Isopiestic method |
|  | 288 |  | 95.6 |  |  |
|  | 293 |  | 92.3 |  |  |
|  | 298 |  | 92.0 |  |  |
|  | 303 |  | 90.5 |  |  |
|  | 313 |  | 87.9 |  |  |
|  | 323 |  | 85.0 |  |  |
| Stokes and Robinson, 1949 | 298 | - | 92.5 | - | Nonisopiestic method |
| Rockland, 1960 | 278 | - | 96 | - | Nonisopiestic method |
|  | 283 |  | 95 |  |  |
|  | 288 |  | 95 |  |  |
|  | 293 |  | 94 |  |  |
|  | 298 |  | 93 |  |  |
|  | 303 |  | 92 |  |  |
|  | 308 |  | 91 |  |  |
|  | 313 |  | 89 |  |  |
| Greenspan, 1977 | 273 | - | 96.3±2.9 | - | Nonisopiestic method |
|  | 278 |  | 96.3±2.1 |  |  |
|  | 283 |  | 96.0±1.4 |  |  |
|  | 288 |  | 95.4±1.0 |  |  |
|  | 293 |  | 94.6±0.7 |  |  |
|  | 298 |  | 93.6±0.6 |  |  |
|  | 303 |  | 92.3±0.6 |  |  |
|  | 308 |  | 90.8±0.8 |  |  |
|  | 313 |  | 89.0±1.2 |  |  |
|  | 318 |  | 87.0±1.8 |  |  |
|  | 323 |  | 84.8±2.5 |  |  |
| Apelblat and Korin, 1998 | 278 | - | 91.6 | - | Nonisopiestic method |
|  | 283 |  | 92.4 |  |  |
|  | 288 |  | 92.6 |  |  |
|  | 293 |  | 92.4 |  |  |
|  | 298 |  | 91.7 |  |  |
|  | 303 |  | 90.7 |  |  |
|  | 308 |  | 89.4 |  |  |
|  | 313 |  | 87.7 |  |  |
|  | 318 |  | 85.8 |  |  |
|  | 323 |  | 83.7 |  |  |
| Freney et al., 2009 | 288 | ~1 μm |  | - | ETEM |
|  |  |  | n. o. |  | atomized without heating |
|  |  |  | >90 |  | atomized with heating |
|  |  |  | 93 | - | ESEM  mechanically grinding |
| Tereshchenko, 2020 | 298 | - | 92.5±0.7 | - | Isopiestic method |
| Zhang et al., 2020 | 288 | - | 93.5±1.0 | - | VSA |
|  | 293 |  | 93.5±1.0 |  |  |
|  | 298 |  | 92.5±1.0 |  |  |
|  | 303 |  | 91.5±1.0 |  |  |
|  | 308 |  | 90.5±1.0 |  |  |

**Comments:**

The DRH of KNO_3_ was measured by eight studies (Adams and Merz, 1929; Stokes and Robinson, 1949; Rockland, 1960; Greenspan, 1977; Apelblat and Korin, 1998; Freney et al., 2009; Tereshchenko, 2020; Zhang et al., 2020). It was measured to be 92-94% at 288-298 K, showing good agreement among diferent studies. In addition, Freney et al. (2009) found that the pretreatment of atomized particles had an important effect on the measured DRH of KNO_3_: it was determined to be >90% after particles were heated overnight to remove surface and structurally absorbed water; for comparison, continuous water uptake was observed for atomized particles without heating.


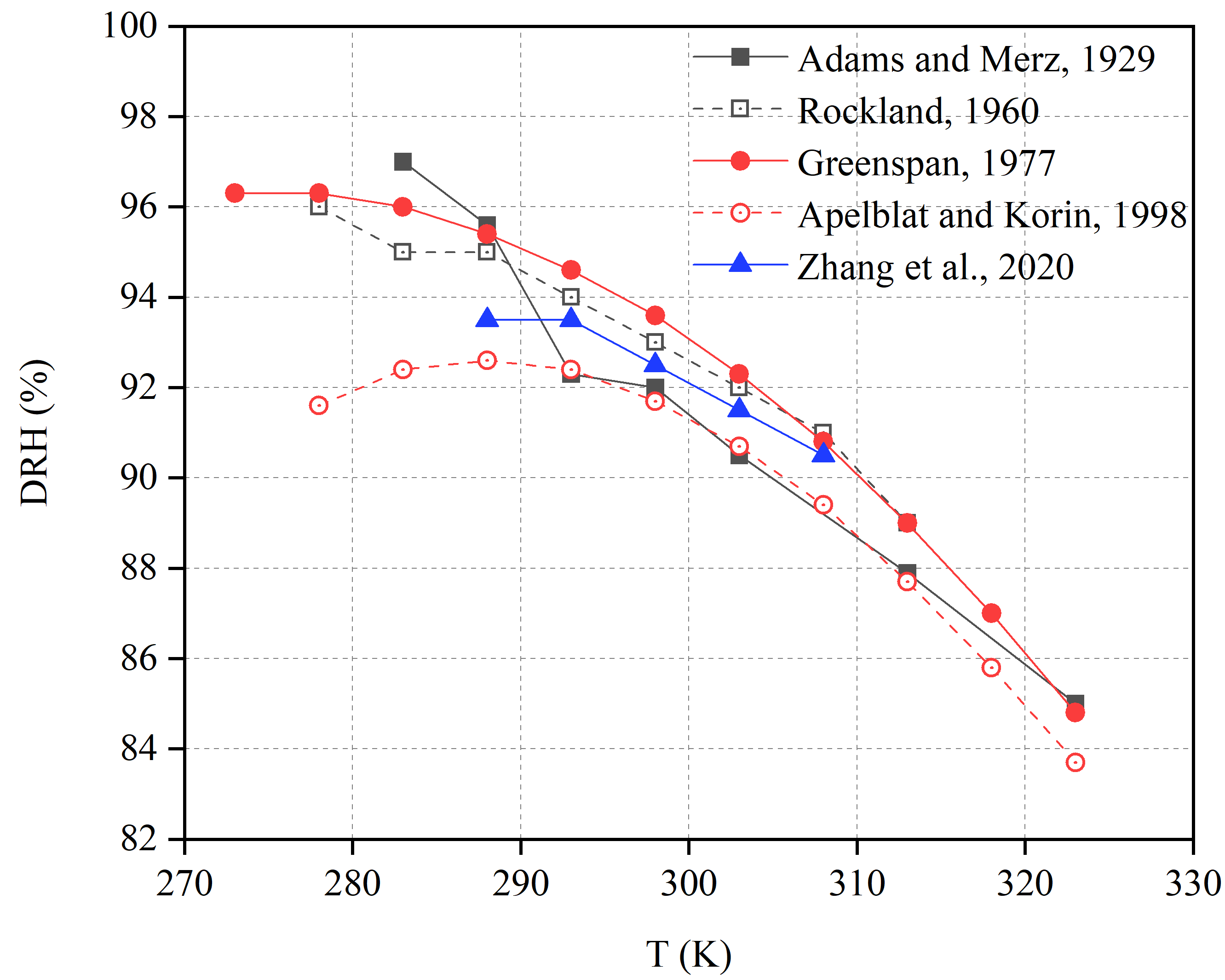


Four studies (Adams and Merz, 1929; Rockland, 1960; Greenspan, 1977; Zhang et al., 2020) measured DRH of KNO_3_ at different temperature, and revealed a negative dependence on temperature. To be more specific, Adams and Merz (1929) found that it decreased with temperature from 97.0% at 283 K to 85.0% at 323 K, Rockland (1960) found that it decreased with temperature from 96% at 278 K to 89% at 313 K, Greenspan (1977) suggested that it decreased with temperature from 96.3±2.9% at 273 K to 84.8±2.5% at 323 K, and Zhang et al. (2020) suggested that it decreased with temperature from 93.5±1.0% at 288 K to 90.5±1.0% at 308 K. Moreover, Apelblat and Korin (1998) found that the DRH of KNO_3_ first slightly increased with temperature from 91.6% at 278 K to 92.6% at 288 K, then decreased with temperature to 83.7% at 323 K.

**Preferred values at 298 K for KNO_3_:**

DRH: 92-94%

ERH: no preferred value

**References:**

Adams, J. R., and Merz, A. R.: Hygroscopicity of fertilizer materials and mixtures, Industrial and Engineering Chemistry, 21, 305-307, 1929.

Apelblat, A., and Korin, E.: Vapour pressures of saturated aqueous solutions of ammonium iodide, potassium iodide, potassium nitrate, strontium chloride, lithium sulphate, sodium thiosulphate, magnesium nitrate, and uranyl nitrate from T = (278 to 323) K, Journal of Chemical Thermodynamics, 30, 459-471, 1998.

Freney, E. J., Martin, S. T., and Buseck, P. R.: Deliquescence and Efflorescence of Potassium Salts Relevant to Biomass-Burning Aerosol Particles, Aerosol Science and Technology, 43, 799-807, 2009.

Greenspan, L.: Humidity fixed-points of binary saturated aqueous-solutions, Journal of Research of the National Bureau of Standards Section a-Physics and Chemistry, 81, 89-96, 1977.

Rockland, L. B.: Saturated Salt Solutions for Static Control of Relative Humidity between 5° and 40°C., Analytical Chemistry, 32, 1375-1376, 1960.

Stokes, R. H., and Robinson, R. A.: Standard Solutions for Humidity Control at 25°C., Industrial and Engineering Chemistry, 41, 2013-2013, 1949.

Tereshchenko, A. G.: Dynamic Method for the Determination of Hygroscopicity of Water-Soluble Solids, Journal of Solution Chemistry, 49, 1029-1051, 2020.

Zhang, H., Gu, W., Li, Y. J., and Tang, M.: Hygroscopic properties of sodium and potassium salts as related to saline mineral dusts and sea salt aerosols, Journal of environmental sciences, 95, 65-72, 2020.

## Ca(NO_3_)_2_ (calcium nitrate)

| Reference | *T* (K) | *D* | DRH (%) | ERH (%) | Techniques/Comments |
| --- | --- | --- | --- | --- | --- |
| Rockland, 1960 | 283 | - | 66 | - | Nonisopiestic method |
|  | 288 |  | 60 |  |  |
|  | 293 |  | 56 |  |  |
|  | 298 |  | 54 |  |  |
|  | 303 |  | 51 |  |  |
|  | 308 |  | 48 |  |  |
|  | 313 |  | 46 |  |  |
| Apelblat, 1992 | 283 | - | 57.2 | - | Nonisopiestic method |
|  | 288 |  | 59.6 |  |  |
|  | 293 |  | 59.1 |  |  |
|  | 298 |  | 56.0 |  |  |
|  | 303 |  | 50.8 |  |  |
|  | 308 |  | 44.2 |  |  |
| Tang and Fung, 1997 | 298 | 14-16 μm | 10-18 | n. o. | EDB |
| Al-Abadleh et al., 2003 | 295 | - | 57±5 | - | Nonisopiestic method |
|  |  |  | 7-18 | - | FTIR |
| Krueger et al., 2003 | 288 | ~5 μm | ~12 | - | ESEM |
| Laskin et al., 2005a | 298 | ~2 μm | 9-11 | - | ESEM |
| Laskin et al., 2005b | 298 | ~5 μm | 10-11 | - | ESEM |
| Gibson et al., 2006 | 298 | 100 nm | ~10 | - | HTDMA |
| Liu et al., 2008 | 298 | 5-10 μm | 7-10 | n. o. | Micro-Raman |
| Jing et al., 2018 | 298 | 100 nm | n. o. | - | HTDMA |
| Guo et al., 2019 | 298 | 100 nm | n. o. | - | HTDMA |
| Ma et al., 2019 | 298 | 300 nm | n. o. | - | HTDMA |

**Comments:**

The DRH of Ca(NO_3_)_2_ was measured by a number of studies (Rockland, 1960; Apelblat, 1992; Tang and Fung, 1997; Al-Abadleh et al., 2003; Krueger et al., 2003; Laskin et al., 2005a; Laskin et al., 2005b; Gibson et al., 2006; Liu et al., 2008; Jing et al., 2018; Guo et al., 2019; Ma et al., 2019). It was determined to be 52-62% at 295-298 K by three studies (Rockland, 1960; Apelblat, 1992; Al-Abadleh et al., 2003); it was measured to be 7-18% at 295-298 K by another seven studies (Tang and Fung, 1997; Al-Abadleh et al., 2003; Krueger et al., 2003; Laskin et al., 2005a; Laskin et al., 2005b; Gibson et al., 2006; Liu et al., 2008); in addition, three other studies (Jing et al., 2018; Guo et al., 2019; Ma et al., 2019) observed continuous water uptake by Ca(NO_3_)_2_.


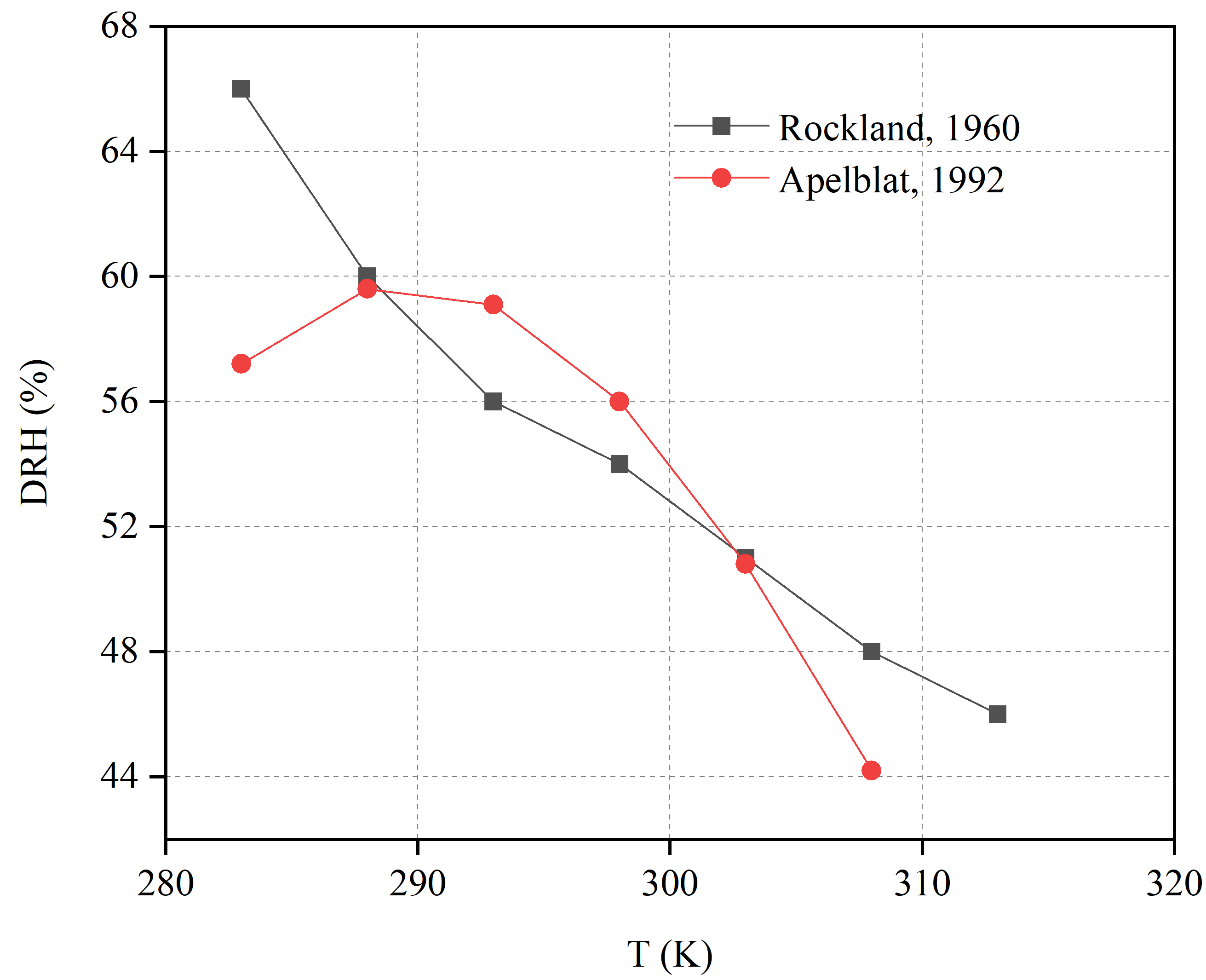


Temperature dependence of DRH of Ca(NO_3_)_2_ was investigated by two studies (Rockland, 1960; Apelblat, 1992). Rockland (1960) found that it decreased with temperature from 66% at 283 K to 46% at 313 K; similarly, Apelblat (1992) suggested that it decreased with temperature from 57.2% at 283 K to 44.2% at 308 K.

The ERH of Ca(NO_3_)_2_ was measured by two studies (Tang and Fung, 1997; Liu et al., 2008), Both studies observed gradual water evaporation with the decrease in RH, thus no ERH was reported.

**Preferred values at 298 K for Ca(NO_3_)_2_:**

DRH: 7-18% or 52-62%

ERH: no preferred value

**References:**

Al-Abadleh, H. A., Krueger, B. J., Ross, J. L., and Grassian, V. H.: Phase transitions in calcium nitrate thin films, Chemical Communications, 2796-2797, 2003.

Apelblat, A.: The vapor-pressures of water over saturated aqueous-solutions of barium-chloride, magnesium-nitrate, calcium nitrate, potassium carbonate, and zinc-sulfate at temperatures from 283 K to 313 K, Journal of Chemical Thermodynamics, 24, 619-626, 1992.

Gibson, E. R., Hudson, P. K., and Grassian, V. H.: Physicochemical properties of nitrate aerosols: Implications for the atmosphere, Journal of Physical Chemistry A, 110, 11785-11799, 2006.

Guo, L., Gu, W., Peng, C., Wang, W., Li, Y. J., Zong, T., Tang, Y., Wu, Z., Lin, Q., Ge, M., Zhang, G., Hu, M., Bi, X., Wang, X., and Tang, M.: A comprehensive study of hygroscopic properties of calcium- and magnesium-containing salts: implication for hygroscopicity of mineral dust and sea salt aerosols, Atmospheric Chemistry and Physics, 19, 2115-2133, 2019.

Jing, B., Wang, Z., Tan, F., Guo, Y., Tong, S., Wang, W., Zhang, Y., and Ge, M.: Hygroscopic behavior of atmospheric aerosols containing nitrate salts and water-soluble organic acids, Atmospheric Chemistry and Physics, 18, 5115-5127, 2018.

Krueger, B. J., Grassian, V. H., Iedema, M. J., Cowin, J. P., and Laskin, A.: Probing heterogeneous chemistry of individual atmospheric particles using scanning electron microscopy and energy-dispersive X-ray analysis, Analytical Chemistry, 75, 5170-5179, 2003.

Laskin, A., Iedema, M. J., Ichkovich, A., Graber, E. R., Taraniuk, I., and Rudich, Y.: Direct observation of completely processed calcium carbonate dust particles, Faraday Discussions, 130, 453-468, 2005a.

Laskin, A., Wietsma, T. W., Krueger, B. J., and Grassian, V. H.: Heterogeneous chemistry of individual mineral dust particles with nitric acid: A combined CCSEM/EDX, ESEM, and ICP-MS study, Journal of Geophysical Research-Atmospheres, 110, 2005b.

Liu, Y. J., Zhu, T., Zhao, D. F., and Zhang, Z. F.: Investigation of the hygroscopic properties of Ca(NO_3_)_2_ and internally mixed Ca(NO_3_)_2_/CaCO_3_ particles by micro-Raman spectrometry, Atmospheric Chemistry and Physics, 8, 7205-7215, 2008.

Ma, Q., Zhong, C., Liu, C., Liu, J., Ma, J., Wu, L., and He, H.: A Comprehensive Study about the Hygroscopic Behavior of Mixtures of Oxalic Acid and Nitrate Salts: Implication for the Occurrence of Atmospheric Metal Oxalate Complex, Acs Earth and Space Chemistry, 3, 1216-1225, 2019.

Rockland, L. B.: Saturated Salt Solutions for Static Control of Relative Humidity between 5° and 40°C., Analytical Chemistry, 32, 1375-1376, 1960.

Tang, I. N., and Fung, K. H.: Hydration and Raman scattering studies of levitated microparticles: Ba(NO_3_)_2_, Sr(NO_3_)_2_, and Ca(NO_3_)_2_, Journal of Chemical Physics, 106, 1653-1660, 1997.

## Ca(NO_3_)_2_∙4H_2_O (calcium nitrate tetrahydrate)

| Reference | *T* (K) | *D* | DRH (%) | ERH (%) | Techniques/Comments |
| --- | --- | --- | --- | --- | --- |
| Adams and Merz, 1929 | 288 | - | 55.9 | - | Isopiestic method |
|  | 293 |  | 55.4 |  |  |
|  | 298 |  | 50.5 |  |  |
|  | 303 |  | 46.7 |  |  |
|  | 313 |  | 35.5 |  |  |
| Schuttlefield et al., 2007 | 298 | - | 54±2 | - | ATR-FTIR |
| Guo et al., 2019 | 278 | - | 60.5±1.0 | - | VSA |
|  | 283 |  | 58.0±1.0 |  |  |
|  | 288 |  | 55.5±1.0 |  |  |
|  | 293 |  | 52.5±1.0 |  |  |
|  | 298 |  | 49.5±1.0 |  |  |
|  | 303 |  | 46.0±1.0 |  |  |
| Chao et al., 2020 | 298 | - | 52±3 | - | QCM |

**Comments:**

Four studies (Adams and Merz, 1929; Schuttlefield et al., 2007; Guo et al., 2019; Chao et al., 2020) measured the DRH of Ca(NO_3_)_2_∙4H_2_O. It was determined to be 49-56% at 298 K, suggesting good agreement.


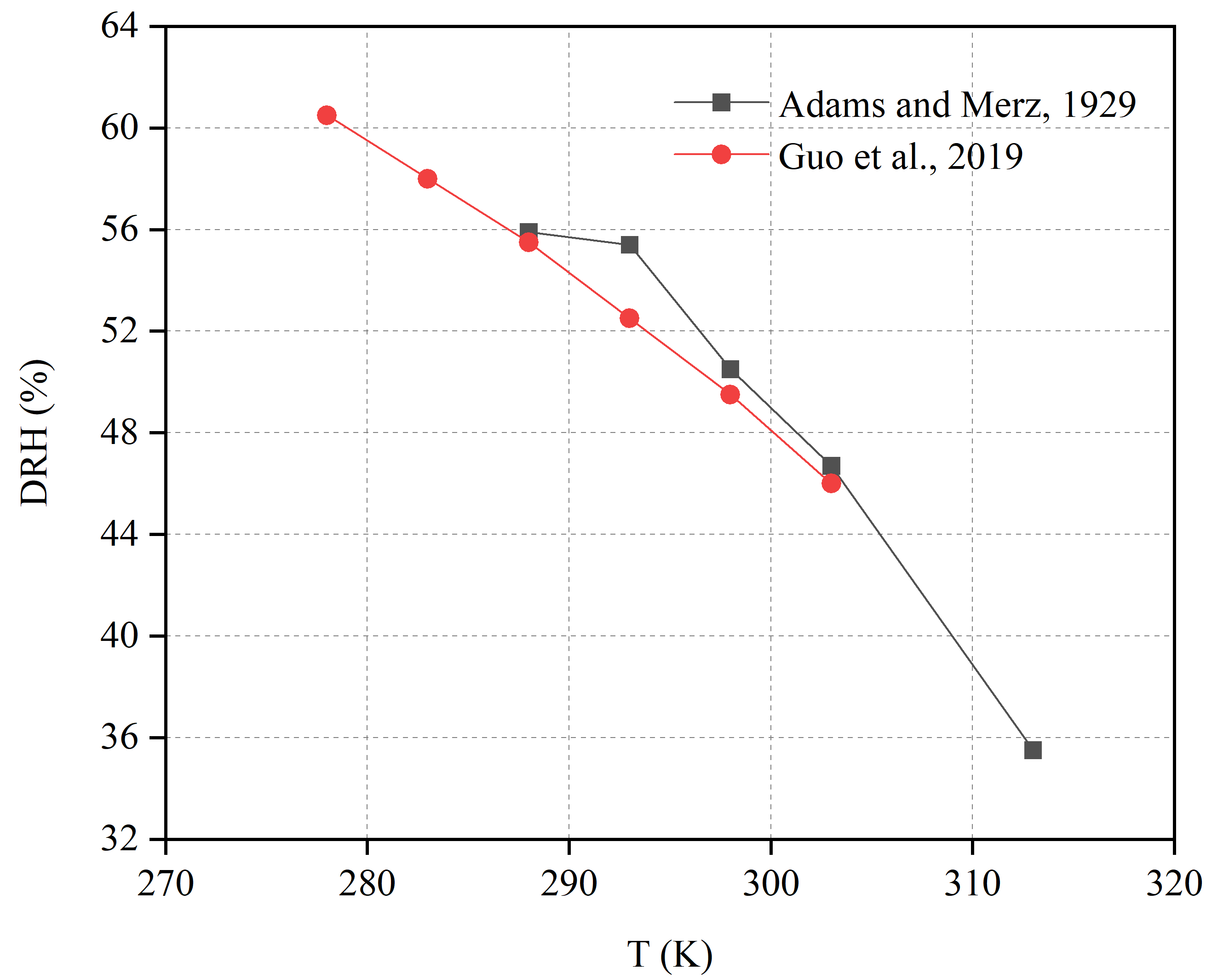


Two studies (Adams and Merz, 1929; Guo et al., 2019) investigated the DRH of Ca(NO_3_)_2_∙4H_2_O at different temperatures, and revealed similar temperature dependence: Adams and Merz (1929) found that it decreased with temperature from 55.9% at 288 K to 35.5% at 313 K; similarly, Guo et al. (2019) suggested that it decreased with temperature from 60.5±1.0 at 278 K to 46.0±1.0% at 303 K.

**Preferred values at 298 K for Ca(NO_3_)_2_∙4H_2_O:**

DRH: 49-56%

ERH: no preferred value

**References:**

Chao, H.-J., Huang, W.-C., Chen, C.-L., Chou, C. C. K., and Hung, H.-M.: Water Adsorption vs Phase Transition of Aerosols Monitored by a Quartz Crystal Microbalance, Acs Omega, 5, 31858-31866, 2020.

Guo, L., Gu, W., Peng, C., Wang, W., Li, Y. J., Zong, T., Tang, Y., Wu, Z., Lin, Q., Ge, M., Zhang, G., Hu, M., Bi, X., Wang, X., and Tang, M.: A comprehensive study of hygroscopic properties of calcium- and magnesium-containing salts: implication for hygroscopicity of mineral dust and sea salt aerosols, Atmospheric Chemistry and Physics, 19, 2115-2133, 2019.

Schuttlefield, J., Al-Hosney, H., Zachariah, A., and Grassian, V. H.: Attenuated Total Reflection Fourier Transform Infrared Spectroscopy to Investigate Water Uptake and Phase Transitions in Atmospherically Relevant Particles, Applied Spectroscopy, 61, 283-292, 2007.

## Mg(NO_3_)_2_ (magnesium nitrate)

| Reference | *T* (K) | *D* | DRH (%) | ERH (%) | Techniques/Comments |
| --- | --- | --- | --- | --- | --- |
| Rockland, 1960 | 278 | - | 54 | - | Nonisopiestic method |
|  | 283 |  | 53 |  |  |
|  | 288 |  | 53 |  |  |
|  | 293 |  | 52 |  |  |
|  | 298 |  | 52 |  |  |
|  | 303 |  | 52 |  |  |
|  | 308 |  | 51 |  |  |
|  | 313 |  | 51 |  |  |
| Greenspan, 1977 | 273 | - | 60.4±0.6 | - | Nonisopiestic method |
|  | 278 |  | 58.9±0.4 |  |  |
|  | 283 |  | 57.4±0.3 |  |  |
|  | 288 |  | 55.9±0.3 |  |  |
|  | 293 |  | 54.4±0.2 |  |  |
|  | 298 |  | 52.9±0.2 |  |  |
|  | 303 |  | 51.4±0.2 |  |  |
|  | 308 |  | 49.9±0.3 |  |  |
|  | 313 |  | 48.4±0.4 |  |  |
|  | 318 |  | 46.9±0.5 |  |  |
|  | 323 |  | 45.4±0.6 |  |  |
| Apelblat, 1992 | 283 | - | 59.2 | - | Nonisopiestic method |
|  | 288 |  | 58.6 |  |  |
|  | 293 |  | 57.3 |  |  |
|  | 298 |  | 55.2 |  |  |
|  | 303 |  | 52.6 |  |  |
|  | 308 |  | 49.5 |  |  |
|  | 313 |  | 46.0 |  |  |
| Apelblat and Korin, 1998 | 278 | - | 53.1 | - | Nonisopiestic method |
|  | 283 |  | 54.0 |  |  |
|  | 288 |  | 54.6 |  |  |
|  | 293 |  | 54.7 |  |  |
|  | 298 |  | 54.6 |  |  |
|  | 303 |  | 54.1 |  |  |
|  | 308 |  | 53.3 |  |  |
|  | 313 |  | 52.3 |  |  |
|  | 318 |  | 51.1 |  |  |
|  | 323 |  | 49.6 |  |  |
| Gibson et al., 2006 | 298 | 100 nm | n. o. | - | HTDMA |
| Guo et al., 2019 | 298 | 100 nm | n. o. | - | HTDMA |

**Comments:**

The DRH of Mg(NO_3_)_2_ was measured by six studies (Rockland, 1960; Greenspan, 1977; Apelblat, 1992; Apelblat and Korin, 1998; Gibson et al., 2006; Guo et al., 2019). It was measured to be 52-55% at 298 K by four studies (Rockland, 1960; Greenspan, 1977; Apelblat, 1992; Apelblat and Korin, 1998), showing good agreement. Moreover, continuous hygroscopic growth was observed by the other two studies (Gibson et al., 2006; Guo et al., 2019), thus no DRH was reported.


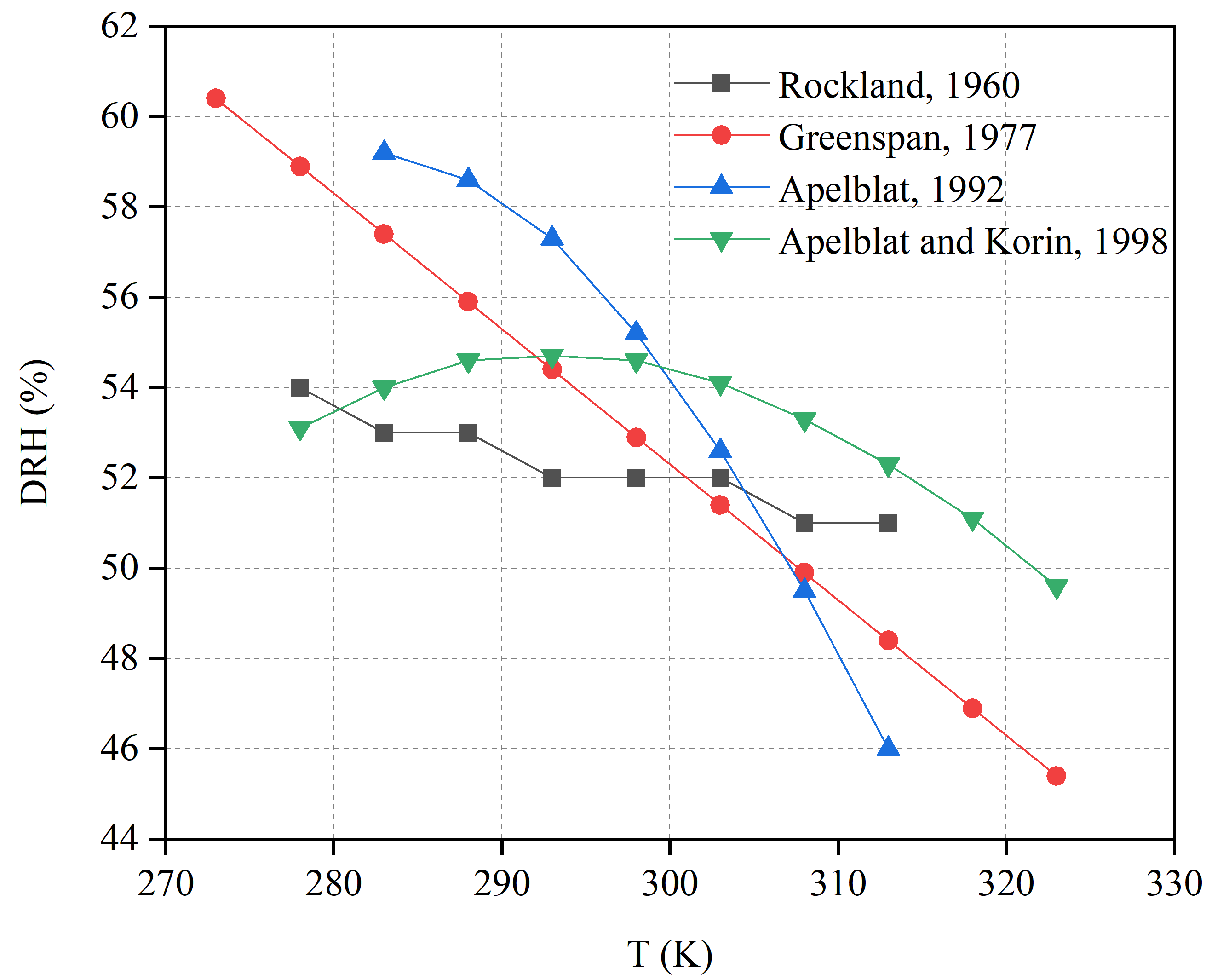


Three studies (Rockland, 1960; Greenspan, 1977; Apelblat, 1992) revealed similar temperature dependence of DRH of Mg(NO_3_)_2_: Rockland (1960) found that it slightly decreased with temperature from 54% at 278 K to 51% at 313 K, Greenspan (1977) found that it decreased with temperature from 60.4±0.6% at 273 K to 45.4±0.6% at 323 K, and Apelblat (1992) suggested that it decreased with temperature from 59.2% at 283 K to 46.0% at 313 K. Moreover, Apelblat and Korin (1998) found that the DRH of Mg(NO_3_)_2_ first slightly increased with temperature from 53.1% at 278 K to 54.7% at 293 K, then decreased with temperature to 49.6% at 323 K.

**Preferred values at 298 K for Mg(NO_3_)_2_:**

DRH: 52-55%

ERH: no preferred value

**References:**

Apelblat, A.: The vapor-pressures of water over saturated aqueous-solutions of barium-chloride, magnesium-nitrate, calcium nitrate, potassium carbonate, and zinc-sulfate at temperatures from 283 K to 313 K, Journal of Chemical Thermodynamics, 24, 619-626, 1992.

Apelblat, A., and Korin, E.: Vapour pressures of saturated aqueous solutions of ammonium iodide, potassium iodide, potassium nitrate, strontium chloride, lithium sulphate, sodium thiosulphate, magnesium nitrate, and uranyl nitrate from T = (278 to 323) K, Journal of Chemical Thermodynamics, 30, 459-471, 1998.

Gibson, E. R., Hudson, P. K., and Grassian, V. H.: Physicochemical properties of nitrate aerosols: Implications for the atmosphere, Journal of Physical Chemistry A, 110, 11785-11799, 2006.

Greenspan, L.: Humidity fixed-points of binary saturated aqueous-solutions, Journal of Research of the National Bureau of Standards Section a-Physics and Chemistry, 81, 89-96, 1977.

Guo, L., Gu, W., Peng, C., Wang, W., Li, Y. J., Zong, T., Tang, Y., Wu, Z., Lin, Q., Ge, M., Zhang, G., Hu, M., Bi, X., Wang, X., and Tang, M.: A comprehensive study of hygroscopic properties of calcium- and magnesium-containing salts: implication for hygroscopicity of mineral dust and sea salt aerosols, Atmospheric Chemistry and Physics, 19, 2115-2133, 2019.

Rockland, L. B.: Saturated Salt Solutions for Static Control of Relative Humidity between 5° and 40°C., Analytical Chemistry, 32, 1375-1376, 1960.

## Mg(NO_3_)_2_∙6H_2_O (magnesium nitrate hexahydrate)

| Reference | *T* (K) | *D* | DRH (%) | ERH (%) | Techniques/Comments |
| --- | --- | --- | --- | --- | --- |
| Stokes and Robinson, 1949 | 298 | - | 52.9 | - | Nonisopiestic method |
| Al-Abadleh and Grassian, 2003 | 296 | - | 49-54 | - | FTIR |
| Li et al., 2008 | 296 | - | ~52 | n. o. (<3) | ATR-FTIR |
| Gu et al., 2017 | 278 | - | 58.2±1.0 | - | VSA |
|  | 283 |  | 57.1±1.0 |  |  |
|  | 288 |  | 55.6±1.0 |  |  |
|  | 293 |  | 54.4±1.0 |  |  |
|  | 298 |  | 53.1±1.0 |  |  |
|  | 303 |  | 51.8±1.0 |  |  |
| Guo et al., 2019 | 278 | - | 57.5±1.0 | - | VSA |
|  | 283 |  | 56.5±1.0 |  |  |
|  | 288 |  | 54.5±1.0 |  |  |
|  | 293 |  | 53.5±1.0 |  |  |
|  | 298 |  | 52.5±1.0 |  |  |
|  | 303 |  | 50.5±1.0 |  |  |

**Comments:**

Five studies (Stokes and Robinson, 1949; Al-Abadleh and Grassian, 2003; Li et al., 2008; Gu et al., 2017; Guo et al., 2019) measured the DRH of Mg(NO_3_)_2_∙6H_2_O. The measured DRH showed good agreement among these studies, being 49-54% at 296-298 K.

In addition, the DRH of Mg(NO_3_)_2_∙6H_2_O was measured at different temperature by two studies (Gu et al., 2017; Guo et al., 2019). Gu et al. (2017) found that it decreased with temperature from 58.2±1.0% at 278 K to 51.8±1.0% at 303 K; similarly, Guo et al. (2019) suggested that it decreased with temperature from 57.5±1.0% at 278 K to 50.5±1.0% at 303 K.


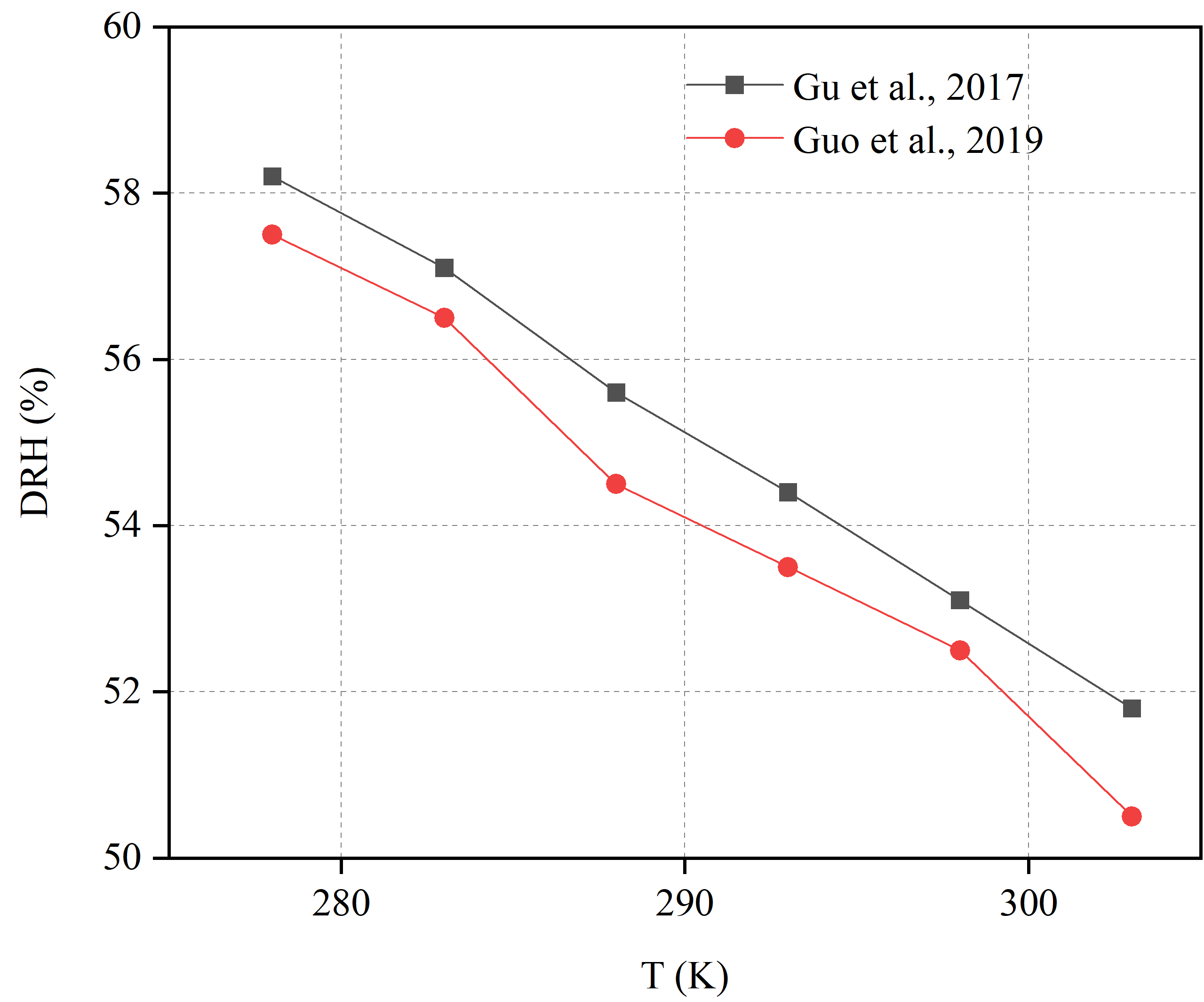


Continuous loss of water was observed with the decrease in RH for Mg(NO_3_)_2_∙6H_2_O (Li et al., 2008), and thus no ERH was reported in the study.

**Preferred values at 298 K for Mg(NO_3_)_2_∙6H_2_O:**

DRH: 49-54%

ERH: no preferred value

**References:**

Al-Abadleh, H. A., and Grassian, V. H.: Phase transitions in magnesium nitrate thin films: A transmission FT-IR study of the deliquescence and efflorescence of nitric acid reacted magnesium oxide interfaces, Journal of Physical Chemistry B, 107, 10829-10839, 2003.

Gu, W., Li, Y., Zhu, J., Jia, X., Lin, Q., Zhang, G., Ding, X., Song, W., Bi, X., Wang, X., and Tang, M.: Investigation of water adsorption and hygroscopicity of atmospherically relevant particles using a commercial vapor sorption analyzer, Atmospheric Measurement Techniques, 10, 3821-3832, 2017.

Guo, L., Gu, W., Peng, C., Wang, W., Li, Y. J., Zong, T., Tang, Y., Wu, Z., Lin, Q., Ge, M., Zhang, G., Hu, M., Bi, X., Wang, X., and Tang, M.: A comprehensive study of hygroscopic properties of calcium- and magnesium-containing salts: implication for hygroscopicity of mineral dust and sea salt aerosols, Atmospheric Chemistry and Physics, 19, 2115-2133, 2019.

Li, X. H., Dong, J. L., Xiao, H. S., Lu, P. D., Hu, Y. A., and Zhang, Y. H.: FTIR-ATR in situ observation on the efflorescence and deliquescence processes of Mg(NO_3_)_2_ aerosols, Sci. China Ser. B-Chem., 51, 128-137, 2008.

Stokes, R. H., and Robinson, R. A.: Standard Solutions for Humidity Control at 25°C., Industrial and Engineering Chemistry, 41, 2013-2013, 1949.

# Fluorides

## NaF (sodium fluoride)

| Reference | *T* (K) | *D* | DRH (%) | ERH (%) | Techniques/Comments |
| --- | --- | --- | --- | --- | --- |
| Shpunt, 1968 | 373 | - | 96.6 | - | Nonisopiestic method |
| Tang et al., 1977 | 298 | 0.3-2 μm | 96.7 | - | H-DMA-OPC |
| Krieger and Braun, 2001 | 278 | 5 μm | 95-96 | - | EDB |
| Miñambres et al., 2011 | 298 | 0.63 μm | 85-90 | - | FTIR |
| Gupta et al., 2014 | 295 | 1-10 μm | 94.3 | 69.1-77.2 | Optical microscopy |

**Comments:**

Three studies (Tang et al., 1977; Miñambres et al., 2011; Gupta et al., 2014) measured DRH of NaF at around room temperature. It was determined to be 96.7% at 298 K by Tang et al. (1977), in good agreement with that (94.3% at 295 K) reported by Gupta et al. (2014) and slightly higher than that (85-90%at 298 K) reported by Miñambres et al. (2011). In addition, the DRH values were measured to be 95-96% at 278 K (Krieger and Braun , 2001) and 96.6% at 373 K (Shpunt, 1968), showing no significance difference with that at ~298 K.

The ERH of NaF was only measured by one study (Gupta et al., 2014), and it was determined to be 69.1-77.2% at 295 K.

**Preferred values at 298 K for NaF:**

DRH: 94-97%

ERH: 69-77%

**References:**

Gupta, D., Eom, H.-J., Li, X., Cho, H.-R., Park, G.-H., Lee, J.-S., Kim, H.-K., and Ro, C.-U.: Hygroscopic properties of pure and mixed halide particles as nascent sea salt aerosol surrogates, The 8th International Conference on Combustion, Incineration/Pyrolysis, Emission and Climate Change (i-CIPEC), Hangzhou, China, October 15-18, 2014.

Krieger, U. K., and Braun, C.: Light-scattering intensity fluctuations in single aerosol particles during deliquescence, Journal of Quantitative Spectroscopy & Radiative Transfer, 70, 545-554, 2001.

Minambres, L., Mendez, E., Sanchez, M. N., Castano, F., and Basterretxea, F. J.: Water uptake properties of internally mixed sodium halide and succinic acid particles, Atmospheric Environment, 45, 5896-5902, 2011.

Shpunt, A. A.: Hygroresistance (Hygroscopicity) of crystals, Measurement Techniques-Ussr, 1698-1700, 1968.

Tang, I. N., Munkelwitz, H. R., and Davis, J. G.: Aerosol growth studies—II. Preparation and growth measurements of monodisperse salt aerosols, Journal of aerosol science, 8, 149-159, 1977.

## KF (potassium fluoride) and KF∙2H_2_O (potassium fluoride dihydrate)

| Species | Reference | *T* (K) | *D* | DRH (%) | ERH (%) | Techniques/Comments |
| --- | --- | --- | --- | --- | --- | --- |
| KF | Greenspan, 1977 | 298 | - | 30.9±1.3 | - | Nonisopiestic method |
|  |  | 303 |  | 27.3±1.1 |  |  |
|  |  | 308 |  | 24.6±0.9 |  |  |
|  |  | 313 |  | 22.7±0.8 |  |  |
|  |  | 318 |  | 21.5±0.7 |  |  |
|  |  | 323 |  | 20.8±0.6 |  |  |
|  |  | 328 |  | 20.6±0.6 |  |  |
|  |  | 333 |  | 20.8±0.5 |  |  |
|  |  | 338 |  | 21.2±0.5 |  |  |
|  |  | 343 |  | 21.7±0.6 |  |  |
|  |  | 348 |  | 22.3±0.6 |  |  |
|  |  | 353 |  | 22.9±0.7 |  |  |
|  |  | 358 |  | 23.2±0.8 |  |  |
|  |  | 363 |  | 23.3±0.9 |  |  |
|  | Tang et al., 1977 | 298 | 0.3-2 μm | 30.3 | - | H-DMA-OPC |
|  | Richardson and Snyder, 1994 | 298 | 10 μm | 17.7 | 9.0±0.1 | EDB |
|  | Gupta et al., 2014 | 295 | 1-10 μm | 18.5-19.2 | 12.9-13.3 | Optical microscopy |
| KF∙2H_2_O | Shpunt, 1968 | 291 | - | 34 | - | Nonisopiestic method |

**Comments:**

Greenspan (1977) used the nonisopiestic method to measure DRH of KF, which decreased with temperature from 30.9±1.3% at 298 K to 20.6±0.6% at 328 K, and then increased with temperature to 23.3±0.9% at 363 K. The DRH of KF at 298 K was measured to be 30.3% reported by Tang et al. (1977), agreed well with that reported by Greenspan (1977). In addition, the DRH values at 295-298 K were reported to be 17.7% and 18.5-19.2% by the other two studies (Richardson and Snyder, 1994; Gupta et al., 2014), significantly lower than those reported by the first two studies (Greenspan, 1977; Tang et al., 1977).

The ERH values of KF were measured to be 9.0±0.1% at 298 K by Richardson and Snyder (1994) and 12.9-13.3% at 295 K by Gupta et al. (2014), showing moderate agreement.

The DRH of KF∙2H_2_O was measured to be 34% at 291 K (Shpunt, 1968), using the nonisopiestic method.

**Preferred values at 298 K for KF:**

DRH: 30-32%

ERH: 8-13%

**Preferred values at 298 K for KF∙2H_2_O:**

DRH: 34%

ERH: no preferred value

**References:**

Greenspan, L.: Humidity fixed-points of binary saturated aqueous-solutions, Journal of Research of the National Bureau of Standards Section a-Physics and Chemistry, 81, 89-96, 1977.

Gupta, D., Eom, H.-J., Li, X., Cho, H.-R., Park, G.-H., Lee, J.-S., Kim, H.-K., and Ro, C.-U.: Hygroscopic properties of pure and mixed halide particles as nascent sea salt aerosol surrogates, The 8th International Conference on Combustion, Incineration/Pyrolysis, Emission and Climate Change (i-CIPEC), Hangzhou, China, October 15-18, 2014.

Richardson, C. B., and Snyder, T. D.: A study of heterogeneous nucleation in aqueous-solutions, Langmuir, 10, 2462-2465, 1994.

Shpunt, A. A.: Hygroresistance (Hygroscopicity) of crystals, Measurement Techniques-Ussr, 1698-1700, 1968.

Tang, I. N., Munkelwitz, H. R., and Davis, J. G.: Aerosol growth studies—II. Preparation and growth measurements of monodisperse salt aerosols, Journal of aerosol science, 8, 149-159, 1977.

# Chlorides

## NH_4_Cl (ammonium chloride)

| Reference | *T* (K) | *D* | DRH (%) | ERH (%) | Techniques/Comments |
| --- | --- | --- | --- | --- | --- |
| Adams and Merz, 1929 | 283 | - | 79.5 | - | Isopiestic method |
|  | 288 |  | 79.2 |  |  |
|  | 293 |  | 79.3 |  |  |
|  | 298 |  | 76.0 |  |  |
|  | 303 |  | 77.2 |  |  |
|  | 313 |  | 73.7 |  |  |
|  | 323 |  | 71.3 |  |  |
| Pearce and Pumplin, 1937 | 298 | - | 77.0 | - | Nonisopiestic method |
| Greenspan, 1977 | 283 | - | 80.6±1.0 | - | Nonisopiestic method |
|  | 288 |  | 79.9±0.6 |  |  |
|  | 293 |  | 79.2±0.4 |  |  |
|  | 298 |  | 78.6±0.4 |  |  |
|  | 303 |  | 77.9±0.6 |  |  |
| Cohen et al., 1987 | 293 | 20 μm | 76.5-77.3 | 45 | EDB |
| Apelblat, 1993 | 283 | - | 79.1 | - | Nonisopiestic method |
|  | 288 |  | 77.4 |  |  |
|  | 293 |  | 77.9 |  |  |
|  | 298 |  | 78.1 |  |  |
|  | 303 |  | 77.4 |  |  |
|  | 308 |  | 75.5 |  |  |
|  | 313 |  | 72.6 |  |  |
| Lee and Chang, 2002 | 298 | 0.6-1.3 μm | 78 | 45 | Katharometer |
| Hu et al., 2011 | 298 | 40-200 nm | 78 | - | HTDMA |

**Comments:**

The DRH of NH_4_Cl was measured by several studies (Adams and Merz, 1929; Pearce and Pumplin, 1937; Greenspan, 1977; Cohen et al., 1987; Apelblat, 1993; Lee and Chang, 2002; Hu et al., 2011), and their measured DRH showed good agreement, being in the range of 76-79% at 293-298 K.


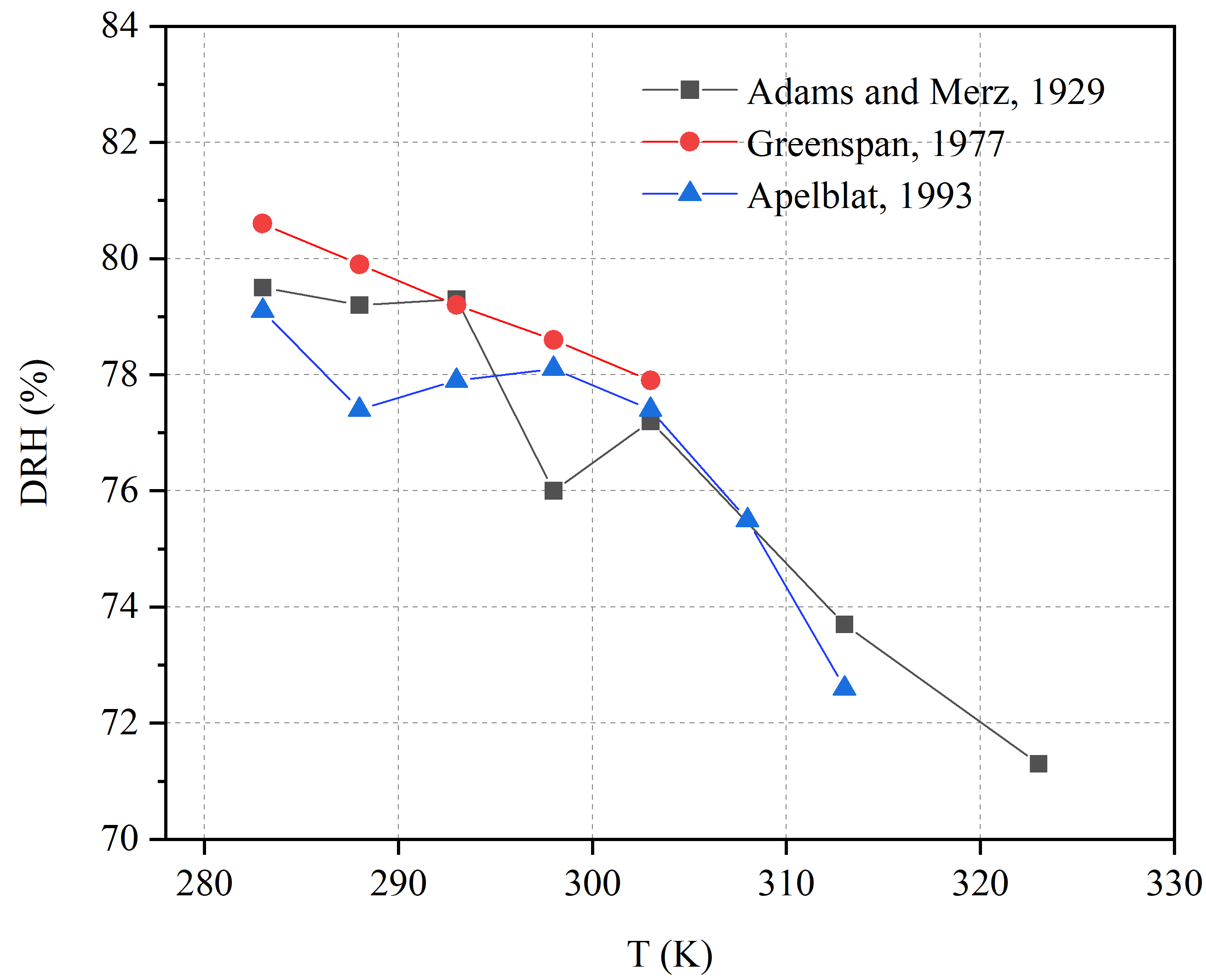


In addition, three studies (Adams and Merz, 1929; Greenspan, 1977; Apelblat, 1993) measured the DRH of NH_4_Cl at different RH, showing similar dependence on temperature: Adams and Merz (1929) found that the DRH of NH_4_Cl decreased with temperature from 79.5% at 283 K to 71.3% at 323 K, Greenspan (1977) found that it decreased with temperature from 80.6% at 283 K to 77.9% at 303 K, and Apelblat (1993) found that it decreased with temperature from 79.1% at 283 K to 72.6% at 313 K.

The ERH of of NH_4_Cl was only measured by two studies (Cohen et al., 1987; Lee and Chang, 2002), and their measured ERH showed good agreement, being 45% at 293-298 K.

**Preferred values at 298 K for NH_4_Cl:**

DRH: 76-79%

ERH: 45%

**References:**

Adams, J. R., and Merz, A. R.: Hygroscopicity of fertilizer materials and mixtures, Industrial and Engineering Chemistry, 21, 305-307, 1929.

Apelblat, A.: The vapor-pressures of saturated aqueous lithium-chloride, sodium-bromide, sodium-nitrate, ammonium-nitrate, and ammonium-chloride at temperatures from 283 K to 313 K, Journal of Chemical Thermodynamics, 25, 63-71, 1993.

Cohen, M. D., Flagan, R. C., and Seinfeld, J. H.: Studies of concentrated electrolyte-solutions using the electrodynamic balance .1. Water activities for single-electrolyte solutions, Journal of Physical Chemistry, 91, 4563-4574, 1987.

Cohen, M. D., Flagan, R. C., and Seinfeld, J. H.: Studies of concentrated electrolyte solutions using the electrodynamic balance. 3. Solute nucleation, Journal of Physical Chemistry, 91, 4583-4590, 1987.

Greenspan, L.: Humidity fixed-points of binary saturated aqueous-solutions, Journal of Research of the National Bureau of Standards Section a-Physics and Chemistry, 81, 89-96, 1977.

Hu, D., Chen, J., Ye, X., Li, L., and Yang, X.: Hygroscopicity and evaporation of ammonium chloride and ammonium nitrate: Relative humidity and size effects on the growth factor, Atmospheric Environment, 45, 2349-2355, 2011.

Lee, C. T., and Chang, S. Y.: A GC-TCD method for measuring the liquid water mass of collected aerosols, Atmospheric Environment, 36, 1883-1894, 2002.

Pearce, J. N., and Pumplin, G. G.: The vapor pressures and activity coefficients of aqueous solutions of ammonium chloride at 25°C, Journal of the American Chemical Society, 59, 1219-1220, 1937.

## LiCl (lithium chloride) and LiCl∙H_2_O (lithium chloride monohydrate)

| Species | Reference | *T* (K) | *D* | DRH (%) | ERH (%) | Techniques/Comments |
| --- | --- | --- | --- | --- | --- | --- |
| LiCl | Pearce and Nelson, 1932 | 298 | - | 11.7 | - | Nonisopiestic method |
|  | Rockland, 1960 | 278 | - | 16 | - | Nonisopiestic method |
|  |  | 283 |  | 14 |  |  |
|  |  | 288 |  | 13 |  |  |
|  |  | 293 |  | 12 |  |  |
|  |  | 298 |  | 11 |  |  |
|  |  | 303 |  | 11 |  |  |
|  |  | 308 |  | 11 |  |  |
|  |  | 313 |  | 11 |  |  |
|  | Greenspan, 1977 | 273 | - | 11.2±0.5 | - | Nonisopiestic method |
|  |  | 278 |  | 11.3±0.5 |  |  |
|  |  | 283 |  | 11.3±0.4 |  |  |
|  |  | 288 |  | 11.3±0.4 |  |  |
|  |  | 293 |  | 11.3±0.3 |  |  |
|  |  | 298 |  | 11.3±0.3 |  |  |
|  |  | 303 |  | 11.3±0.2 |  |  |
|  |  | 308 |  | 11.3±0.2 |  |  |
|  |  | 313 |  | 11.2±0.2 |  |  |
|  |  | 318 |  | 11.2±0.2 |  |  |
|  |  | 323 |  | 11.1±0.2 |  |  |
|  |  | 328 |  | 11.0±0.2 |  |  |
|  |  | 333 |  | 11.0±0.3 |  |  |
|  |  | 338 |  | 10.9±0.3 |  |  |
|  |  | 343 |  | 10.8±0.3 |  |  |
|  |  | 348 |  | 10.6±0.4 |  |  |
|  |  | 353 |  | 10.5±0.4 |  |  |
|  |  | 358 |  | 10.4±0.5 |  |  |
|  |  | 363 |  | 10.2±0.6 |  |  |
|  |  | 368 |  | 10.1±0.7 |  |  |
|  |  | 373 |  | 9.9±0.8 |  |  |
|  | Tang et al., 1977 | 298 | 0.3-2 μm | 11.2 | - | H-DMA-OPC |
|  | Apelblat, 1993 | 283 | - | 10.4 | - | Nonisopiestic method |
|  |  | 288 |  | 11.3 |  |  |
|  |  | 293 |  | 11.9 |  |  |
|  |  | 298 |  | 12.1 |  |  |
|  |  | 303 |  | 12.0 |  |  |
|  |  | 308 |  | 11.5 |  |  |
|  |  | 313 |  | 10.7 |  |  |
|  | Arenas et al., 2012 | 298 | - | 10.3±1.1 | - | QCM |
|  | Beyer et al., 2014 | 277 | - | 12.9±1 | - | VSA |
|  |  | 298 | - | 10.2±1 |  |  |
|  | Schindelholz et al., 2014 | 294 | ~100 μm | 10±1.5 | 1-4 | Electrical conductivity/ impedance method |
| LiCl∙H_2_O | Stokes and Robinson, 1949 | 298 | - | 11.1 | - | Nonisopiestic method |

**Comments:**

The DRH of LiCl was measured by several studies (Pearce and Nelson, 1932; Rockland, 1960; Greenspan, 1977; Tang et al., 1977; Apelblat, 1993; Arenas et al., 2012; Beyer et al., 2014; Schindelholz et al., 2014), and their measured DRH showed good agreement, being 9-12% at 294-298 K.


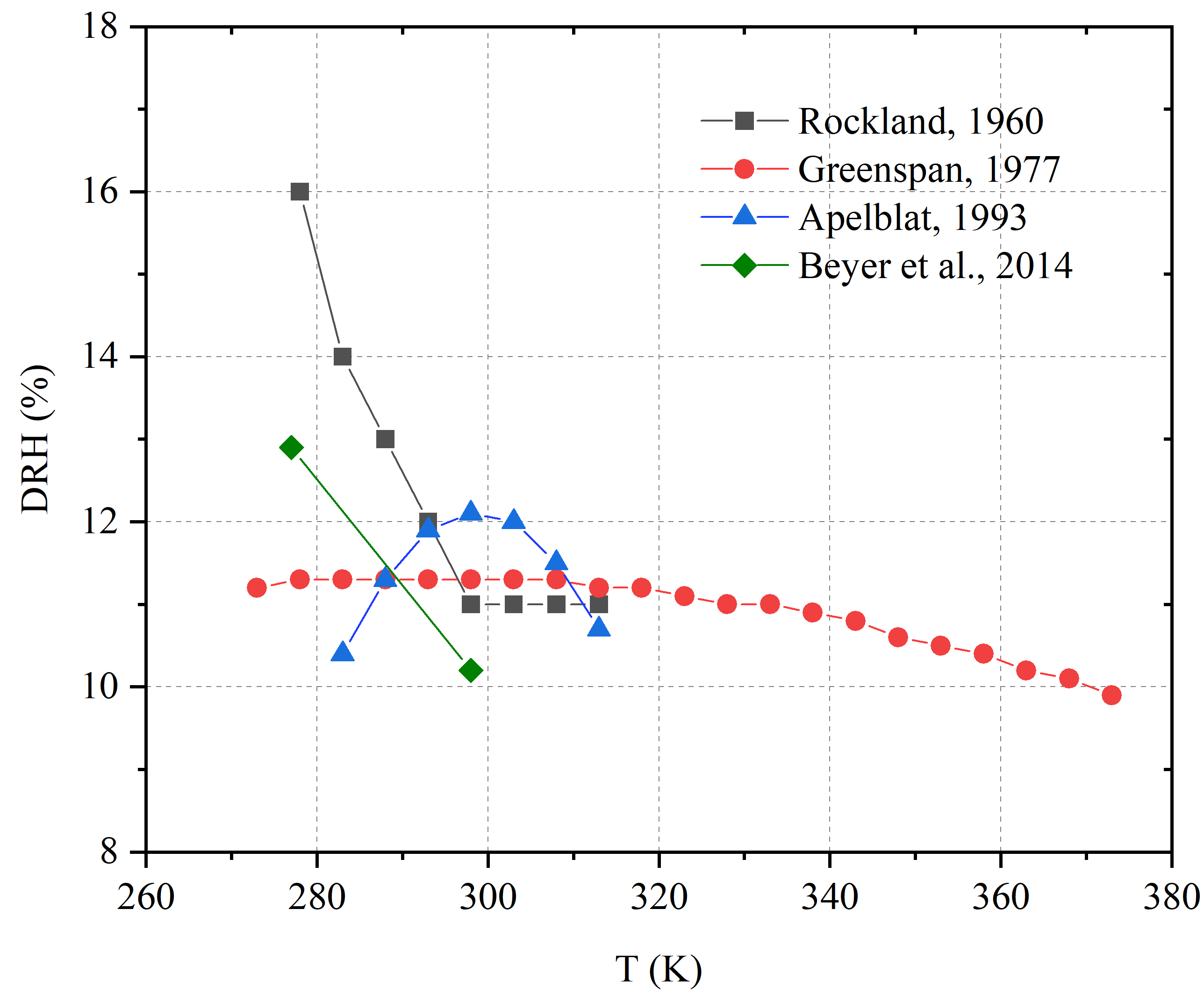


Four studies (Rockland, 1960; Greenspan, 1977; Apelblat, 1993; Beyer et al., 2014) measured DRH of LiCl at different RH. The DRH measured by Greenspan (1977), did not change significantly when temperature increased from 273 to 318 K (being ~11.3%), and then slightly decreased with temperature from 11.2±0.2% at 318 K to 9.9±0.8% at 373 K; in the second study (Apelblat, 1993), it was measured to be 10.4-12.1%, largely independent of temperature (283-313 K). Beyer et al. (2014) found that the DRH of LiCl decreased with temperature from 12.9±1% at 277 K to 10.2±1 at 298 K; Rockland (1960) found that it first decreased with temperature from 16% at 278 K to 11% at 298 K, and showed no significant change when temperature further increased to 313 K.

One study (Schindelholz et al., 2014) measured the ERH of LiCl, and it was determined to be 1-4% at 294 K.

The DRH of LiCl∙H_2_O was measured to be 11.1% at 298 K (Stokes and Robinson, 1949), using the nonisopiestic method.

**Preferred values at 298 K for LiCl:**

DRH: 9-12%

ERH: 1-4%

**Preferred values at 298 K for LiCl ∙H_2_O:**

DRH: 11%

ERH: no preferred value

**References:**

Apelblat, A.: The vapor-pressures of saturated aqueous lithium-chloride, sodium-bromide, sodium-nitrate, ammonium-nitrate, and ammonium-chloride at temperatures from 283 K to 313 K, Journal of Chemical Thermodynamics, 25, 63-71, 1993.

Arenas, K. J. L., Schill, S. R., Malla, A., and Hudson, P. K.: Deliquescence Phase Transition Measurements by Quartz Crystal Microbalance Frequency Shifts, Journal of Physical Chemistry A, 116, 7658-7667, 2012.

Beyer, K. D., Schroeder, J. R., and Kissinger, J. A.: Temperature-Dependent Deliquescence Relative Humidities and Water Activities Using Humidity Controlled Thermogravimetric Analysis with Application to Malonic Acid, Journal of Physical Chemistry A, 118, 2488-2497, 2014.

Greenspan, L.: Humidity fixed-points of binary saturated aqueous-solutions, Journal of Research of the National Bureau of Standards Section a-Physics and Chemistry, 81, 89-96, 1977.

Pearce, J. N., and Nelson, A. F.: The vapor pressures of aqueous solutions of lithium nitrate and the activity coefficients of some alkali salts in solutions of high concentration at 25 degrees, Journal of the American Chemical Society, 54, 3544-3555, 1932.

Rockland, L. B.: Saturated Salt Solutions for Static Control of Relative Humidity between 5° and 40°C., Analytical Chemistry, 32, 1375-1376, 1960.

Schindelholz, E., Tsui, L.-k., and Kelly, R. G.: Hygroscopic Particle Behavior Studied by Interdigitated Array Microelectrode Impedance Sensors, Journal of Physical Chemistry A, 118, 167-177, 2014.

Stokes, R. H., and Robinson, R. A.: Standard Solutions for Humidity Control at 25°C., Industrial and Engineering Chemistry, 41, 2013-2013, 1949.

Tang, I. N., Munkelwitz, H. R., and Davis, J. G.: Aerosol growth studies—II. Preparation and growth measurements of monodisperse salt aerosols, Journal of aerosol science, 8, 149-159, 1977.

## NaCl (sodium chloride)

| Reference | *T* (K) | *D* | DRH (%) | ERH (%) | Techniques/Comments |
| --- | --- | --- | --- | --- | --- |
| Adams and Merz, 1929 | 283 | - | 76.6 | - | Isopiestic method |
|  | 288 |  | 77.0 |  |  |
|  | 293 |  | 77.6 |  |  |
|  | 298 |  | 75.5 |  |  |
|  | 303 |  | 75.2 |  |  |
|  | 313 |  | 74.7 |  |  |
|  | 323 |  | 74.1 |  |  |
| Stokes and Robinson, 1949 | 298 | - | 75.3 | - | Nonisopiestic method |
| Rockland, 1960 | 278 | - | 76 | - | Nonisopiestic method |
|  | 283 |  | 75 |  |  |
|  | 288 |  | 75 |  |  |
|  | 293 |  | 75 |  |  |
|  | 298 |  | 75 |  |  |
|  | 303 |  | 75 |  |  |
|  | 308 |  | 75 |  |  |
|  | 313 |  | 75 |  |  |
| Greenspan, 1977 | 273 | - | 75.5±0.3 | - | Nonisopiestic method |
|  | 278 |  | 75.7±0.3 | - |  |
|  | 283 |  | 75.7±0.2 | - |  |
|  | 288 |  | 75.6±0.2 | - |  |
|  | 293 |  | 75.5±0.1 | - |  |
|  | 298 |  | 75.3±0.1 | - |  |
|  | 303 |  | 75.1±0.1 | - |  |
|  | 308 |  | 74.9±0.1 | - |  |
|  | 313 |  | 74.7±0.1 | - |  |
|  | 318 |  | 74.5±0.2 | - |  |
|  | 323 |  | 74.4±0.2 | - |  |
|  | 328 |  | 74.4±0.2 | - |  |
|  | 333 |  | 74.5±0.3 | - |  |
|  | 338 |  | 74.7±0.4 | - |  |
|  | 343 |  | 75.1±0.5 | - |  |
|  | 348 |  | 75.6±0.6 | - |  |
|  | 353 |  | 76.3±0.7 | - |  |
| Tang et al., 1977 | 298 | 0.3-2 μm | 75.7±0.4 | - | H-DMA-OPC |
| Charlson et al., 1978 | 298 | - | 75 | - | Nonisopiestic method |
| Cohen et al., 1987 | 293 | 20 μm | 74-76 | 44 | EDB |
| Tang and Munkelwitz, 1993 | 298 | 6-8 μm | 75.3±0.1 | - | EDB |
| Richardson and Snyder, 1994 | 298 | 10 μm | 75.3 | 45.5±0.6 | EDB |
| Cziczo et al., 1997 | 298 | 0.45 μm | 75±1 | 43±2 | FTIR |
| Dai et al., 1997 | 298 | - | 73 | - | AFM |
| Tang et al., 1997 | 298 | 6-8 μm | 75.3 | 45-48 | EDB |
| Weis and Ewing, 1999 | 296 | ~0.4 μm | - | 50 | FTIR |
| Cruz and Pandis, 2000 | 297 | 50-120 nm | 75±1 | - | HTDMA |
| Cziczo and Abbatt, 2000 | 253 | 0.18 μm | 80±4 | 35±4 | FTIR |
|  | 263 |  | 77±3 | 41±4 |  |
|  | 273 |  | 78±2 | 38±3 |  |
|  | 283 |  | 75±2 | 45±3 |  |
| Koop et al., 2000 | 249-273 | 5-25 μm | 76±4 | 40±4 | DSC-FCM |
| Lee and Hsu, 2000 | 294 | ~1 μm | 76 | 47 | Katharometer |
| Hämeri et al., 2001 | 298 | 8 nm | 80.9±2 | - | HTDMA |
|  |  | 10 nm | 82.4±2 | - |  |
|  |  | 15 nm | 81.3±2 | - |  |
|  |  | 30 nm | 75.6±1 | - |  |
|  |  | 50 nm | 76.0±1 | - |  |
| Joutsensaari et al., 2001 | 296 | 100 nm | 75 | - | HTDMA |
| Ebert et al., 2002 | 278 | 0.1-20 μm | 78.0±1.3 | - | ESEM |
| Gysel et al., 2002 | 263 | 100 nm | 75±1.5 | - | HTDMA |
|  | 293 |  | 75±1.2 | - |  |
| Krueger et al., 2003 | 288 | ~1μm | 75±0.8 | - | ESEM |
| Wise et al., 2005 | 279 | 0.1-4 μm | 76 | 42 | ETEM |
| Biskos et al., 2006 | 298 | 6 nm | 87±2.5 | 53±2.5 | HTDMA |
|  |  | 8 nm | 84±2.5 | 50±2.5 |  |
|  |  | 10 nm | 82±2.5 | 49±2.5 |  |
|  |  | 15 nm | 79±2.5 | 47±2.5 |  |
|  |  | 20 nm | 78±2.5 | 46±2.5 |  |
|  |  | 30 nm | 77±2.5 | 45±2.5 |  |
|  |  | 40 nm | 76±2.5 | 44±2.5 |  |
|  |  | 60 nm | 76±2.5 | 44±2.5 |  |
| Schuttlefield et al., 2007 | 298 | - | 75±1 | 51 | ATR-FTIR |
| Woods et al., 2007 | 298 | ~100 nm | 76±2 | 42±2 | HTDMA |
| Liu Y et al., 2008 | 296 | ~1μm | 76 | 48 | FTIR |
| Lu et al., 2008 | 298 | 1-5 μm | 72±2.5 | - | ATR-FTIR |
| Ahn et al., 2010 | 298 | 2.5-10 μm | 75.5±0.2 | 47.7±0.5 | Optical microscopy |
| Ghorai and Tivanski, 2010 | 299 | ~1 μm | 73±2 | 46±2 | STXM |
| Ma et al., 2010 | 274 | 1-30μm | 75 | - | PSA |
| Pope et al., 2010 | 295 | 20-50 μm | 75.5±1 | - | EDB |
| Mikhailov et al., 2011 | 295 | ~1 μm | 75 | 64 | Katharometer |
| Arenas et al., 2012 | 298 | - | 77.1±2.1 | - | QCM |
| Beyer et al., 2014 | 277 | - | 77.2±1 | - | VSA |
|  | 298 | - | 76.0±1 |  |  |
| Ghorai et al., 2014 | 298 | 0.2~1.2 μm | ~75 | ~48 | Micro-FTIR |
| Li et al., 2014 | 298 | 1-5 μm | 75.6±0.3 | 47.4±0.5 | Optical microscopy |
| Schindelholz et al., 2014 | 294 | ~100 μm | 76±1.5 | 50±1.5 | Electrical conductivity/ impedance method |
|  |  |  |  |  |  |
| Zeng et al., 2014 | 268 | ~1 μm | 78±2 | 43±2 | ATR-FTIR |
|  | 278 |  | 77±2 | 45±2 |  |
|  | 285 |  | 76±2 | 46±2 |  |
|  | 296 |  | 75±2 | 48±2 |  |
| Gupta et al., 2015 | 296 | 1-10 μm | 75.1±0.5 | 45.7-47.6 | Micro-Raman |
| Laskina et al., 2015 | 298 K | 100 nm | 74.0±0.2 | 43.0±1.0 | HTDMA |
|  |  | 3-12 μm | 77.5±2.1 | 52.8±1.1 | Micro-Raman |
| Morris et al., 2016 | 298 | ~1 μm | 75 | 44 | AFM |
| Gu et al., 2017 | 278 | - | 76.0±1 | - | VSA |
|  | 283 |  | 75.7±1 |  |  |
|  | 288 |  | 75.7±1 |  |  |
|  | 293 |  | 75.6±1 |  |  |
|  | 298 |  | 75.2±1 |  |  |
|  | 303 |  | 75.5±1 |  |  |
| Zieger et al., 2017 | 298 | 50-150 nm | 74.3±1.5 | - | HTDMA |
| Zielinski et al., 2018 | 292-295 | 300 nm | 72-77 | 42-51 | MEMS |
| Ma et al., 2019 | 298 | ~3 μm | ~75 | 46-51 | Vacuum-FTIR |
| Wang et al., 2020 | 298 | 10 nm | 86±1 | - | AFM |
|  |  | 200 nm | 78±1 |  |  |

**Comments:**

The DRH of NaCl was measured by a large number of studies (Adams and Merz, 1929; Stokes and Robinson, 1949; Rockland, 1960; Greenspan, 1977; Tang et al., 1977; Charlson et al., 1978; Cohen et al., 1987; Tang and Munkelwitz, 1993; Richardson and Snyder, 1994; Cziczo et al., 1997; Dai et al., 1997; Tang et al., 1997; Weis and Ewing, 1999; Cruz and Pandis, 2000; Cziczo and Abbatt, 2000; Koop et al., 2000; Lee and Hsu, 2000; Hämeri et al., 2001; Joutsensaari et al., 2001; Ebert et al., 2002; Gysel et al., 2002; Krueger et al., 2003; Wise et al., 2005; Biskos et al., 2006; Schuttlefield et al., 2007; Woods et al., 2007; Liu et al., 2008; Lu et al., 2008; Ahn et al., 2010; Ghorai and Tivanski, 2010; Ma et al., 2010; Pope et al., 2010; Mikhailov et al., 2011; Arenas et al., 2012; Beyer et al., 2014; Ghorai et al., 2014; Li et al., 2014; Schindelholz et al., 2014; Zeng et al., 2014; Gupta et al., 2015; Laskina et al., 2015; Morris et al., 2016; Gu et al., 2017; Zieger et al., 2017; Zielinski et al., 2018; Ma et al., 2019; Wang et al., 2020). It was measured to be 73-77% at 298 K, showing good agreement among diferent studies.


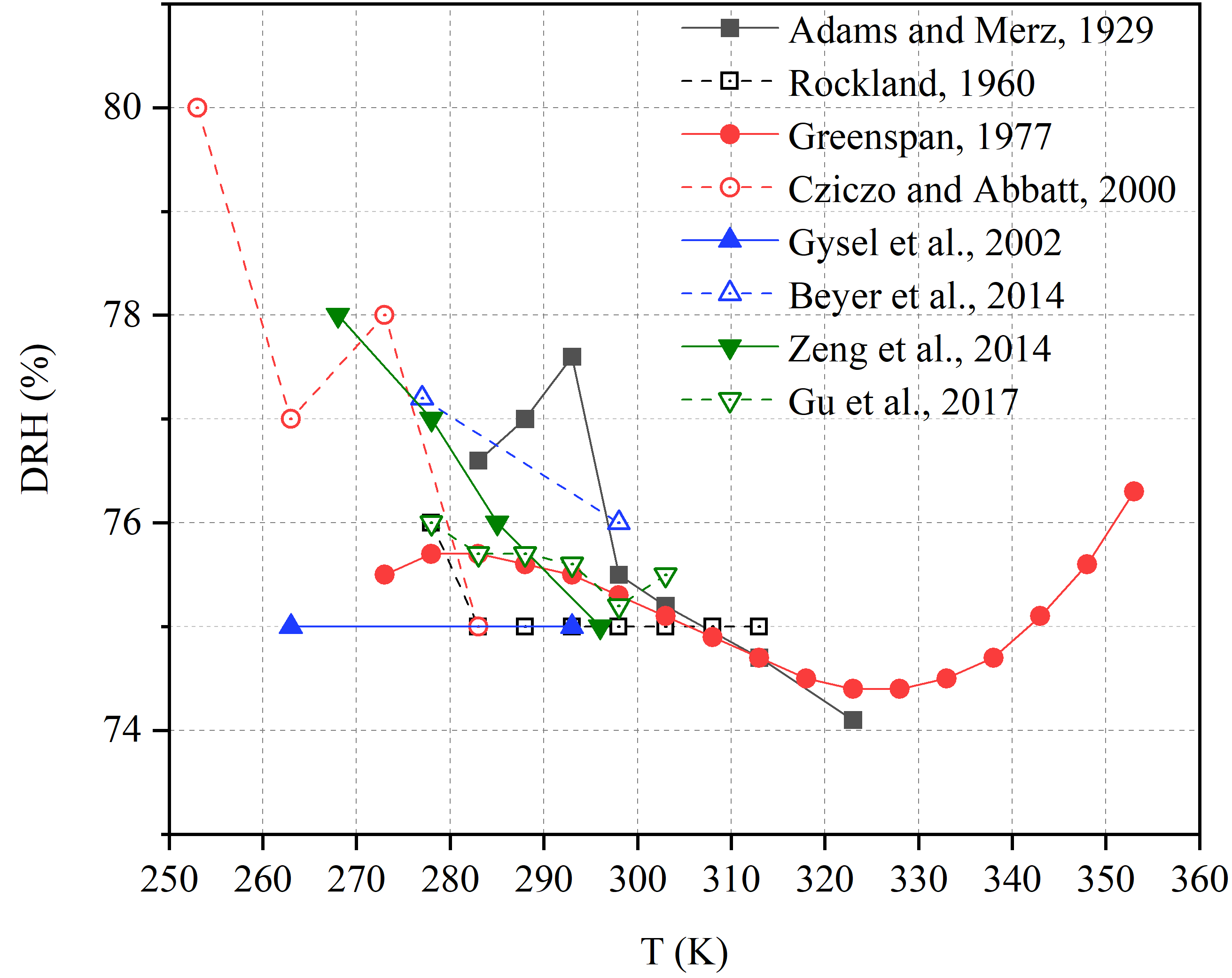


Eight studies (Adams and Merz, 1929; Rockland, 1960; Greenspan, 1977; Cziczo and Abbatt, 2000; Gysel et al., 2002; Beyer et al., 2014; Zeng et al., 2014; Gu et al., 2017) measured DRH of NaCl at different RH. The DRH at 273-353 K was measured to range from 74.4±0.2% to 76.3±0.7% (Greenspan, 1977), showing no significant dependence on temperature. Three studies (Adams and Merz, 1929; Cziczo and Abbatt, 2000; Zeng et al., 2014) revealed a slightly negative dependence on temperature: Adams and Merz (1929) found that the DRH of NaCl decreased slightly with temperature from 76.6% at 283 to 74.1% at 323 K, Cziczo and Abbatt (2000) suggested that it decreased with temperature from 80±4% at 253 to 75±2% at 283 K, and Zeng et al. (2014) found that it decreased slightly with temperature from 78±2% at 268 K to 75±2% at 296 K. The other four studies (Rockland, 1960; Gysel et al., 2002; Beyer et al., 2014; Gu et al., 2017) also revealed no significant dependence on temperature: the DRH of NaCl was measured to be 75-76% at 278-313 K (Rockland, 1960), ~75% at 263-293 K (Gysel et al., 2002), 77.2±1% at 277 K and 76.0±1% at 298 K (Beyer et al., 2014), and between 75.2±1% and 76±1% at 278-303 K (Gu et al., 2017).

The ERH values of NaCl were measured by a number of studies (Cohen et al., 1987; Richardson and Snyder, 1994; Cziczo et al., 1997; Tang et al., 1997; Weis and Ewing, 1999; Cziczo and Abbatt, 2000; Koop et al., 2000; Lee and Hsu, 2000; Wise et al., 2005; Biskos et al., 2006; Schuttlefield et al., 2007; Woods et al., 2007; Liu et al., 2008; Ahn et al., 2010; Ghorai and Tivanski, 2010; Ghorai et al., 2014; Li et al., 2014; Schindelholz et al., 2014; Zeng et al., 2014; Gupta et al., 2015; Laskina et al., 2015; Morris et al., 2016; Zielinski et al., 2018; Ma et al., 2019). The ERH was determined to be 41-51% at 298 K, showed relatively good agreement among different studies. However, it was measured to be 64% at 295 K (Mikhailov et al., 2011), significantly higher than those reported by the other studies.


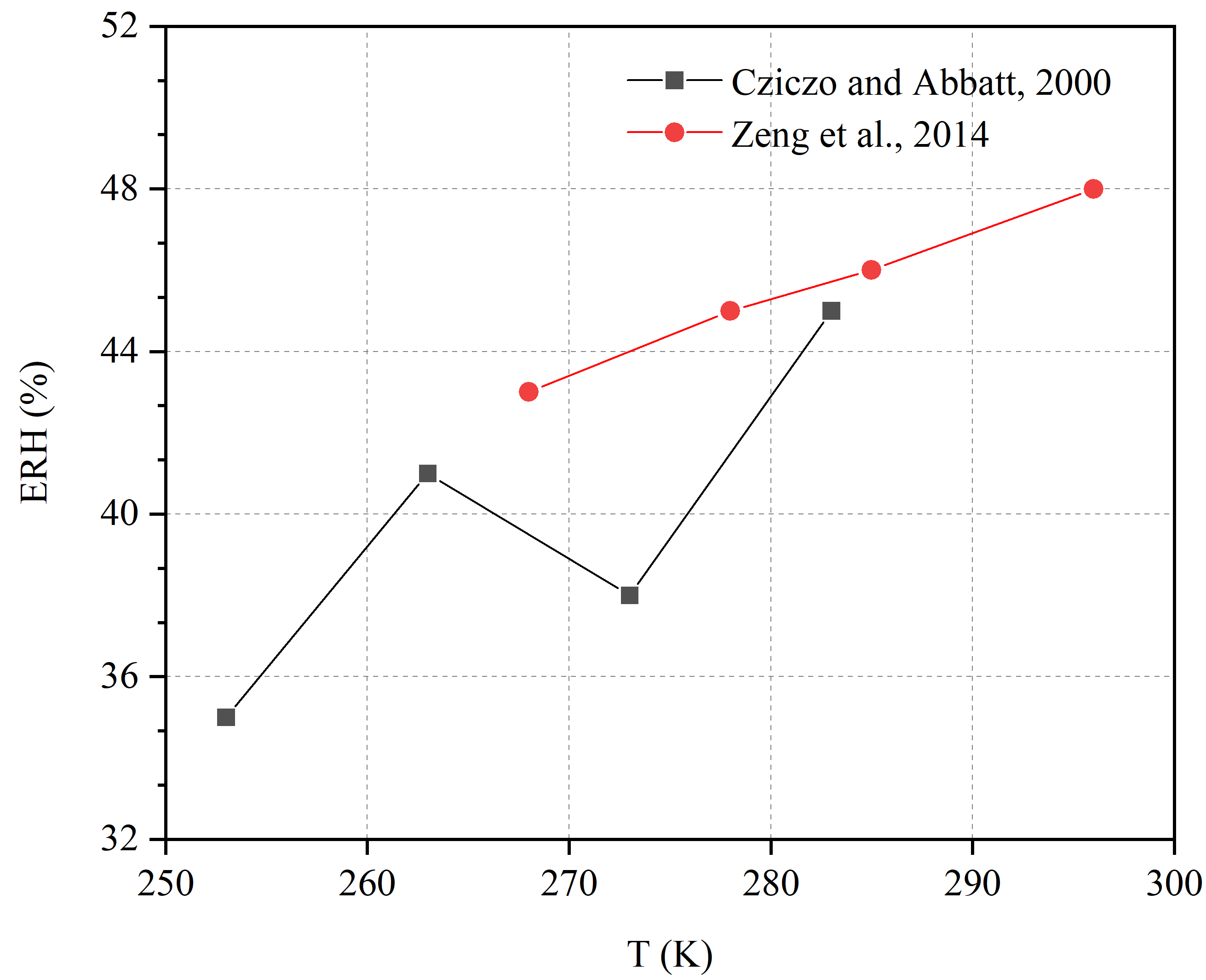


Temperature dependence of ERH values of NaCl was investigated by two studies (Cziczo and Abbatt, 2000; Zeng et al., 2014). Cziczo and Abbatt (2000) found that the ERH of NaCl increased with temperature from 35±4% at 253 K to 45±3% at 283 K; similarly, Zeng et al. (2014) found that it increased with temperature from 43±2% at 268 K to 48±2% at 296 K.

**Preferred values at 298 K for NaCl:**

DRH: 73-77%

ERH: 41-51%

**References:**

Adams, J. R., and Merz, A. R.: Hygroscopicity of fertilizer materials and mixtures, Industrial and Engineering Chemistry, 21, 305-307, 1929.

Ahn, K.-H., Kim, S.-M., Jung, H.-J., Lee, M.-J., Eom, H.-J., Maskey, S., and Ro, C.-U.: Combined Use of Optical and Electron Microscopic Techniques for the Measurement of Hygroscopic Property, Chemical Composition, and Morphology of Individual Aerosol Particles, Analytical Chemistry, 82, 7999-8009, 2010.

Arenas, K. J. L., Schill, S. R., Malla, A., and Hudson, P. K.: Deliquescence Phase Transition Measurements by Quartz Crystal Microbalance Frequency Shifts, Journal of Physical Chemistry A, 116, 7658-7667, 2012.

Beyer, K. D., Schroeder, J. R., and Kissinger, J. A.: Temperature-Dependent Deliquescence Relative Humidities and Water Activities Using Humidity Controlled Thermogravimetric Analysis with Application to Malonic Acid, Journal of Physical Chemistry A, 118, 2488-2497, 2014.

Biskos, G., Malinowski, A., Russell, L. M., Buseck, P. R., and Martin, S. T.: Nanosize effect on the deliquescence and the efflorescence of sodium chloride particles, Aerosol Science and Technology, 40, 97-106, 2006.

Charlson, R. J., Covert, D. S., Larson, T. V., and Waggoner, A. P.: Chemical properties of tropospheric sulfur aerosols, Atmospheric Environment (1967), 12, 39-53, 1978.

Cohen, M. D., Flagan, R. C., and Seinfeld, J. H.: Studies of concentrated electrolyte-solutions using the electrodynamic balance .1. Water activities for single-electrolyte solutions, Journal of Physical Chemistry, 91, 4563-4574, 1987.

Cohen, M. D., Flagan, R. C., and Seinfeld, J. H.: Studies of concentrated electrolyte solutions using the electrodynamic balance. 3. Solute nucleation, Journal of Physical Chemistry, 91, 4583-4590, 1987.

Cruz, C. N., and Pandis, S. N.: Deliquescence and hygroscopic growth of mixed inorganic-organic atmospheric aerosol, Environmental Science & Technology, 34, 4313-4319, 2000.

Cziczo, D. J., and Abbatt, J. P. D.: Infrared observations of the response of NaCl, MgCl_2_, NH_4_HSO_4_, and NH_4_NO_3_ aerosols to changes in relative humidity from 298 to 238 K, Journal of Physical Chemistry A, 104, 2038-2047, 2000.

Cziczo, D. J., Nowak, J. B., Hu, J. H., and Abbatt, J. P. D.: Infrared spectroscopy of model tropospheric aerosols as a function of relative humidity: Observation of deliquescence and crystallization, Journal of Geophysical Research-Atmospheres, 102, 18843-18850, 1997.

Dai, Q., Hu, J., and Salmeron, M.: Adsorption of water on NaCl (100) surfaces: Role of atomic steps, Journal of Physical Chemistry B, 101, 1994-1998, 1997.

Ebert, M., Inerle-Hof, M., and Weinbruch, S.: Environmental scanning electron microscopy as a new technique to determine the hygroscopic behaviour of individual aerosol particles, Atmospheric Environment, 36, 5909-5916, 2002.

Ghorai, S., and Tivanski, A. V.: Hygroscopic Behavior of Individual Submicrometer Particles Studied by X-ray Spectromicroscopy, Analytical Chemistry, 82, 9289-9298, 2010.

Ghorai, S., Wang, B., Tivanski, A., and Laskin, A.: Hygroscopic Properties of Internally Mixed Particles Composed of NaCl and Water-Soluble Organic Acids, Environmental Science & Technology, 48, 2234-2241, 2014.

Greenspan, L.: Humidity fixed-points of binary saturated aqueous-solutions, Journal of Research of the National Bureau of Standards Section a-Physics and Chemistry, 81, 89-96, 1977.

Gu, W., Li, Y., Zhu, J., Jia, X., Lin, Q., Zhang, G., Ding, X., Song, W., Bi, X., Wang, X., and Tang, M.: Investigation of water adsorption and hygroscopicity of atmospherically relevant particles using a commercial vapor sorption analyzer, Atmospheric Measurement Techniques, 10, 3821-3832, 2017.

Gupta, D., Eom, H. J., Cho, H. R., and Ro, C. U.: Hygroscopic behavior of NaCl-MgCl_2_ mixture particles as nascent sea-spray aerosol surrogates and observation of efflorescence during humidification, Atmospheric Chemistry and Physics, 15, 11273-11290, 2015.

Gysel, M., Weingartner, E., and Baltensperger, U.: Hygroscopicity of aerosol particles at low temperatures. 2. Theoretical and experimental hygroscopic properties of laboratory generated aerosols, Environmental Science & Technology, 36, 63-68, 2002.

Hämeri, K., Laaksonen, A., Vakeva, M., and Suni, T.: Hygroscopic growth of ultrafine sodium chloride particles, Journal of Geophysical Research-Atmospheres, 106, 20749-20757, 2001.

Joutsensaari, J., Vaattovaara, P., Vesterinen, M., Hameri, K., and Laaksonen, A.: A novel tandem differential mobility analyzer with organic vapor treatment of aerosol particles, Atmospheric Chemistry and Physics, 1, 51-60, 2001.

Koop, T., Kapilashrami, A., Molina, L. T., and Molina, M. J.: Phase transitions of sea-salt/water mixtures at low temperatures: Implications for ozone chemistry in the polar marine boundary layer, Journal of Geophysical Research-Atmospheres, 105, 26393-26402, 2000.

Krueger, B. J., Grassian, V. H., Iedema, M. J., Cowin, J. P., and Laskin, A.: Probing heterogeneous chemistry of individual atmospheric particles using scanning electron microscopy and energy-dispersive X-ray analysis, Analytical Chemistry, 75, 5170-5179, 2003.

Laskina, O., Morris, H. S., Grandquist, J. R., Qin, Z., Stone, E. A., Tivanski, A. V., and Grassian, V. H.: Size Matters in the Water Uptake and Hygroscopic Growth of Atmospherically Relevant Multicomponent Aerosol Particles, The journal of physical chemistry. A, 119, 4489-4497, 2015.

Lee, C.-T., and Hsu, W.-C.: The measurement of liquid water mass associated with collected hygroscopic particles, Journal of Aerosol Science, 31, 189-197, 2000.

Li, X., Gupta, D., Eom, H.-J., Kim, H., and Ro, C.-U.: Deliquescence and efflorescence behavior of individual NaCl and KCl mixture aerosol particles, Atmospheric Environment, 82, 36-43, 2014.

Liu, Y., Yang, Z., Desyaterik, Y., Gassman, P. L., Wang, H., and Laskin, A.: Hygroscopic behavior of substrate-deposited particles studied by micro-FT-IR spectroscopy and complementary methods of particle analysis, Analytical Chemistry, 80, 633-642, 2008.

Lu, P. D., He, T., and Zhang, Y. H.: Relative humidity anneal effect on hygroscopicity of aerosol particles studied by rapid-scan FTIR-ATR spectroscopy, Geophysical Research Letters, 35, 3, 2008.

Ma, Q., Liu, Y., and He, H.: The Utilization of Physisorption Analyzer for Studying the Hygroscopic Properties of Atmospheric Relevant Particles, Journal of Physical Chemistry A, 114, 4232-4237, 2010.

Ma, S.-S., Yang, W., Zheng, C.-M., Pang, S.-F., and Zhang, Y.-H.: Subsecond measurements on aerosols: From hygroscopic growth factors to efflorescence kinetics, Atmospheric Environment, 210, 177-185, 2019.

Mikhailov, E. F., Merkulov, V. V., Vlasenko, S. S., Ryshkevich, T. I., and Poeschl, U. J.: Filter-based differential hygroscopicity analyzer of aerosol particles, Izvestiya Atmospheric and Oceanic Physics, 47, 747-759, 2011.

Morris, H. S., Estillore, A. D., Laskina, O., Grassian, V. H., and Tivanski, A. V.: Quantifying the Hygroscopic Growth of Individual Submicrometer Particles with Atomic Force Microscopy, Analytical Chemistry, 88, 3647-3654, 2016.

Pope, F. D., Dennis-Smither, B. J., Griffiths, P. T., Clegg, S. L., and Cox, R. A.: Studies of Single Aerosol Particles Containing Malonic Acid, Glutaric Acid, and Their Mixtures with Sodium Chloride. I. Hygroscopic Growth, Journal of Physical Chemistry A, 114, 5335-5341, 2010.

Richardson, C. B., and Snyder, T. D.: A study of heterogeneous nucleation in aqueous-solutions, Langmuir, 10, 2462-2465, 1994.

Rockland, L. B.: Saturated Salt Solutions for Static Control of Relative Humidity between 5° and 40°C., Analytical Chemistry, 32, 1375-1376, 1960.

Schindelholz, E., Tsui, L.-k., and Kelly, R. G.: Hygroscopic Particle Behavior Studied by Interdigitated Array Microelectrode Impedance Sensors, Journal of Physical Chemistry A, 118, 167-177, 2014.

Schuttlefield, J., Al-Hosney, H., Zachariah, A., and Grassian, V. H.: Attenuated Total Reflection Fourier Transform Infrared Spectroscopy to Investigate Water Uptake and Phase Transitions in Atmospherically Relevant Particles, Applied Spectroscopy, 61, 283-292, 2007.

Stokes, R. H., and Robinson, R. A.: Standard Solutions for Humidity Control at 25°C., Industrial and Engineering Chemistry, 41, 2013-2013, 1949.

Tang, I. N., and Munkelwitz, H. R.: Composition and temperature-dependence of the deliquescence properties of hygroscopic aerosols, Atmospheric Environment Part a-General Topics, 27, 467-473, 1993.

Tang, I. N., Munkelwitz, H. R., and Davis, J. G.: Aerosol growth studies—II. Preparation and growth measurements of monodisperse salt aerosols, Journal of aerosol science, 8, 149-159, 1977.

Tang, I. N., Tridico, A. C., and Fung, K. H.: Thermodynamic and optical properties of sea salt aerosols, Journal of Geophysical Research-Atmospheres, 102, 23269-23275, 1997.

Wang, X., Lei, H., Berger, R., Zhang, Y., Su, H., and Cheng, Y.: Hygroscopic properties of NaCl nanoparticles on the surface: a scanning force microscopy study, Physical Chemistry Chemical Physics, 22, 9967-9973, 2020.

Weis, D. D., and Ewing, G. E.: Water content and morphology of sodium chloride aerosol particles, Journal of Geophysical Research-Atmospheres, 104, 21275-21285, 1999.

Wise, M. E., Biskos, G., Martin, S. T., Russell, L. M., and Buseck, P. R.: Phase transitions of single salt particles studied using a transmission electron microscope with an environmental cell, Aerosol Science and Technology, 39, 849-856, 2005.

Woods, E., III, Kim, H. S., Wivagg, C. N., Dotson, S. J., Broekhuizen, K. E., and Frohardt, E. F.: Phase transitions and surface morphology of surfactant-coated aerosol particles, Journal of Physical Chemistry A, 111, 11013-11020, 2007.

Zeng, G., Kelley, J., Kish, J. D., and Liu, Y.: Temperature-dependent deliquescent and efflorescent properties of methanesulfonate sodium studied by ATR-FTIR spectroscopy, The journal of physical chemistry. A, 118, 583-591, 2014.

Zieger, P., Vaisanen, O., Corbin, J. C., Partridge, D. G., Bastelberger, S., Mousavi-Fard, M., Rosati, B., Gysel, M., Krieger, U. K., Leck, C., Nenes, A., Riipinen, I., Virtanen, A., and Salter, M. E.: Revising the hygroscopicity of inorganic sea salt particles, Nature Communications, 8, 2017.

Zielinski, A. T., Gallimore, P. J., Griffiths, P. T., Jones, R. L., Seshia, A. A., and Kalberer, M.: Measuring Aerosol Phase Changes and Hygroscopicity with a Microresonator Mass Sensor, Analytical Chemistry, 90, 9716-9724, 2018.

## KCl (potassium chloride)

| Reference | *T* (K) | *D* | DRH (%) | ERH (%) | Techniques/Comments |
| --- | --- | --- | --- | --- | --- |
| Adams and Merz, 1929 | 283 | - | 88.3 | - | Isopiestic method |
|  | 288 |  | 86.2 |  |  |
|  | 293 |  | 85.7 |  |  |
|  | 298 |  | 83.4 |  |  |
|  | 303 |  | 84.0 |  |  |
|  | 313 |  | 81.2 |  |  |
|  | 323 |  | 80.0 |  |  |
| Pearce and Nelson, 1932 | 298 | - | 84.3 | - | Nonisopiestic method |
| Stokes and Robinson, 1949 | 298 | - | 84.3 | - | Nonisopiestic method |
| Rockland, 1960 | 278 | - | 88 | - | Nonisopiestic method |
|  | 283 |  | 87 |  |  |
|  | 288 |  | 87 |  |  |
|  | 293 |  | 86 |  |  |
|  | 298 |  | 86 |  |  |
|  | 303 |  | 84 |  |  |
|  | 308 |  | 84 |  |  |
|  | 313 |  | 83 |  |  |
| Greenspan, 1977 | 273 | - | 88.6±0.5 | - | Nonisopiestic method |
|  | 278 |  | 87.7±0.5 |  |  |
|  | 283 |  | 86.8±0.4 |  |  |
|  | 288 |  | 85.9±0.3 |  |  |
|  | 293 |  | 85.1±0.3 |  |  |
|  | 298 |  | 84.3±0.3 |  |  |
|  | 303 |  | 83.6±0.3 |  |  |
|  | 308 |  | 83.0±0.3 |  |  |
|  | 313 |  | 82.3±0.3 |  |  |
|  | 318 |  | 81.7±0.3 |  |  |
|  | 323 |  | 81.2±0.3 |  |  |
|  | 328 |  | 80.7±0.4 |  |  |
|  | 333 |  | 80.3±0.4 |  |  |
|  | 338 |  | 79.9±0.5 |  |  |
|  | 343 |  | 79.5±0.6 |  |  |
|  | 348 |  | 79.2±0.7 |  |  |
|  | 353 |  | 78.9±0.8 |  |  |
|  | 358 |  | 78.7±0.9 |  |  |
|  | 363 |  | 78.5±1.0 |  |  |
| Tang et al., 1977 | 298 | 0.3-2 μm | 84.3 | - | H-DMA-OPC |
| Cohen et al., 1987 | 293 | 20 μm | 83-86 | 59 | EDB |
| Tang and Munkelwitz, 1993 | 298 | 6-8 μm | 84.2±0.3 | - | EDB |
| Hamza et al., 2004 | 293 | 38-70 μm | 86±1 | 57±1 | EDB |
| Freney et al., 2009 | 288 | ~1 μm | 85±1 | 56±1 | ETEM |
| Ahn et al., 2010 | 298 | 2.5-10 μm | 84.6±0.3 | 58.7±0.7 | Optical microscopy |
| Carrico et al., 2010 | 298 | 100 | 83±2 | 52±2 | HTDMA |
| Beyer et al., 2014 | 277 | - | 87.7±1 | - | VSA |
|  | 298 | - | 84.2±1 |  |  |
| Li et al., 2014 | 298 | 1-5 μm | 84.7±0.2 | 57.7±0.5 | Optical microscopy |
| Schindelholz et al., 2014 | 294 | ~100 μm | 88±1.5 | 59-61 | Electrical conductivity/ impedance method |
| Gu et al., 2017 | 278 | - | 86.7±1 | - | VSA |
|  | 283 |  | 86.3±1 |  |  |
|  | 288 |  | 85.6±1 |  |  |
|  | 293 |  | 85.0±1 |  |  |
|  | 298 |  | 83.9±1 |  |  |
|  | 303 |  | 83.4±1 |  |  |
| Jing et al., 2017 | 297 | 100 nm | 84.0±1.5 | - | HTDMA |
| Giamarelou et al., 2018 | 298 | 8 | n. o. | - | HTDMA |
|  |  | 15 | 90.5-91.2 | n. o. |  |
|  |  | 20 | 79.6-85.5 | 52.6-61.8 |  |
|  |  | 40 | 80.8-82.9 | 51.4-60.7 |  |
| Zhang et al., 2020 | 288 | - | 85.5±1.1 | - | VSA |
|  | 293 |  | 84.7±1.1 |  |  |
|  | 298 |  | 84.1±1.1 |  |  |
|  | 303 |  | 83.6±1.0 |  |  |
|  | 308 |  | 83.0±1.0 |  |  |

**Comments:**

A number of studies (Adams and Merz, 1929; Pearce and Nelson, 1932; Stokes and Robinson, 1949; Rockland, 1960; Greenspan, 1977; Tang et al., 1977; Cohen et al., 1987; Tang and Munkelwitz, 1993; Hamza et al., 2004; Freney et al., 2009; Ahn et al., 2010; Carrico et al., 2010; Beyer et al., 2014; Li et al., 2014; Schindelholz et al., 2014; Gu et al., 2017; Jing et al., 2017; Giamarelou et al., 2018; Zhang et al., 2020) measured DRH of KCl at around room temperature. The DRH was measured to be 83-86% at 298 K, showing good agreement.


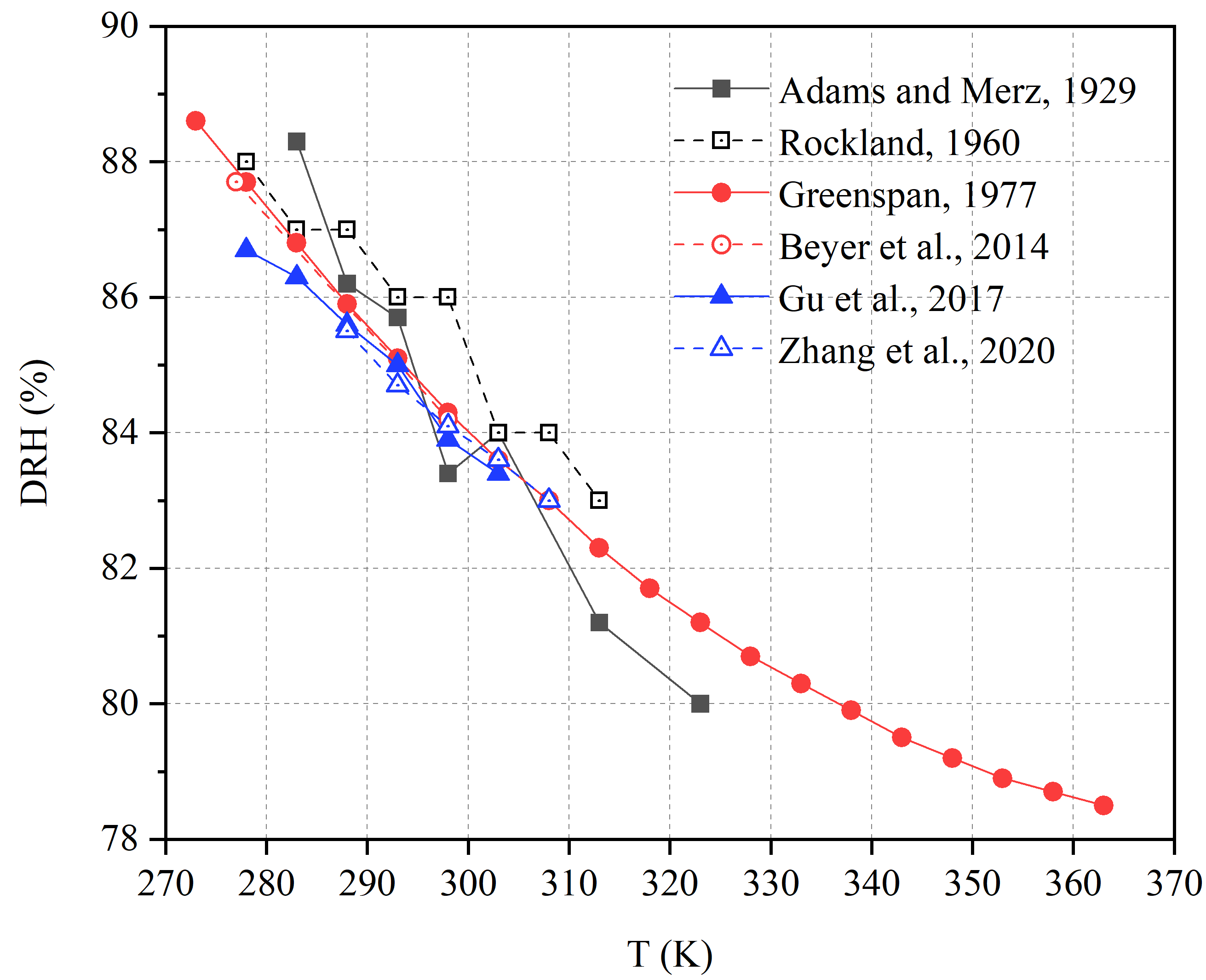


Six studies (Adams and Merz, 1929; Rockland, 1960; Greenspan, 1977; Beyer et al., 2014; Gu et al., 2017; Zhang et al., 2020) measured DRH of KCl at different temperature, and revealed similar temperature dependence. To be more specific, Adams and Merz (1929) found that the DRH of KCl decreased with temperature from 88.3% at 283 to 80.0% at 323 K, Rockland (1960) found that it decreased with temperature from 88% at 278 to 80.0% at 323 K, and Greenspan (1977) suggested that it decreased with temperature from 88.6±0.5% at 273 K to 78.5±1.0% at 363 K; Beyer et al. (2014) found that the DRH of KCl decreased with temperature from 87.7±1% at 277 K and 84.2±1% at 298 K, Gu et al (2017) found that it decreased with temperature from 86.7±1% at 278 K to 83.4±1% at 303 K, and Zhang et al. (2020) suggested that it decreased with temperature from 85.5±1.1% at 288 K to 83.0±1.0% at 308 K.

Eight studies (Cohen et al., 1987; Hamza et al., 2004; Freney et al., 2009; Ahn et al., 2010; Carrico et al., 2010; Li et al., 2014; Schindelholz et al., 2014; Giamarelou et al., 2018) measured the ERH of KCl. The ERH was determined to be 50-60% at 288-298 K, showing relatively good agreement.

**Preferred values at 298 K for KCl:**

DRH: 83-86%

ERH: 50-60%

**References:**

Adams, J. R., and Merz, A. R.: Hygroscopicity of fertilizer materials and mixtures, Industrial and Engineering Chemistry, 21, 305-307, 1929.

Ahn, K.-H., Kim, S.-M., Jung, H.-J., Lee, M.-J., Eom, H.-J., Maskey, S., and Ro, C.-U.: Combined Use of Optical and Electron Microscopic Techniques for the Measurement of Hygroscopic Property, Chemical Composition, and Morphology of Individual Aerosol Particles, Analytical Chemistry, 82, 7999-8009, 2010.

Beyer, K. D., Schroeder, J. R., and Kissinger, J. A.: Temperature-Dependent Deliquescence Relative Humidities and Water Activities Using Humidity Controlled Thermogravimetric Analysis with Application to Malonic Acid, Journal of Physical Chemistry A, 118, 2488-2497, 2014.

Carrico, C. M., Petters, M. D., Kreidenweis, S. M., Sullivan, A. P., McMeeking, G. R., Levin, E. J. T., Engling, G., Malm, W. C., and Collett, J. L., Jr.: Water uptake and chemical composition of fresh aerosols generated in open burning of biomass, Atmospheric Chemistry and Physics, 10, 5165-5178, 2010.

Cohen, M. D., Flagan, R. C., and Seinfeld, J. H.: Studies of concentrated electrolyte-solutions using the electrodynamic balance .1. Water activities for single-electrolyte solutions, Journal of Physical Chemistry, 91, 4563-4574, 1987.

Cohen, M. D., Flagan, R. C., and Seinfeld, J. H.: Studies of concentrated electrolyte solutions using the electrodynamic balance. 3. Solute nucleation, Journal of Physical Chemistry, 91, 4583-4590, 1987.

Freney, E. J., Martin, S. T., and Buseck, P. R.: Deliquescence and Efflorescence of Potassium Salts Relevant to Biomass-Burning Aerosol Particles, Aerosol Science and Technology, 43, 799-807, 2009.

Giamarelou, M., Smith, M., Papapanagiotou, E., Martin, S. T., and Biskos, G.: Hygroscopic properties of potassium-halide nanoparticles, Aerosol Science and Technology, 52, 536-545, 2018.

Greenspan, L.: Humidity fixed-points of binary saturated aqueous-solutions, Journal of Research of the National Bureau of Standards Section a-Physics and Chemistry, 81, 89-96, 1977.

Gu, W., Li, Y., Zhu, J., Jia, X., Lin, Q., Zhang, G., Ding, X., Song, W., Bi, X., Wang, X., and Tang, M.: Investigation of water adsorption and hygroscopicity of atmospherically relevant particles using a commercial vapor sorption analyzer, Atmospheric Measurement Techniques, 10, 3821-3832, 2017.

Hamza, M. A., Berge, B., Mikosch, W., and Ruhl, E.: Homogeneous nucleation of supersaturated KCl-solutions from single levitated microdroplets, Physical Chemistry Chemical Physics, 6, 3484-3489, 2004.

Jing, B., Peng, C., Wang, Y., Liu, Q., Tong, S., Zhang, Y., and Ge, M.: Hygroscopic properties of potassium chloride and its internal mixtures with organic compounds relevant to biomass burning aerosol particles, Scientific reports, 7, 2017.

Li, X., Gupta, D., Eom, H.-J., Kim, H., and Ro, C.-U.: Deliquescence and efflorescence behavior of individual NaCl and KCl mixture aerosol particles, Atmospheric Environment, 82, 36-43, 2014.

Pearce, J. N., and Nelson, A. F.: The vapor pressures of aqueous solutions of lithium nitrate and the activity coefficients of some alkali salts in solutions of high concentration at 25 degrees, Journal of the American Chemical Society, 54, 3544-3555, 1932.

Rockland, L. B.: Saturated Salt Solutions for Static Control of Relative Humidity between 5° and 40°C., Analytical Chemistry, 32, 1375-1376, 1960.

Schindelholz, E., Tsui, L.-k., and Kelly, R. G.: Hygroscopic Particle Behavior Studied by Interdigitated Array Microelectrode Impedance Sensors, Journal of Physical Chemistry A, 118, 167-177, 2014.

Stokes, R. H., and Robinson, R. A.: Standard Solutions for Humidity Control at 25°C., Industrial and Engineering Chemistry, 41, 2013-2013, 1949.

Tang, I. N., and Munkelwitz, H. R.: Composition and temperature-dependence of the deliquescence properties of hygroscopic aerosols, Atmospheric Environment Part a-General Topics, 27, 467-473, 1993.

Tang, I. N., Munkelwitz, H. R., and Davis, J. G.: Aerosol growth studies—II. Preparation and growth measurements of monodisperse salt aerosols, Journal of aerosol science, 8, 149-159, 1977.

Zhang, H., Gu, W., Li, Y. J., and Tang, M.: Hygroscopic properties of sodium and potassium salts as related to saline mineral dusts and sea salt aerosols, Journal of environmental sciences, 95, 65-72, 2020.

## CaCl_2_ (calcium chloride)

| Reference | *T* (K) | *D* | DRH (%) | ERH (%) | Techniques/Comments |
| --- | --- | --- | --- | --- | --- |
| Goldberg and Nuttall, 1978 | 298 | - | 28.3 | - | Nonisopiestic method |
| Cohen et al., 1987 | 293 | 20 μm | n. o. | - | EDB |
| Yang et al., 2006 | 323 | - | 15±1 | - | Electrical conductivity/ impedance method |
| Park et al., 2009 | 298 | 20-50 nm | 29±2 | - | HTDMA |
| Gough et al., 2016 | 223 | 5-20 μm | - | 8.7±1.5 | Micro-Raman |
|  | 233 |  |  | 3.9±1.2 |  |
|  | 243 |  |  | 2.6±1.4 |  |
|  | 253 |  |  | 1.9±1.2 |  |
|  | 263 |  |  | 3.2±1.1 |  |
|  | 273 |  |  | 3.5 |  |

**Comments:**

Four studies (Goldberg and Nuttall, 1978; Cohen et al., 1987; Yang et al., 2006; Park et al., 2009) measured the DRH of CaCl_2_. It was determined to be 28.3% at 298 K (Goldberg and Nuttall, 1978) and 29±2% at 298 K (Park et al., 2009), suggesting good agreement between the two studies. The third study (Cohen et al., 1987) found that CaCl_2_ displayed continuous hygroscopic growth at 296 K, and thus no DRH was reported. In the four study (Yang et al., 2006), the DRH of CaCl_2_ was determined to be 15±1% at 323 K.

The ERH of CaCl_2_ was only measured by one study (Gough et al., 2016). The measured ERH first decreased with temperature from 8.7±1.5% at 223 K to 1.9±1.2% at 253 K, then increased with temperature to 3.5% at 273 K.

**Preferred values at 298 K for CaCl_2_:**

DRH: 27-31%

ERH: no preferred value

**References:**

Cohen, M. D., Flagan, R. C., and Seinfeld, J. H.: Studies of concentrated electrolyte-solutions using the electrodynamic balance .1. Water activities for single-electrolyte solutions, Journal of Physical Chemistry, 91, 4563-4574, 1987.

Goldberg, R. N., and Nutall, R. L.: Evaluated activity and osmotic coefficients for aqueous solutions: The alkaline earth metal halides, Journal of Physical and Chemical Reference Data, 7, 263-310, 1978.

Gough, R. V., Chevrier, V. F., and Tolbert, M. A.: Formation of liquid water at low temperatures via the deliquescence of calcium chloride: Implications for Antarctica and Mars, Planetary and Space Science, 131, 79-87, 2016.

Park, K., Kim, J.-S., and Miller, A. L.: A study on effects of size and structure on hygroscopicity of nanoparticles using a tandem differential mobility analyzer and TEM, Journal of Nanoparticle Research, 11, 175-183, 2009.

Yang, L., Pabalan, R. T., and Juckett, M. R.: Deliquescence relative humidity measurements using an electrical conductivity method, Journal of Solution Chemistry, 35, 583-604, 2006.

## CaCl_2_∙2H_2_O, CaCl_2_∙4H_2_O and CaCl_2_∙6H_2_O (calcium chloride didehydrate, calcium chloride tetradehydrate and calcium chloride hexadehydrate)

| Species | Reference | *T* (K) | *D* | DRH (%) | ERH (%) | Techniques/Comments |
| --- | --- | --- | --- | --- | --- | --- |
| CaCl_2_∙2H_2_O | Pitzer and Shi, 1993 | 319 | - | 16 | - | Nonisopiestic method |
|  | Gough et al., 2016 | 223 | 5-20 μm | 19.5±4.9 | - | Micro-Raman |
|  |  | 233 |  | 20.9±7 |  |  |
|  |  | 243 |  | 14.3±2.6 |  |  |
|  |  | 253 |  | 15.2±2.6 |  |  |
|  |  | 263 |  | 12.7±0.4 |  |  |
|  |  | 273 |  | 12.9 |  |  |
|  | Guo et al., 2019 | 298 | - | ~18.5 | - | VSA |
| CaCl_2_∙4H_2_O | Pitzer and Shi, 1993 | 303 | - | 23 | - | Nonisopiestic method |
| CaCl_2_∙6H_2_O | Gough et al., 2016 | 223 | 5-20 μm | 80.2±4.2 | - | Micro-Raman |
|  |  | 233 |  | 64.8±6.7 |  |  |
|  |  | 243 |  | 56.4±6.0 |  |  |
|  |  | 253 |  | 51.7 |  |  |
|  | Guo et al., 2019 | 298 | - | ~28.5 | - | VSA |

**Comments:**

Three studies (Pitzer and Shi, 1993; Gough et al., 2016; Guo et al., 2019) measured the DRH of CaCl_2_∙2H_2_O. In the first study (Pitzer and Shi, 1993), it was measured to be 16% at 319 K; in the second study (Gough et al., 2016), it decreased with temperature from 19.5±4.9% at 223 K to 12.9% at 273 K; in the third study (Guo et al., 2019), it was measured to be ~18.5% at 298 K.

Pitzer and Shi (1993) measured the DRH of CaCl_2_∙4H_2_O, which was reported to be 23% at 303 K.

Two studies (Guo et al., 2019; Gough et al., 2016) measured the DRH of CaCl_2_∙6H_2_O. In the first study (Gough et al., 2016), it decreased with temperature 80.2±4.2% at 223 K to 51.7% at 253 K. In the second study (Guo et al., 2019), it was measured to be ~28.5% at 298 K.

**Preferred values at 298 K for CaCl_2_∙2H_2_O:**

DRH: 18-19%

ERH: no preferred value

**Preferred values at 298 K for CaCl_2_∙4H_2_O:**

DRH: 23%

ERH: no preferred value

**Preferred values at 298 K for CaCl_2_∙6H_2_O:**

DRH: 28-29%

ERH: no preferred value

**References:**

Gough, R. V., Chevrier, V. F., and Tolbert, M. A.: Formation of liquid water at low temperatures via the deliquescence of calcium chloride: Implications for Antarctica and Mars, Planetary and Space Science, 131, 79-87, 2016.

Guo, L., Gu, W., Peng, C., Wang, W., Li, Y. J., Zong, T., Tang, Y., Wu, Z., Lin, Q., Ge, M., Zhang, G., Hu, M., Bi, X., Wang, X., and Tang, M.: A comprehensive study of hygroscopic properties of calcium- and magnesium-containing salts: implication for hygroscopicity of mineral dust and sea salt aerosols, Atmospheric Chemistry and Physics, 19, 2115-2133, 2019.

Pitzer, K. S., and Shi, Y. W.: Thermodynamics of calcium chloride in highly concentrated aqueous solution and in hydrated crystals, Journal of Solution Chemistry, 22, 99-105, 1993.

## MgCl_2_ (magnesium chloride)

| Reference | *T* (K) | *D* | DRH (%) | ERH (%) | Techniques/Comments |
| --- | --- | --- | --- | --- | --- |
| Rockland, 1960 | 278 | - | 33 | - | Nonisopiestic method |
|  | 283 |  | 33 |  |  |
|  | 288 |  | 33 |  |  |
|  | 293 |  | 33 |  |  |
|  | 298 |  | 33 |  |  |
|  | 303 |  | 32 |  |  |
|  | 308 |  | 32 |  |  |
|  | 313 |  | 31 |  |  |
| Greenspan, 1977 | 273 | - | 33.7±0.3 | - | Nonisopiestic method |
|  | 278 |  | 33.6±0.3 |  |  |
|  | 283 |  | 33.5±0.2 |  |  |
|  | 288 |  | 33.3±0.2 |  |  |
|  | 293 |  | 33.1±0.2 |  |  |
|  | 298 |  | 32.8±0.2 |  |  |
|  | 303 |  | 32.4±0.1 |  |  |
|  | 308 |  | 32.1±0.1 |  |  |
|  | 313 |  | 31.6±0.1 |  |  |
|  | 318 |  | 31.1±0.1 |  |  |
|  | 323 |  | 30.5±0.4 |  |  |
|  | 328 |  | 29.9±0.2 |  |  |
|  | 333 |  | 29.3±0.2 |  |  |
|  | 338 |  | 28.5±0.2 |  |  |
|  | 343 |  | 27.8±0.3 |  |  |
|  | 348 |  | 26.9±0.3 |  |  |
|  | 353 |  | 26.1±0.3 |  |  |
|  | 358 |  | 25.1±0.4 |  |  |
|  | 363 |  | 24.1±0.5 |  |  |
|  | 368 |  | 23.1±0.5 |  |  |
|  | 373 |  | 22.0±0.6 |  |  |
| Park et al., 2009 | 298 | 20-50 nm | 33±2 | - | HTDMA |
| Arenas et al., 2012 | 298 | - | 30.7±0.9 | - | QCM |
| Schindelholz et al., 2014 | 294 | ~100 μm | 12-15 | <1.5 | Electrical conductivity/ impedance method |
| Chao et al., 2020 | 298 | - | 37±1 | - | QCM |

**Comments:**

Six studies (Rockland, 1960; Greenspan, 1977; Park et al., 2009; Arenas et al., 2012; Schindelholz et al., 2014; Chao et al., 2020) measured DRH of MgCl_2_ at around room temperature. The DRH at 298 K was measured to be 33% (Rockland, 1960), 32.8±0.2% (Greenspan, 1977), 33±2% (Park et al., 2009) and 30.7±0.9% (Arenas et al., 2012), showing good agreement. In addition, it was measured to be 37±1% at 298 K by Chao et al. (2020) and 12-15% at 294 K (Schindelholz et al., 2014), significantly different from those reported by the first four studies (Rockland, 1960; Greenspan, 1977; Park et al., 2009; Arenas et al., 2012).


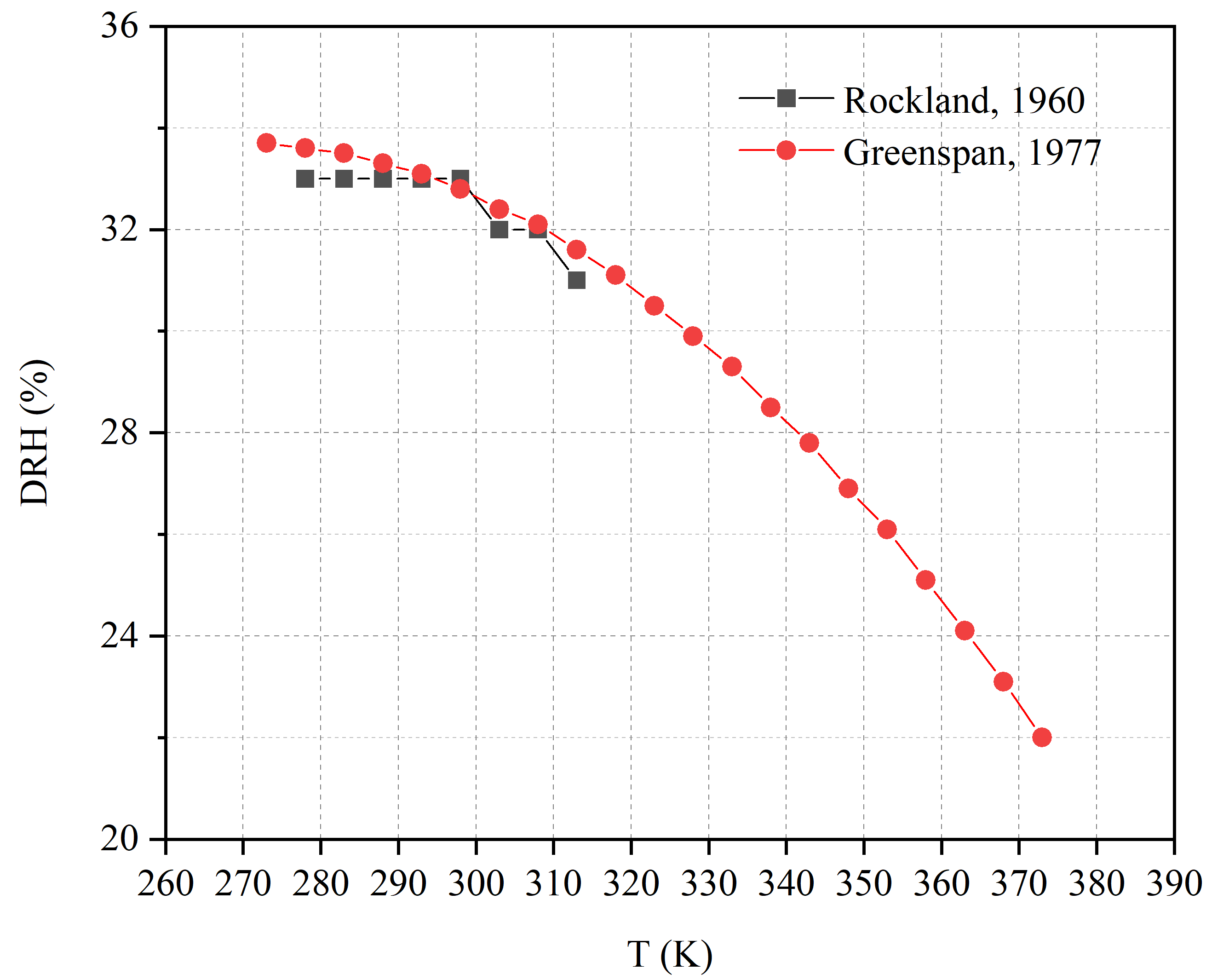


Two studies (Rockland, 1960; Greenspan, 1977) measured DRH of MgCl_2_ at different temperature, and revealed similar temperature dependence. To be more specific, Rockland (1960) found that it decreased with temperature from 33% at 278 K to 31% at 313 K, and Greenspan (1977) suggested that it decreased with temperature from 33.7±0.3% at 273 K to 22.0±0.6% at 373 K.

The ERH of MgCl_2_ was only measured by one study (Schindelholz et al., 2014), and it was determined to be <1.5 at 294 K.

**Preferred values at 298 K for MgCl_2_:**

DRH: 31-35%

ERH: <1.5%

**References:**

Arenas, K. J. L., Schill, S. R., Malla, A., and Hudson, P. K.: Deliquescence Phase Transition Measurements by Quartz Crystal Microbalance Frequency Shifts, Journal of Physical Chemistry A, 116, 7658-7667, 2012.

Chao, H.-J., Huang, W.-C., Chen, C.-L., Chou, C. C. K., and Hung, H.-M.: Water Adsorption vs Phase Transition of Aerosols Monitored by a Quartz Crystal Microbalance, Acs Omega, 5, 31858-31866, 2020.

Greenspan, L.: Humidity fixed-points of binary saturated aqueous-solutions, Journal of Research of the National Bureau of Standards Section a-Physics and Chemistry, 81, 89-96, 1977.

Park, K., Kim, J.-S., and Miller, A. L.: A study on effects of size and structure on hygroscopicity of nanoparticles using a tandem differential mobility analyzer and TEM, Journal of Nanoparticle Research, 11, 175-183, 2009.

Rockland, L. B.: Saturated Salt Solutions for Static Control of Relative Humidity between 5° and 40°C., Analytical Chemistry, 32, 1375-1376, 1960.

Schindelholz, E., Tsui, L.-k., and Kelly, R. G.: Hygroscopic Particle Behavior Studied by Interdigitated Array Microelectrode Impedance Sensors, Journal of Physical Chemistry A, 118, 167-177, 2014.

## MgCl_2_∙4H_2_O and MgCl_2_∙6H_2_O (magnesium chloride tetrahydrate and magnesium chloride hexahydrate)

| Species | Reference | *T* (K) | *D* | DRH (%) | ERH (%) | Techniques/Comments |
| --- | --- | --- | --- | --- | --- | --- |
| MgCl_2_∙4H_2_O | Gupta et al., 2015 | 296 | 1-10 μm | 15.5-16.5 | 6.8-7.2 | Micro-Raman |
| MgCl_2_∙6H_2_O | Stokes and Robinson, 1949 | 298 | - | 33.0 | - | Nonisopiestic method |
|  | Rard and Miller, 1981 | 298 | - | 32.8 | - | Isopiestic method |
|  | Gough et al., 2014 | 243 | 2-20 μm | - | 14±4 | Micro-Raman |
|  | Gupta et al., 2015 | 296 | 1-10 μm | 30.6-34.7 | 9.1-10.8 | Micro-Raman |
|  | Gu et al., 2017 | 278 | - | 33.3±1 | - | VSA |
|  |  | 283 |  | 33.9±1 |  |  |
|  |  | 288 |  | 33.6±1 |  |  |
|  |  | 293 |  | 33.5±1 |  |  |
|  |  | 298 |  | 33.2±1 |  |  |
|  |  | 303 |  | 33.6±1 |  |  |
|  | Guo et al., 2019 | 278 | - | 32.5±1.0 | - | VSA |
|  |  | 283 |  | 32.5±1.0 |  |  |
|  |  | 288 |  | 32.5±1.0 |  |  |
|  |  | 293 |  | 32.5±1.0 |  |  |
|  |  | 298 |  | 31.5±1.0 |  |  |
|  |  | 303 |  | 31.5±1.0 |  |  |

**Comments:**

The DRH and ERH of MgCl_2_∙4H_2_O were measured by Gupta et al. (2015) to be 15.5-16.5% and 6.8-7.2% at 296 K.

Five studies (Stokes and Robinson, 1949; Rard and Miller, 1981; Gupta et al., 2015; Gu et al., 2017; Guo et al., 2019) measured DRH of MgCl_2_∙6H_2_O. The DRH was measured to be 30-35% at 296-298 K, showing good agreement among these studies. In addition, two studies (Gu et al., 2017; Guo et al., 2019) investigated the dependence of DRH of MgCl_2_∙6H_2_O on temperature, and both work suggested that it did not change significantly with temperature between 278 and 303 K.

The ERH of MgCl_2_∙6H_2_O was measured by two studies (Gough et al., 2011; Gupta et al., 2015). The measured ERH was determined to be 14±4% at 243 K (Gough et al., 2011) and 9.1-10.8% at 296 K (Gupta et al., 2015).

**Preferred values at 298 K for MgCl_2_∙4H_2_O:**

DRH: 15-17%

ERH: 6-8%

**Preferred values at 298 K for MgCl_2_∙6H_2_O:**

DRH: 30-35%

ERH: 9-11%

**References:**

Gough, R. V., Chevrier, V. F., and Tolbert, M. A.: Formation of aqueous solutions on Mars via deliquescence of chloride-perchlorate binary mixtures, Earth and Planetary Science Letters, 393, 73-82, 2014.

Gu, W., Li, Y., Zhu, J., Jia, X., Lin, Q., Zhang, G., Ding, X., Song, W., Bi, X., Wang, X., and Tang, M.: Investigation of water adsorption and hygroscopicity of atmospherically relevant particles using a commercial vapor sorption analyzer, Atmospheric Measurement Techniques, 10, 3821-3832, 2017.

Guo, L., Gu, W., Peng, C., Wang, W., Li, Y. J., Zong, T., Tang, Y., Wu, Z., Lin, Q., Ge, M., Zhang, G., Hu, M., Bi, X., Wang, X., and Tang, M.: A comprehensive study of hygroscopic properties of calcium- and magnesium-containing salts: implication for hygroscopicity of mineral dust and sea salt aerosols, Atmospheric Chemistry and Physics, 19, 2115-2133, 2019.

Gupta, D., Eom, H. J., Cho, H. R., and Ro, C. U.: Hygroscopic behavior of NaCl–MgCl_2_ mixture particles as nascent sea-spray aerosol surrogates and observation of efflorescence during humidification, Atmospheric Chemistry and Physics, 15, 11273-11290, 2015.

Rard, J. A., and Miller, D. G.: Isopiestic determination of the osmotic and activity coefficients of aqueous magnesium chloride solutions at 25℃, Journal of Chemical and Engineering Data, 26, 38-43, 1981.

Stokes, R. H., and Robinson, R. A.: Standard Solutions for Humidity Control at 25°C., Industrial and Engineering Chemistry, 41, 2013-2013, 1949.

# Bromides

## NH_4_Br (ammonium bromide)

| Reference | *T* (K) | *D* | DRH (%) | ERH (%) | Techniques/Comments |
| --- | --- | --- | --- | --- | --- |
| Apelblat and Korin, 2003 | 278 | - | 81.5 | - | Nonisopiestic method |
|  | 283 |  | 81.2 |  |  |
|  | 288 |  | 80.9 |  |  |
|  | 293 |  | 80.6 |  |  |
|  | 298 |  | 80.3 |  |  |
|  | 303 |  | 80.0 |  |  |
|  | 308 |  | 79.7 |  |  |
|  | 313 |  | 79.4 |  |  |
|  | 318 |  | 79.0 |  |  |
|  | 323 |  | 78.7 |  |  |

**Comments:**

The nonisopiestic method was used to determine the DRH of NH_4_Br (Apelblat and Korin, 2003), which decreased with temperature from 81.5% at 278 K to 80.3% at 298 K and to 78.7% at 323 K.

**Preferred values at 298 K for NH_4_Br:**

DRH: 80%

ERH: no preferred value

**References:**

Apelblat, A., and Korin, E.: The molar enthalpies of solution and vapour pressures of saturated aqueous solutions of some ammonium salts, Journal of Chemical Thermodynamics, 35, 699-709, 2003.

## LiBr (lithium bromide) and LiBr∙2H_2_O (lithium bromide dihydrate)

| Species | Reference | *T* (K) | *D* | DRH (%) | ERH (%) | Techniques/Comments |
| --- | --- | --- | --- | --- | --- | --- |
| LiBr | Greenspan, 1977 | 273 | - | 7.8±0.8 | - | Nonisopiestic method |
|  |  | 278 |  | 7.4±0.8 |  |  |
|  |  | 283 |  | 7.1±0.7 |  |  |
|  |  | 288 |  | 6.9±0.6 |  |  |
|  |  | 293 |  | 6.6±0.6 |  |  |
|  |  | 298 |  | 6.4±0.5 |  |  |
|  |  | 303 |  | 6.2±0.5 |  |  |
|  |  | 308 |  | 6.0±0.4 |  |  |
|  |  | 313 |  | 5.8±0.4 |  |  |
|  |  | 318 |  | 5.7±0.4 |  |  |
|  |  | 323 |  | 5.5±0.3 |  |  |
|  |  | 328 |  | 5.4±0.3 |  |  |
|  |  | 333 |  | 5.3±0.3 |  |  |
|  |  | 338 |  | 5.3±0.2 |  |  |
|  |  | 343 |  | 5.2±0.2 |  |  |
|  |  | 348 |  | 5.2±0.2 |  |  |
|  |  | 353 |  | 5.2±0.2 |  |  |
|  |  | 358 |  | 5.2±0.2 |  |  |
|  |  | 363 |  | 5.3±0.2 |  |  |
|  |  | 368 |  | 5.3±0.2 |  |  |
|  |  | 373 |  | 5.4±0.2 |  |  |
| LiBr∙2H_2_O | Shpunt, 1968 | 291 | - | 7 | - | Nonisopiestic method |

**Comments:**

Greenspan (1977) used the nonisopiestic method to measure DRH of LiBr, which decreased with temperature from 7.8±0.8% at 273 K to 5.4±0.3% at 328 K, and showed no significant change with further increase in temperature to 373 K.

Shpunt (1968) used the nonisopiestic method to measure DRH of LiBr∙2H_2_O, and its DRH was reported to be 7% at 291 K.

**Preferred values at 298 K for LiBr:**

DRH: 6-7%

ERH: no preferred value

**Preferred values at 298 K for LiBr∙2H_2_O:**

DRH: 7%

ERH: no preferred value

**References:**

Greenspan, L.: Humidity fixed-points of binary saturated aqueous-solutions, Journal of Research of the National Bureau of Standards Section a-Physics and Chemistry, 81, 89-96, 1977.

Shpunt, A. A.: Hygroresistance (Hygroscopicity) of crystals, Measurement Techniques-Ussr, 1698-1700, 1968.

## NaBr (sodium bromide)

| Reference | *T* (K) | *D* | DRH (%) | ERH (%) | Techniques/Comments |
| --- | --- | --- | --- | --- | --- |
| Pearce and Nelson, 1932 | 298 | - | 56.5 | - | Nonisopiestic method |
| Rockland, 1960 | 278 | - | 59 | - | Nonisopiestic method |
|  | 283 |  | 58 |  |  |
|  | 288 |  | 58 |  |  |
|  | 293 |  | 57 |  |  |
|  | 298 |  | 57 |  |  |
|  | 303 |  | 57 |  |  |
|  | 308 |  | 57 |  |  |
|  | 313 |  | 57 |  |  |
| Greenspan, 1977 | 278 | - | 63.5±0.7 | - | Nonisopiestic method |
|  | 283 |  | 62.2±0.6 |  |  |
|  | 288 |  | 60.7±0.5 |  |  |
|  | 293 |  | 59.1±0.4 |  |  |
|  | 298 |  | 57.6±0.4 |  |  |
|  | 303 |  | 56.0±0.4 |  |  |
|  | 308 |  | 54.6±0.4 |  |  |
|  | 313 |  | 53.2±0.4 |  |  |
|  | 318 |  | 52.0±0.5 |  |  |
|  | 323 |  | 50.9±0.6 |  |  |
|  | 328 |  | 50.2±0.7 |  |  |
|  | 333 |  | 49.7±0.8 |  |  |
|  | 338 |  | 49.5±0.9 |  |  |
|  | 343 |  | 49.7±1.1 |  |  |
|  | 348 |  | 50.3±1.3 |  |  |
|  | 353 |  | 51.4±1.5 |  |  |
| Tang et al., 1977 | 298 | 0.3-2 μm | 57.7 | - | H-DMA-OPC |
| Cohen et al., 1987 | 293 | 20 μm | 44.5-45.5 | 22 | EDB |
| Apelblat, 1993 | 283 | - | 57.2 | - | Nonisopiestic method |
|  | 288 |  | 58.3 |  |  |
|  | 293 |  | 58.5 |  |  |
|  | 298 |  | 57.8 |  |  |
|  | 303 |  | 56.3 |  |  |
|  | 308 |  | 54.1 |  |  |
|  | 313 |  | 51.4 |  |  |
| Apelblat and Korin, 1998 | 278 | - | 58.6 | - | Nonisopiestic method |
|  | 283 |  | 58.8 |  |  |
|  | 288 |  | 58.9 |  |  |
|  | 293 |  | 58.8 |  |  |
|  | 298 |  | 58.6 |  |  |
|  | 303 |  | 58.3 |  |  |
|  | 308 |  | 57.9 |  |  |
|  | 313 |  | 57.3 |  |  |
|  | 318 |  | 56.6 |  |  |
|  | 323 |  | 55.9 |  |  |
| Wise et al., 2005 | 279 | 0.1-4 μm | 46-47 | 21-24 | ETEM |
| Miñambres et al., 2008 | 298 | 0.15-0.24 μm | 35-40 | 25-30 | FTIR |
| Ghorai and Tivanski, 2010 | 299 | ~1 μm | 45±4 | 22±1 | STXM |
| Miñambres et al., 2011 | 298 | 0.56 μm | 30-35 | - | FTIR |
| Beyer et al., 2014 | 277 | - | 63.2±1 | - | VSA |
|  | 298 | - | 57.3±1 |  |  |
| Gupta et al., 2014 | 295 | 1-10 μm | 48.5-49.0 | 27.0-28.6 | Optical microscopy |
| Schindelholz et al., 2014 | 294 | ~100 μm | 60 | 27-30 | electrical impedance method |
| Piens et al., 2016 | 298 | 0.56 μm | 47-49 | - | STXM |
| Estillore et al., 2017 | 298 | 100 nm | 45.8±0.7 | 17±2.2 | HTDMA |
| Gu et al., 2017 | 278 | - | 62.2±1.0 | - | VSA |
|  | 283 |  | 61.2±1.0 |  |  |
|  | 288 |  | 60.0±1.0 |  |  |
|  | 293 |  | 58.5±1.0 |  |  |
|  | 298 |  | 57.6±1.0 |  |  |
|  | 303 |  | 56.1±1.0 |  |  |

**Comments:**

A number of studies (Pearce and Nelson, 1932; Rockland, 1960; Greenspan, 1977; Tang et al., 1977; Apelblat, 1993; Apelblat and Korin, 1998; Beyer et al., 2014; Schindelholz et al., 2014; Gu et al., 2017) measured DRH of NaBr at around room temperature, and their measured DRH showed good agreement being 56-60% at 298 K. Several other studies (Cohen et al., 1987; Wise et al., 2005; Miñambres et al., 2008; Ghorai and Tivanski, 2010; Miñambres et al., 2011; Gupta et al., 2014; Piens et al., 2016; Estillore et al., 2017) also measured DRH of NaBr at 279-299 K, and their reported values (30-49%) were significantly lower.


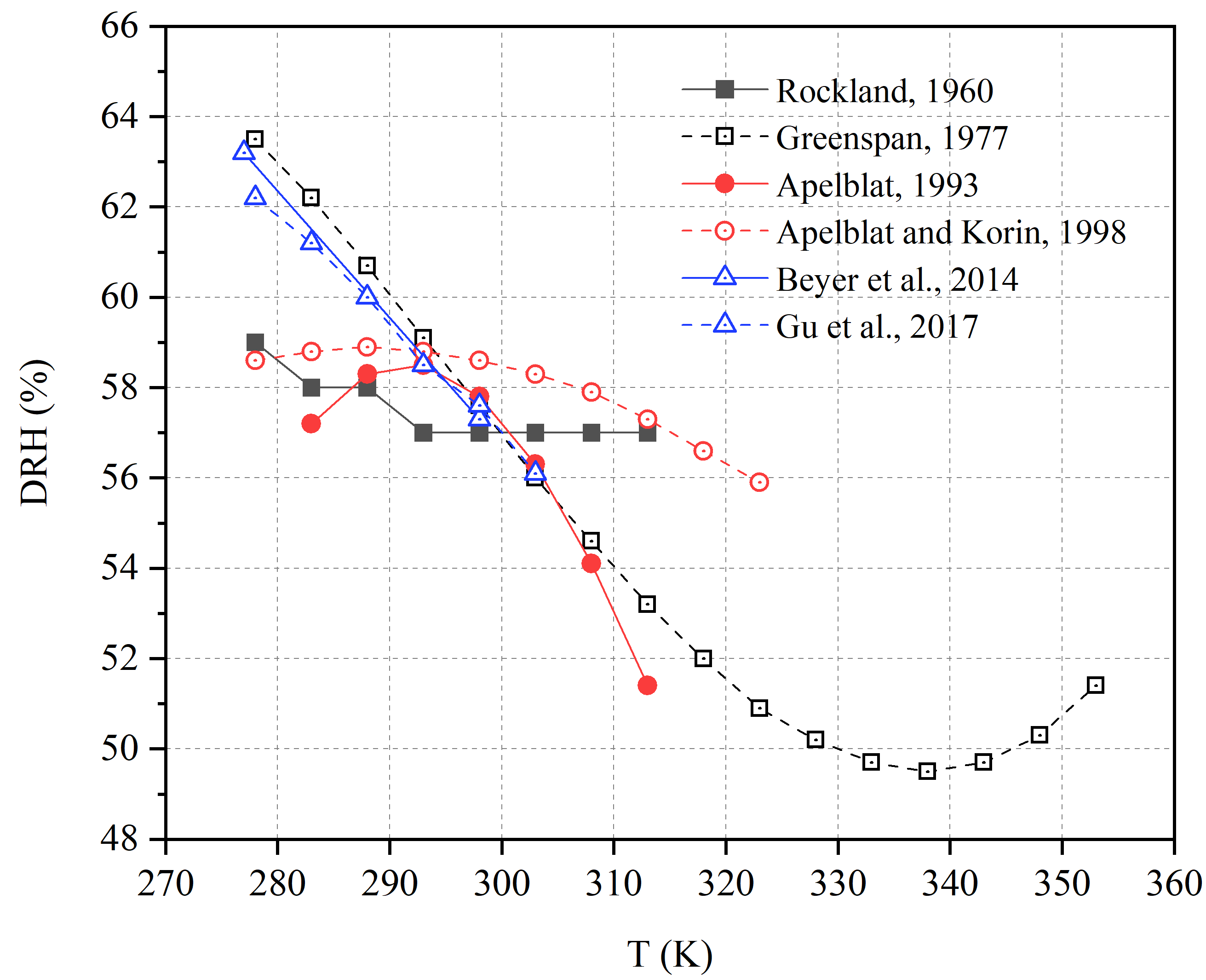


Six studies (Rockland, 1960; Greenspan, 1977; Apelblat, 1993; Apelblat and Korin, 1998; Beyer et al., 2014; Gu et al., 2017) measured DRH of NaBr at different RH. Greenspan suggested that it decreased with temperature from 63.5±0.7% at 278 K to 49.5±0.9% at 338 K, and then slightly increased with temperature to 51.4±1.5% at 353 K. Another two studies (Beyer et al., 2017; Gu et al., 2017) revealed similar temperature dependence: Beyer et al. (2017) found that it increase with temperature from 63.2% at 277 K and 57.3% at 298 K, and Gu et al. (2017) found that it increased with temperature from 62.2±1.0% at 278 K to 56.1±1.0% at 303 K. However, the other three studies (Rockland, 1960; Apelblat, 1993; Apelblat and Korin, 1998) reported different temperature dependence: Rockland (1960) suggested that the DRH of NaBr decreased slightly with temperature from 59% at 278 K to 57% at 313 K, Apelblat and Korin (1998) also suggested it slightly decreased with temperature from 58.6% at 278 K to 55.9% at 323 K, while Apelblat (1993) found it first increased with temperature from 57.2% at 283 K to 58.5% at 293 K and then decreased with temperature to 51.4% at 313 K.

The ERH values of NaBr at around room temperature were measured by several studies (Cohen et al., 1987; Wise et al., 2005; Miñambres et al., 2008; Ghorai and Tivanski, 2010; Gupta et al., 2014), and the measured ERH values, in the range of 21-30% at 279-299 K, showed relatively good agreement. Estillore et al. (2017) also measured the ERH of NaBr at 298 K; and the measured ERH (17±2.2%) was lower than those reported by other studies (Cohen et al., 1987; Wise et al., 2005; Miñambres et al., 2008; Ghorai and Tivanski, 2010; Gupta et al., 2014).

**Preferred values at 298 K for NaBr:**

DRH: 56-60%

ERH: 21-30%

**References:**

Apelblat, A.: The vapor-pressures of saturated aqueous lithium-chloride, sodium-bromide, sodium-nitrate, ammonium-nitrate, and ammonium-chloride at temperatures from 283 K to 313 K, Journal of Chemical Thermodynamics, 25, 63-71, 1993.

Apelblat, A., and Korin, E.: The vapour pressures of saturated aqueous solutions of sodium chloride, sodium bromide, sodium nitrate, sodium nitrite, potassium iodate, and rubidium chloride at temperatures from 227 K to 323 K, Journal of Chemical Thermodynamics, 30, 59-71, 1998.

Beyer, K. D., Schroeder, J. R., and Kissinger, J. A.: Temperature-Dependent Deliquescence Relative Humidities and Water Activities Using Humidity Controlled Thermogravimetric Analysis with Application to Malonic Acid, Journal of Physical Chemistry A, 118, 2488-2497, 2014.

Cohen, M. D., Flagan, R. C., and Seinfeld, J. H.: Studies of concentrated electrolyte-solutions using the electrodynamic balance .1. Water activities for single-electrolyte solutions, Journal of Physical Chemistry, 91, 4563-4574, 1987.

Cohen, M. D., Flagan, R. C., and Seinfeld, J. H.: Studies of concentrated electrolyte solutions using the electrodynamic balance. 3. Solute nucleation, Journal of Physical Chemistry, 91, 4583-4590, 1987.

Estillore, A. D., Morris, H. S., Or, V. W., Lee, H. D., Alves, M. R., Marciano, M. A., Laskina, O., Qin, Z., Tivanski, A. V., and Grassian, V. H.: Linking hygroscopicity and the surface microstructure of model inorganic salts, simple and complex carbohydrates, and authentic sea spray aerosol particles, Physical Chemistry Chemical Physics, 19, 21101-21111, 2017.

Ghorai, S., and Tivanski, A. V.: Hygroscopic Behavior of Individual Submicrometer Particles Studied by X-ray Spectromicroscopy, Analytical Chemistry, 82, 9289-9298, 2010.

Greenspan, L.: Humidity fixed-points of binary saturated aqueous-solutions, Journal of Research of the National Bureau of Standards Section a-Physics and Chemistry, 81, 89-96, 1977.

Gu, W., Li, Y., Zhu, J., Jia, X., Lin, Q., Zhang, G., Ding, X., Song, W., Bi, X., Wang, X., and Tang, M.: Investigation of water adsorption and hygroscopicity of atmospherically relevant particles using a commercial vapor sorption analyzer, Atmospheric Measurement Techniques, 10, 3821-3832, 2017.

Gupta, D., Eom, H.-J., Li, X., Cho, H.-R., Park, G.-H., Lee, J.-S., Kim, H.-K., and Ro, C.-U.: Hygroscopic properties of pure and mixed halide particles as nascent sea salt aerosol surrogates, The 8th International Conference on Combustion, Incineration/Pyrolysis, Emission and Climate Change (i-CIPEC), Hangzhou, China, October 15-18, 2014.

Minambres, L., Sanchez, M. N., Castano, F., and Basterretxea, F. J.: Infrared spectroscopic properties of sodium bromide aerosols, Journal of Physical Chemistry A, 112, 6601-6608, 2008.

Minambres, L., Mendez, E., Sanchez, M. N., Castano, F., and Basterretxea, F. J.: Water uptake properties of internally mixed sodium halide and succinic acid particles, Atmospheric Environment, 45, 5896-5902, 2011.

Pearce, J. N., and Nelson, A. F.: The vapor pressures of aqueous solutions of lithium nitrate and the activity coefficients of some alkali salts in solutions of high concentration at 25 degrees, Journal of the American Chemical Society, 54, 3544-3555, 1932.

Piens, D. S., Kelly, S. T., Harder, T. H., Petters, M. D., O'Brien, R. E., Wang, B., Teske, K., Dowell, P., Laskin, A., and Gilles, M. K.: Measuring Mass-Based Hygroscopicity of Atmospheric Particles through in Situ Imaging, Environmental Science & Technology, 50, 5172-5180, 2016.

Rockland, L. B.: Saturated Salt Solutions for Static Control of Relative Humidity between 5° and 40° C., Analytical Chemistry, 32, 1375-1376, 1960.

Schindelholz, E., Tsui, L.-k., and Kelly, R. G.: Hygroscopic Particle Behavior Studied by Interdigitated Array Microelectrode Impedance Sensors, Journal of Physical Chemistry A, 118, 167-177, 2014.

Tang, I. N., Munkelwitz, H. R., and Davis, J. G.: Aerosol growth studies—II. Preparation and growth measurements of monodisperse salt aerosols, Journal of aerosol science, 8, 149-159, 1977.

Wise, M. E., Biskos, G., Martin, S. T., Russell, L. M., and Buseck, P. R.: Phase transitions of single salt particles studied using a transmission electron microscope with an environmental cell, Aerosol Science and Technology, 39, 849-856, 2005.

## NaBr∙2H_2_O (sodium bromide dihydrate)

| Species | Reference | *T* (K) | *D* | DRH (%) | ERH (%) | Techniques/Comments |
| --- | --- | --- | --- | --- | --- | --- |
| NaBr∙2H_2_O | Stokes and Robinson, 1949 | 298 | - | 57.7 | - | Nonisopiestic method |
|  | Shpunt, 1968 | 293 | - | 58 | - | Nonisopiestic method |
|  | Cohen et al., 1987 | 293 | 20 μm | 58 | - | EDB |

**Comments:**

Two studies (Stokes and Robinson, 1949; Shpunt, 1968) used the nonisopiestic method to measure DRH of NaBr∙2H_2_O, which were determined to be 57.7% at 298 K and 58% at 293 K, respectively. The DRH of NaBr∙2H_2_O was measured by Cohen et al. (1987) to be 58% at 293 K, agreeing well with those reported by the first two studies (Stokes and Robinson, 1949; Shpunt, 1968).

**Preferred values at 298 K for NaBr∙2H_2_O:**

DRH: 57-58%

ERH: no preferred value

**References:**

Cohen, M. D., Flagan, R. C., and Seinfeld, J. H.: Studies of concentrated electrolyte-solutions using the electrodynamic balance .1. Water activities for single-electrolyte solutions, Journal of Physical Chemistry, 91, 4563-4574, 1987.

Shpunt, A. A.: Hygroresistance (Hygroscopicity) of crystals, Measurement Techniques-Ussr, 1698-1700, 1968.

Stokes, R. H., and Robinson, R. A.: Standard Solutions for Humidity Control at 25°C., Industrial and Engineering Chemistry, 41, 2013-2013, 1949.

## KBr (potassium bromide)

| Reference | *T* (K) | *D* | DRH (%) | ERH (%) | Techniques/Comments |
| --- | --- | --- | --- | --- | --- |
| Stokes and Robinson, 1949 | 298 | - | 80.7 | - | Nonisopiestic method |
| Rockland, 1960 | 283 | - | 86 | - | Nonisopiestic method |
|  | 288 |  | 85 |  |  |
|  | 293 |  | 84 |  |  |
|  | 298 |  | 83 |  |  |
|  | 303 |  | 82 |  |  |
|  | 308 |  | 81 |  |  |
|  | 313 |  | 80 |  |  |
| Shpunt, 1968 | 293 | - | 81.7 | - | Nonisopiestic method |
| Greenspan, 1977 | 278 | - | 85.1±0.3 | - | Nonisopiestic method |
|  | 283 |  | 83.8±0.2 |  |  |
|  | 288 |  | 82.6±0.2 |  |  |
|  | 293 |  | 81.7±0.2 |  |  |
|  | 298 |  | 80.9±0.2 |  |  |
|  | 303 |  | 80.3±0.2 |  |  |
|  | 308 |  | 79.8±0.2 |  |  |
|  | 313 |  | 79.4±0.2 |  |  |
|  | 318 |  | 79.2±0.3 |  |  |
|  | 323 |  | 79.0±0.3 |  |  |
|  | 328 |  | 79.0±0.3 |  |  |
|  | 333 |  | 78.9±0.4 |  |  |
|  | 338 |  | 79.0±0.4 |  |  |
|  | 343 |  | 79.1±0.5 |  |  |
|  | 348 |  | 79.2±0.5 |  |  |
|  | 353 |  | 79.3±0.6 |  |  |
| Tang et al., 1977 | 298 | 0.3-2 μm | 80.8 | - | H-DMA-OPC |
| Cohen et al., 1987 | 293 | 20 μm | 81±1 | 52 | EDB |
| Wise et al., 2005 | 279 | 0.1-4 μm | 81-83 | 51-53 | ETEM |
| Gupta et al., 2014 | 295 | 1-10 μm | 79.7-80.9 | 54.7-55.3 | Optical microscopy |
| Giamarelou et al., 2018 | 298 | 8 | 89.7-91.1 | - | HTDMA |
|  |  | 10 | 86.9-89.6 | 61.0-67.6 |  |
|  |  | 15 | - | 62.6-65.0 |  |
|  |  | 20 | 80.7-81.7 | 58.0-62.1 |  |
|  |  | 30 | 79.7-80.9 | 57.8-59.7 |  |
|  |  | 40 | 77.5-81.1 | 57.9-60.0 |  |
|  |  | 60 | 72.4-78.1 | 55.3-56.2 |  |

**Comments:**

Several studies (Stokes and Robinson, 1949; Rockland, 1960; Shpunt, 1968; Greenspan, 1977; Tang et al., 1977; Cohen et al., 1987; Wise et al., 2005; Gupta et al., 2014) measured DRH of KBr at 279-298 K, and the measured DRH (80-83%) showed good agreement. In addition, Giamarelou et al. (2018) also measured the DRH of KBr at 298 K; however, their measured DRH (72.4-78.1% for 60 nm particles) was lower than those reported by other studies.


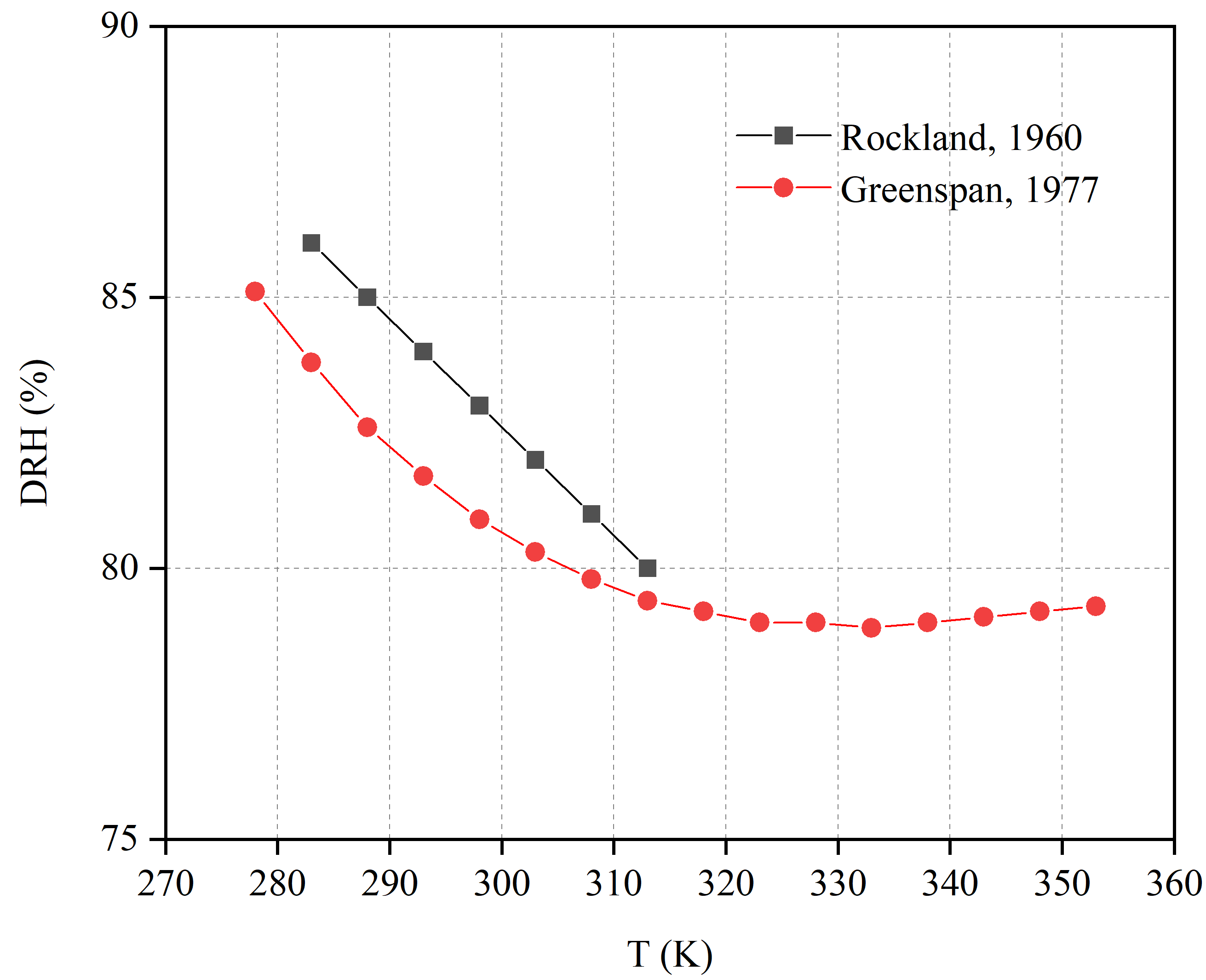


Two studies (Rockland, 1960; Greenspan, 1977) measured DRH of KBr at different RH. Rockland (1960) found that it decreased with temperature from 86% at 283 K to 80% at 313 K; similarly, Greenspan found it first decreased with temperature from 85.1±0.3% at 278 K to 79.4±0.2% at 313 K and then did not change with temperature when temperature further increased to 353 K.

The ERH values of KBr at 279-298 K were measured by four studies (Cohen et al., 1987; Wise et al., 2005; Gupta et al., 2014; Giamarelou et al., 2018), and their measured ERH values, in the range of 51-56%, showed good agreement.

**Preferred values at 298 K for KBr:**

DRH: 80-83%

ERH: 51-56%

**References:**

Cohen, M. D., Flagan, R. C., and Seinfeld, J. H.: Studies of concentrated electrolyte-solutions using the electrodynamic balance .1. Water activities for single-electrolyte solutions, Journal of Physical Chemistry, 91, 4563-4574, 1987.

Cohen, M. D., Flagan, R. C., and Seinfeld, J. H.: Studies of concentrated electrolyte solutions using the electrodynamic balance. 3. Solute nucleation, Journal of Physical Chemistry, 91, 4583-4590, 1987.

Giamarelou, M., Smith, M., Papapanagiotou, E., Martin, S. T., and Biskos, G.: Hygroscopic properties of potassium-halide nanoparticles, Aerosol Science and Technology, 52, 536-545, 2018.

Greenspan, L.: Humidity fixed-points of binary saturated aqueous-solutions, Journal of Research of the National Bureau of Standards Section a-Physics and Chemistry, 81, 89-96, 1977.

Gupta, D., Eom, H.-J., Li, X., Cho, H.-R., Park, G.-H., Lee, J.-S., Kim, H.-K., and Ro, C.-U.: Hygroscopic properties of pure and mixed halide particles as nascent sea salt aerosol surrogates, The 8th International Conference on Combustion, Incineration/Pyrolysis, Emission and Climate Change (i-CIPEC), Hangzhou, China, October 15-18, 2014.

Rockland, L. B.: Saturated Salt Solutions for Static Control of Relative Humidity between 5° and 40°C., Analytical Chemistry, 32, 1375-1376, 1960.

Shpunt, A. A.: Hygroresistance (Hygroscopicity) of crystals, Measurement Techniques-Ussr, 1698-1700, 1968.

Stokes, R. H., and Robinson, R. A.: Standard Solutions for Humidity Control at 25°C., Industrial and Engineering Chemistry, 41, 2013-2013, 1949.

Tang, I. N., Munkelwitz, H. R., and Davis, J. G.: Aerosol growth studies—II. Preparation and growth measurements of monodisperse salt aerosols, Journal of aerosol science, 8, 149-159, 1977.

Wise, M. E., Biskos, G., Martin, S. T., Russell, L. M., and Buseck, P. R.: Phase transitions of single salt particles studied using a transmission electron microscope with an environmental cell, Aerosol Science and Technology, 39, 849-856, 2005.

## CaBr_2_ (calcium bromide)

| Reference | *T* (K) | *D* | DRH (%) | ERH (%) | Techniques/Comments |
| --- | --- | --- | --- | --- | --- |
| Greenspan, 1977 | 283 | - | 21.6±0.5 | - | Nonisopiestic method |
|  | 288 |  | 20.2±0.5 |  |  |
|  | 293 |  | 18.5±0.5 |  |  |
|  | 298 |  | 16.5±0.2 |  |  |
| Goldberg and Nutall, 1978 | 298 | - | 14.3 | - | Nonisopiestic method |
| Apelblat and Korin, 2002 | 283 | - | 21.5 | - | Nonisopiestic method |
|  | 288 |  | 20.1 |  |  |
|  | 293 |  | 18.5 |  |  |
|  | 295 |  | 16.7 |  |  |
|  | 297 |  | 15.7 |  |  |
|  | 298 |  | 14.7-16.5 |  |  |
|  | 299 |  | 14.9 |  |  |
|  | 302 |  | 13.4 |  |  |
|  | 304 |  | 12.8 |  |  |
|  | 306 |  | 13.5 |  |  |
|  | 308 |  | 14.3 |  |  |
|  | 311 |  | 15.6 |  |  |
|  | 313 |  | 16.4 |  |  |
| Gu et al., 2017 | 278 | - | 22.9±1.0 | - | VSA |
|  | 283 |  | 21.6±1.0 |  |  |
|  | 288 |  | 20.0±1.0 |  |  |
|  | 293 |  | 18.0±1.0 |  |  |
|  | 298 |  | 17.1±1.0 |  |  |
|  | 303 |  | 17.7±1.0 |  |  |

**Comments:**

The DRH of CaBr_2_ was measured by four studies (Greenspan, 1977; Goldberg and Nutall, 1978; Apelblat and Korin, 2002; Gu et al., 2017), and it was determined to be 14-18% at 298 K, showing good agreement.


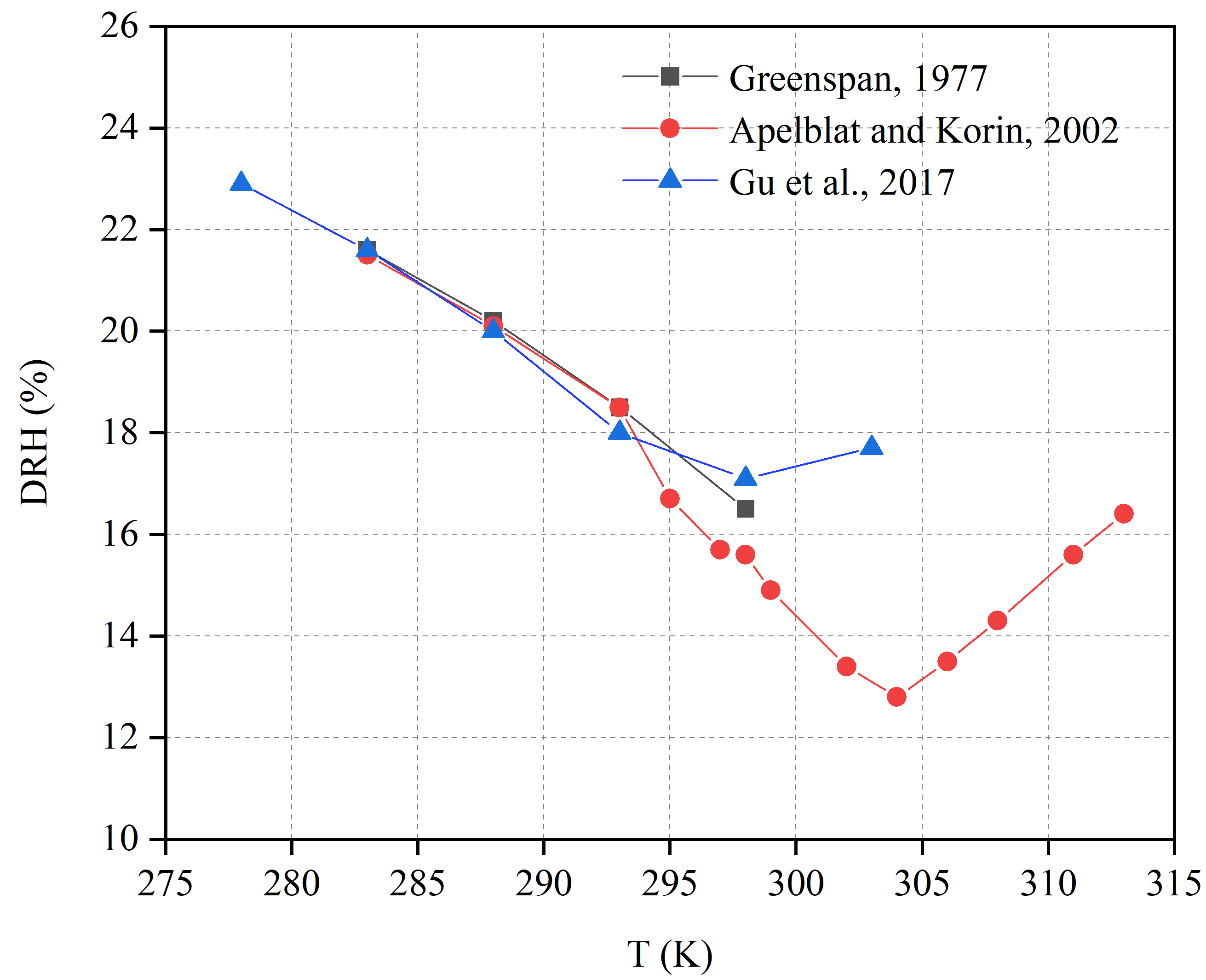


Three studies (Greenspan, 1977; Apelblat and Korin, 2002; Gu et al., 2017) examined the dependence of DRH of CaBr_2_ on temperature, and revealed similar temperature dependence at 278-304 K. To be more specific, Greenspan (1977) found that the DRH of CaBr_2_ decreased with temperature from 21.6±0.5% at 283 K to 16.5±0.2% at 298 K, Gu et al. (2017) found it decreased with temperature from 22.9±1.0% at 278 K to 17.7±1.0% at 303 K), and Apelblat and Korin (2002) suggested that it decreased with temperature from 21.5% at 283 K to 12.8% at 304 K and then increased with temperature to 16.4% at 313 K.

**Preferred values at 298 K for CaBr_2_:**

DRH: 14-18%

ERH: no preferred value

**References:**

Apelblat, A., and Korin, E.: The vapour pressure of water over saturated solutions of sodium sulfate, calcium bromide, ferric chloride, zinc nitrate, calcium nitrate, and lithium nitrate at temperatures from 278.15 K to 323.15 K, Journal of Chemical Thermodynamics, 34, 1621-1637, 2002.

Goldberg, R. N., and Nutall, R. L.: Evaluated activity and osmotic coefficients for aqueous solutions: The alkaline earth metal halides, Journal of Physical and Chemical Reference Data, 7, 263-310, 1978.

Greenspan, L.: Humidity fixed-points of binary saturated aqueous-solutions, Journal of Research of the National Bureau of Standards Section a-Physics and Chemistry, 81, 89-96, 1977.

Gu, W., Li, Y., Zhu, J., Jia, X., Lin, Q., Zhang, G., Ding, X., Song, W., Bi, X., Wang, X., and Tang, M.: Investigation of water adsorption and hygroscopicity of atmospherically relevant particles using a commercial vapor sorption analyzer, Atmospheric Measurement Techniques, 10, 3821-3832, 2017.

## MgBr_2_ (magnesium bromide)

| Reference | *T* (K) | *D* | DRH (%) | ERH (%) | Techniques/Comments |
| --- | --- | --- | --- | --- | --- |
| Rockland, 1960 | 278 | - | 32 | - | Nonisopiestic method |
|  | 283 |  | 31 |  |  |
|  | 288 |  | 31 |  |  |
|  | 293 |  | 31 |  |  |
|  | 298 |  | 31 |  |  |
|  | 303 |  | 30 |  |  |
|  | 308 |  | 30 |  |  |
|  | 313 |  | 30 |  |  |
| Goldberg and Nutall, 1978 | 298 | - | 31.0 | - | Nonisopiestic method |

**Comments:**

Rockland (1960) used the nonisopiestic method to measure DRH of MgBr_2_, which slightly decreased with temperature from 32% at 278 K to 30% at 313 K. The DRH of MgBr_2_ was measured to be 31.0% at 298 K by Goldberg and Nutall (1978), in good agreement with that (31% at 298 K) reported by Rockland (1960).

**Preferred values at 298 K for MgBr_2_:**

DRH: 31%

ERH: no preferred value

**References:**

Goldberg, R. N., and Nutall, R. L.: Evaluated activity and osmotic coefficients for aqueous solutions: The alkaline earth metal halides, Journal of Physical and Chemical Reference Data, 7, 263-310, 1978.

Rockland, L. B.: Saturated Salt Solutions for Static Control of Relative Humidity between 5° and 40°C., Analytical Chemistry, 32, 1375-1376, 1960.

# Iodides

## NH_4_I (ammonium iodide)

| Reference | *T* (K) | *D* | DRH (%) | ERH (%) | Techniques/Comments |
| --- | --- | --- | --- | --- | --- |
| Apelblat and Korin, 1998 | 278 | - | 69.1 | - | Nonisopiestic method |
|  | 283 |  | 70.9 |  |  |
|  | 288 |  | 72.2 |  |  |
|  | 293 |  | 72.9 |  |  |
|  | 298 |  | 73.0 |  |  |
|  | 303 |  | 72.6 |  |  |
|  | 308 |  | 71.8 |  |  |
|  | 313 |  | 70.5 |  |  |
|  | 318 |  | 68.9 |  |  |
|  | 323 |  | 66.9 |  |  |

**Comments:**

The nonisopiestic method was used to determine the DRH of NH_4_I at 278-323 K (Apelblat and Korin, 1998). The measured DRH increased with temperature from 69.1% at 278 K to 73.0% at 298 K, and then decreased with temperature to 66.9% at 323 K.

**Preferred values at 298 K for NH_4_I:**

DRH: 73%

ERH: no preferred value

**References:**

Apelblat, A., and Korin, E.: Vapour pressures of saturated aqueous solutions of ammonium iodide, potassium iodide, potassium nitrate, strontium chloride, lithium sulphate, sodium thiosulphate, magnesium nitrate, and uranyl nitrate from T = (278 to 323) K, Journal of Chemical Thermodynamics, 30, 459-471, 1998.

## LiI (lithium iodide) and LiI∙3H_2_O (lithium iodide trihydrate)

| Species | Reference | *T* (K) | *D* | DRH (%) | ERH (%) | Techniques/Comments |
| --- | --- | --- | --- | --- | --- | --- |
| LiI | Greenspan, 1977 | 278 | - | 21.7±0.3 | - | Nonisopiestic method |
|  |  | 283 |  | 20.6±0.3 |  |  |
|  |  | 288 |  | 19.6±0.2 |  |  |
|  |  | 293 |  | 18.6±0.2 |  |  |
|  |  | 298 |  | 17.6±0.1 |  |  |
|  |  | 303 |  | 16.6±0.1 |  |  |
|  |  | 308 |  | 15.6±0.1 |  |  |
|  |  | 313 |  | 14.6±0.1 |  |  |
|  |  | 318 |  | 13.5±0.1 |  |  |
|  |  | 323 |  | 12.4±0.1 |  |  |
|  |  | 328 |  | 11.2±0.1 |  |  |
|  |  | 333 |  | 10.0±0.1 |  |  |
|  |  | 338 |  | 8.7±0.1 |  |  |
|  |  | 343 |  | 7.2±0.1 |  |  |
| LiI∙3H_2_O | Shpunt, 1968 | 291 | - | 19 | - | Nonisopiestic method |

**Comments:**

The nonisopiestic method was used to measure DRH of LiI from 278 to 343 K (Greenspan, 1977). The measured DRH decreased with temperature, from 21.7±0.3% at 278 K to 7.2±0.1% at 343 K.

Shpunt (1968) used the nonisopiestic method to measure DRH of lithium iodide trihydrates (LiI∙3H_2_O) at 291 K, and its DRH was reported to be 19%.

**Preferred values at 298 K for NaI:**

DRH: 17-18%

ERH: no preferred value

**Preferred values at 298 K for LiI∙3H_2_O:**

DRH: 19%

ERH: no preferred value

**References:**

Greenspan, L.: Humidity fixed-points of binary saturated aqueous-solutions, Journal of Research of the National Bureau of Standards Section a-Physics and Chemistry, 81, 89-96, 1977.

Shpunt, A. A.: Hygroresistance (Hygroscopicity) of crystals, Measurement Techniques-Ussr, 1698-1700, 1968.

## NaI (sodium iodide) and NaI∙2H_2_O (sodium iodide dihydrate)

| Species | Reference | *T* (K) | *D* | DRH (%) | ERH (%) | Techniques/Comments |
| --- | --- | --- | --- | --- | --- | --- |
| NaI | Greenspan, 1977 | 278 | - | 42.4±1.0 | - | Nonisopiestic method |
|  |  | 283 |  | 41.8±0.8 |  |  |
|  |  | 288 |  | 40.9±0.7 |  |  |
|  |  | 293 |  | 39.7±0.6 |  |  |
|  |  | 298 |  | 38.2±0.5 |  |  |
|  |  | 303 |  | 36.2±0.4 |  |  |
|  |  | 308 |  | 34.7±0.4 |  |  |
|  |  | 313 |  | 32.9±0.4 |  |  |
|  |  | 318 |  | 31.0±0.4 |  |  |
|  |  | 323 |  | 29.2±0.4 |  |  |
|  |  | 328 |  | 27.5±0.5 |  |  |
|  |  | 333 |  | 26.0±0.5 |  |  |
|  |  | 338 |  | 24.6±0.6 |  |  |
|  |  | 343 |  | 23.6±0.7 |  |  |
|  |  | 348 |  | 22.9±0.9 |  |  |
|  |  | 353 |  | 22.5±1.0 |  |  |
|  |  | 358 |  | 22.6±1.2 |  |  |
|  |  | 363 |  | 23.3±1.4 |  |  |
|  | Tang et al., 1977 | 298 | 0.3-2 μm | 37.8 | - | H-DMA-OPC |
|  | Ghorai and Tivanski, 2010 | 299 | ~1 μm | 19±4 | 10±1 | STXM |
|  | Gupta et al., 2014 | 295 | 1-10 μm | 24.6-25.2 | 8.4-8.7 | Optical microscopy |
| NaI∙2H_2_O | Shpunt, 1968 | 293 | - | 39.2 | - | Nonisopiestic method |

**Comments:**

Greenspan (1977) used the nonisopiestic method to measure DRH of NaI, which decreased with temperature from 42.4±1.0% at 278 K to 23.3±1.4% at 363 K. The DRH of NaI was measured to be 37.8% at 298 K by the second study (Tang et al., 1977), in good agreement with that (38.2±0.5% at 298 K) reported by Greenspan (1977). The DRH values at 295-299 K, reported by the other two studies (Ghorai and Tivanski, 2010; Gupta et al., 2014), were significantly lower than those reported by the first two studies (Greenspan, 1977; Tang et al., 1977).

The ERH values of NaI were measured to be 10±1% at 299 K by Ghorai and Tivanski (2010) and 8.4-8.7% at 295 K by Gupta et al. (2014), showing good agreement between the two studies.

The DRH of NaI∙2H_2_O was measured to be 39.2% at 293 K, using the nonisopiestic method (Shpunt, 1968).

**Preferred values at 298 K for NaI:**

DRH: 37-39%

ERH: 8-11%

**Preferred values at 298 K for NaI∙2H_2_O:**

DRH: 39%

ERH: no preferred value

**References:**

Ghorai, S., and Tivanski, A. V.: Hygroscopic Behavior of Individual Submicrometer Particles Studied by X-ray Spectromicroscopy, Analytical Chemistry, 82, 9289-9298, 2010.

Greenspan, L.: Humidity fixed-points of binary saturated aqueous-solutions, Journal of Research of the National Bureau of Standards Section a-Physics and Chemistry, 81, 89-96, 1977.

Gupta, D., Eom, H.-J., Li, X., Cho, H.-R., Park, G.-H., Lee, J.-S., Kim, H.-K., and Ro, C.-U.: Hygroscopic properties of pure and mixed halide particles as nascent sea salt aerosol surrogates, The 8th International Conference on Combustion, Incineration/Pyrolysis, Emission and Climate Change (i-CIPEC), Hangzhou, China, October 15-18, 2014.

Shpunt, A. A.: Hygroresistance (Hygroscopicity) of crystals, Measurement Techniques-Ussr, 1698-1700, 1968.

Tang, I. N., Munkelwitz, H. R., and Davis, J. G.: Aerosol growth studies—II. Preparation and growth measurements of monodisperse salt aerosols, Journal of aerosol science, 8, 149-159, 1977.

## KI (potassium iodide)

| Reference | *T* (K) | *D* | DRH (%) | ERH (%) | Techniques/Comments |
| --- | --- | --- | --- | --- | --- |
| Pearce and Nelson, 1932 | 298 | - | 68.9 | - | Nonisopiestic method |
| Shpunt, 1968 | 293 | - | 69.9 | - | Nonisopiestic method |
| Greenspan, 1977 | 278 | - | 73.3±0.3 | - | Nonisopiestic method |
|  | 283 |  | 72.1±0.3 |  |  |
|  | 288 |  | 71.0±0.3 |  |  |
|  | 293 |  | 69.9±0.3 |  |  |
|  | 298 |  | 68.9±0.2 |  |  |
|  | 303 |  | 67.9±0.2 |  |  |
|  | 308 |  | 67.0±0.2 |  |  |
|  | 313 |  | 66.1±0.2 |  |  |
|  | 318 |  | 65.3±0.2 |  |  |
|  | 323 |  | 64.5±0.3 |  |  |
|  | 328 |  | 63.8±0.3 |  |  |
|  | 333 |  | 63.1±0.3 |  |  |
|  | 338 |  | 62.5±0.3 |  |  |
|  | 343 |  | 61.9±0.4 |  |  |
|  | 348 |  | 61.4±0.4 |  |  |
|  | 353 |  | 61.0±0.5 |  |  |
|  | 358 |  | 60.6±0.5 |  |  |
|  | 363 |  | 60.2±0.6 |  |  |
| Tang et al., 1977 | 298 | 0.3-2 μm | 68.8 | - | H-DMA-OPC |
| Apelblat and Korin, 1998 | 278 | - | 75.8 | - | Nonisopiestic method |
|  | 283 |  | 77.2 |  |  |
|  | 288 |  | 78.1 |  |  |
|  | 293 |  | 78.6 |  |  |
|  | 298 |  | 78.6 |  |  |
|  | 303 |  | 78.2 |  |  |
|  | 308 |  | 77.5 |  |  |
|  | 313 |  | 76.4 |  |  |
|  | 318 |  | 75.0 |  |  |
|  | 323 |  | 73.4 |  |  |
| Woods et al., 2007 | 298 | ~100 nm | 67±2 | 38±2 | HTDMA |
| Arenas et al., 2012 | 298 | - | 69.2±1.2 | - | QCM |
| Gupta et al., 2014 | 295 | 1-10 μm | 68.8-69.3 | 40.9-41.0 | Optical microscopy |
| Giamarelou et al., 2018 | 298 | 15 nm | 58.4-64.9 | 48.6-63.7 | HTDMA |
|  |  | 20 nm | 57.1-63.9 | 43.1-57.1 |  |
|  |  | 30 nm | 54.6-64.4 | 41.9-55.6 |  |
|  |  | 60 nm | 53.6-64.3 | 40.6-49.9 |  |

**Comments:**

The nonisopiestic method was used by Greenspan (1977) to measure DRH of KI, and it decreased with temperature from 73.3±0.3% at 278 K to 60.2±0.6% at 363 K. The DRH of KI at around room temperature, measured by several other studies (Pearce and Nelson, 1932; Shpunt, 1968; Tang et al., 1977; Woods et al., 2007; Arenas et al., 2012; Gupta et al., 2014; Giamarelou et al., 2018), agreed well with that reported by Greenspan (1977). Apelblat and Korin (1998) also measured the DRH of KI at different temperatures (278-323); however, their measured DRH (78.6%) at 298 K was significantly higher than those reported by other studies (65-70% at 298 K).

The ERH values of KI at around room temperature were measured by three studies (Woods et al., 2007; Gupta et al., 2014; Giamarelou et al., 2018), and the measured ERH values showed relatively good agreement, being in the range of 36-50% at 295-298 K.

**Preferred values at 298 K for KI:**

DRH: 65-70%

ERH: 36-50%

**References:**

Apelblat, A., and Korin, E.: Vapour pressures of saturated aqueous solutions of ammonium iodide, potassium iodide, potassium nitrate, strontium chloride, lithium sulphate, sodium thiosulphate, magnesium nitrate, and uranyl nitrate from T = (278 to 323) K, Journal of Chemical Thermodynamics, 30, 459-471, 1998.

Arenas, K. J. L., Schill, S. R., Malla, A., and Hudson, P. K.: Deliquescence Phase Transition Measurements by Quartz Crystal Microbalance Frequency Shifts, Journal of Physical Chemistry A, 116, 7658-7667, 2012.

Giamarelou, M., Smith, M., Papapanagiotou, E., Martin, S. T., and Biskos, G.: Hygroscopic properties of potassium-halide nanoparticles, Aerosol Science and Technology, 52, 536-545, 2018.

Greenspan, L.: Humidity fixed-points of binary saturated aqueous-solutions, Journal of Research of the National Bureau of Standards Section a-Physics and Chemistry, 81, 89-96, 1977.

Gupta, D., Eom, H.-J., Li, X., Cho, H.-R., Park, G.-H., Lee, J.-S., Kim, H.-K., and Ro, C.-U.: Hygroscopic properties of pure and mixed halide particles as nascent sea salt aerosol surrogates, The 8th International Conference on Combustion, Incineration/Pyrolysis, Emission and Climate Change (i-CIPEC), Hangzhou, China, October 15-18, 2014.

Pearce, J. N., and Nelson, A. F.: The vapor pressures of aqueous solutions of lithium nitrate and the activity coefficients of some alkali salts in solutions of high concentration at 25 degrees, Journal of the American Chemical Society, 54, 3544-3555, 1932.

Shpunt, A. A.: Hygroresistance (Hygroscopicity) of crystals, Measurement Techniques-Ussr, 1698-1700, 1968.

Tang, I. N., Munkelwitz, H. R., and Davis, J. G.: Aerosol growth studies—II. Preparation and growth measurements of monodisperse salt aerosols, Journal of aerosol science, 8, 149-159, 1977.

Woods, E., III, Kim, H. S., Wivagg, C. N., Dotson, S. J., Broekhuizen, K. E., and Frohardt, E. F.: Phase transitions and surface morphology of surfactant-coated aerosol particles, Journal of Physical Chemistry A, 111, 11013-11020, 2007.

# Chlorates

## NaClO_3_ (sodium chlorate)

| Reference | *T* (K) | *D* | DRH (%) | ERH (%) | Techniques/Comments |
| --- | --- | --- | --- | --- | --- |
| Apelblat and Manzurola, 2007 | 288 | - | 75.6 | - | Nonisopiestic method |
|  | 293 |  | 74.1 |  |  |
|  | 298 |  | 72.9 |  |  |
|  | 303 |  | 71.9 |  |  |
| Toner and Catling, 2018 | 298 | - | 73.3 | - | Nonisopiestic method |

**Comments:**

The DRH of NaClO_3_ was measured by two studies (Apelblat and Manzurola, 2007; Toner and Catling, 2018). It was determined to be 72-74% at 298 K, showing good agreement. Moreover, Apelblat and Manzurola (2007) measured its DRH at different temperature, and found that it decreased with temperature from 75.6% at 288 K to 71.9% at 303 K.

**Preferred values at 298 K for NaClO_3_:**

DRH: 72-74%

ERH: no preferred value

**References:**

Apelblat, A., and Manzurola, E.: The vapour pressures over saturated aqueous solutions of sodium and potassium acetates, chlorates, and perchlorates, Journal of Chemical Thermodynamics, 39, 1176-1181, 2007.

Toner, J. D., and Catling, D. C.: Chlorate brines on Mars: Implications for the occurrence of liquid water and deliquescence, Earth and Planetary Science Letters, 497, 161-168, 2018.

## KClO_3_ (potassium chlorate)

| Reference | *T* (K) | *D* | DRH (%) | ERH (%) | Techniques/Comments |
| --- | --- | --- | --- | --- | --- |
| Apelblat and Manzurola, 2007 | 298 | - | 97.7 | - | Nonisopiestic method |
|  | 303 |  | 97.3 |  |  |
|  | 308 |  | 96.8 |  |  |
|  | 313 |  | 96.4 |  |  |
|  | 318 |  | 95.8 |  |  |
|  | 323 |  | 95.3 |  |  |
|  | 328 |  | 94.7 |  |  |
|  | 333 |  | 94.1 |  |  |
|  | 338 |  | 93.4 |  |  |
|  | 343 |  | 92.7 |  |  |

**Comments:**

One study (Apelblat and Manzurola, 2007) measured the DRH of KClO_3_ as a function of temperature, and found that it decreased with temperature from 97.7% at 298 K to 92.7% at 343 K.

**Preferred values at 298 K for KClO_3_:**

DRH: 97-98%

ERH: no preferred value

**References:**

Apelblat, A., and Manzurola, E.: The vapour pressures over saturated aqueous solutions of sodium and potassium acetates, chlorates, and perchlorates, Journal of Chemical Thermodynamics, 39, 1176-1181, 2007.

## Ca(ClO_3_)_2_∙2H_2_O (calcium chlorate dihydrate) and Mg(ClO_3_)_2_∙6H_2_O (magnesium chlorate hexahydrate)

| Species | Reference | *T* (K) | *D* | DRH (%) | ERH (%) | Techniques/Comments |
| --- | --- | --- | --- | --- | --- | --- |
| Ca(ClO_3_)_2_∙2H_2_O | Toner and Catling, 2018 | 298 | - | 26.9 | - | Nonisopiestic method |
| Mg(ClO_3_)_2_∙6H_2_O | Toner and Catling, 2018 | 298 | - | 20.4 | - | Nonisopiestic method |

**Comments:**

The DRH values at 298 K were determined to be 26.9% for Ca(ClO_3_)_2_∙2H_2_O and 20.4% for Mg(ClO_3_)_2_∙6H_2_O (Toner and Catling, 2018).

**Preferred values at 298 K for Ca(ClO_3_)_2_∙2H_2_O:**

DRH: 26-27%

ERH: no preferred value

**Preferred values at 298 K for Mg(ClO_3_)_2_∙6H_2_O:**

DRH: 20-21%

ERH: no preferred value

**References:**

Toner, J. D., and Catling, D. C.: Chlorate brines on Mars: Implications for the occurrence of liquid water and deliquescence, Earth and Planetary Science Letters, 497, 161-168, 2018.

# Iodates

## I_2_O_5_ (iodic anhydride)

| Reference | *T* (K) | *D* | DRH (%) | ERH (%) | Techniques/Comments |
| --- | --- | --- | --- | --- | --- |
| Kumar et al., 2010 | 293 | - | 80±1 | - | Nonisopiestic method |
|  | 273 | 10-50 μm | 84.5±1 | n. o. | Optical microscopy |
|  | 278 |  | 83.3±1 |  |  |
|  | 283 |  | 82.7±1 |  |  |
|  | 288 |  | 81.2±1 |  |  |
|  | 293 |  | 80.8±1 |  |  |
|  | 298 |  | 79.5±1 |  |  |
|  | 303 |  | 78.3±1 |  |  |

**Comments:**

Kumar et al. (2010) used two methods to measure the DRH of I_2_O_5_. It was measured to be 80±1% and 79.5±1% using the nonisopiestic method and optical microscopy, respectively. Moreover, they found that the measured DRH decreased with temperature from 84.5±1% at 273 K to 78.3±1% at 303 K.

Kumar et al. (2010) found that the decrease in RH did not lead to crystallisation of I_2_O_5_, and thus no ERH was reported.

**Preferred values at 298 K for I_2_O_5_:**

DRH: 78-81%

ERH: no preferred value

**References:**

Kumar, R., Saunders, R. W., Mahajan, A. S., Plane, J. M. C., and Murray, B. J.: Physical properties of iodate solutions and the deliquescence of crystalline I_2_O_5_ and HIO_3_, Atmos. Chem. Phys., 10, 12251-12260, 2010.

## HIO_3_ (iodic acid)

| Reference | *T* (K) | *D* | DRH (%) | ERH (%) | Techniques/Comments |
| --- | --- | --- | --- | --- | --- |
| Kumar et al., 2010 | 293 | - | 84±1 | - | Nonisopiestic method |
|  | 273 | 10-50 μm | 90.9±1 | n. o. | Optical microscopy |
|  | 278 |  | 89.4±1 |  |  |
|  | 283 |  | 87.4±1 |  |  |
|  | 288 |  | 86.7±1 |  |  |
|  | 293 |  | 85.0±1 |  |  |
|  | 298 |  | 83.5±1 |  |  |
|  | 303 |  | 81.1±1 |  |  |
| Murray et al., 2012 | 294 | ~100 μm | 83.5±2 | n. o. | Micro-Raman |
|  |  | ~5 μm | - | n. o. | EDB |

**Comments:**

Two studies (Kumar et al., 2010; Murray et al., 2012) measured the DRH of HIO_3_. It was determined to be 83.5±1% at 298 K (Kumar et al., 2010) and 83.5±2% at 294 K (Murray et al., 2012), showing good agreement. Moreover, Kumar et al. (2010) also measured the DRH of HIO_3_ at different temperature, and found that it decreased with temperature from 90.9±1% at 273 K to 81.1±1% at 303 K.

Kumar et al. (2010) and Murray et al. (2012) observed gradual water evaporation for HIO_3_ particles with the decrease in RH, and thus no ERH was reported.

**Preferred values at 298 K for HIO_3_:**

DRH: 82-85%

ERH: no preferred value

**References:**

Kumar, R., Saunders, R. W., Mahajan, A. S., Plane, J. M. C., and Murray, B. J.: Physical properties of iodate solutions and the deliquescence of crystalline I_2_O_5_ and HIO_3_, Atmos. Chem. Phys., 10, 12251-12260, 2010.

Murray, B. J., Haddrell, A. E., Peppe, S., Davies, J. F., Reid, J. P., O'Sullivan, D., Price, H. C., Kumar, R., Saunders, R. W., Plane, J. M. C., Umo, N. S., and Wilson, T. W.: Glass formation and unusual hygroscopic growth of iodic acid solution droplets with relevance for iodine mediated particle formation in the marine boundary layer, Atmospheric Chemistry and Physics, 12, 8575-8587, 2012.

## KIO_3_ (potassium iodate)

| Reference | *T* (K) | *D* | DRH (%) | ERH (%) | Techniques/Comments |
| --- | --- | --- | --- | --- | --- |
| Apelblat and Korin, 1998 | 278 | - | 86.7 | - | Nonisopiestic method |
|  | 283 |  | 89.6 |  |  |
|  | 288 |  | 91.7 |  |  |
|  | 293 |  | 93.1 |  |  |
|  | 298 |  | 93.7 |  |  |
|  | 303 |  | 93.7 |  |  |
|  | 308 |  | 93.1 |  |  |
|  | 313 |  | 91.8 |  |  |
|  | 318 |  | 90.0 |  |  |

**Comments:**

Apelblat and Korin (1998) measured the DRH of KIO_3_ as a function of temperature. They found that it first increased with temperature from 86.7% at 278 K to 93.7% at 298 K, and then decreased with temperature to 90.0% at 318 K.

**Preferred values at 298 K for KIO_3_:**

DRH: 93-94%

ERH: no preferred value

**References:**

Apelblat, A., and Korin, E.: The vapour pressures of saturated aqueous solutions of sodium chloride, sodium bromide, sodium nitrate, sodium nitrite, potassium iodate, and rubidium chloride at temperatures from 227 K to 323 K, Journal of Chemical Thermodynamics, 30, 59-71, 1998.

# Perchlorates

## NH_4_ClO_4_ (ammonium perchlorate)

| Reference | *T* (K) | *D* | DRH (%) | ERH (%) | Techniques/Comments |
| --- | --- | --- | --- | --- | --- |
| Tereshchenko, 2020 | 298 | - | 94.4 | - | Isopiestic method |

**Comments:**

The DRH of NH_4_ClO_4_ was determined to be 94.4% at 298 K by Tereshchenko (2020).

**Preferred values at 298 K for NH_4_ClO_4_:**

DRH: 94-95%

ERH: no preferred value

**References:**

Tereshchenko, A. G.: Dynamic Method for the Determination of Hygroscopicity of Water-Soluble Solids, Journal of Solution Chemistry, 49, 1029-1051, 2020.

## LiClO_4_ (lithium perchlorate) and LiClO_4_∙3H_2_O (lithium perchlorate trihydrate)

| Species | Reference | *T* (K) | *D* | DRH (%) | ERH (%) | Techniques/Comments |
| --- | --- | --- | --- | --- | --- | --- |
| LiClO_4_ | Zhang and Chan, 2003 | 298 | - | - | 3 | EDB |
| LiClO_4_∙3H_2_O | Tang et al., 1995 | 298 | 14-16 μm | 67 | ~40 | EDB |

**Comments:**

The ERH of LiClO_4_ was measured to be 3% at 298 K by Zhang and Chan (2003).

The DRH and ERH values of LiClO_4_∙3H_2_O were determined to be 67% and ~40% at 298 K by Tang et al. (1995).

**Preferred values at 298 K for LiClO_4_:**

DRH: no preferred value

ERH: 3%

**Preferred values at 298 K for LiClO_4_∙3H_2_O:**

DRH: 67%

ERH: 40%

**References:**

Tang, I. N., Fung, K. H., Imre, D. G., and Munkelwitz, H. R.: Phase Transformation and Metastability of Hygroscopic Microparticles, Aerosol Science and Technology, 23, 443-453, 1995.

Zhang, Y. H., and Chan, C. K.: Observations of water monomers in supersaturated NaClO_4_, LiClO_4_, and Mg(ClO_4_)_2_ droplets using Raman spectroscopy, Journal of Physical Chemistry A, 107, 5956-5962, 2003.

## NaClO_4_ (sodium perchlorate)

| Reference | *T* (K) | *D* | DRH (%) | ERH (%) | Techniques/Comments |
| --- | --- | --- | --- | --- | --- |
| Zhang and Chan, 2003 | 298 | - | - | 11 | EDB |
| Zhang et al., 2005 | 298 | 5-20 μm | 46±2.5 | 20±2.5 | ATR-FTIR |
| Zhao et al., 2005 | 298 | 1-5 μm | 43±2.5 | 18±2.5 | FTIR |
| Apelblat and Manzurola, 2007 | 278 | - | 50.2 | - | Nonisopiestic method |
|  | 283 |  | 49.1 |  |  |
|  | 288 |  | 47.7 |  |  |
|  | 293 |  | 46.2 |  |  |
|  | 298 |  | 44.6 |  |  |
|  | 303 |  | 42.8 |  |  |
|  | 308 |  | 40.9 |  |  |
|  | 313 |  | 39.0 |  |  |
|  | 318 |  | 37.1 |  |  |
| Lu et al., 2008 | 298 | 1-5 μm | 41±2.5 | - | ATR-FTIR |
| Gough et al., 2011 | 223-273 | >1 μm | 38.0±2.9 | 12.6±2.0 | Micro-Raman |
|  | 273 |  | 35.4±2.3 | 12.6±2.0 |  |
| Toner and Catling, 2018 | 298 | - | 43.9 | - | Nonisopiestic method |

**Comments:**

The DRH of NaClO_4_ was measured by six studies (Zhang et al., 2005; Zhao et al., 2005; Apelblat and Manzurola, 2007; Lu et al., 2008; Gough et al., 2011; Toner and Catling, 2018). It was determined to be 38-49% at 298 K by five studies (Zhang et al., 2005; Zhao et al., 2005; Apelblat and Manzurola, 2007; Lu et al., 2008; Toner and Catling, 2018), showing relatively good agreement. Moreover, the DRH of NaClO_4_ was measured to be 38.0±2.9% at 223-273 K and 35.4±2.3% at 273 K (Gough et al., 2011).

Apelblat and Manzurola (2007) measured the DRH of NaClO_4_ at different temperature, and found that it decreased with temperature from 50.2% at 278 K to 37.1% at 318 K.

The ERH of NaClO_4_ was measured by four studies (Zhang and Chan, 2003; Zhang et al., 2005; Zhao et al., 2005; Gough et al., 2011). It was determined to be 20±2.5% (Zhang et al., 2005) and 18±2.5% (Zhao et al., 2005) at 298 K, suggesting good agreement. Moreover, the ERH of NaClO_4_ was measured to be 11% at 298 K (Zhang and Chan, 2003) and 12.6±2.0% at 223-273 K (Gough et al., 2011), respectively.

**Preferred values at 298 K for NaClO_4_:**

DRH: 38-49%

ERH: 15-23%

**References:**

Apelblat, A., and Manzurola, E.: The vapour pressures over saturated aqueous solutions of sodium and potassium acetates, chlorates, and perchlorates, Journal of Chemical Thermodynamics, 39, 1176-1181, 2007.

Gough, R. V., Chevrier, V. F., Baustian, K. J., Wise, M. E., and Tolbert, M. A.: Laboratory studies of perchlorate phase transitions: Support for metastable aqueous perchlorate solutions on Mars, Earth and Planetary Science Letters, 312, 371-377, 2011.

Lu, P. D., He, T., and Zhang, Y. H.: Relative humidity anneal effect on hygroscopicity of aerosol particles studied by rapid-scan FTIR-ATR spectroscopy, Geophysical Research Letters, 35, 3, 2008.

Toner, J. D., and Catling, D. C.: Chlorate brines on Mars: Implications for the occurrence of liquid water and deliquescence, Earth and Planetary Science Letters, 497, 161-168, 2018.

Zhang, Y. H., and Chan, C. K.: Observations of water monomers in supersaturated NaClO_4_, LiClO_4_, and Mg(ClO_4_)_2_ droplets using Raman spectroscopy, Journal of Physical Chemistry A, 107, 5956-5962, 2003.

Zhang, Y. H., Hu, Y. A., Ding, F., and Zhao, L. J.: FTIR-ATR chamber for observation of efflorescence and deliquescence processes of NaClO_4_ aerosol particles on ZnSe substrate, Chinese Science Bulletin, 50, 2149-2152, 2005.

Zhao, L. J., Zhang, Y. H., Wang, L. Y., Hu, Y. A., and Ding, F.: FTIR spectroscopic investigations of supersaturated NaClO_4_ aerosols, Physical Chemistry Chemical Physics, 7, 2723-2730, 2005.

## NaClO_4_∙H_2_O (sodium perchlorate monohydrate)

| Reference | *T* (K) | *D* | DRH (%) | ERH (%) | Techniques/Comments |
| --- | --- | --- | --- | --- | --- |
| Gough et al., 2011 | 228 | >1 μm | 64.1±4.0 | - | Micro-Raman |
|  | 273 |  | 50.9±1.6 | - |  |
| Jia et al., 2018 | 278 | - | 51.5±1 | - | VSA |
|  | 283 |  | 50.5±1 |  |  |
|  | 288 |  | 48.5±1 |  |  |
|  | 293 |  | 46.5±1 |  |  |
|  | 298 |  | 45.5±1 |  |  |
|  | 308 |  | 43.5±1 |  |  |
| Zhang et al., 2021 | 298 | - | 46.9±0.6 | - | VSA |

**Comments:**

The DRH of NaClO_4_∙H_2_O was measured by three studies (Gough et al., 2011; Jia et al., 2018; Zhang et al., 2021). It was determined to be 44-48% at 298 K (Jia et al., 2018; Zhang et al., 2021), showing good agreement between the two studies. In the third study (Gough et al., 2011), the DRH of NaClO_4_∙H_2_O was measured to be 64.1±4.0% at 228 K and 50.9±1.6% at 273 K. In addition, Jia et al. (2018) further investigated its dependence on temperature, and found that the measured DRH decreased with temperature from 51.5±1% at 278 K to 43.5±1% at 308 K.

**Preferred values at 298 K for NaClO_4_∙H_2_O:**

DRH: 44-48%

ERH: no preferred value

**References:**

Gough, R. V., Chevrier, V. F., Baustian, K. J., Wise, M. E., and Tolbert, M. A.: Laboratory studies of perchlorate phase transitions: Support for metastable aqueous perchlorate solutions on Mars, Earth and Planetary Science Letters, 312, 371-377, 2011.

Jia, X., Gu, W., Li, Y. J., Cheng, P., Tang, Y., Guo, L., Wang, X., and Tang, M.: Phase Transitions and Hygroscopic Growth of Mg(ClO_4_)_2_, NaClO_4_, and NaClO_4_·H_2_O: Implications for the Stability of Aqueous Water in Hyperarid Environments on Mars and on Earth, ACS Earth and Space Chemistry, 2, 159-167, 2018.

Zhang, Q.-N., Zhao, L.-J., Chen, S.-H., Guo, X., Luan, Y.-M., and Zhang, Y.-H.: Hygroscopic property of inorganic salts in atmospheric aerosols measured with physisorption analyzer, Atmospheric Environment, 247, 2021.

## KClO_4_ (potassium perchlorate)

| Reference | *T* (K) | *D* | DRH (%) | ERH (%) | Techniques/Comments |
| --- | --- | --- | --- | --- | --- |
| Apelblat and Manzurola, 2007 | 278 | - | 99.4 | - | Nonisopiestic method |
|  | 283 |  | 99.4 |  |  |
|  | 288 |  | 99.4 |  |  |
|  | 293 |  | 99.4 |  |  |
|  | 298 |  | 99.4 |  |  |
|  | 303 |  | 99.4 |  |  |
|  | 308 |  | 99.4 |  |  |
|  | 313 |  | 99.4 |  |  |
|  | 318 |  | 99.4 |  |  |
|  | 323 |  | 99.4 |  |  |

**Comments:**

One study (Apelblat and Manzurola, 2007) measured the DRH of KClO_4_ as a function of temperature, and it was determined to be 99.4% at 278-323 K, showing no variation with temperature.

**Preferred values at 298 K for KClO_4_:**

DRH: >99%

ERH: no preferred value

**References:**

Apelblat, A., and Manzurola, E.: The vapour pressures over saturated aqueous solutions of sodium and potassium acetates, chlorates, and perchlorates, Journal of Chemical Thermodynamics, 39, 1176-1181, 2007.

## Ca(ClO_4_)_2_ (calcium perchlorate)

| Reference | *T* (K) | *D* | DRH (%) | ERH (%) | Techniques/Comments |
| --- | --- | --- | --- | --- | --- |
| Pestova et al., 2005 | 298 | - | 54.6 | - | Solubility polytherms method |
| Nuding et al., 2014 | 223 | - | 55 | 15±4 | Micro-Raman |
|  | 253 |  | 26 | 2-10 |  |
|  | 273 |  | 13 | 3±2 |  |
|  | 298 | 10 μm | - | n. o. | EDB |
| Toner and Catling, 2018 | 298 | - | 13.8 | - | Nonisopiestic method |

**Comments:**

The DRH of Ca(ClO_4_)_2_ was measured by three studies (Pestova et al., 2005; Nuding et al., 2014; Toner and Catling, 2018). It was determined to be 54.6% at 298 K by Pestova et al. (2005) and 13.8% at 298 K by Toner and Catling (2018), showing a very large discrepancy. The third study (Nuding et al., 2014) found that the measured DRH of Ca(ClO_4_)_2_ decreased significantly with temperature from 55% at 223 K to 13% at 273 K.

Nuding et al. (2014) used two methods to measure the ERH of Ca(ClO_4_)_2_ at different temperature. It was determined to be 11-19% at 223 K, 2-10% at 253 K and 1-5% at 273 K; In addition, Nuding et al. (2014) observed gradual water evaporation of Ca(ClO_4_)_2_ particles with the decrease in RH at 298 K, and thus no ERH was reported.

**Preferred values at 298 K for Ca(ClO_4_)_2_:**

DRH: no preferred value

ERH: no preferred value

**References:**

Nuding, D. L., Rivera-Valentin, E. G., Davis, R. D., Gough, R. V., Chevrier, V. F., and Tolbert, M. A.: Deliquescence and efflorescence of calcium perchlorate: An investigation of stable aqueous solutions relevant to Mars, Icarus, 243, 420-428, 2014.

Pestova, O. N., Myund, L. A., Khripun, M. K., and Prigaro, A. V.: Polythermal study of the systems M(ClO_4_)_2_-H_2_O(M^2+^ = Mg^2+^, Ca^2+^, Sr^2+^, Ba^2+^), Russian Journal of Applied Chemistry, 78, 409-413, 2005.

Toner, J. D., and Catling, D. C.: Chlorate brines on Mars: Implications for the occurrence of liquid water and deliquescence, Earth and Planetary Science Letters, 497, 161-168, 2018.

## Ca(ClO_4_)_2_∙4H_2_O (calcium perchlorate tetrahydrate)

| Reference | *T* (K) | *D* | DRH (%) | ERH (%) | Techniques/Comments |
| --- | --- | --- | --- | --- | --- |
| Gu et al., 2017 | 278 | - | 18.5±0.5 | - | VSA |
|  | 283 |  | 17.5±0.5 |  |  |
|  | 288 |  | 17.5±0.5 |  |  |
|  | 293 |  | 16.5±0.5 |  |  |
|  | 298 |  | 16.5±0.5 |  |  |
|  | 303 |  | 16.5±0.5 |  |  |

**Comments:**

Gu et al. (2017) measured the DRH of Ca(ClO_4_)_2_∙4H_2_O as a function of temperature, and found that it decreased slightly with temperature from 18.5±0.5% at 278 K to 16.5±0.5% at 303 K.

**Preferred values at 298 K for Ca(ClO_4_)_2_∙4H_2_O:**

DRH: 16-17%

ERH: no preferred value

**References:**

Gu, W., Li, Y., Tang, M., Jia, X., Ding, X., Bi, X., and Wang, X.: Water uptake and hygroscopicity of perchlorates and implications for the existence of liquid water in some hyperarid environments, RSC Adv., 7, 46866-46873, 2017.

## Mg(ClO_4_)_2_ (magnesium perchlorate)

| Reference | *T* (K) | *D* | DRH (%) | ERH (%) | Techniques/Comments |
| --- | --- | --- | --- | --- | --- |
| Besley and Bottomley, 1969 | 296 | - | 50 | - | Nonisopiestic method |
| Zhang and Chan, 2003 | 298 | - | - | 10-18 | EDB |
| Pestova et al., 2005 | 298 | - | 50.3 | - | Solubility polytherms method |
| Nuding et al., 2014 | 298 | 10 μm | - | 15 | EDB |

**Comments:**

Two studies (Besley and Bottomley, 1969; Pestova et al., 2005) measured the DRH of Mg(ClO_4_)_2_. It was determined to be 50% at 296 K (Besley and Bottomley, 1969) and 50.3% at 298 K (Pestova et al., 2005), showing good agreement.

The ERH of Mg(ClO_4_)_2_ at 298 K was measured to be 10-18% (Zhang and Chan, 2003) and 15% (Nuding et al., 2014), suggesting good agreement.

**Preferred values at 298 K for Mg(ClO_4_)_2_:**

DRH: 50-51%

ERH: 10-18%

**References:**

Besley, L. M., and Bottomley, G. A.: The water vapour equilibria over magnesium perchlorate hydrates, The Journal of Chemical Thermodynamics, 1, 13-19, 1969.

Nuding, D. L., Rivera-Valentin, E. G., Davis, R. D., Gough, R. V., Chevrier, V. F., and Tolbert, M. A.: Deliquescence and efflorescence of calcium perchlorate: An investigation of stable aqueous solutions relevant to Mars, Icarus, 243, 420-428, 2014.

Pestova, O. N., Myund, L. A., Khripun, M. K., and Prigaro, A. V.: Polythermal study of the systems M(ClO_4_)_2_-H_2_O(M^2+^ = Mg^2+^, Ca^2+^, Sr^2+^, Ba^2+^), Russian Journal of Applied Chemistry, 78, 409-413, 2005.

Zhang, Y. H., and Chan, C. K.: Observations of water monomers in supersaturated NaClO_4_, LiClO_4_, and Mg(ClO_4_)_2_ droplets using Raman spectroscopy, Journal of Physical Chemistry A, 107, 5956-5962, 2003.

## Mg(ClO_4_)_2_∙6H_2_O (magnesium perchlorate hexahydrate)

| Reference | *T* (K) | *D* | DRH (%) | ERH (%) | Techniques/Comments |
| --- | --- | --- | --- | --- | --- |
| Gough et al., 2011 | 223 | >1 μm | 55.1±1.6 | 19.3±2.9 | Micro-Raman |
|  | 273 |  | 42.0±2.3 | 19.3±2.9 |  |
| Robertson and Bish, 2011 | 276-325 | - | ~40 | - | Thermogravimetric analyzer |
| Gu et al., 2017 | 278 | - | 42.8±0.6 | - | VSA |
|  | 283 |  | 42.2±0.6 |  |  |
|  | 288 |  | 41.5±0.5 |  |  |
|  | 293 |  | 41.2±0.5 |  |  |
|  | 298 |  | 40.5±0.5 |  |  |
|  | 303 |  | 40.5±0.5 |  |  |
| Toner and Catling, 2018 | 298 | - | 40.1 | - | Nonisopiestic method |

**Comments:**

The DRH of Mg(ClO_4_)_2_∙6H_2_O was measured by four studies (Gough et al., 2011; Robertson and Bish, 2011; Gu et al., 2017; Toner and Catling, 2018). It was determined to be 40-41% at 298 K by three studies (Robertson and Bish, 2011; Gu et al., 2017; Toner and Catling, 2018), showing good agreement.

In addition, Gough et al. (2011) suggested that the DRH of Mg(ClO_4_)_2_∙6H_2_O decreased with temperature from 55.1±1.6% at 223 K and 42.0±2.3% at 273 K; similarly, Gu et al. (2017) found that it decrease slightly with temperature from 42.8±0.6% at 278 K to 40.5±0.5% at 303 K.

The ERH of Mg(ClO_4_)_2_∙6H_2_O was determined to be 19.3±2.9% at both 223 and 273 K (Gough et al., 2011).

**Preferred values at 298 K for Mg(ClO_4_)_2_∙6H_2_O:**

DRH: 40-41%

ERH: no preferred value

**References:**

Gough, R. V., Chevrier, V. F., Baustian, K. J., Wise, M. E., and Tolbert, M. A.: Laboratory studies of perchlorate phase transitions: Support for metastable aqueous perchlorate solutions on Mars, Earth and Planetary Science Letters, 312, 371-377, 2011.

Gu, W., Li, Y., Tang, M., Jia, X., Ding, X., Bi, X., and Wang, X.: Water uptake and hygroscopicity of perchlorates and implications for the existence of liquid water in some hyperarid environments, RSC Adv., 7, 46866-46873, 2017.

Robertson, K., and Bish, D.: Stability of phases in the Mg(ClO_4_)_2_∙nH_2_O system and implications for perchlorate occurrences on Mars, Journal of Geophysical Research-Planets, 116, 2011.

Toner, J. D., and Catling, D. C.: Chlorate brines on Mars: Implications for the occurrence of liquid water and deliquescence, Earth and Planetary Science Letters, 497, 161-168, 2018.

# Carbonates

## Li_2_CO_3_ (lithium carbonate)

| Reference | *T* (K) | *D* | DRH (%) | ERH (%) | Techniques/Comments |
| --- | --- | --- | --- | --- | --- |
| Ma et al., 2020 | 298 | ~3 μm | >85 | - | Vacuum-FTIR |

**Comments:**

The DRH of LiCO_3_ was determined to be >85% at 298 K by Ma et al. (2020)

**Preferred values at 298 K for LiCO_3_:**

DRH: >85%

ERH: no preferred value

**References:**

Ma, S., Yang, M., Pang, S., and Zhang, Y.: Hygroscopic Growth and Phase Transitions of Na_2_CO_3_ and Mixed Na_2_CO_3_/Li_2_CO_3_ Particles: Influence of Li_2_CO_3_ on Phase Transitions of Na_2_CO_3_ and Formation of LiNaCO_3_, The journal of physical chemistry. A, 124, 10870-10878, 2020.

## Na_2_CO_3_ (sodium carbonate)

| Reference | *T* (K) | *D* | DRH (%) | ERH (%) | Techniques/Comments |
| --- | --- | --- | --- | --- | --- |
| Goldberg, 1981 | 298 | - | 89.8 | - | Nonisopiestic method |
| Lee and Chang, 2002 | 298 | 0.6-1.3 μm | 78±1 | 39-50 | Katharometer |
| Apelblat and Manzurola, 2003 | 278 | - | 86.8 | - | Nonisopiestic method |
|  | 283 |  | 88.9 |  |  |
|  | 288 |  | 90.6 |  |  |
|  | 293 |  | 91.6 |  |  |
|  | 298 |  | 92.6 |  |  |
|  | 303 |  | 92.9 |  |  |
|  | 308 |  | 92.8 |  |  |
|  | 313 |  | 92.4 |  |  |
|  | 318 |  | 91.6 |  |  |
|  | 323 |  | 90.5 |  |  |
| Ma et al., 2020 | 298 | ~3 μm | 60.1-75.0 | 38.9-50.8 | Vacuum-FTIR |
| Yang et al., 2020 | 298 | 2-8 μm | 72 | 59.8 | ATR-FTIR |

**Comments:**

Five studies (Goldberg, 1981; Lee and Chang, 2002; Apelblat and Manzurola, 2003; Ma et al., 2020; Yang et al., 2020) measured the DRH of Na_2_CO_3_, and reported divergent results: it was determined to be 90-93% at 298 K by Goldberg (1981) and Apelblat and Manzurola (2003), while the other three studies (Lee and Chang, 2002; Ma et al., 2020; Yang et al., 2020) found that the deliquescence of Na_2_CO_3_ took place at 60-79% at 298 K. In addition, Apelblat and Manzurola (2003) measured the DRH of Na_2_CO_3_ at different temperatures, and found that it first increased with temperature from 86.8% at 278 K to 92.9% at 303 K, then slightly decreased with temperature to 90.5% at 323 K.

Three studies (Lee and Chang, 2002; Ma et al., 2020; Yang et al., 2020) measured the ERH of Na_2_CO_3_. It was determined to be 39-50% (Lee and Chang, 2002) and 38.9-50.8% (Ma et al., 2020) at 298 K, showing good agreement. In addition, the ERH of Na_2_CO_3_ was measured to be 59.8% at 298 K by Yang et al. (2020), higher than those reported by the other two studies.

**Preferred values at 298 K for Na_2_CO_3_:**

DRH: >60%

ERH: 39-60%

**References:**

Apelblat, A., and Manzurola, E.: Solubilities and vapour pressures of saturated aqueous solutions of sodium tetraborate, sodium carbonate, and magnesium sulfate and freezing-temperature lowerings of sodium tetraborate and sodium carbonate solutions, Journal of Chemical Thermodynamics, 35, 221-238, 2003.

Goldberg, R. N.: Evaluated activity and osmotic coefficients for aqueous solutions: thirty-six uni-bivalent electrolytes, Journal of Physical and Chemical Reference Data, 10, 671-764, 1981.

Lee, C. T., and Chang, S. Y.: A GC-TCD method for measuring the liquid water mass of collected aerosols, Atmospheric Environment, 36, 1883-1894, 2002.

Ma, S., Yang, M., Pang, S., and Zhang, Y.: Hygroscopic Growth and Phase Transitions of Na_2_CO_3_ and Mixed Na_2_CO_3_/Li_2_CO_3_ Particles: Influence of Li_2_CO_3_ on Phase Transitions of Na_2_CO_3_ and Formation of LiNaCO_3_, The journal of physical chemistry. A, 124, 10870-10878, 2020.

Yang, P., Yang, H., Wang, N., Du, C., Pang, S., and Zhang, Y.: Hygroscopicity measurement of sodium carbonate, beta-alanine and internally mixed beta-alanine/Na_2_CO_3_ particles by ATR-FTIR, Journal of environmental sciences, 87, 250-259, 2020.

## K_2_CO_3_ (potassium carbonate) and K_2_CO_3_∙2H_2_O (potassium carbonate dihydrate)

| Species | Reference | *T* (K) | *D* | DRH (%) | ERH (%) | Techniques/Comments |
| --- | --- | --- | --- | --- | --- | --- |
| K_2_CO_3_ | Rockland, 1960 | 283 | - | 47 | - | Nonisopiestic method |
|  |  | 288 |  | 45 |  |  |
|  |  | 293 |  | 44 |  |  |
|  |  | 298 |  | 43 |  |  |
|  |  | 303 |  | 42 |  |  |
|  |  | 308 |  | 41 |  |  |
|  |  | 313 |  | 40 |  |  |
|  | Greenspan, 1977 | 273 | - | 43.1±0.7 | - | Nonisopiestic method |
|  |  | 278 |  | 43.1±0.5 |  |  |
|  |  | 283 |  | 43.1±0.4 |  |  |
|  |  | 288 |  | 43.2±0.3 |  |  |
|  |  | 293 |  | 43.2±0.3 |  |  |
|  |  | 298 |  | 43.2±0.4 |  |  |
|  |  | 303 |  | 43.2±0.5 |  |  |
|  | Apelblat et al., 1992 | 283 | - | 44.1 | - | Nonisopiestic method |
|  |  | 288 |  | 47.2 |  |  |
|  |  | 293 |  | 48.5 |  |  |
|  |  | 298 |  | 48.5 |  |  |
|  |  | 303 |  | 47.1 |  |  |
|  |  | 308 |  | 44.4 |  |  |
|  |  | 313 |  | 40.9 |  |  |
|  | Esat et al., 2018 | 283 | 0.6-4 μm | 44-50 | 9-25 | Optical tweezers |
| K_2_CO_3_∙2H_2_O | Stokes and Robinson, 1949 | 298 | - | 42.8 | - | Nonisopiestic method |

**Comments:**

The DRH of K_2_CO_3_ was measured by four studies (Rockland, 1960; Greenspan, 1977; Apelblat et al., 1992; Esat et al., 2018). It was determined to be 43-50% at 283-298 K. showed relatively good agreement.

In addition, three studies (Rockland, 1960; Greenspan, 1977; Apelblat et al., 1992) measured the DRH of K_2_CO_3_ as a function of temperature, and revealed different temperature dependence: Rockland (1960) found that it decreased with temperature from 47% at 283 K to 40% at 313 K, Greenspan (1977) found that it did not change significantly with temperature (273-303 K), and Apelblat et al. (1992) suggested that it first increased with temperature from 44.1% at 283 K to 48.5% at 298 K, and then decreased with temperature to 40.9% at 313 K.


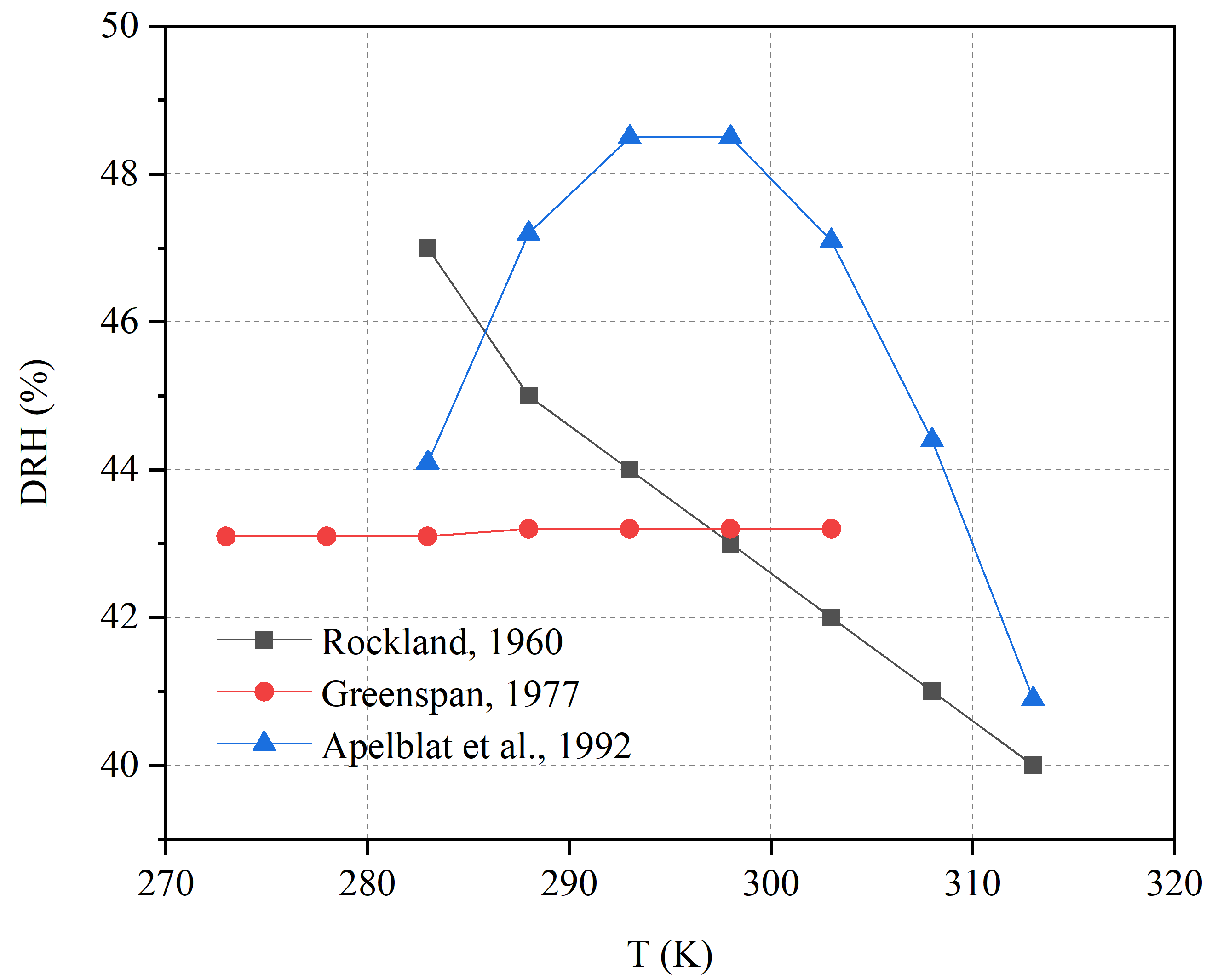


The ERH of K_2_CO_3_ was measured to be 9-25% at 283 K by Esat et al. (2018).

The DRH of K_2_CO_3_∙2H_2_O was determined to be 42.8% at 298 K by Stokes and Robinson (1949).

**Preferred values at 298 K for K_2_CO_3_:**

DRH: 43-50%

ERH: 9-25%

**Preferred values at 298 K for K_2_CO_3_∙2H_2_O:**

DRH: 42-43%

ERH: no preferred value

**References:**

Apelblat, A.: The vapor-pressures of water over saturated aqueous-solutions of barium-chloride, magnesium-nitrate, calcium nitrate, potassium carbonate, and zinc-sulfate at temperatures from 283 K to 313 K, Journal of Chemical Thermodynamics, 24, 619-626, 1992.

Esat, K., David, G., Poulkas, T., Shein, M., and Signorell, R.: Phase transition dynamics of single optically trapped aqueous potassium carbonate particles, Physical Chemistry Chemical Physics, 20, 11598-11607, 2018.

Greenspan, L.: Humidity fixed-points of binary saturated aqueous-solutions, Journal of Research of the National Bureau of Standards Section a-Physics and Chemistry, 81, 89-96, 1977.

Rockland, L. B.: Saturated Salt Solutions for Static Control of Relative Humidity between 5° and 40°C., Analytical Chemistry, 32, 1375-1376, 1960.

Stokes, R. H., and Robinson, R. A.: Standard Solutions for Humidity Control at 25°C., Industrial and Engineering Chemistry, 41, 2013-2013, 1949.

# Methanesulfonates

## CH_3_SO_3_NH_4_ (ammonium methanesulfonate)

| Reference | *T* (K) | *D* | DRH (%) | ERH (%) | Techniques/Comments |
| --- | --- | --- | --- | --- | --- |
| Liu and Laskin, 2009 | 297 | ~1 μm | n. o. | n. o. | Micro-FTIR |

**Comments:**

Liu and Laskin (2009) investigated hygroscopic properties of of CH_3_SO_3_NH_4_ particles at 297 K, and found these particles showed continueous uptake (with the increase in RH) or loss of water (with the decrease in RH); therefore, no DRH and ERH values were reported.

**Preferred values at 298 K for CH_3_SO_3_NH_4_:**

DRH: no preferred value

ERH: no preferred value

**References:**

Liu, Y., and Laskin, A.: Hygroscopic Properties of CH_3_SO_3_Na, CH_3_SO_3_NH_4_, (CH_3_SO_3_)_2_Mg, and (CH_3_SO_3_)_2_Ca Particles Studied by micro-FTIR Spectroscopy, Journal of Physical Chemistry A, 113, 1531-1538, 2009.

## CH_3_SO_3_Na (sodium methanesulfonate)

| Reference | *T* (K) | *D* | DRH (%) | ERH (%) | Techniques/Comments |
| --- | --- | --- | --- | --- | --- |
| Peng and Chan, 2001 | 298 | ~20 μm | 65.2-68.9 | 50.1-51.1 | EDB |
| Liu and Laskin, 2009 | 297 | ~1 μm | 71±1.0 | 49±1.0 | Micro-FTIR |
| Zeng et al., 2014 | 268 | ~1 μm | 82-84 | 63-65 | ATR-FTIR |
|  | 278 |  | 79-81 | 61-63 |  |
|  | 285 |  | 73-75 | 53-55 |  |
|  | 296 |  | 71-73 | 50-52 |  |
| Guo et al., 2020 | 298 | - | 70.5±0.5 | - | VSA |
|  | 298 | 100 nm | ~70 | - | HTDMA |

**Comments:**

Four studies (Peng and Chan, 2001; Liu and Laskin, 2009; Zeng et al., 2014; Guo et al., 2020) measured the DRH of CH_3_SO_3_Na. It was determined to be 65-73% at 296-298 K, showing good agreement. Moreover, Zeng et al. (2014) measured the DRH of CH_3_SO_3_Na at different temperatures, and found that it decreased with temperature from 82-84% at 268 K to 71-73% at 296 K.

The ERH of CH_3_SO_3_Na was measured to be 48-52% at 296-298 K by three studies (Peng and Chan, 2001; Liu and Laskin, 2009; Zeng et al., 2014), suggesting good agreement. Moreover, Zeng et al. (2014) also measured the ERH of CH_3_SO_3_Na at different temperatures, and found that it decreased with temperature from 63-65% at 268 K to 50-52% at 296 K.

**Preferred values at 298 K for CH_3_SO_3_Na:**

DRH: 65-73%

ERH: 48-52%

**References:**

Guo, L., Peng, C., Zong, T., Gu, W., Ma, Q., Wu, Z., Wang, Z., Ding, X., Hu, M., Wang, X., and Tang, M.: Comprehensive characterization of hygroscopic properties of methanesulfonates, Atmospheric Environment, 224, 117349, 2020.

Liu, Y., and Laskin, A.: Hygroscopic Properties of CH_3_SO_3_Na, CH_3_SO_3_NH_4_, (CH_3_SO_3_)_2_Mg, and (CH_3_SO_3_)_2_Ca Particles Studied by micro-FTIR Spectroscopy, Journal of Physical Chemistry A, 113, 1531-1538, 2009.

Peng, C. G., and Chan, C. K.: The water cycles of water-soluble organic salts of atmospheric importance, Atmospheric Environment, 35, 1183-1192, 2001.

Zeng, G., Kelley, J., Kish, J. D., and Liu, Y.: Temperature-dependent deliquescent and efflorescent properties of methanesulfonate sodium studied by ATR-FTIR spectroscopy, The journal of physical chemistry. A, 118, 583-591, 2014.

## CH_3_SO_3_K (potassium methanesulfonate)

| Reference | *T* (K) | *D* | DRH (%) | ERH (%) | Techniques/Comments |
| --- | --- | --- | --- | --- | --- |
| Guo et al., 2020 | 298 | - | 70-75 | - | VSA |
|  | 298 | 100 nm | 70-75 | - | HTDMA |

**Comments:**

Guo et al. (2020) used two methods to measure the DRH of CH_3_SO_3_K, and found that it was determined to be 70-75% at 298 K.

**Preferred values at 298 K for CH_3_SO_3_K:**

DRH: 70-75%

ERH: no preferred value

**References:**

Guo, L., Peng, C., Zong, T., Gu, W., Ma, Q., Wu, Z., Wang, Z., Ding, X., Hu, M., Wang, X., and Tang, M.: Comprehensive characterization of hygroscopic properties of methanesulfonates, Atmospheric Environment, 224, 117349, 2020.

## Ca(CH_3_SO_3_)_2_ (calcium methanesulfonate)

| Reference | *T* (K) | *D* | DRH (%) | ERH (%) | Techniques/Comments |
| --- | --- | --- | --- | --- | --- |
| Liu and Laskin, 2009 | 297 | ~1 μm | n. o. | n. o. | Micro-FTIR |
| Guo et al., 2020 | 298 | - | 70-75 | - | VSA |
|  | 298 | 100 nm | n. o. | - | HTDMA |

**Comments:**

Two studies (Liu and Laskin, 2009; Guo et al., 2020) measured the DRH of Ca(CH_3_SO_3_)_2_. Guo et al. (2020) suggested that hygroscopic behavior of Ca(CH_3_SO_3_)_2_ was closely related to the initial phase state of particles: deliquescence took place at 70-75% at 298 K for dry deposited (or crystalline) particles, and wet dispersed (or amorphous) particles displayed continuous water uptake. Liu and Laskin (2009) also observed gradual hygroscopic growth for Ca(CH_3_SO_3_)_2_ with the increase in RH at 297 K.

In addition, Liu and Laskin (2009) found that Ca(CH_3_SO_3_)_2_ displayed continuous water evaporation with the decrease in RH at 297 K, and thus no ERH was reported.

**Preferred values at 298 K for Ca(CH_3_SO_3_)_2_:**

DRH: 70-75%

ERH: no preferred value

**References:**

Guo, L., Peng, C., Zong, T., Gu, W., Ma, Q., Wu, Z., Wang, Z., Ding, X., Hu, M., Wang, X., and Tang, M.: Comprehensive characterization of hygroscopic properties of methanesulfonates, Atmospheric Environment, 224, 117349, 2020.

Liu, Y., and Laskin, A.: Hygroscopic Properties of CH_3_SO_3_Na, CH_3_SO_3_NH_4_, (CH_3_SO_3_)_2_Mg, and (CH_3_SO_3_)_2_Ca Particles Studied by micro-FTIR Spectroscopy, Journal of Physical Chemistry A, 113, 1531-1538, 2009.

## Mg(CH_3_SO_3_)_2_ (magnesium methanesulfonate)

| Reference | *T* (K) | *D* | DRH (%) | ERH (%) | Techniques/Comments |
| --- | --- | --- | --- | --- | --- |
| Liu and Laskin, 2009 | 297 | ~1 μm | n. o. | n. o. | Micro-FTIR |

**Comments:**

Liu and Laskin (2009) investigated hygroscopic properties of of Mg(CH_3_SO_3_)_2_ particles at 297 K, and found these particles showed continueous uptake (with the increase in RH) or loss of water (with the decrease in RH); therefore, no DRH and ERH values were reported.

**Preferred values at 298 K for Mg(CH_3_SO_3_)_2_:**

DRH: no preferred value

ERH: no preferred value

**References:**

Liu, Y., and Laskin, A.: Hygroscopic Properties of CH_3_SO_3_Na, CH_3_SO_3_NH_4_, (CH_3_SO_3_)_2_Mg, and (CH_3_SO_3_)_2_Ca Particles Studied by micro-FTIR Spectroscopy, Journal of Physical Chemistry A, 113, 1531-1538, 2009.

# Monocarboxylic salts

## HCOONa (sodium formate)

| Reference | *T* (K) | *D* | DRH (%) | ERH (%) | Techniques/Comments |
| --- | --- | --- | --- | --- | --- |
| Peng and Chan, 2001 | 298 | ~20 μm | 50.5-52.1 | 26.8-29.1 | EDB |
| Gao et al., 2018 | 270 | ~0.7 μm | 44-46 | 16-21 | ATR-FTIR |
|  | 278 |  | 50-52 | 20-27 |  |
|  | 284 |  | 56-58 | 26-32 |  |
|  | 290 |  | 58-61 | 29-35 |  |
|  | 296 |  | 60-62 | 31-37 |  |

**Comments:**

Two studies (Peng and Chan, 2001; Gao et al., 2018) measured the DRH of HCOONa. Peng and Chan (2001) observed that HCOONa particle was deliquesced at 50.5-52.1% at 298 K. Gao et al. (2018) measured the DRH of HCOONa at different temperature, and found that it increased with temperature from 44-46% at 270 K to 60-62% at 296 K.

Peng and Chan (2001) and Gao et al. (2018) also measured the ERH of HCOONa. It was determined to be 26.8-29.1% at 298 K by Peng and Chan (2001). Gao et al. (2018) found that the ERH of HCOONa increased with temperature from 16-21% at 270 K to 31-37% at 296 K.

**Preferred values at 298 K for HCOONa:**

DRH: 50-62%

ERH: 26-37%

**References:**

Gao, X., Zhang, Y., and Liu, Y.: Temperature-dependent hygroscopic behaviors of atmospherically relevant water-soluble carboxylic acid salts studied by ATR-FTIR spectroscopy, Atmospheric Environment, 191, 312-319, 2018.

Peng, C. G., and Chan, C. K.: The water cycles of water-soluble organic salts of atmospheric importance, Atmospheric Environment, 35, 1183-1192, 2001.

## Ca(HCOO)_2_ (calcium formate) and Mg(HCOO)_2_∙2H_2_O (magnesium formate dihydrate)

| Species | Reference | *T* (K) | *D* | DRH (%) | ERH (%) | Techniques/Comments |
| --- | --- | --- | --- | --- | --- | --- |
| Ca(HCOO)_2_ | Guo et al., 2019 | 298 | - | >95 | - | VSA |
| Mg(HCOO)_2_∙2H_2_O | Guo et al., 2019 | 298 | - | >95 | - | VSA |

**Comments:**

The DRH values at 298 K were determined to be >95% for both Ca(HCOO)_2_ and Mg(HCOO)_2_∙2H_2_O (Guo et al., 2019).

**Preferred values at 298 K for Ca(HCOO)_2_:**

DRH: >95%

ERH: no preferred value

**Preferred values at 298 K for Mg(HCOO)_2_∙2H_2_O:**

DRH: >95%

ERH: no preferred value

**References:**

Guo, L., Gu, W., Peng, C., Wang, W., Li, Y. J., Zong, T., Tang, Y., Wu, Z., Lin, Q., Ge, M., Zhang, G., Hu, M., Bi, X., Wang, X., and Tang, M.: A comprehensive study of hygroscopic properties of calcium- and magnesium-containing salts: implication for hygroscopicity of mineral dust and sea salt aerosols, Atmospheric Chemistry and Physics, 19, 2115-2133, 2019.

## CH_3_COONH_4_ (ammonium acetate)

| Reference | *T* (K) | *D* | DRH (%) | ERH (%) | Techniques/Comments |
| --- | --- | --- | --- | --- | --- |
| Apelblat and Korin, 2003 | 281-327 | - | 57-68 | - | Nonisopiestic method |

**Comments:**

The DRH of CH_3_COONH_4_ was determined to be 57-68% at 281-327 K by Apelblat and Korin (2003).

**Preferred values at 298 K for CH_3_COONH_4_:**

DRH: 57-68%

ERH: no preferred value

**References:**

Apelblat, A., and Korin, E.: The molar enthalpies of solution and vapour pressures of saturated aqueous solutions of some ammonium salts, Journal of Chemical Thermodynamics, 35, 699-709, 2003.

## CH_3_COOLi (lithium acetate)

| Reference | *T* (K) | *D* | DRH (%) | ERH (%) | Techniques/Comments |
| --- | --- | --- | --- | --- | --- |
| Rockland, 1960 | 278 | - | 72 | - | Nonisopiestic method |
|  | 283 |  | 72 |  |  |
|  | 288 |  | 71 |  |  |
|  | 293 |  | 70 |  |  |
|  | 298 |  | 68 |  |  |
|  | 303 |  | 66 |  |  |
|  | 308 |  | 65 |  |  |
|  | 313 |  | 64 |  |  |

**Comments:**

Rockland (1960) measured the DRH of CH_3_COOLi as a function of temperature, and it was determined to decrease with temperature from 72% at 278 K to 64% at 313 K.

**Preferred values at 298 K for CH_3_COOLi:**

DRH: 68%

ERH: no preferred value

**References:**

Rockland, L. B.: Saturated Salt Solutions for Static Control of Relative Humidity between 5° and 40°C., Analytical Chemistry, 32, 1375-1376, 1960.

## CH_3_COONa (sodium acetate)

| Reference | *T* (K) | *D* | DRH (%) | ERH (%) | Techniques/Comments |
| --- | --- | --- | --- | --- | --- |
| Peng and Chan, 2001 | 298 | ~20 μm | 43.5-45.2 | 36.7-40.6 | EDB |
| Wang et al., 2005 | 295-297 | 30-60 μm | - | 34.2 | EDB-Raman |
| Apelblat and Manzurola, 2007 | 278 | - | 79.1 | - | Nonisopiestic method |
|  | 283 |  | 79.1 |  |  |
|  | 288 |  | 78.5 |  |  |
|  | 293 |  | 77.3 |  |  |
|  | 298 |  | 75.7 |  |  |
|  | 303 |  | 73.6 |  |  |
|  | 308 |  | 71.2 |  |  |
|  | 313 |  | 68.4 |  |  |
|  | 318 |  | 65.4 |  |  |
| Wu et al., 2011 | 293 | 100 nm | 39-42 | - | HTDMA |
| Arenas et al., 2012 | 298 | - | 43.0±0.6 | - | QCM |
| Gao et al., 2018 | 270 | ~0.7 μm | 27-29 | 17-21 | ATR-FTIR |
|  | 278 |  | 35-37 | 27-31 |  |
|  | 284 |  | 39-41 | 32-36 |  |
|  | 290 |  | 44-46 | 37-41 |  |
|  | 296 |  | 46-48 | 39-43 |  |

**Comments:**

The DRH of CH_3_COONa was measured by five studies (Peng and Chan, 2001; Apelblat and Manzurola, 2007; Wu et al., 2011; Arenas et al., 2012; Gao et al., 2018). It was determined to be 39-48% at 293-298 K by four studies (Peng and Chan, 2001; Wu et al., 2011; Arenas et al., 2012; Gao et al., 2018), showing good agreement. Moreover, the DRH of CH_3_COONa was measured to be 75.7% at 298 K by Apelblat and Manzurola (2007), significantly higher than those reported by the other four studies.

Two studies (Apelblat and Manzurola, 2007; Gao et al., 2018) measured the DRH of CH_3_COONa as a function of temperature. Apelblat and Manzurola (2007) found that it decreased with temperature from 79.1% at 278 K to 65.4% at 318 K; on the contrary, Gao et al. (2018) suggested that it increased with temperature from 27-29% at 270 K to 46-48% at 296 K.


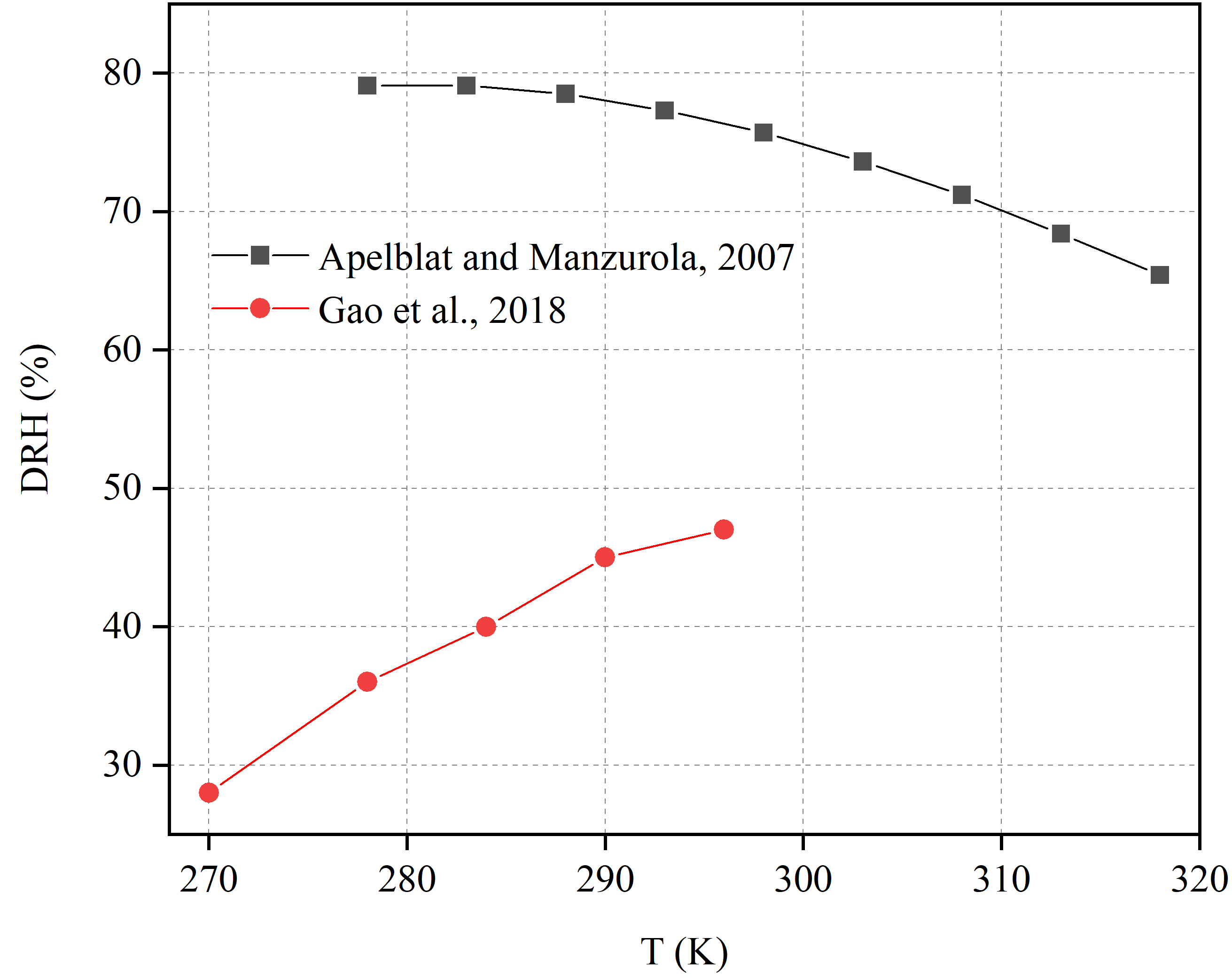


Three studies (Peng and Chan, 2001; Wang et al., 2005; Gao et al., 2018) measured the ERH of CH_3_COONa. It was determined to be 34-43% at 295-298 K, suggesting good agreement among these studies. In addition, Gao et al. (2018) measured the ERH of CH_3_COONa as a function of temperature, and found that it increased with temperature from 17-21% at 270 K to 39-43% at 296 K.

**Preferred values at 298 K for CH_3_COONa:**

DRH: 39-48%

ERH: 34-43%

**References:**

Apelblat, A., and Manzurola, E.: The vapour pressures over saturated aqueous solutions of sodium and potassium acetates, chlorates, and perchlorates, Journal of Chemical Thermodynamics, 39, 1176-1181, 2007.

Arenas, K. J. L., Schill, S. R., Malla, A., and Hudson, P. K.: Deliquescence Phase Transition Measurements by Quartz Crystal Microbalance Frequency Shifts, Journal of Physical Chemistry A, 116, 7658-7667, 2012.

Gao, X., Zhang, Y., and Liu, Y.: Temperature-dependent hygroscopic behaviors of atmospherically relevant water-soluble carboxylic acid salts studied by ATR-FTIR spectroscopy, Atmospheric Environment, 191, 312-319, 2018.

Peng, C. G., and Chan, C. K.: The water cycles of water-soluble organic salts of atmospheric importance, Atmospheric Environment, 35, 1183-1192, 2001.

Wang, L. Y., Zhang, Y. H., and Zhao, L. J.: Raman spectroscopic studies on single supersaturated droplets of sodium and magnesium acetate, Journal of Physical Chemistry A, 109, 609-614, 2005.

Wu, Z. J., Nowak, A., Poulain, L., Herrmann, H., and Wiedensohler, A.: Hygroscopic behavior of atmospherically relevant water-soluble carboxylic salts and their influence on the water uptake of ammonium sulfate, Atmospheric Chemistry and Physics, 11, 12617-12626, 2011.

## CH_3_COOK (potassium acetate)

| Reference | *T* (K) | *D* | DRH (%) | ERH (%) | Techniques/Comments |
| --- | --- | --- | --- | --- | --- |
| Rockland, 1960 | 278 | - | 25 | - | Nonisopiestic method |
|  | 283 |  | 24 |  |  |
|  | 288 |  | 24 |  |  |
|  | 293 |  | 23 |  |  |
|  | 298 |  | 23 |  |  |
|  | 303 |  | 23 |  |  |
|  | 308 |  | 23 |  |  |
|  | 313 |  | 23 |  |  |
| Greenspan, 1977 | 283 | - | 23.38±0.53 | - | Nonisopiestic method |
|  | 288 |  | 23.40±0.32 |  |  |
|  | 293 |  | 23.11±0.25 |  |  |
|  | 298 |  | 22.51±0.32 |  |  |
|  | 303 |  | 21.61±0.53 |  |  |
| Apelblat and Manzurola, 2007 | 278 | - | 19.8 | - | Nonisopiestic method |
|  | 283 |  | 19.8 |  |  |
|  | 288 |  | 19.5 |  |  |
|  | 293 |  | 19.0 |  |  |
|  | 298 |  | 18.3 |  |  |
|  | 303 |  | 17.5 |  |  |
|  | 308 |  | 16.5 |  |  |
|  | 313 |  | 15.5 |  |  |
|  | 318 |  | 14.4 |  |  |
| Arenas et al., 2012 | 298 | - | 20.1±0.1 | - | QCM |

**Comments:**

The DRH of CH_3_COOK was measured by four studies (Rockland, 1960; Greenspan, 1977; Apelblat and Manzurola, 2007; Arenas et al., 2012). It was determined to be 20-23% at 298 K by three studies (Rockland, 1960; Greenspan, 1977; Arenas et al., 2012), showing good agreement. Moreover, the DRH of CH_3_COOK was measured to be 18.3% at 298 K (Apelblat and Manzurola, 2007), slightly lower than that reported by the other studies.


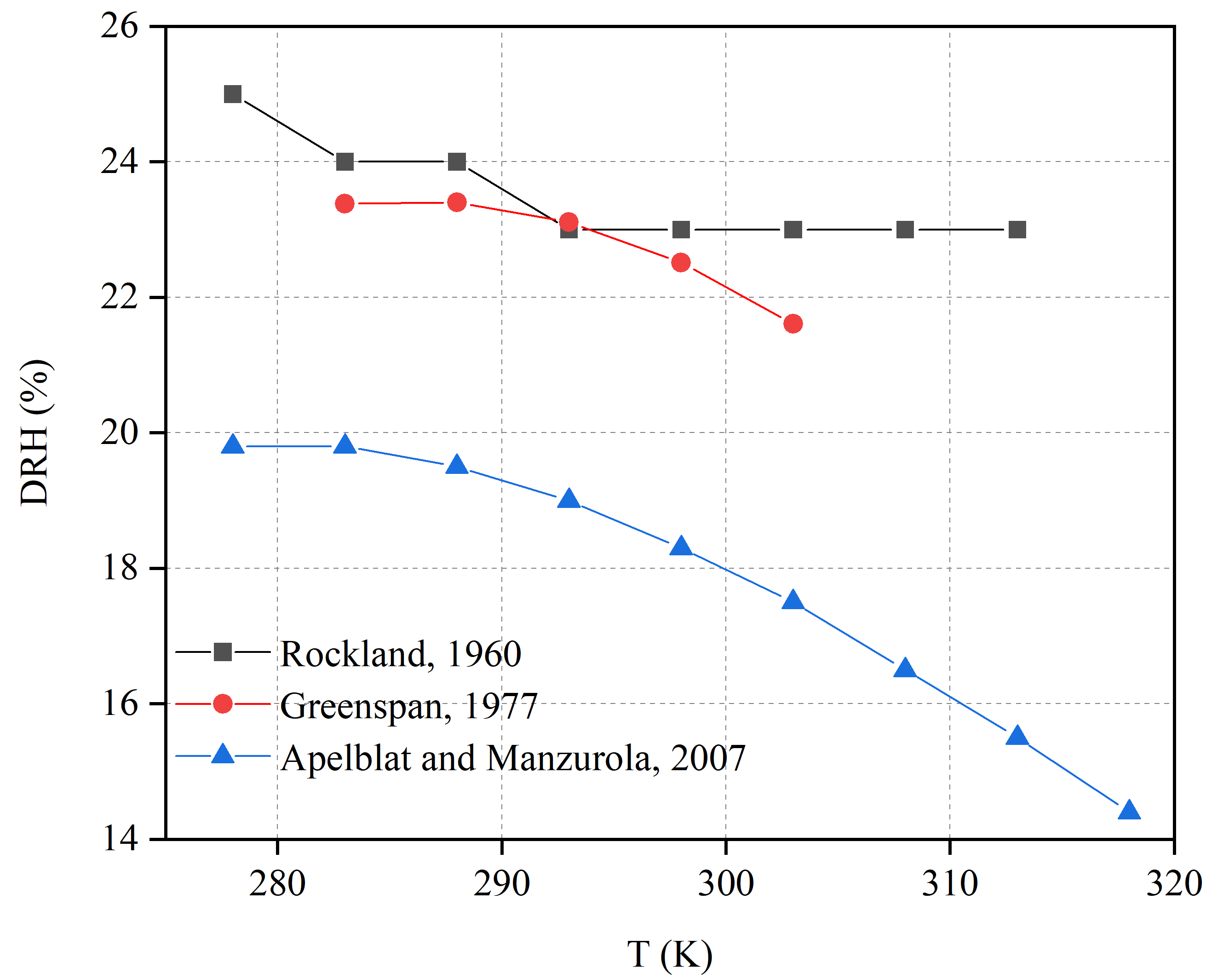


Three studies (Rockland, 1960; Greenspan, 1977; Apelblat and Manzurola, 2007) measured the DRH of CH_3_COOK at different temperature, showing similar temperature dependence: Rockland (1960) found that it decreased slightly with temperature from 25% at 278 K to 23% at 313 K, Greenspan (1977) found that it decreased slightly with temperature from 23.38±0.53% at 283 K to 21.61±0.53% at 303 K, and Apelblat and Manzurola (2007) suggested that it decreased with temperature from 19.8% at 278 K to 14.4% at 318 K.

**Preferred values at 298 K for CH_3_COOK:**

DRH: 18-23%

ERH: no preferred value

**References:**

Apelblat, A., and Manzurola, E.: The vapour pressures over saturated aqueous solutions of sodium and potassium acetates, chlorates, and perchlorates, Journal of Chemical Thermodynamics, 39, 1176-1181, 2007.

Arenas, K. J. L., Schill, S. R., Malla, A., and Hudson, P. K.: Deliquescence Phase Transition Measurements by Quartz Crystal Microbalance Frequency Shifts, Journal of Physical Chemistry A, 116, 7658-7667, 2012.

Greenspan, L.: Humidity fixed-points of binary saturated aqueous-solutions, Journal of Research of the National Bureau of Standards Section a-Physics and Chemistry, 81, 89-96, 1977.

Rockland, L. B.: Saturated Salt Solutions for Static Control of Relative Humidity between 5° and 40°C., Analytical Chemistry, 32, 1375-1376, 1960.

## Ca(CH_3_COO)_2_ (calcium acetate) and Ca(CH_3_COO)_2_∙H_2_O (calcium acetate monohydrate)

| Species | Reference | *T* (K) | *D* | DRH (%) | ERH (%) | Techniques/Comments |
| --- | --- | --- | --- | --- | --- | --- |
| Ca(CH_3_COO)_2_ | Apelblat and Korin, 2001 | 278 | - | 78.5 | - | Nonisopiestic method |
|  |  | 283 |  | 79.7 |  |  |
|  |  | 288 |  | 80.9 |  |  |
|  |  | 293 |  | 82.1 |  |  |
|  |  | 298 |  | 83.2 |  |  |
|  |  | 303 |  | 84.3 |  |  |
|  |  | 308 |  | 85.3 |  |  |
|  |  | 313 |  | 86.3 |  |  |
|  |  | 318 |  | 87.3 |  |  |
|  | Ma et al., 2012 | 278 | - | 85 | - | PSA |
| Ca(CH_3_COO)_2_∙H_2_O | Guo et al., 2019 | 298 | - | 90.5±1.0 | - | VSA |

**Comments:**

The DRH of Ca(CH_3_COO)_2_ was measured by two studies (Apelblat and Korin, 2001; Ma et al., 2012). Apelblat and Korin (2001) found that it increased with temperature from 78.5% at 278 K to 87.3% at 318 K. Moreover, the DRH of Ca(CH_3_COO)_2_ was determined to be 85% at 278 K by Ma et al. (2012), slightly higher than that measured at 278 K (78.5%) by Apelblat and Korin (2001).

The DRH of Ca(CH_3_COO)_2_∙H_2_O was measured to be 90.5±1.0% at 298 K by Guo et al. (2019).

**Preferred values at 298 K for Ca(CH_3_COO)_2_:**

DRH: 83-84%

ERH: no preferred value

**Preferred values at 298 K for Ca(CH_3_COO)_2_∙H_2_O:**

DRH: 89-92%

ERH: no preferred value

**References:**

Apelblat, A., and Korin, E.: The vapour pressures of saturated aqueous solutions of magnesium, calcium, nickel and zinc acetates and molar enthalpies of solution of magnesium, calcium, zinc and lead acetates, Journal of Chemical Thermodynamics, 33, 113-120, 2001.

Guo, L., Gu, W., Peng, C., Wang, W., Li, Y. J., Zong, T., Tang, Y., Wu, Z., Lin, Q., Ge, M., Zhang, G., Hu, M., Bi, X., Wang, X., and Tang, M.: A comprehensive study of hygroscopic properties of calcium- and magnesium-containing salts: implication for hygroscopicity of mineral dust and sea salt aerosols, Atmospheric Chemistry and Physics, 19, 2115-2133, 2019.

Ma, Q., Liu, Y., Liu, C., and He, H.: Heterogeneous reaction of acetic acid on MgO, alpha-Al_2_O_3_, and CaCO_3_ and the effect on the hygroscopic behaviour of these particles, Physical Chemistry Chemical Physics, 14, 8403-8409, 2012.

## Mg(CH_3_COO)_2_ (magnesium acetate) and Mg(CH_3_COO)_2_∙4H_2_O (magnesium acetate tetrahydrate)

| Species | Reference | *T* (K) | *D* | DRH (%) | ERH (%) | Techniques/Comments |
| --- | --- | --- | --- | --- | --- | --- |
| Mg(CH_3_COO)_2_ | Apelblat and Korin, 2001 | 283 | - | 68.4 | - | Nonisopiestic method |
|  |  | 288 |  | 70.8 |  |  |
|  |  | 293 |  | 72.7 |  |  |
|  |  | 298 |  | 74.1 |  |  |
|  |  | 303 |  | 75.0 |  |  |
|  |  | 308 |  | 75.5 |  |  |
|  |  | 313 |  | 75.6 |  |  |
|  |  | 318 |  | 75.2 |  |  |
|  |  | 323 |  | 74.4 |  |  |
|  | Wang et al., 2005 | 295-297 | 30-60 μm | 65 | 43.9 | EDB-Raman |
| Mg(CH_3_COO)_2_∙4H_2_O | Guo et al., 2019 | 298 | - | 71.5±1.0 | - | VSA |

**Comments:**

The DRH of Mg(CH_3_COO)_2_ was measured by two studies (Apelblat and Korin, 2001; Wang et al., 2005). Apelblat and Korin (2001) found that it first increased with temperature from 68.4% at 283 K to 75.6% at 313 K, and then slightly decreased with temperature to 74.4% at 323 K; in the other study (Wang et al., 2005), the DRH of Mg(CH_3_COO)_2_ was determined to be 65% at 295-297 K, lower than that measured at 298 K (74.1%) by Apelblat and Korin (2001). In addition, the ERH of Mg(CH_3_COO)_2_ was determined to be 43.9% at 295-297 K by Wang et al. (2005).

The DRH of Mg(CH_3_COO)_2_∙4H_2_O was measured to be 71.5±1.0 at 298 K (Guo et al., 2019).

**Preferred values at 298 K for Mg(CH_3_COO)_2_:**

DRH: 65-74%

ERH: 43-44%

**Preferred values at 298 K for Mg(CH_3_COO)_2_∙4H_2_O:**

DRH: 70-73%

ERH: no preferred value

**References:**

Apelblat, A., and Korin, E.: The vapour pressures of saturated aqueous solutions of magnesium, calcium, nickel and zinc acetates and molar enthalpies of solution of magnesium, calcium, zinc and lead acetates, Journal of Chemical Thermodynamics, 33, 113-120, 2001.

Guo, L., Gu, W., Peng, C., Wang, W., Li, Y. J., Zong, T., Tang, Y., Wu, Z., Lin, Q., Ge, M., Zhang, G., Hu, M., Bi, X., Wang, X., and Tang, M.: A comprehensive study of hygroscopic properties of calcium- and magnesium-containing salts: implication for hygroscopicity of mineral dust and sea salt aerosols, Atmospheric Chemistry and Physics, 19, 2115-2133, 2019.

Wang, L. Y., Zhang, Y. H., and Zhao, L. J.: Raman spectroscopic studies on single supersaturated droplets of sodium and magnesium acetate, Journal of Physical Chemistry A, 109, 609-614, 2005.

## NaC_3_H_3_O_3_ (sodium pyruvate)

| Reference | *T* (K) | *D* | DRH (%) | ERH (%) | Techniques/Comments |
| --- | --- | --- | --- | --- | --- |
| Peng and Chan, 2001 | 298 | ~20 μm | 71.6-83.9 | 56.3-60.0 | EDB |
| Wu et al., 2011 | 293 | 100 nm | 81-82 | - | HTDMA |

**Comments:**

The DRH of NaC_3_H_3_O_3_ was measured by two studies (Peng and Chan, 2001; Wu et al., 2011). It was determined to be 71.6-83.9% at 298 K (Peng and Chan, 2001) and 81-82% at 293 K (Wu et al., 2011), showing relatively good agreement.

The ERH of NaC_3_H_3_O_3_ was measured to be 56.3-60.0% at 298 K by Peng and Chan (2001).

**Preferred values at 298 K for NaC_3_H_3_O_3_:**

DRH: 71-84%

ERH: 56-60%

**References:**

Peng, C. G., and Chan, C. K.: The water cycles of water-soluble organic salts of atmospheric importance, Atmospheric Environment, 35, 1183-1192, 2001.

Wu, Z. J., Nowak, A., Poulain, L., Herrmann, H., and Wiedensohler, A.: Hygroscopic behavior of atmospherically relevant water-soluble carboxylic salts and their influence on the water uptake of ammonium sulfate, Atmospheric Chemistry and Physics, 11, 12617-12626, 2011.

# Dicarboxylic acids

## H_2_C_2_O_4_ (oxalic acid)

| Reference | *T* (K) | *D* | DRH (%) | ERH (%) | Techniques/Comments |
| --- | --- | --- | --- | --- | --- |
| Saxena and Hildemann, 1997 | 298 | - | 97-99 | - | Estimated by UNIFAC |
| Peng et al., 2001 | 298 | - | 97.3 | - | Nonisopiestic method |
|  |  | 10~20 μm | >94 | 51.8-56.7 | EDB |
| Prenni et al., 2001 | 303 | 50, 100 nm | n. o. | - | HTDMA |
| Brooks et al., 2002 | 277 | - | ≥95.0 | - | Nonisopiestic method |
|  | 297 |  | 93.0±3 |  |  |
| Braban et al., 2003 | 279 | - | 97 | - | AFT-FTIR |
|  | 284 |  | 96.8-99.6 | - |  |
|  | 288 |  | 97 | - |  |
|  | 294 |  | 98 | - |  |
|  | 298 |  | - | <5 |  |
| Wise et al., 2003 | 298 | - | 97.1 | - | Nonisopiestic method |
| Marcolli et al., 2004 | 298 | - | 97.8 | - | Nonisopiestic method |
| Clegg and Seinfeld, 2006 | 298 | - | 97.8 | - | Nonisopiestic method |
| Mikhailov et al., 2009 | 298 | 100 nm | n. o. | n. o. | HTDMA |
| Zamora et al., 2011 | 298 | - | 97.9±1.8 | - | Nonisopiestic method |
| Ma et al., 2013a,b | 278 | - | >95 | - | PSA |
| Jing et al., 2016 | 297 | 100 nm | >90 | - | HTDMA |
| Wang et al., 2017 | 297 | 10~20 μm | >94 | 71±2.5 | Micro-Raman |
| Boreddy and Kawamura, 2018 | 290 | 100 nm | n. o. | - | HTDMA |
| Ma et al., 2019 | 298 | 300 nm | >90 | - | HTDMA |

**Comments:**

A number of studies (Saxena and Hildemann, 1997; Peng et al., 2001; Prenni et al., 2001; Brooks et al., 2002; Braban et al., 2003; Wise et al., 2003; Marcolli et al., 2004; Clegg and Seinfeld, 2006; Mikhailov et al., 2009; Zamora et al., 2011; Ma et al., 2013; Jing et al., 2016; Wang et al., 2017; Boreddy and Kawamura, 2018; Ma et al., 2019) measured the DRH of H_2_C_2_O_4_. Most of these studies (Saxena and Hildemann, 1997; Peng et al., 2001; Brooks et al., 2002; Braban et al., 2003; Wise et al., 2003; Marcolli et al., 2004; Clegg and Seinfeld, 2006; Zamora et al., 2011; Ma et al., 2013; Jing et al., 2016; Wang et al., 2017; Ma et al., 2019) found that the deliquescence of H_2_C_2_O_4_ took place at >90% at 277-298 K, showing good agreement. However, the other three studies (Prenni et al., 2001; Mikhailov et al., 2009; Boreddy and Kawamura, 2018) observed continuous water uptake for H_2_C_2_O_4_ particles with increase in RH, and thus no DRH was reported.

In addition, two studies (Brooks et al., 2002; Braban et al., 2003) measured the DRH of H_2_C_2_O_4_ at different temperatures, and showed similar temperature dependence: it was determined to be ≥95.0% at 277 K and 93.0±3% at 297 K by Brooks et al. (2002); similarly, Braban et al. (2003) found that the DRH of H_2_C_2_O_4_ did not change significantly with temperature, and their measured DRH was determined to be 96.8-99.6% at 279-294 K.

Four studies (Peng et al., 2001; Braban et al., 2003; Mikhailov et al., 2009; Wang et al., 2017) measured the ERH of H_2_C_2_O_4_, and reported different results. It was determined to be 51.8-56.7% at 298 K by Peng et al. (2001) and 71±2.5% at 297 K by Wang et al. (2017), and Braban et al. (2003) found that the efflorescence took place at <5% at 298 K; moreover, Mikhailov et al. (2009) observed continuous loss of water for H_2_C_2_O_4_ at 298 K, and thus no ERH was reported.

**Preferred values at 298 K for H_2_C_2_O_4_:**

DRH: >90%

ERH: no preferred value

**References:**

Boreddy, S. K. R., and Kawamura, K.: Investigation on the hygroscopicity of oxalic acid and atmospherically relevant oxalate salts under sub- and supersaturated conditions, Environmental Science-Processes & Impacts, 20, 1069-1080, 2018.

Braban, C. F., Carroll, M. F., Styler, S. A., and Abbatt, J. P. D.: Phase transitions of malonic and oxalic acid aerosols, Journal of Physical Chemistry A, 107, 6594-6602, 2003.

Brooks, S. D., Wise, M. E., Cushing, M., and Tolbert, M. A.: Deliquescence behavior of organic/ammonium sulfate aerosol, Geophysical Research Letters, 29, 2002.

Clegg, S. L., and Seinfeld, J. H.: Thermodynamic models of aqueous solutions containing inorganic electrolytes and dicarboxylic acids at 298.15 K. 1. The acids as nondissociating components, Journal of Physical Chemistry A, 110, 5692-5717, 2006.

Jing, B., Tong, S., Liu, Q., Li, K., Wang, W., Zhang, Y., and Ge, M.: Hygroscopic behavior of multicomponent organic aerosols and their internal mixtures with ammonium sulfate, Atmospheric Chemistry and Physics, 16, 4101-4118, 2016.

Ma, Q., He, H., and Liu, C.: Hygroscopic properties of oxalic acid and atmospherically relevant oxalates, Atmospheric Environment, 69, 281-288, 2013.

Ma, Q., Ma, J., Liu, C., Lai, C., and He, H.: Laboratory study on the hygroscopic behavior of external and internal C2-C4 dicarboxylic acid-NaCl mixtures, Environ Sci Technol, 47, 10381-10388, 2013.

Ma, Q., Zhong, C., Liu, C., Liu, J., Ma, J., Wu, L., and He, H.: A Comprehensive Study about the Hygroscopic Behavior of Mixtures of Oxalic Acid and Nitrate Salts: Implication for the Occurrence of Atmospheric Metal Oxalate Complex, Acs Earth and Space Chemistry, 3, 1216-1225, 2019.

Marcolli, C., Luo, B. P., and Peter, T.: Mixing of the organic aerosol fractions: Liquids as the thermodynamically stable phases, Journal of Physical Chemistry A, 108, 2216-2224, 2004.

Mikhailov, E., Vlasenko, S., Martin, S. T., Koop, T., and Poeschl, U.: Amorphous and crystalline aerosol particles interacting with water vapor: conceptual framework and experimental evidence for restructuring, phase transitions and kinetic limitations, Atmospheric Chemistry And Physics, 9, 9491-9522, 2009.

Peng, C. G., and Chan, C. K.: The water cycles of water-soluble organic salts of atmospheric importance, Atmospheric Environment, 35, 1183-1192, 2001.

Prenni, A. J., DeMott, P. J., Kreidenweis, S. M., Sherman, D. E., Russell, L. M., and Ming, Y.: The effects of low molecular weight dicarboxylic acids on cloud formation, Journal Of Physical Chemistry A, 105, 11240-11248, 2001.

Saxena, P., and Hildemann, L. M.: Water absorption by organics: Survey of laboratory evidence and evaluation of UNIFAC for estimating water activity, Environmental Science & Technology, 31, 3318-3324, 1997.

Wang, X., Jing, B., Tan, F., Ma, J., Zhang, Y., and Ge, M.: Hygroscopic behavior and chemical composition evolution of internally mixed aerosols composed of oxalic acid and ammonium sulfate, Atmos. Chem. Phys., 17, 12797-12812, 2017.

Wise, M. E., Surratt, J. D., Curtis, D. B., Shilling, J. E., and Tolbert, M. A.: Hygroscopic growth of ammonium sulfate/dicarboxylic acids, Journal Of Geophysical Research-Atmospheres, 108, 2003.

Zamora, I. R., Tabazadeh, A., Golden, D. M., and Jacobson, M. Z.: Hygroscopic growth of common organic aerosol solutes, including humic substances, as derived from water activity measurements, Journal of Geophysical Research-Atmospheres, 116, 2011.

## CH_2_(COOH)_2_ (malonic acid)

| Reference | *T* (K) | *D* | DRH (%) | ERH (%) | Techniques/Comments |
| --- | --- | --- | --- | --- | --- |
| Saxena and Hildemann, 1997 | 298 | - | 74-91 | - | Estimated by UNIFAC |
| Peng et al., 2001 | 298 | - | 65.2 | - | Nonisopiestic method |
|  |  | 10~20 μm | n. o. | n. o. | EDB |
| Prenni et al., 2001 | 303 | 50, 100 nm | n. o. | <5 | HTDMA |
| Brooks et al., 2002 | 263 | - | 88.5±3 | - | Nonisopiestic method |
|  | 277 |  | 80.6±3 |  |  |
|  | 297 |  | 74.3±3 |  |  |
| Braban et al., 2003 | 264 | - | 83.7 | - | AFT-FTIR |
|  | 269 |  | 86.0 | - |  |
|  | 274 |  | 81.1 | - |  |
|  | 279 |  | 79.0 | - |  |
|  | 284 |  | 80.0 | - |  |
|  | 288 |  | 80.1 | - |  |
|  | 294 |  | 69.0 | 6 |  |
|  | 298 |  | - | 6 |  |
|  | 252 | - | 91.2 | - | SMC (Static mode chamber) |
|  | 258 |  | 86.5-87.3 | - |  |
|  | 260 |  | 88.3 | - |  |
|  | 264 |  | 85.7 | - |  |
|  | 277 |  | 82.5-84.9 | - |  |
| Wise et al., 2003 | 298 | - | 71.9 | - | Nonisopiestic method |
| Marcolli et al., 2004 | 298 | - | 72.4 | - | Nonisopiestic method |
| Parsons et al., 2004 | 243 | 2-40 μm | 86.7±2 | - | Optical microscopy |
|  | 246 |  | 86.5±2 |  |  |
|  | 251 |  | 85.5±2 |  |  |
|  | 254 |  | 85.2±2 |  |  |
|  | 263 |  | 81.7±2 |  |  |
|  | 272 |  | 80.5±2 |  |  |
|  | 283 |  | 75.6±2 |  |  |
|  | 292 |  | 73.7±2 |  |  |
| Salcedo, 2006 | 298 | - | 70.9 | - | Nonisopiestic method |
| Clegg and Seinfeld, 2006 | 298 | - | 72.5 | - | Nonisopiestic method |
| Moore and Raymond, 2008 | 296 | 80 nm | n. o. | - | HTDMA |
| Treuel et al., 2009 | 298 | - | 71±2 | - | Optical microscopy |
| Pope et al., 2010 | 280.5 | 20-50 μm | 75±1 | 17-22 | EDB |
|  | 295 |  | n. o. | - |  |
| Yeung and Chan, 2010 | 297 | 10-30 μm | 67±2 | <10 | Micro-Raman |
| Peckhaus et al., 2012 | 293 | - | 72±2 | - | Optical microscopy |
| Ma et al., 2013 | 278 | - | 80 | - | PSA |
| Beyer et al., 2014 | 277.4 | - | 80.2±1 | - | VSA |
|  | 283.0 |  | 79.7±1 |  |  |
|  | 288.0 |  | 75.5±1 |  |  |
|  | 293.2 |  | 73.2±1 |  |  |
|  | 298.1 |  | 71.5±1 |  |  |
|  | 303.0 |  | 69.5±1 |  |  |
| Ghorai et al., 2014 | 298 | 0.2~1.2 μm | n. o. | - | Micro-FTIR |
| Jing et al., 2016 | 297 | 100 nm | n. o. | - | HTDMA |
| Li et al., 2017 | 298 | - | 67.4±0.5 | 11.1±0.5 | Micro-FTIR |
| Shao et al., 2017 | 298 | - | ~76 | 12~17 | FTIR |

**Comments:**

The DRH of CH_2_(COOH)_2_ was measured by a number of studies (Saxena and Hildemann, 1997; Peng et al., 2001; Prenni et al., 2001; Brooks et al., 2002; Braban et al., 2003; Wise et al., 2003; Marcolli et al., 2004; Parsons et al., 2004; Salcedo, 2006; Clegg and Seinfeld, 2006; Moore and Raymond, 2008; Treuel et al., 2009; Pope et al., 2010; Yeung and Chan, 2010; Peckhaus et al., 2012; Ma et al., 2013; Beyer et al., 2014; Ghorai et al., 2014; Jing et al., 2016; Li et al., 2017; Shao et al., 2017). It was determined to be 65-76% at around room temperature (292-298 K) by most of these studie (Peng et al., 2001; Brooks et al., 2002; Braban et al., 2003; Wise et al., 2003; Marcolli et al., 2004; Parsons et al., 2004; Salcedo, 2006; Clegg and Seinfeld, 2006; Treuel et al., 2009; Yeung and Chan, 2010; Peckhaus et al., 2012; Beyer et al., 2014; Li et al., 2017; Shao et al., 2017), showing relatively good agreement. In addition, the DRH of CH_2_(COOH)_2_ was estimated to be 74-91% at 298 K by Saxena and Hildemann (1997), and Pope et al. (2010) and Ma et al. (2013) found that the deliquescence took place at 74-80% RH at 278-280 K; furthermore, four studies (Prenni et al., 2001; Pope et al., 2010; Ghorai et al., 2014; Jing et al., 2016) observed continuous water uptake for CH_2_(COOH)_2_ at 295-303 K, and thus no DRH was reported.


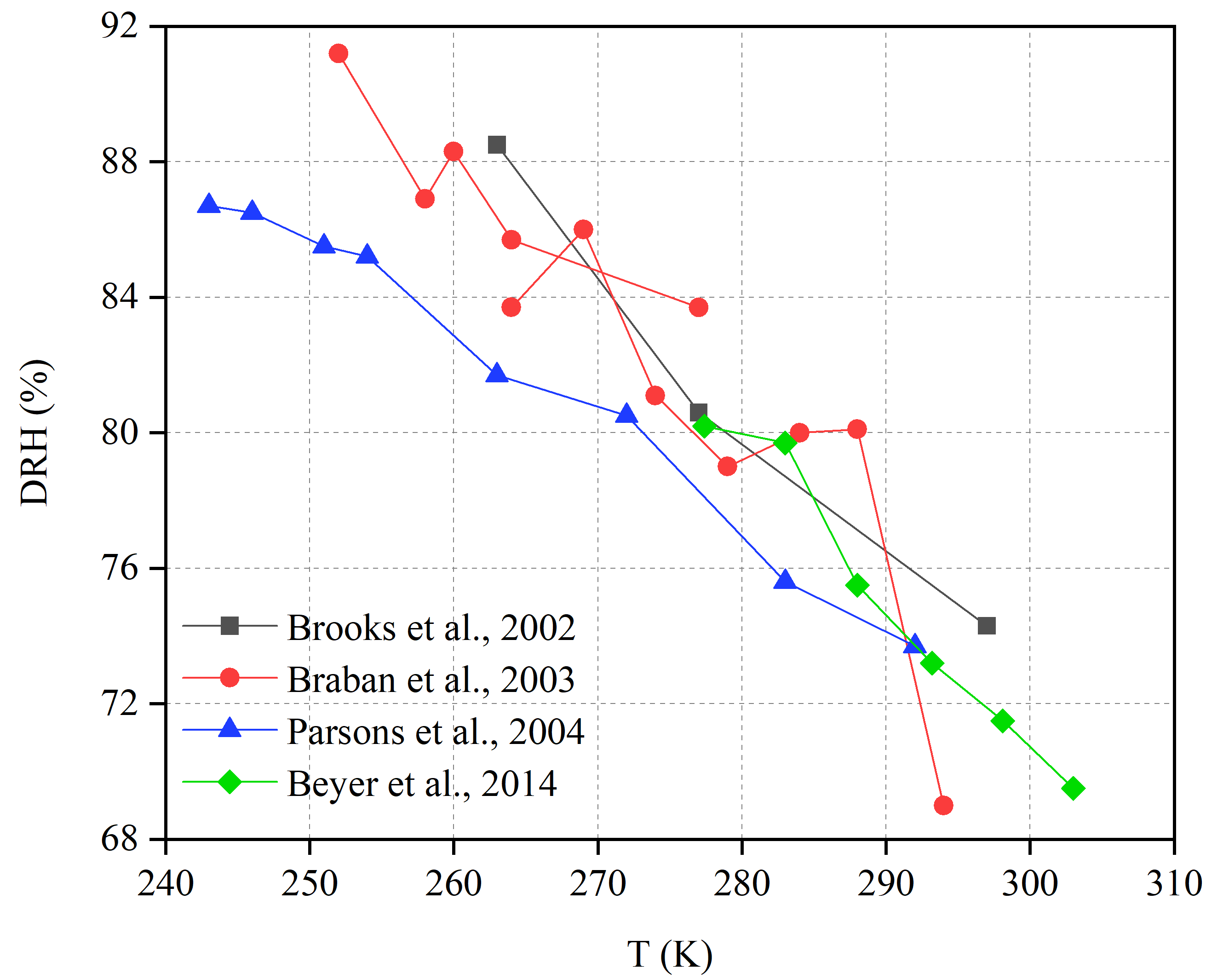


Four studies (Brooks et al., 2002; Braban et al., 2003; Parsons et al., 2004; Beyer et al., 2014) measured the DRH of CH_2_(COOH)_2_ at different temperatures, and revealed similar temperature dependence: Brooks et al. (2002) found that it decreased with temperature from 88.5±3% at 263 K to 74.3±3% at 297 K; Braban et al. (2003) found that it decreased with temperature from 91.2% at 252 K to 69.0% at 294 K; Parsons et al. (2004) suggested that it decreased with temperature from 86.7±2% at 243 K to 73.7±2% at 292 K; and Beyer et al. (2014) suggested that it decreased with temperature from 80.2±1% at 277.4 K to 69.5±1% at 303.0 K.

Seven studies (Peng et al., 2001; Prenni et al., 2001; Braban et al., 2003; Pope et al., 2010; Yeung and Chan, 2010; Li et al., 2017; Shao et al., 2017) measured the ERH of CH_2_(COOH)_2_, and reported different results. Four studies (Pope et al., 2010; Yeung and Chan, 2010; Li et al., 2017; Shao et al., 2017) suggested the efflorescence of CH_2_(COOH)_2_ took place at 10-22% at 280-298 K, and two other studies (Prenni et al., 2001; Braban et al., 2003) found that the efflorescence occurred at 5-6% at 294-303 K; in addition, Peng et al. (2001) observed continuous water evaporation for CH_2_(COOH)_2_ at 298 K, and thus no ERH was reported.

**Preferred values at 298 K for CH_2_(COOH)_2_:**

DRH: 65-76%

ERH: <22%

**References:**

Beyer, K. D., Schroeder, J. R., and Kissinger, J. A.: Temperature-Dependent Deliquescence Relative Humidities and Water Activities Using Humidity Controlled Thermogravimetric Analysis with Application to Malonic Acid, Journal of Physical Chemistry A, 118, 2488-2497, 2014.

Braban, C. F., Carroll, M. F., Styler, S. A., and Abbatt, J. P. D.: Phase transitions of malonic and oxalic acid aerosols, Journal of Physical Chemistry A, 107, 6594-6602, 2003.

Brooks, S. D., Wise, M. E., Cushing, M., and Tolbert, M. A.: Deliquescence behavior of organic/ammonium sulfate aerosol, Geophysical Research Letters, 29, 2002.

Clegg, S. L., and Seinfeld, J. H.: Thermodynamic models of aqueous solutions containing inorganic electrolytes and dicarboxylic acids at 298.15 K. 1. The acids as nondissociating components, Journal of Physical Chemistry A, 110, 5692-5717, 2006.

Ghorai, S., Wang, B., Tivanski, A., and Laskin, A.: Hygroscopic Properties of Internally Mixed Particles Composed of NaCl and Water-Soluble Organic Acids, Environmental Science & Technology, 48, 2234-2241, 2014.

Jing, B., Tong, S., Liu, Q., Li, K., Wang, W., Zhang, Y., and Ge, M.: Hygroscopic behavior of multicomponent organic aerosols and their internal mixtures with ammonium sulfate, Atmospheric Chemistry and Physics, 16, 4101-4118, 2016.

Li, X., Gupta, D., Lee, J., Park, G., and Ro, C.-U.: Real-Time Investigation of Chemical Compositions and Hygroscopic Properties of Aerosols Generated from NaCl and Malonic Acid Mixture Solutions Using in Situ Raman Microspectrometry, Environmental Science & Technology, 51, 263-270, 2017.

Ma, Q., Ma, J., Liu, C., Lai, C., and He, H.: Laboratory study on the hygroscopic behavior of external and internal C2-C4 dicarboxylic acid-NaCl mixtures, Environ Sci Technol, 47, 10381-10388, 2013.

Marcolli, C., Luo, B. P., and Peter, T.: Mixing of the organic aerosol fractions: Liquids as the thermodynamically stable phases, Journal of Physical Chemistry A, 108, 2216-2224, 2004.

Moore, R. H., and Raymond, T. M.: HTDMA analysis of multicomponent dicarboxylic acid aerosols with comparison to UNIFAC and ZSR, Journal of Geophysical Research, 113, 2008.

Parsons, M. T., Mak, J., Lipetz, S. R., and Bertram, A. K.: Deliquescence of malonic, succinic, glutaric, and adipic acid particles, Journal of Geophysical Research-Atmospheres, 109, 2004.

Peckhaus, A., Grass, S., Treuel, L., and Zellner, R.: Deliquescence and Efflorescence Behavior of Ternary Inorganic/Organic/Water Aerosol Particles, Journal of Physical Chemistry A, 116, 6199-6210, 2012.

Peng, C., Chan, M. N., and Chan, C. K.: The hygroscopic properties of dicarboxylic and multifunctional acids: Measurements and UNIFAC predictions, Environmental Science & Technology, 35, 4495-4501, 2001.

Pope, F. D., Dennis-Smither, B. J., Griffiths, P. T., Clegg, S. L., and Cox, R. A.: Studies of Single Aerosol Particles Containing Malonic Acid, Glutaric Acid, and Their Mixtures with Sodium Chloride. I. Hygroscopic Growth, Journal Of Physical Chemistry A, 114, 5335-5341, 2010.

Prenni, A. J., DeMott, P. J., Kreidenweis, S. M., Sherman, D. E., Russell, L. M., and Ming, Y.: The effects of low molecular weight dicarboxylic acids on cloud formation, Journal Of Physical Chemistry A, 105, 11240-11248, 2001.

Salcedo, D.: Equilibrium phase diagrams of aqueous mixtures of malonic acid and sulfate/ammonium salts, Journal of Physical Chemistry A, 110, 12158-12165, 2006.

Saxena, P., and Hildemann, L. M.: Water absorption by organics: Survey of laboratory evidence and evaluation of UNIFAC for estimating water activity, Environmental Science & Technology, 31, 3318-3324, 1997.

Shao, X., Zhang, Y., Pang, S.-F., and Zhang, Y.-H.: Vacuum FTIR observation on hygroscopic properties and phase transition of malonic acid aerosols, Chemical Physics, 483, 7-11, 2017.

Treuel, L., Pederzani, S., and Zellner, R.: Deliquescence behaviour and crystallisation of ternary ammonium sulfate/dicarboxylic acid/water aerosols, Physical Chemistry Chemical Physics, 11, 7976-7984, 2009.

Wise, M. E., Surratt, J. D., Curtis, D. B., Shilling, J. E., and Tolbert, M. A.: Hygroscopic growth of ammonium sulfate/dicarboxylic acids, Journal Of Geophysical Research-Atmospheres, 108, 2003.

Yeung, M. C., and Chan, C. K.: Water Content and Phase Transitions in Particles of Inorganic and Organic Species and their Mixtures Using Micro-Raman Spectroscopy, Aerosol Science and Technology, 44, 269-280, 2010.

## (CH_2_)_2_(COOH)_2_ (succinic acid)

| Reference | *T* (K) | *D* | DRH (%) | ERH (%) | Techniques/Comments |
| --- | --- | --- | --- | --- | --- |
| Saxena and Hildemann, 1997 | 298 | - | ≥98 | - | Estimated by UNIFAC |
| Peng et al., 2001 | 298 | - | 98.8 | - | Nonisopiestic method |
|  |  | 10~20 μm | >90 | 55.2-59.3 | EDB |
| Prenni et al., 2001 | 303 | 50, 100 nm | >92 | - | HTDMA |
| Brooks et al., 2002 | 277 | - | ≥95.0 | - | Nonisopiestic method |
|  | 297 |  | 91.0±3 |  |  |
| Wise et al., 2003 | 298 | - | 97.6 | - | Nonisopiestic method |
| Marcolli et al., 2004 | 298 | - | 99.1 | - | Nonisopiestic method |
| Parsons et al., 2004 | 243 | 2-40 μm | 99.9±2 | - | Optical microscopy |
|  | 245 |  | 100.4±2 |  |  |
|  | 254 |  | 100±2 |  |  |
|  | 264 |  | 99.5±2 |  |  |
|  | 274 |  | 100±2 |  |  |
|  | 282 |  | 100±2 |  |  |
|  | 293 |  | 99.4±2 |  |  |
| Clegg and Seinfeld, 2006 | 298 | - | 98.9 | - | Nonisopiestic method |
| Wex et al., 2007 | 295.5 | 200, 250 nm | 99±0.2 | - | H-DMA-OPC |
| Moore and Raymond, 2008 | 296 | 80 nm | >95 | - | HTDMA |
| Zamora et al., 2011 | 298 | - | 99.5±1.8 | - | Nonisopiestic method |
| Ma et al., 2013 | 278 | - | >95 | - | PSA |
| Jing et al., 2016 | 297 | 100 nm | >90 | - | HTDMA |

**Comments:**

A number of studies (Saxena and Hildemann, 1997; Peng et al., 2001; Prenni et al., 2001; Brooks et al., 2002; Wise et al., 2003; Marcolli et al., 2004; Parsons et al., 2004; Clegg and Seinfeld, 2006; Wex et al., 2007; Moore and Raymond, 2008; Zamora et al., 2011; Ma et al., 2013; Jing et al., 2016) measured the DRH of (CH_2_)_2_(COOH)_2_, and it was determined to be >90% at 278-303 K, showing good agreement. The DRH values of (CH_2_)_2_(COOH)_2_ at different temperatures were measured by two studies (Brooks et al., 2002; Parsons et al., 2004). Brooks et al. (2002) observed the deliquescence occurred at ≥95.0% at 277 K and 91.0±3% at 297 K; similarly, Parsons et al. (2004) found that the DRH (99-100%) of (CH_2_)_2_(COOH)_2_ did not change significantly with temperature (243-293 K).

The ERH of (CH_2_)_2_(COOH)_2_ was measured to be 55.2-59.3% at 298 K by Peng et al. (2001).

**Preferred values at 298 K for (CH_2_)_2_(COOH)_2_:**

DRH: >90%

ERH: 55-60%

**References:**

Brooks, S. D., Wise, M. E., Cushing, M., and Tolbert, M. A.: Deliquescence behavior of organic/ammonium sulfate aerosol, Geophysical Research Letters, 29, 2002.

Clegg, S. L., and Seinfeld, J. H.: Thermodynamic models of aqueous solutions containing inorganic electrolytes and dicarboxylic acids at 298.15 K. 1. The acids as nondissociating components, Journal of Physical Chemistry A, 110, 5692-5717, 2006.

Jing, B., Tong, S., Liu, Q., Li, K., Wang, W., Zhang, Y., and Ge, M.: Hygroscopic behavior of multicomponent organic aerosols and their internal mixtures with ammonium sulfate, Atmospheric Chemistry and Physics, 16, 4101-4118, 2016.

Ma, Q., Ma, J., Liu, C., Lai, C., and He, H.: Laboratory study on the hygroscopic behavior of external and internal C2-C4 dicarboxylic acid-NaCl mixtures, Environ Sci Technol, 47, 10381-10388, 2013.

Marcolli, C., Luo, B. P., and Peter, T.: Mixing of the organic aerosol fractions: Liquids as the thermodynamically stable phases, Journal of Physical Chemistry A, 108, 2216-2224, 2004.

Moore, R. H., and Raymond, T. M.: HTDMA analysis of multicomponent dicarboxylic acid aerosols with comparison to UNIFAC and ZSR, Journal of Geophysical Research, 113, 2008.

Parsons, M. T., Mak, J., Lipetz, S. R., and Bertram, A. K.: Deliquescence of malonic, succinic, glutaric, and adipic acid particles, Journal of Geophysical Research-Atmospheres, 109, 2004.

Peng, C., Chan, M. N., and Chan, C. K.: The hygroscopic properties of dicarboxylic and multifunctional acids: Measurements and UNIFAC predictions, Environmental Science & Technology, 35, 4495-4501, 2001.

Prenni, A. J., DeMott, P. J., Kreidenweis, S. M., Sherman, D. E., Russell, L. M., and Ming, Y.: The effects of low molecular weight dicarboxylic acids on cloud formation, Journal Of Physical Chemistry A, 105, 11240-11248, 2001.

Saxena, P., and Hildemann, L. M.: Water absorption by organics: Survey of laboratory evidence and evaluation of UNIFAC for estimating water activity, Environmental Science & Technology, 31, 3318-3324, 1997.

Wex, H., Ziese, M., Kiselev, A., Henning, S., and Stratmann, F.: Deliquescence and hygroscopic growth of succinic acid particles measured with LACIS, Geophysical Research Letters, 34, 2007.

Wise, M. E., Surratt, J. D., Curtis, D. B., Shilling, J. E., and Tolbert, M. A.: Hygroscopic growth of ammonium sulfate/dicarboxylic acids, Journal Of Geophysical Research-Atmospheres, 108, 2003.

Zamora, I. R., Tabazadeh, A., Golden, D. M., and Jacobson, M. Z.: Hygroscopic growth of common organic aerosol solutes, including humic substances, as derived from water activity measurements, Journal of Geophysical Research-Atmospheres, 116, 2011.

## (CH_2_)_3_(COOH)_2_ (glutaric acid)

| Reference | *T* (K) | *D* | DRH (%) | ERH (%) | Techniques/Comments |
| --- | --- | --- | --- | --- | --- |
| Saxena and Hildemann, 1997 | 298 | - | 89-99 | - | Estimated by UNIFAC |
| Cruz and Pandis, 2000 | 297 | 50-100 nm | 85±5 | - | HTDMA |
| Peng et al., 2001 | 298 | - | 88.0-88.5 | - | Nonisopiestic method |
|  |  | 10~20 μm | 83.5-85 | 29-33 | EDB |
| Prenni et al., 2001 | 303 | 50, 100 nm | n. o. | <5 | HTDMA |
| Brooks et al., 2002 | 277 | - | 94.2±3 | - | Nonisopiestic method |
|  | 297 |  | 87.5±3 |  |  |
| Choi and Chan, 2002 | 295 | 10-15 μm | 83-85.2 |  | EDB |
| Wise et al., 2003 | 298 | - | 88.9 | - | Nonisopiestic method |
| Marcolli et al., 2004 | 298 | - | 88.2 | - | Nonisopiestic method |
| Pant et al., 2004 | 293 | 2-20 μm | 89.5±2 | 22.5-36 | Optical microscopy |
| Parsons et al., 2004 | 243 | 2-40 μm | 100.9±2 | - | Optical microscopy |
|  | 245 |  | 99.5±2 |  |  |
|  | 254 |  | 97.7±2 |  |  |
|  | 263 |  | 96.3±2 |  |  |
|  | 273 |  | 95.5±2 |  |  |
|  | 282 |  | 94.2±2 |  |  |
|  | 291 |  | 91.0±2 |  |  |
|  | 293 |  | 88.2±2 |  |  |
| Clegg and Seinfeld, 2006 | 298 | - | 87.8 | - | Nonisopiestic method |
| Treuel et al., 2008 | 298 | ~50 μm | 80-85 | - | EDB |
| Zardini et al., 2008 | 291 | 10~50 μm | 90±1.5 | 18-43 | EDB |
| Treuel et al., 2009 | 298 | - | 89±2 | - | Optical microscopy |
| Yeung et al., 2009 | 295-298 | 10-30 μm | - | 30-35.5 | Optical microscopy |
|  | 295-300 | 10-30 μm | - | 22-37 |  |
|  | 297-300 | 9-25 | - | 30-38 |  |
| Pope et al., 2010 | 295 | 20-50 μm | 85-93 | 20-30 | EDB |
| Yeung and Chan, 2010 | 297 | 10-30 μm | 86±2 | <30 | Micro-Raman |
| Zamora et al., 2011 | 298 | - | 90.1±1.8 | - | Nonisopiestic method |
| Peckhaus et al., 2012 | 293 | - | 88±2 | - | Optical microscopy |
| Ghorai et al., 2014 | 298 | 0.2~1.2 μm | n. o. | - | Micro-FTIR |
| Wu et al., 2019 | 298 | 2-8 μm | 85.7 | 40.1 | Vaccum-FTIR |

**Comments:**

The DRH of (CH_2_)_3_(COOH)_2_ was measured by a number of studies (Saxena and Hildemann, 1997; Cruz and Pandis, 2000; Peng et al., 2001; Prenni et al., 2001; Brooks et al., 2002; Choi and Chan, 2002; Wise et al., 2003; Marcolli et al., 2004; Pant et al., 2004; Parsons et al., 2004; Clegg and Seinfeld, 2006; Treuel et al., 2008; Zardini et al., 2008; Treuel et al., 2009; Pope et al., 2010; Yeung and Chan, 2010; Zamora et al., 2011; Peckhaus et al., 2012; Ghorai et al., 2014; Wu et al., 2019). It was determined to be 80-93% at 291-298 K by most studies (Cruz and Pandis, 2000; Peng et al., 2001; Brooks et al., 2002; Choi and Chan, 2002; Wise et al., 2003; Marcolli et al., 2004; Pant et al., 2004; Parsons et al., 2004; Clegg and Seinfeld, 2006; Treuel et al., 2008; Zardini et al., 2008; Treuel et al., 2009; Pope et al., 2010; Yeung and Chan, 2010; Zamora et al., 2011; Peckhaus et al., 2012; Wu et al., 2019) , showing relatively good agreement. Saxena and Hildemann (1997) estimated the DRH of (CH_2_)_3_(COOH)_2_ to be 89-99% at 298 K, slightly higher than those (80-93%) reported by other studies; in addition, Prenni et al. (2001) and Ghorai et al. (2014) observed continuous water uptake with the increase in RH at 298-303 K, thus no DRH was reported.


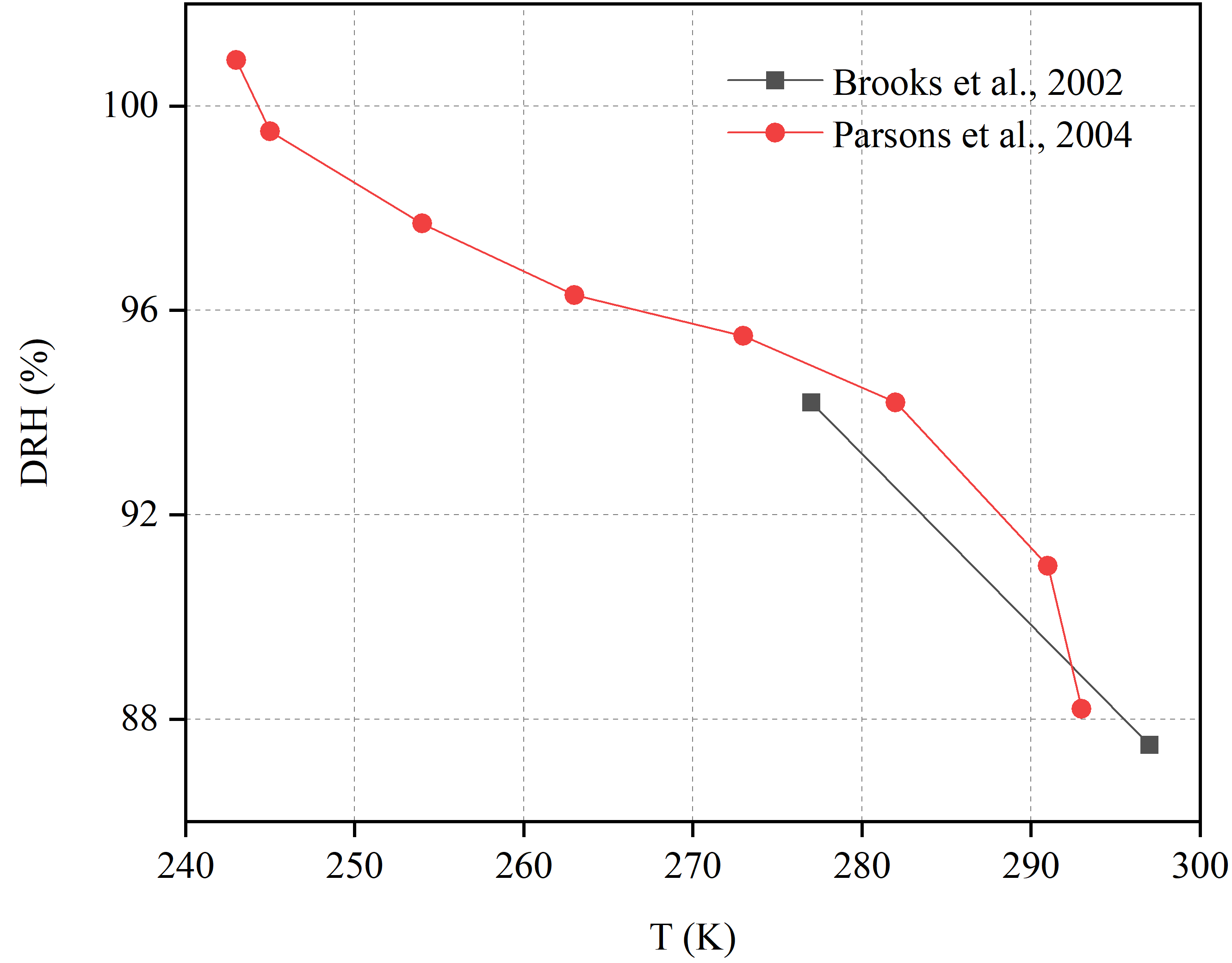


Two studies (Brooks et al., 2002; Parsons et al., 2004) measured the DRH of (CH_2_)_3_(COOH)_2_ at different temperatures, suggesting similar temperature dependence: Brooks et al. (2002) found that it decreased with temperature from 94.2±3% at 277 K to 87.5±3% at 297 K; and Parsons et al. (2004) suggested that it decreased with temperature from 100.9±2% at 243 K to 88.2±2% at 293 K.

Eight studies (Peng et al., 2001; Prenni et al., 2001; Pant et al., 2004; Zardini et al., 2008; Yeung et al., 2009; Pope et al., 2010; Yeung and Chan, 2010; Wu et al., 2019) measured the ERH of (CH_2_)_3_(COOH)_2_, and reported different results. Seven studies (Peng et al., 2001; Pant et al., 2004; Zardini et al., 2008; Yeung et al., 2009; Pope et al., 2010; Yeung and Chan, 2010; Wu et al., 2019) found that the efflorescence of (CH_2_)_3_(COOH)_2_ particles took place at 18-43% at 291-300 K, while Prenni et al. (2001) suggested that the efflorescence at 303 K occurred at <5% RH, significantly lower than those reported by the other studies.

**Preferred values at 298 K for (CH_2_)_3_(COOH)_2_:**

DRH: 80-93%

ERH: 18-43%

**References:**

Brooks, S. D., Wise, M. E., Cushing, M., and Tolbert, M. A.: Deliquescence behavior of organic/ammonium sulfate aerosol, Geophysical Research Letters, 29, 2002.

Choi, M. Y., and Chan, C. K.: Continuous measurements of the water activities of aqueous droplets of water-soluble organic compounds, Journal Of Physical Chemistry A, 106, 4566-4572, 2002.

Clegg, S. L., and Seinfeld, J. H.: Thermodynamic models of aqueous solutions containing inorganic electrolytes and dicarboxylic acids at 298.15 K. 1. The acids as nondissociating components, Journal of Physical Chemistry A, 110, 5692-5717, 2006.

Cruz, C. N., and Pandis, S. N.: Deliquescence and hygroscopic growth of mixed inorganic-organic atmospheric aerosol, Environmental Science & Technology, 34, 4313-4319, 2000.

Ghorai, S., Wang, B., Tivanski, A., and Laskin, A.: Hygroscopic Properties of Internally Mixed Particles Composed of NaCl and Water-Soluble Organic Acids, Environmental Science & Technology, 48, 2234-2241, 2014.

Marcolli, C., Luo, B. P., and Peter, T.: Mixing of the organic aerosol fractions: Liquids as the thermodynamically stable phases, Journal of Physical Chemistry A, 108, 2216-2224, 2004.

Pant, A., Fok, A., Parsons, M. T., Mak, J., and Bertram, A. K.: Deliquescence and crystallization of ammonium sulfate-glutaric acid and sodium chloride-glutaric acid particles, Geophysical Research Letters, 31, 2004.

Parsons, M. T., Mak, J., Lipetz, S. R., and Bertram, A. K.: Deliquescence of malonic, succinic, glutaric, and adipic acid particles, Journal of Geophysical Research-Atmospheres, 109, 2004.

Peckhaus, A., Grass, S., Treuel, L., and Zellner, R.: Deliquescence and Efflorescence Behavior of Ternary Inorganic/Organic/Water Aerosol Particles, Journal of Physical Chemistry A, 116, 6199-6210, 2012.

Peng, C., Chan, M. N., and Chan, C. K.: The hygroscopic properties of dicarboxylic and multifunctional acids: Measurements and UNIFAC predictions, Environmental Science & Technology, 35, 4495-4501, 2001.

Pope, F. D., Dennis-Smither, B. J., Griffiths, P. T., Clegg, S. L., and Cox, R. A.: Studies of Single Aerosol Particles Containing Malonic Acid, Glutaric Acid, and Their Mixtures with Sodium Chloride. I. Hygroscopic Growth, Journal Of Physical Chemistry A, 114, 5335-5341, 2010.

Prenni, A. J., DeMott, P. J., Kreidenweis, S. M., Sherman, D. E., Russell, L. M., and Ming, Y.: The effects of low molecular weight dicarboxylic acids on cloud formation, Journal Of Physical Chemistry A, 105, 11240-11248, 2001.

Saxena, P., and Hildemann, L. M.: Water absorption by organics: Survey of laboratory evidence and evaluation of UNIFAC for estimating water activity, Environmental Science & Technology, 31, 3318-3324, 1997.

Treuel, L., Pederzani, S., and Zellner, R.: Deliquescence behaviour and crystallisation of ternary ammonium sulfate/dicarboxylic acid/water aerosols, Physical Chemistry Chemical Physics, 11, 7976-7984, 2009.

Treuel, L., Schulze, S., Leisner, T., and Zellner, R.: Deliquescence behaviour of single levitated ternary salt/carboxylic acid/water microdroplets, Faraday Discussions, 137, 265-278, 2008.

Wise, M. E., Surratt, J. D., Curtis, D. B., Shilling, J. E., and Tolbert, M. A.: Hygroscopic growth of ammonium sulfate/dicarboxylic acids, Journal Of Geophysical Research-Atmospheres, 108, 2003.

Wu, F.-M., Wang, N., Pang, S.-F., and Zhang, Y.-H.: Hygroscopic behavior and fractional crystallization of mixed (NH_4_)_2_SO_4_/glutaric acid aerosols by vacuum FTIR, Spectrochimica Acta Part a-Molecular and Biomolecular Spectroscopy, 208, 255-261, 2019.

Yeung, M. C., and Chan, C. K.: Water Content and Phase Transitions in Particles of Inorganic and Organic Species and their Mixtures Using Micro-Raman Spectroscopy, Aerosol Science and Technology, 44, 269-280, 2010.

Yeung, M. C., Lee, A. K. Y., and Chan, C. K.: Phase Transition and Hygroscopic Properties of Internally Mixed Ammonium Sulfate and Adipic Acid (AS-AA) Particles by Optical Microscopic Imaging and Raman Spectroscopy, Aerosol Science and Technology, 43, 387-399, 2009.

Zamora, I. R., Tabazadeh, A., Golden, D. M., and Jacobson, M. Z.: Hygroscopic growth of common organic aerosol solutes, including humic substances, as derived from water activity measurements, Journal of Geophysical Research-Atmospheres, 116, 2011.

Zardini, A. A., Sjogren, S., Marcolli, C., Krieger, U. K., Gysel, M., Weingartner, E., Baltensperger, U., and Peter, T.: A combined particle trap/HTDMA hygroscopicity study of mixed inorganic/organic aerosol particles, Atmospheric Chemistry and Physics, 8, 5589-5601, 2008.

## (CH_2_)_4_(COOH)_2_ (adipic acid)

| Reference | *T* (K) | *D* | DRH (%) | ERH (%) | Techniques/Comments |
| --- | --- | --- | --- | --- | --- |
| Saxena and Hildemann, 1997 | 298 | - | ≥99 | - | Estimated by UNIFAC |
| Prenni et al., 2001 | 303 | 50, 100 nm | >93 | - | HTDMA |
| Brooks et al., 2002 | 277 | - | ≥95.0 | - | Nonisopiestic method |
|  | 297 |  | ≥95.0 |  |  |
| Hämeri et al., 2002 | 298 | 100 nm | >88 | - | HTDMA |
| Marcolli et al., 2004 | 298 | - | 99.9 | - | Nonisopiestic method |
| Parsons et al., 2004 | 243 | 2-40 μm | 100.0±2 | - | Optical microscopy |
|  | 246 |  | 100.0±2 |  |  |
|  | 254 |  | 100.1±2 |  |  |
|  | 264 |  | 100.6±2 |  |  |
|  | 274 |  | 100.0±2 |  |  |
|  | 282 |  | 100.7±2 |  |  |
|  | 292 |  | 100.6±2 |  |  |
| Chan et al., 2008 | 298 | 20~40 μm | >90 | >85 | EDB |
|  |  | - | 99.8 | - | Nonisopiestic method |
| Moore and Raymond, 2008 | 296 | 80 nm | >95 | - | HTDMA |
| Zardini et al., 2008 | 291 | 10~50 μm | >99 | 18-43 | EDB |
| Yeung et al., 2009 | 295-296 | 5-20 μm | - | ≥93 | Optical microscopy |

**Comments:**

The DRH of (CH_2_)_4_(COOH)_2_ was measured by nine studies (Saxena and Hildemann, 1997; Prenni et al., 2001; Brooks et al., 2002; Hämeri et al., 2002; Marcolli et al., 2004; Parsons et al., 2004; Chan et al., 2008; Moore and Raymond, 2008; Zardini et al., 2008). It was determined to be >88% at 291-303 K, suggesting good agreement among these studies. Moreover, two studies (Brooks et al., 2002; Parsons et al., 2004) measured the DRH of (CH_2_)_4_(COOH)_2_ at different temperatures. Both studies found that it did not change significantly with temperature, and their measured DRH was determined to be ≥95.0 at 277-297 K (Brooks et al., 2002) and ~100% at 243-292 K (Parsons et al., 2004).

Three studies (Chan et al., 2008; Zardini et al., 2008; Yeung et al., 2009) measured the ERH of (CH_2_)_4_(COOH)_2_, and reported different results. To be more specific, it was determined to be >85% at 298 K by Chan et al. (2008) and ≥93% at 295-296 K by Yeung et al. (2009), showing relatively good agreement; in the third study, Zardini et al. (2008) observed that the efflorescence of (CH_2_)_4_(COOH)_2_ took place at 18-43% at 291 K, significantly lower than those reported by the other two studies.

**Preferred values at 298 K for (CH_2_)_4_(COOH)_2_:**

DRH: >88%

ERH: no preferred value

**References:**

Brooks, S. D., Wise, M. E., Cushing, M., and Tolbert, M. A.: Deliquescence behavior of organic/ammonium sulfate aerosol, Geophysical Research Letters, 29, 2002.

Chan, M. N., Kreidenweis, S. M., and Chan, C. K.: Measurements of the hygroscopic and deliquescence properties of organic compounds of different solubilities in water and their relationship with cloud condensation nuclei activities, Environmental Science & Technology, 42, 3602-3608, 2008.

Hämeri, K., Charlson, R., and Hansson, H. C.: Hygroscopic properties of mixed ammonium sulfate and carboxylic acids particles, Aiche Journal, 48, 1309-1316, 2002.

Marcolli, C., Luo, B. P., and Peter, T.: Mixing of the organic aerosol fractions: Liquids as the thermodynamically stable phases, Journal of Physical Chemistry A, 108, 2216-2224, 2004.

Moore, R. H., and Raymond, T. M.: HTDMA analysis of multicomponent dicarboxylic acid aerosols with comparison to UNIFAC and ZSR, Journal of Geophysical Research, 113, 2008.

Parsons, M. T., Mak, J., Lipetz, S. R., and Bertram, A. K.: Deliquescence of malonic, succinic, glutaric, and adipic acid particles, Journal of Geophysical Research-Atmospheres, 109, 2004.

Prenni, A. J., DeMott, P. J., Kreidenweis, S. M., Sherman, D. E., Russell, L. M., and Ming, Y.: The effects of low molecular weight dicarboxylic acids on cloud formation, Journal of Physical Chemistry A, 105, 11240-11248, 2001.

Saxena, P., and Hildemann, L. M.: Water absorption by organics: Survey of laboratory evidence and evaluation of UNIFAC for estimating water activity, Environmental Science & Technology, 31, 3318-3324, 1997.

Yeung, M. C., Lee, A. K. Y., and Chan, C. K.: Phase Transition and Hygroscopic Properties of Internally Mixed Ammonium Sulfate and Adipic Acid (AS-AA) Particles by Optical Microscopic Imaging and Raman Spectroscopy, Aerosol Science and Technology, 43, 387-399, 2009.

Zardini, A. A., Sjogren, S., Marcolli, C., Krieger, U. K., Gysel, M., Weingartner, E., Baltensperger, U., and Peter, T.: A combined particle trap/HTDMA hygroscopicity study of mixed inorganic/organic aerosol particles, Atmospheric Chemistry and Physics, 8, 5589-5601, 2008.

## (CH_2_)_5_(COOH)_2_ (pimelic acid)

| Reference | *T* (K) | *D* | DRH (%) | ERH (%) | Techniques/Comments |
| --- | --- | --- | --- | --- | --- |
| Chan et al., 2008 | 298 | 20~40 μm | >90 | 51.5-53 | EDB |
|  |  | - | 99.5 | - | Nonisopiestic method |
| Miñambres et al., 2013 | 298 | - | >90 | - | FTIR |

**Comments:**

Two studies (Chan et al., 2008; Miñambres et al., 2013) measured the DRH of (CH_2_)_5_(COOH)_2_ at 298 K, and it was determined to be 99-99.5% by Chan et al. (2008) and >90% at 298 by Miñambres et al. (2013), showing good agreement.

The ERH of (CH_2_)_5_(COOH)_2_ was measured to be 51.5-53% at 298 K by Chan et al. (2008).

**Preferred values at 298 K for (CH_2_)_5_(COOH)_2_:**

DRH: >90%

ERH: 51-53%

**References:**

Chan, M. N., Kreidenweis, S. M., and Chan, C. K.: Measurements of the hygroscopic and deliquescence properties of organic compounds of different solubilities in water and their relationship with cloud condensation nuclei activities, Environmental Science & Technology, 42, 3602-3608, 2008.

Miñambres, L., Mendez, E., Sanchez, M. N., Castano, F., and Basterretxea, F. J.: Water uptake of internally mixed ammonium sulfate and dicarboxylic acid particles probed by infrared spectroscopy, Atmospheric Environment, 70, 108-116, 2013.

## (CH_2_)_6_(COOH)_2_ (suberic acid)

| Reference | *T* (K) | *D* | DRH (%) | ERH (%) | Techniques/Comments |
| --- | --- | --- | --- | --- | --- |
| Chan et al., 2008 | 298 | 20~40 μm | >90 | >85 | EDB |
|  |  | - | 99.9 | - | Nonisopiestic method |

**Comments:**

Chan et al. (2008) investigated hygroscopic property of (CH_2_)_6_(COOH)_2_, and found that the DRH and ERH of (CH_2_)_6_(COOH)_2_ were determined to be 90-99.9% and >85% at 298 K, respectively.

**Preferred values at 298 K for (CH_2_)_6_(COOH)_2_:**

DRH: >90%

ERH: >85%

**References:**

Chan, M. N., Kreidenweis, S. M., and Chan, C. K.: Measurements of the hygroscopic and deliquescence properties of organic compounds of different solubilities in water and their relationship with cloud condensation nuclei activities, Environmental Science & Technology, 42, 3602-3608, 2008.

## (CH_2_)_7_(COOH)_2_ (azelaic acid)

| Reference | *T* (K) | *D* | DRH (%) | ERH (%) | Techniques/Comments |
| --- | --- | --- | --- | --- | --- |
| Andrews and Larson, 1993 | 293-298 | 30 μm | >86 | - | EDB |
| Chan et al., 2008 | 298 | 20~40 μm | >90 | >85 | EDB |
|  |  | - | 99.9 | - | Nonisopiestic method |
| Moore and Raymond, 2008 | 296 | 80 nm | >95 | - | HTDMA |

**Comments:**

The DRH of (CH_2_)_7_(COOH)_2_ was measured by three studies (Andrews and Larson, 1993; Chan et al., 2008; Moore and Raymond, 2008) and it was determined to be >86% at 293-298 K, showing good agreement among these studies.

The ERH of (CH_2_)_7_(COOH)_2_ was measured to be >85% at 298 K by Chan et al. (2008).

**Preferred values at 298 K for (CH_2_)_7_(COOH)_2_:**

DRH: >86%

ERH: >85%

**References:**

Andrews, E., and Larson, S. M.: Effect of surfactant layers on the size changes of aerosol particles as a function of relative humidity, Environmental Science & Technology, 27, 857-865, 1993.

Chan, M. N., Kreidenweis, S. M., and Chan, C. K.: Measurements of the hygroscopic and deliquescence properties of organic compounds of different solubilities in water and their relationship with cloud condensation nuclei activities, Environmental Science & Technology, 42, 3602-3608, 2008.

Moore, R. H., and Raymond, T. M.: HTDMA analysis of multicomponent dicarboxylic acid aerosols with comparison to UNIFAC and ZSR, Journal of Geophysical Research, 113, 2008.

## C_2_H_2_(COOH)_2_ (maleic acid)

| Reference | *T* (K) | *D* | DRH (%) | ERH (%) | Techniques/Comments |
| --- | --- | --- | --- | --- | --- |
| Saxena and Hildemann, 1997 | 298 | - | 85-99 | - | Estimated by UNIFAC |
| Brooks et al., 2002 | 277 | - | 91.9±3 | - | Nonisopiestic method |
|  | 297 |  | 87.5±3 |  |  |
| Choi and Chan, 2002 | 295 | 10-15μm | 71-86 | 48-51 | EDB |
| Brooks et al., 2003 | 273 | 0.5~1 μm | 89 | ~18 | FTIR |
| Wise et al., 2003 | 298 | - | 88.9 | - | Nonisopiestic method |
| Marcolli et al., 2004 | 298 | - | 89.1 | - | Nonisopiestic method |
| Clegg and Seinfeld, 2006 | 298 | - | 88.5 | - | Nonisopiestic method |
| Moore and Raymond, 2008 | 296 | 80 nm | 83-85 | - | HTDMA |
| Treuel et al., 2008 | 298 | ~50 μm | 83±5 | - | EDB |
| Treuel et al., 2009 | 298 | - | 90±2 | - | Optical microscopy |

**Comments:**

The DRH of C_2_H_2_(COOH)_2_ was measured by ten studies (Saxena and Hildemann, 1997; Brooks et al., 2002; Choi and Chan, 2002; Brooks et al., 2003; Wise et al., 2003; Marcolli et al., 2004; Clegg and Seinfeld, 2006; Moore and Raymond, 2008; Treuel et al., 2008; Treuel et al., 2009). It was determined to be 71-99% at 273-298 K by these studies, suggesting relatively good agreement. In addition, Brooks et al. (2002) measured the DRH of C_2_H_2_(COOH)_2_ at different temperatures, and found that it decreased slightly with temperature from 91.9±3% at 277 K to 87.5±3% at 297 K.

Two studies (Choi and Chan, 2002; Brooks et al., 2003) measured the ERH of C_2_H_2_(COOH)_2_, and reported different results. Choi and Chan (2002) found that the efflorescence took place at 48-51% at 295 K; while Brooks et al. (2003) observed that the C_2_H_2_(COOH)_2_ particles effloresced at ~18% at 273 K, significantly lower than that reported by Choi and Chan (2002).

**Preferred values at 298 K for C_2_H_2_(COOH)_2_:**

DRH: 71-99%

ERH: 18-51%

**References:**

Brooks, S. D., Wise, M. E., Cushing, M., and Tolbert, M. A.: Deliquescence behavior of organic/ammonium sulfate aerosol, Geophysical Research Letters, 29, 2002.

Brooks, S. D., Garland, R. M., Wise, M. E., Prenni, A. J., Cushing, M., Hewitt, E., and Tolbert, M. A.: Phase changes in internally mixed maleic acid/ammonium sulfate aerosols, Journal of Geophysical Research-Atmospheres, 108, 2003.

Choi, M. Y., and Chan, C. K.: Continuous measurements of the water activities of aqueous droplets of water-soluble organic compounds, Journal Of Physical Chemistry A, 106, 4566-4572, 2002.

Clegg, S. L., and Seinfeld, J. H.: Thermodynamic models of aqueous solutions containing inorganic electrolytes and dicarboxylic acids at 298.15 K. 1. The acids as nondissociating components, Journal of Physical Chemistry A, 110, 5692-5717, 2006.

Marcolli, C., Luo, B. P., and Peter, T.: Mixing of the organic aerosol fractions: Liquids as the thermodynamically stable phases, Journal of Physical Chemistry A, 108, 2216-2224, 2004.

Moore, R. H., and Raymond, T. M.: HTDMA analysis of multicomponent dicarboxylic acid aerosols with comparison to UNIFAC and ZSR, Journal of Geophysical Research, 113, 2008.

Saxena, P., and Hildemann, L. M.: Water absorption by organics: Survey of laboratory evidence and evaluation of UNIFAC for estimating water activity, Environmental Science & Technology, 31, 3318-3324, 1997.

Treuel, L., Schulze, S., Leisner, T., and Zellner, R.: Deliquescence behaviour of single levitated ternary salt/carboxylic acid/water microdroplets, Faraday Discussions, 137, 265-278, 2008.

Treuel, L., Pederzani, S., and Zellner, R.: Deliquescence behaviour and crystallisation of ternary ammonium sulfate/dicarboxylic acid/water aerosols, Physical Chemistry Chemical Physics, 11, 7976-7984, 2009.

Wise, M. E., Surratt, J. D., Curtis, D. B., Shilling, J. E., and Tolbert, M. A.: Hygroscopic growth of ammonium sulfate/dicarboxylic acids, Journal Of Geophysical Research-Atmospheres, 108, 2003.

## (CH_2_CHOH)(COOH)_2_ (malic acid)

| Reference | *T* (K) | *D* | DRH (%) | ERH (%) | Techniques/Comments |
| --- | --- | --- | --- | --- | --- |
| Apelblat et al., 1995 | 288 | - | 82.6 | - | Nonisopiestic method (DL-) |
|  | 293 |  | 80.1 |  |  |
|  | 298 |  | 78.3 |  |  |
|  | 303 |  | 76.7 |  |  |
|  | 308 |  | 75.7 |  |  |
|  | 313 |  | 74.9 |  |  |
|  | 318 |  | 74.5 |  |  |
|  | 323 |  | 74.3 |  |  |
| Peng et al., 2001 | 298 | 10~20 μm | n. o. | n. o. | EDB (DL-) |
| Brooks et al., 2002 | 277 | - | 66.0±3 | - | Nonisopiestic method (L-) |
|  | 297 |  | 58.9±3 |  |  |
| Wise et al., 2003 | 298 | - | 57.6 | - | Nonisopiestic method (L-) |
| Marcolli et al., 2004 | 298 | - | 80.5 | - | Nonisopiestic method (DL-) |
| Clegg and Seinfeld, 2006 | 298 | - | 78.6 | - | Nonisopiestic method (DL-) |
|  |  |  | 55.9 |  | Nonisopiestic method (L-) |

**Comments:**

Six studies (Apelblat et al., 1995; Peng et al., 2001; Brooks et al., 2002; Wise et al., 2003; Marcolli et al., 2004; Clegg and Seinfeld, 2006) measured the DRH of (CH_2_CHOH)(COOH)_2_, and showed that it was closely related to the configurations of malic acid enantiomer.

For D,L-malic acid, the DRH was measured to be 78-81% at 298 K by three studies (Apelblat et al., 1995; Marcolli et al., 2004; Clegg and Seinfeld, 2006); in contrast, Peng et al. (2001) observed continuous water uptake for D,L-malic acid with increase in RH, and thus no DRH was reported. In addition, Apelblat et al. (1995) measured the DRH of D,L-malic acid at different temperatures, and found that it decreased with temperature from 82.6% at 288 K to 74.3% at 323 K.


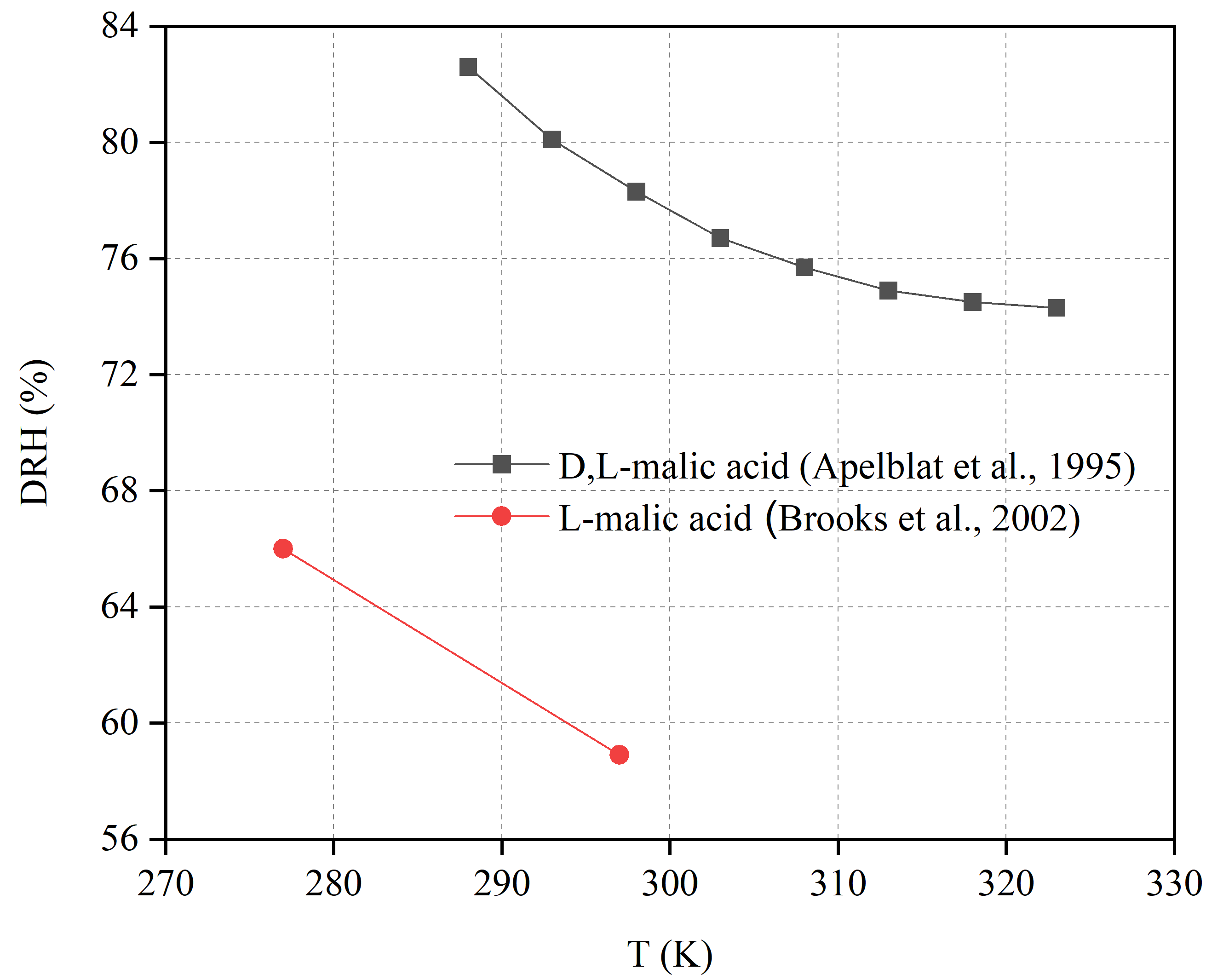


For L-malic acid, the DRH was determined to be 56-62% at 297-298 K by three studies (Brooks et al., 2002; Wise et al., 2003; Clegg and Seinfeld, 2006), showing good agreement. Moreover, Brooks et al. (2002) measured the DRH of L-malic acid at different temperatures, and found that it decreased with temperature from 66.0±3% at 277 K to 58.9±3% at 297 K.

**Preferred values at 298 K for D,L-malic acid:**

DRH: 78-81%

ERH: no preferred value

**Preferred values at 298 K for L-malic acid:**

DRH: 56-62%

ERH: no preferred value

**References:**

Apelblat, A., Dov, M., Wisniak, J., and Zabicky, J.: The vapour pressure of water over saturated aqueous solutions of malic, tartaric, and citric acids, at temperatures from 288 K to 323 K, Journal of Chemical Thermodynamics, 27, 35-41, 1995.

Brooks, S. D., Wise, M. E., Cushing, M., and Tolbert, M. A.: Deliquescence behavior of organic/ammonium sulfate aerosol, Geophysical Research Letters, 29, 2002.

Clegg, S. L., and Seinfeld, J. H.: Thermodynamic models of aqueous solutions containing inorganic electrolytes and dicarboxylic acids at 298.15 K. 1. The acids as nondissociating components, Journal of Physical Chemistry A, 110, 5692-5717, 2006.

Marcolli, C., Luo, B. P., and Peter, T.: Mixing of the organic aerosol fractions: Liquids as the thermodynamically stable phases, Journal of Physical Chemistry A, 108, 2216-2224, 2004.

Peng, C., Chan, M. N., and Chan, C. K.: The hygroscopic properties of dicarboxylic and multifunctional acids: Measurements and UNIFAC predictions, Environmental Science & Technology, 35, 4495-4501, 2001.

Wise, M. E., Surratt, J. D., Curtis, D. B., Shilling, J. E., and Tolbert, M. A.: Hygroscopic growth of ammonium sulfate/dicarboxylic acids, Journal of Geophysical Research-Atmospheres, 108, 2003.

## (CHOH)_2_(COOH)_2_ (tartaric acid)

| Reference | *T* (K) | *D* | DRH (%) | ERH (%) | Techniques/Comments |
| --- | --- | --- | --- | --- | --- |
| Apelblat et al., 1995 | 288 | - | 81.3 | - | Nonisopiestic method |
|  | 293 |  | 79.2 |  |  |
|  | 298 |  | 77.5 |  |  |
|  | 303 |  | 76.1 |  |  |
|  | 308 |  | 75.0 |  |  |
|  | 313 |  | 74.1 |  |  |
|  | 318 |  | 73.6 |  |  |
|  | 323 |  | 73.2 |  |  |
| Peng et al., 2001 | 298 | 10~20 μm | n. o. | n. o. | EDB |
| Moore and Raymond, 2008 | 296 | 80 nm | n. o. | - | HTDMA |

**Comments:**

The DRH of (CHOH)_2_(COOH)_2_ was measured by three studies (Apelblat et al., 1995; Peng et al., 2001; Moore and Raymond, 2008). Apelblat et al. (1995) found that it decreased with temperature from 81.3% at 288 K to 77.5% at 298 and to 73.2% at 323 K; on the contrary, the other two studies (Peng et al., 2001; Moore and Raymond, 2008) observed gradual water uptake with increase in RH, and thus no DRH was reported.

No efflorescence for (CHOH)_2_(COOH)_2_ particles was observed at 298 K (Peng et al., 2001), and thus no ERH was reported.

**Preferred values at 298 K for (CHOH)_2_(COOH)_2_:**

DRH: 77-78%

ERH: no preferred value

**References:**

Apelblat, A., Dov, M., Wisniak, J., and Zabicky, J.: The vapour pressure of water over saturated aqueous solutions of malic, tartaric, and citric acids, at temperatures from 288 K to 323 K, Journal of Chemical Thermodynamics, 27, 35-41, 1995.

Moore, R. H., and Raymond, T. M.: HTDMA analysis of multicomponent dicarboxylic acid aerosols with comparison to UNIFAC and ZSR, Journal of Geophysical Research, 113, 2008.

Peng, C., Chan, M. N., and Chan, C. K.: The hygroscopic properties of dicarboxylic and multifunctional acids: Measurements and UNIFAC predictions, Environmental Science & Technology, 35, 4495-4501, 2001.

## C_6_H_4_(COOH)_2_ (phthalic acid)

| Reference | *T* (K) | *D* | DRH (%) | ERH (%) | Techniques/Comments |
| --- | --- | --- | --- | --- | --- |
| Hämeri et al., 2002 | 298 | 100 nm | >88 | - | HTDMA |
| Brooks et al., 2004 | 303 | 100 nm | n. o. | - | HTDMA |
| Moore and Raymond, 2008 | 296 | 80 nm | >95 | - | HTDMA |
| Zamora et al., 2011 | 298 | - | >99.5 | - | Nonisopiestic method |
| Jing et al., 2016 | 297 | 100 nm | n. o. | - | HTDMA |

**Comments:**

Five studies (Hämeri et al., 2002; Brooks et al., 2004; Moore and Raymond, 2008; Zamora et al., 2011; Jing et al., 2016) measured the DRH of C_6_H_4_(COOH)_2_. Three studies (Hämeri et al., 2002; Moore and Raymond, 2008; Zamora et al., 2011) showed relatively good agreement, suggesting that the deliquescence of C_6_H_4_(COOH)_2_ took place at >88% RH at 296-298 K; in contrast, two other studies (Brooks et al., 2004; Jing et al., 2016) observed continuous water uptake for C_6_H_4_(COOH)_2_ particles with increase in RH, and thus no DRH was reported.

**Preferred values at 298 K for C_6_H_4_(COOH)_2_:**

DRH: >88%

ERH: no preferred value

**References:**

Brooks, S. D., DeMott, P. J., and Kreidenweis, S. M.: Water uptake by particles containing humic materials and mixtures of humic materials with ammonium sulfate, Atmospheric Environment, 38, 1859-1868, 2004.

Hämeri, K., Charlson, R., and Hansson, H. C.: Hygroscopic properties of mixed ammonium sulfate and carboxylic acids particles, Aiche Journal, 48, 1309-1316, 2002.

Jing, B., Tong, S., Liu, Q., Li, K., Wang, W., Zhang, Y., and Ge, M.: Hygroscopic behavior of multicomponent organic aerosols and their internal mixtures with ammonium sulfate, Atmospheric Chemistry and Physics, 16, 4101-4118, 2016.

Moore, R. H., and Raymond, T. M.: HTDMA analysis of multicomponent dicarboxylic acid aerosols with comparison to UNIFAC and ZSR, Journal of Geophysical Research, 113, 2008.

Zamora, I. R., Tabazadeh, A., Golden, D. M., and Jacobson, M. Z.: Hygroscopic growth of common organic aerosol solutes, including humic substances, as derived from water activity measurements, Journal of Geophysical Research-Atmospheres, 116, 2011.

# Dicarboxylic salts

## (NH_4_)_2_C_2_O_4_ (ammonium oxalate)

| Reference | *T* (K) | *D* | DRH (%) | ERH (%) | Techniques/Comments |
| --- | --- | --- | --- | --- | --- |
| Peng and Chan, 2001 | 298 | ~20 μm | >94 | 53.5-55.9 | EDB |
| Apelblat and Korin, 2003 | 278 | - | 90.8 | - | Nonisopiestic method |
|  | 283 |  | 90.6 |  |  |
|  | 288 |  | 90.3 |  |  |
|  | 293 |  | 89.8 |  |  |
|  | 298 |  | 89.4 |  |  |
|  | 303 |  | 88.8 |  |  |
|  | 308 |  | 88.2 |  |  |
|  | 313 |  | 87.6 |  |  |
|  | 318 |  | 86.9 |  |  |
|  | 323 |  | 86.1 |  |  |
| Moore and Raymond, 2008 | 296 | 80 nm | >95 | - | HTDMA |
| Mensah et al., 2009 | - | - | 93.3±1.4 | n. o. | HTDMA |
| Wu et al., 2011 | 293 | 100 nm | >90 | - | HTDMA |
| Ma et al., 2013 | 278 | - | >95 | - | PSA |
| Boreddy and Kawamura, 2018 | 290 | 100 nm | ~72 | - | HTDMA |

**Comments:**

Seven studies (Peng and Chan, 2001; Apelblat and Korin, 2003; Moore and Raymond, 2008; Mensah et al., 2009; Wu et al., 2011; Ma et al., 2013; Boreddy and Kawamura, 2018) measured the DRH of (NH_4_)_2_C_2_O_4_. Six studies (Peng and Chan, 2001; Apelblat and Korin, 2003; Moore and Raymond, 2008; Mensah et al., 2009; Wu et al., 2011; Ma et al., 2013) showed good agreement, and they suggested that the deliquescence of (NH_4_)_2_C_2_O_4_ occurred at >90% RH at 278-298 K; in the seventh study (Boreddy and Kawamura, 2018), the DRH of (NH_4_)_2_C_2_O_4_ was measured to be ~72% at 290 K, significantly lower than those reported by the other six studies. Moreover, Apelblat and Korin (2003) measured the DRH of (NH_4_)_2_C_2_O_4_ at different temperatures, and found that it decreased with temperature from 90.8% at 278 K to 86.1% at 323 K.

Two studies (Peng and Chan, 2001; Mensah et al., 2009) measured the ERH of (NH_4_)_2_C_2_O_4_, and reported different results: it was determined to be 53.5-55.9% at 298 K by Peng and Chan (2001); in contrast, Mensah et al. (2009) observed continuous water evaporation for (NH_4_)_2_C_2_O_4_ particles with decrease in RH, and thus no ERH was reported.

**Preferred values at 298 K for (NH_4_)_2_C_2_O_4_:**

DRH: >90%

ERH: 53-56%

**References:**

Apelblat, A., and Korin, E.: The molar enthalpies of solution and vapour pressures of saturated aqueous solutions of some ammonium salts, Journal of Chemical Thermodynamics, 35, 699-709, 2003.

Boreddy, S. K. R., and Kawamura, K.: Investigation on the hygroscopicity of oxalic acid and atmospherically relevant oxalate salts under sub- and supersaturated conditions, Environmental Science-Processes & Impacts, 20, 1069-1080, 2018.

Ma, Q., He, H., and Liu, C.: Hygroscopic properties of oxalic acid and atmospherically relevant oxalates, Atmospheric Environment, 69, 281-288, 2013.

Mensah, A. A., Buchholz, A., Kiendler-Scharr, A., and Mentel, T. F.: Chemical and physical properties of oxalic acid and oxalate aerosol particles, European Aerosol Conference, Karlsruhe, 2009.

Moore, R. H., and Raymond, T. M.: HTDMA analysis of multicomponent dicarboxylic acid aerosols with comparison to UNIFAC and ZSR, Journal of Geophysical Research, 113, 2008.

Peng, C. G., and Chan, C. K.: The water cycles of water-soluble organic salts of atmospheric importance, Atmospheric Environment, 35, 1183-1192, 2001.

Wu, Z. J., Nowak, A., Poulain, L., Herrmann, H., and Wiedensohler, A.: Hygroscopic behavior of atmospherically relevant water-soluble carboxylic salts and their influence on the water uptake of ammonium sulfate, Atmospheric Chemistry and Physics, 11, 12617-12626, 2011.

## (NH_4_)_2_C_2_O_4_·H_2_O (ammonium oxalate monohydrate)

| Reference | *T* (K) | *D* | DRH (%) | ERH (%) | Techniques/Comments |
| --- | --- | --- | --- | --- | --- |
| Schroeder and Beyer, 2016 | 288 | - | >95 | - | VSA |
|  | 298 |  | >95 |  |  |
|  | 308 |  | >95 |  |  |

**Comments:**

Schroeder and Beyer (2016) measured the DRH of (NH_4_)_2_C_2_O_4_·H_2_O as a function of temperature, and it was determined to be >95% at 288-308 K.

**Preferred values at 298 K for (NH_4_)_2_C_2_O_4_·H_2_O:**

DRH: >95%

ERH: no preferred value

**References:**

Schroeder, J. R., and Beyer, K. D.: Deliquescence Relative Humidities of Organic and Inorganic Salts Important in the Atmosphere, Journal of Physical Chemistry A, 120, 9948-9957, 2016.

## Na_2_C_2_O_4_ (sodium oxalate)

| Reference | *T* (K) | *D* | DRH (%) | ERH (%) | Techniques/Comments |
| --- | --- | --- | --- | --- | --- |
| Peng and Chan, 2001 | 298 | ~20 μm | >93 | 72.0-75.2 | EDB |
| Mensah et al., 2009 | - | - | 92.2±1.4 | 83.3±1.0 | HTDMA |
| Wu et al., 2011 | 293 | 100 nm | >90 | - | HTDMA |
| Ma et al., 2013 | 278 | - | >95 | - | PSA |
| Schroeder and Beyer, 2016 | 288 | - | 75.4 | - | VSA |
|  | 298 |  | 75.3 |  |  |
|  | 308 |  | 75.6 |  |  |
| Boreddy and Kawamura, 2018 | 290 | 100 nm | >94 | - | HTDMA |
| Gao et al., 2018 | 270-296 | ~0.7 μm | ≥100 | - | ATR-FTIR |
| Ma et al., 2019 | 298 | 300 nm | >90 | - | HTDMA |
| Wang et al., 2019 | 298 | <10 μm | >90 | 75.6-77.4 | ATR-FTIR |

**Comments:**

The DRH of Na_2_C_2_O_4_ was measured by nine studies (Peng and Chan, 2001; Mensah et al., 2009; Wu et al., 2011; Ma et al., 2013; Schroeder and Beyer, 2016; Boreddy and Kawamura, 2018; Gao et al., 2018; Ma et al., 2019; Wang et al., 2019). It was determined to be >90% at 270-298 K by eight studies (Peng and Chan, 2001; Mensah et al., 2009; Wu et al., 2011; Ma et al., 2013; Boreddy and Kawamura, 2018; Gao et al., 2018; Ma et al., 2019; Wang et al., 2019), showing good agreement. In addition, Schroeder and Beyer (2016) found that the DRH of Na_2_C_2_O_4_ did not change significantly with temperature (288-308 K) , and their measured DRH at 298 K (75.3%) was significantly lower than those (>90%) reported by the other studies.

Three studies (Peng and Chan, 2001; Mensah et al., 2009; Wang et al., 2019) measured the ERH of Na_2_C_2_O_4_, and it was determined to be 72-84% at 298 K, showing relatively good agreement.

**Preferred values at 298 K for Na_2_C_2_O_4_:**

DRH: >90%

ERH: 72-84%

**References:**

Boreddy, S. K. R., and Kawamura, K.: Investigation on the hygroscopicity of oxalic acid and atmospherically relevant oxalate salts under sub- and supersaturated conditions, Environmental Science-Processes & Impacts, 20, 1069-1080, 2018.

Gao, X., Zhang, Y., and Liu, Y.: Temperature-dependent hygroscopic behaviors of atmospherically relevant water-soluble carboxylic acid salts studied by ATR-FTIR spectroscopy, Atmospheric Environment, 191, 312-319, 2018.

Ma, Q., Ma, J., Liu, C., Lai, C., and He, H.: Laboratory study on the hygroscopic behavior of external and internal C2-C4 dicarboxylic acid-NaCl mixtures, Environ Sci Technol, 47, 10381-10388, 2013.

Ma, Q., Zhong, C., Liu, C., Liu, J., Ma, J., Wu, L., and He, H.: A Comprehensive Study about the Hygroscopic Behavior of Mixtures of Oxalic Acid and Nitrate Salts: Implication for the Occurrence of Atmospheric Metal Oxalate Complex, Acs Earth and Space Chemistry, 3, 1216-1225, 2019.

Mensah, A. A., Buchholz, A., Kiendler-Scharr, A., and Mentel, T. F.: Chemical and physical properties of oxalic acid and oxalate aerosol particles, European Aerosol Conference, Karlsruhe, 2009.

Peng, C. G., and Chan, C. K.: The water cycles of water-soluble organic salts of atmospheric importance, Atmospheric Environment, 35, 1183-1192, 2001.

Schroeder, J. R., and Beyer, K. D.: Deliquescence Relative Humidities of Organic and Inorganic Salts Important in the Atmosphere, Journal of Physical Chemistry A, 120, 9948-9957, 2016.

Wang, N., Jing, B., Wang, P., Wang, Z., Li, J., Pang, S., Zhang, Y., and Ge, M.: Hygroscopicity and Compositional Evolution of Atmospheric Aerosols Containing Water-Soluble Carboxylic Acid Salts and Ammonium Sulfate: Influence of Ammonium Depletion, Environmental Science & Technology, 53, 6225-6234, 2019.

Wu, Z. J., Nowak, A., Poulain, L., Herrmann, H., and Wiedensohler, A.: Hygroscopic behavior of atmospherically relevant water-soluble carboxylic salts and their influence on the water uptake of ammonium sulfate, Atmospheric Chemistry and Physics, 11, 12617-12626, 2011.

## K_2_C_2_O_4_ (potassium oxalate)

| Reference | *T* (K) | *D* | DRH (%) | ERH (%) | Techniques/Comments |
| --- | --- | --- | --- | --- | --- |
| Mensah et al., 2009 | - | - | 84.8±1.0 | 50.5±1.0 | HTDMA |
| Boreddy and Kawamura, 2018 | 290 | 100 nm | 85 | 56 | HTDMA |

**Comments:**

Two studies (Mensah et al., 2009; Boreddy and Kawamura, 2018) measured the DRH of K_2_C_2_O_4_. It was determined to be 84.8±1.0% by Mensah et al. (2009) and 85% at 290 K by Boreddy and Kawamura (2018), showing good agreement.

Mensah et al. (2009) and Boreddy and Kawamura (2018) also measured the ERH of K_2_C_2_O_4_, and it was determined to be 49-56% at 290 K.

**Preferred values at 298 K for K_2_C_2_O_4_:**

DRH: 84-86%

ERH: 49-56%

**References:**

Boreddy, S. K. R., and Kawamura, K.: Investigation on the hygroscopicity of oxalic acid and atmospherically relevant oxalate salts under sub- and supersaturated conditions, Environmental Science-Processes & Impacts, 20, 1069-1080, 2018.

Mensah, A. A., Buchholz, A., Kiendler-Scharr, A., and Mentel, T. F.: Chemical and physical properties of oxalic acid and oxalate aerosol particles, European Aerosol Conference, Karlsruhe, 2009.

## CaC_2_O_4_ (calcium oxalate) and CaC_2_O_4_·H_2_O (calcium oxalate monohydrate)

| Species | Reference | *T* (K) | *D* | DRH (%) | ERH (%) | Techniques/Comments |
| --- | --- | --- | --- | --- | --- | --- |
| CaC_2_O_4_ | Ma et al., 2013 | 278 | - | >95 | - | PSA |
|  | Ma et al., 2019 | 298 | 300 nm | >90 | - | HTDMA |
| CaC_2_O_4_·H_2_O | Ma et al., 2013 | 278 | - | >95 | - | PSA |

**Comments:**

The DRH of CaC_2_O_4_ was measured to be >90% at 278-298 K by two studies (Ma et al., 2013; Ma et al., 2019).

The DRH of CaC_2_O_4_·H_2_O was measured to be >95% at 278 K by Ma et al. (2013).

**Preferred values at 298 K for CaC_2_O_4_:**

DRH: >90%

ERH: no preferred value

**Preferred values at 298 K for CaC_2_O_4_·H_2_O:**

DRH: >95%

ERH: no preferred value

**References:**

Ma, Q., He, H., and Liu, C.: Hygroscopic properties of oxalic acid and atmospherically relevant oxalates, Atmospheric Environment, 69, 281-288, 2013.

Ma, Q., Zhong, C., Liu, C., Liu, J., Ma, J., Wu, L., and He, H.: A Comprehensive Study about the Hygroscopic Behavior of Mixtures of Oxalic Acid and Nitrate Salts: Implication for the Occurrence of Atmospheric Metal Oxalate Complex, Acs Earth and Space Chemistry, 3, 1216-1225, 2019.

## Na_2_C_3_H_2_O_4_ (sodium malonate) and Na_2_C_3_H_2_O_4_·H_2_O (sodium malonate monohydrate)

| Species | Reference | *T* (K) | *D* | DRH (%) | ERH (%) | Techniques/Comments |
| --- | --- | --- | --- | --- | --- | --- |
| Na_2_C_3_H_2_O_4_ | Peng and Chan, 2001 | 298 | ~20 μm | n. o. | n. o. | EDB |
|  | Wu et al., 2011 | 293 | 100 nm | n. o. | - | HTDMA |
|  | Ma et al., 2013 | 278 | - | n. o. | - | PSA |
|  | Wang et al., 2019 | 298 | <10 μm | n. o. | n. o. | ATR-FTIR |
| Na_2_C_3_H_2_O_4_·H_2_O | Schroeder and Beyer, 2016 | 288 | - | 66.5 | - | VSA |
|  |  | 298 |  | 65.3 |  |  |
|  |  | 308 |  | 64.7 |  |  |

**Comments:**

The DRH of Na_2_C_3_H_2_O_4_ was measured by four studies (Peng and Chan, 2001; Wu et al., 2011; Ma et al., 2013; Wang et al., 2019); they all observed continuous water uptake with the increase in RH at 278-298 K, and thus no DRH was reported. In addition, two studies (Peng and Chan, 2001; Wang et al., 2019) observed continuous loss of water for Na_2_C_3_H_2_O_4_ with the decrease in RH at 298 K, and thus no ERH was reported.

The DRH of Na_2_C_3_H_2_O_4_·H_2_O was measured by Schroeder and Beyer (2016). It was determined to decrease slightly with temperature from 66.5% at 288 K to 65.3% at 298 K and to 64.7% at 308 K.

**Preferred values at 298 K for Na_2_C_3_H_2_O_4_:**

DRH: no preferred value

ERH: no preferred value

**Preferred values at 298 K for Na_2_C_3_H_2_O_4_·H_2_O:**

DRH: 65-66%

ERH: no preferred value

**References:**

Ma, Q., Ma, J., Liu, C., Lai, C., and He, H.: Laboratory study on the hygroscopic behavior of external and internal C2-C4 dicarboxylic acid-NaCl mixtures, Environ Sci Technol, 47, 10381-10388, 2013.

Peng, C. G., and Chan, C. K.: The water cycles of water-soluble organic salts of atmospheric importance, Atmospheric Environment, 35, 1183-1192, 2001.

Schroeder, J. R., and Beyer, K. D.: Deliquescence Relative Humidities of Organic and Inorganic Salts Important in the Atmosphere, Journal of Physical Chemistry A, 120, 9948-9957, 2016.

Wang, N., Jing, B., Wang, P., Wang, Z., Li, J., Pang, S., Zhang, Y., and Ge, M.: Hygroscopicity and Compositional Evolution of Atmospheric Aerosols Containing Water-Soluble Carboxylic Acid Salts and Ammonium Sulfate: Influence of Ammonium Depletion, Environmental Science & Technology, 53, 6225-6234, 2019.

Wu, Z. J., Nowak, A., Poulain, L., Herrmann, H., and Wiedensohler, A.: Hygroscopic behavior of atmospherically relevant water-soluble carboxylic salts and their influence on the water uptake of ammonium sulfate, Atmospheric Chemistry and Physics, 11, 12617-12626, 2011.

## Na_2_C_4_H_4_O_4_ (sodium succinate)

| Reference | *T* (K) | *D* | DRH (%) | ERH (%) | Techniques/Comments |
| --- | --- | --- | --- | --- | --- |
| Peng and Chan, 2001 | 298 | ~20 μm | 63.5-66.0 | 46.7-47.9 | EDB |
| Wu et al., 2011 | 293 | 100 nm | n. o. | - | HTDMA |
| Ma et al., 2013 | 278 | - | 50 | - | PSA |
| Gao et al., 2018 | 270 | ~0.7 μm | 55-61 | 34-38 | ATR-FTIR |
|  | 278 |  | 59-65 | 39-43 |  |
|  | 284 |  | 68-74 | 47-52 |  |
|  | 290 |  | 72-78 | 49-56 |  |
|  | 296 |  | 74-79 | 50-57 |  |
| Wang et al., 2019 | 298 | < 10 μm | 69.5 | 57.7-59.3 | ATR-FTIR |

**Comments:**

The DRH of Na_2_C_4_H_4_O_4_ was measured by five studies (Peng and Chan, 2001; Wu et al., 2011; Ma et al., 2013; Gao et al., 2018; Wang et al., 2019). Three studies (Peng and Chan, 2001; Gao et al., 2018; Wang et al., 2019) suggested that the deliquescence of Na_2_C_4_H_4_O_4_ took place at 63-79% at 296-298 K, while Ma et al. (2013) found that the deliquescence took place at 50% RH at 278 K; in addition, Wu et al. (2011) observed continuous water uptake for Na_2_C_4_H_4_O_4_ at 293 K, and thus no DRH was reported. Gao et al. (2018) also measured the DRH of Na_2_C_4_H_4_O_4_ at different temperatures, and suggested that it increased with temperature from 55-61% at 270 K to 74-79% at 296 K.

Three studies (Peng and Chan, 2001; Gao et al., 2018; Wang et al., 2019) measured the ERH of Na_2_C_4_H_4_O_4_, and it was determined to be 46-59% at 296-298 K, showing relatively good agreement. Moreover, Gao et al. (2018) measured the ERH of Na_2_C_4_H_4_O_4_ as a function of temperature, and found that it increased with temperature from 34-38% at 270 K to 50-57% at 296 K.

**Preferred values at 298 K for Na_2_C_4_H_4_O_4_:**

DRH: 63-79%

ERH: 46-59%

**References:**

Gao, X., Zhang, Y., and Liu, Y.: Temperature-dependent hygroscopic behaviors of atmospherically relevant water-soluble carboxylic acid salts studied by ATR-FTIR spectroscopy, Atmospheric Environment, 191, 312-319, 2018.

Ma, Q., Ma, J., Liu, C., Lai, C., and He, H.: Laboratory study on the hygroscopic behavior of external and internal C2-C4 dicarboxylic acid-NaCl mixtures, Environ Sci Technol, 47, 10381-10388, 2013.

Peng, C. G., and Chan, C. K.: The water cycles of water-soluble organic salts of atmospheric importance, Atmospheric Environment, 35, 1183-1192, 2001.

Wang, N., Jing, B., Wang, P., Wang, Z., Li, J., Pang, S., Zhang, Y., and Ge, M.: Hygroscopicity and Compositional Evolution of Atmospheric Aerosols Containing Water-Soluble Carboxylic Acid Salts and Ammonium Sulfate: Influence of Ammonium Depletion, Environmental Science & Technology, 53, 6225-6234, 2019.

Wu, Z. J., Nowak, A., Poulain, L., Herrmann, H., and Wiedensohler, A.: Hygroscopic behavior of atmospherically relevant water-soluble carboxylic salts and their influence on the water uptake of ammonium sulfate, Atmospheric Chemistry and Physics, 11, 12617-12626, 2011.

## Na_2_C_4_H_2_O_4_ (sodium maleate)

| Reference | *T* (K) | *D* | DRH (%) | ERH (%) | Techniques/Comments |
| --- | --- | --- | --- | --- | --- |
| Peng and Chan, 2001 | 298 | ~20 μm | n. o. | n. o. | EDB |
| Wu et al., 2011 | 293 | 100 nm | n. o. | - | HTDMA |

**Comments:**

Two studies (Peng and Chan, 2001; Wu et al., 2011) investigated hygroscopic properties of of Na_2_C_4_H_2_O_4_ particles, and found that these particles showed continueous water uptake with the increase in RH at 293-298 K, thus no DRH was reported.

Peng and Chan (2001) also measured the ERH of Na_2_C_4_H_2_O_4_, and found that no efflorescence took place with the decrease in RH at 298 K.

**Preferred values at 298 K for Na_2_C_4_H_2_O_4_:**

DRH: no preferred value

ERH: no preferred value

**References:**

Peng, C. G., and Chan, C. K.: The water cycles of water-soluble organic salts of atmospheric importance, Atmospheric Environment, 35, 1183-1192, 2001.

Wu, Z. J., Nowak, A., Poulain, L., Herrmann, H., and Wiedensohler, A.: Hygroscopic behavior of atmospherically relevant water-soluble carboxylic salts and their influence on the water uptake of ammonium sulfate, Atmospheric Chemistry and Physics, 11, 12617-12626, 2011.

## (NH_4_)_2_C_4_H_4_O_6_ (ammonium tartrate)

| Reference | *T* (K) | *D* | DRH (%) | ERH (%) | Techniques/Comments |
| --- | --- | --- | --- | --- | --- |
| Apelblat and Korin, 2003 | 278 | - | 85.9 | - | Nonisopiestic method |
|  | 283 |  | 86.0 |  |  |
|  | 288 |  | 85.9 |  |  |
|  | 293 |  | 85.7 |  |  |
|  | 298 |  | 85.4 |  |  |
|  | 303 |  | 85.0 |  |  |
|  | 308 |  | 84.5 |  |  |
|  | 313 |  | 84.0 |  |  |
|  | 318 |  | 83.4 |  |  |
|  | 323 |  | 82.7 |  |  |
| Wu et al., 2011 | 293 | 100 nm | n. o. | - | HTDMA |

**Comments:**

The DRH of (NH_4_)_2_C_4_H_4_O_6_ was measured by two studies (Apelblat and Korin, 2003; Wu et al., 2011). Apelblat and Korin (2003) found that it decreased with temperature from 85.9% at 278 K to 85.4% at 298 K and to 82.7% at 323 K; in contrast, Wu et al. (2011) observed continuous water uptake with the increase in RH at 293 K, and thus no DRH was reported.

**Preferred values at 298 K for (NH_4_)_2_C_4_H_4_O_6_:**

DRH: 85-86%

ERH: no preferred value

**References:**

Apelblat, A., and Korin, E.: The molar enthalpies of solution and vapour pressures of saturated aqueous solutions of some ammonium salts, Journal of Chemical Thermodynamics, 35, 699-709, 2003.

Wu, Z. J., Nowak, A., Poulain, L., Herrmann, H., and Wiedensohler, A.: Hygroscopic behavior of atmospherically relevant water-soluble carboxylic salts and their influence on the water uptake of ammonium sulfate, Atmospheric Chemistry and Physics, 11, 12617-12626, 2011.

## Na_2_C_4_H_4_O_6_ (sodium tartrate)

| Reference | *T* (K) | *D* | DRH (%) | ERH (%) | Techniques/Comments |
| --- | --- | --- | --- | --- | --- |
| Wu et al., 2011 | 293 | 100 nm | n. o. | - | HTDMA |

**Comments:**

Wu et al. (2011) observed continuous water uptake with the increase in RH for sodium tartrate at 293 K, thus no DRH was reported.

**Preferred values at 298 K for Na_2_C_4_H_4_O_6_:**

DRH: no preferred value

ERH: no preferred value

**References:**

Wu, Z. J., Nowak, A., Poulain, L., Herrmann, H., and Wiedensohler, A.: Hygroscopic behavior of atmospherically relevant water-soluble carboxylic salts and their influence on the water uptake of ammonium sulfate, Atmospheric Chemistry and Physics, 11, 12617-12626, 2011.
